# Target-driven machine learning–enabled virtual screening (TAME-VS) enables prioritization of AKR1C3 inhibitors

**DOI:** 10.1038/s42004-026-02092-6

**Published:** 2026-06-15

**Authors:** Longjiang Qiao, Xinyu Li, Shuaishuai Xing, Qianwen Guan, Kun Ning, Weijie Han, Luyu Zhang, Anlan Dai, Weiqi Yang, Yinhang Liu, Jason J. Kwon, Xin Gao, Haopeng Sun, Yuemin Bian

**Affiliations:** 1 Digital Medical Research Institute, School of Medicine, Shanghai University, Shanghai, China; 2 Department of Pharmaceutical Science, School of Medicine, Shanghai University, Shanghai, China; 3 School of Pharmacy, China Pharmaceutical University, Nanjing, China; 4 Department of Bioengineering, University of California, Berkeley, Berkeley, CA USA; 5 Department of Bioengineering and Therapeutic Sciences, University of California, San Francisco, San Francisco, CA USA; 6 Cancer Program, Broad Institute of MIT and Harvard, Cambridge, MA USA; 7 Shanghai Universal Medical Diagnostic Imaging Center, Shanghai, China; 8 State Key Laboratory of Natural Medicines, China Pharmaceutical University, Nanjing, China; 9 Shanghai Engineering Research Center of Organ Repair, Joint International Research Laboratory of Biomaterials and Biotechnology in Organ Repair (Ministry of Education), Shanghai, China

**Keywords:** Virtual screening, Drug discovery and development, Cheminformatics, Cheminformatics, Lead optimization

## Abstract

Machine learning–guided virtual screening has the potential to accelerate early-stage drug discovery, but its practical utility is often limited by uncertain generalization to different chemical scaffolds and by insufficient prospective evaluation under realistic experimental constraints. To address these challenges, we conduct a target-driven virtual screening study using TAME-VS against aldo–keto reductase 1C3 (AKR1C3), an enzyme implicated in cancer progression and chemoresistance. By integrating automated target expansion, bioactivity data acquisition, and supervised learning, we construct classification models that leverage homolog-derived information to address data sparsity. Retrospective validation using inhibitor series absent from the training data demonstrates generalization to chemically different scaffolds beyond similarity-based prioritization. Prospective validation on newly designed derivatives shows that model-guided prioritization enriches active inhibitors relative to rational medicinal chemistry design alone under constrained synthesis capacity. **S34-1035** and **S34-1040** were further identified as potent and selective AKR1C3 inhibitors, and were shown to restore doxorubicin sensitivity in resistant breast cancer cells, establishing functional relevance. Building on these findings, a publicly accessible online server with a graphical user interface was further developed for the validated workflow of TAME-VS, enabling reproducible application by the broader community.

**Figure:**
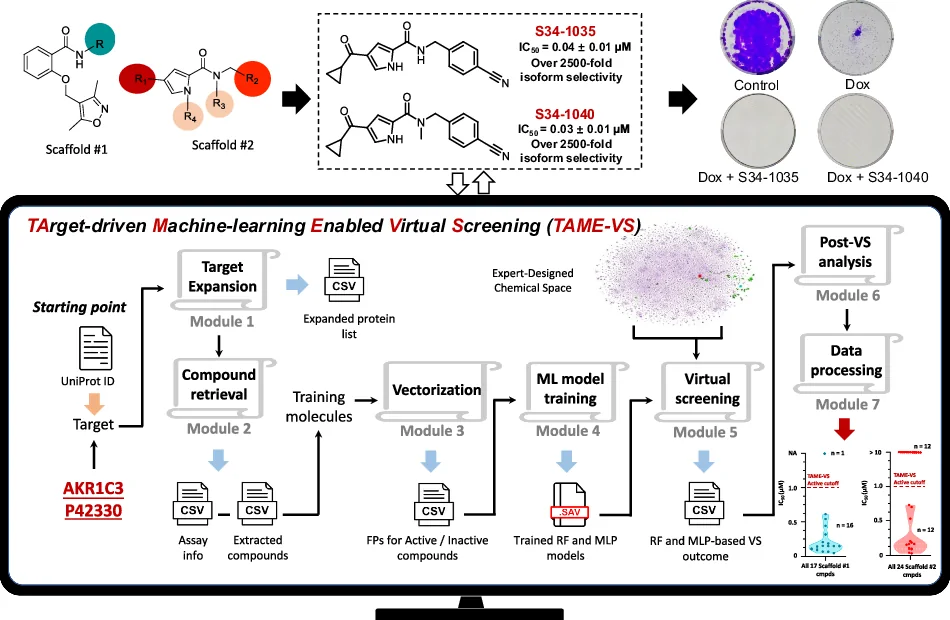

## Abstract (web summary)

Machine learning–guided virtual screening can revolutionize early-stage drug discovery, yet its effectiveness is often hindered by limited generalization and evaluation constraints. Here, the authors employ TAME-VS to target AKR1C3, demonstrating enhanced inhibitor prioritization and functional relevance by generalization to chemically different scaffolds and prospective evaluation under constrained synthesis capacity.

## Introduction

Virtual screening is increasingly expected to do more than retrieve known chemotypes^1,2^. It is now asked to identify actionable chemical matter under experimental constraints, often early in projects where little curated data exists. The rise of public bioactivity repositories^3–7^ and machine learning has created the impression that ligand-based virtual screening can be readily deployed across targets^8–11^, yet practical translation remains inconsistent. Molecular fingerprints, particularly two-dimensional representations, remain among the most widely used descriptors in ligand-based virtual screening due to their efficiency and strong empirical performance, including in identifying compounds with novel scaffolds, and are often integrated with complementary information, such as pharmacophore or structural features, in modern workflows^12^. Two foundational questions are often left unresolved in real discovery settings that (1) does a model generalize beyond familiar chemical neighborhoods, and (2) does it meaningfully improve experimental efficiency under synthesis constraints.

The first question concerns generalization to chemically novel scaffolds, which is the central promise of learning-based screening but also its most frequent failure mode. Models can be trained and evaluated on datasets where the train–test split remains chemically proximal by random splitting, scaffold partitioning, or inadvertent leakage through analog series^13–15^. Such that model performance may largely reflect interpolation within known structure–activity neighborhoods. Under these conditions, a classifier can appear successful while behaving similarly to similarity search, and “high performance” does not necessarily predict value in practice, where the goal is often to identify activity in previously untested chemotypes. Rigorous evaluation of novelty generalization, therefore, requires validation sets that are not merely held out, but temporally or provenance-separated from the data used in training, such that the test compounds represent chemical matter that was not available, conceptually and literally, at training time.

The second question concerns the statistics of small numbers and the decision-making reality of early discovery. Regardless of whether models are applied to millions of purchasable molecules or to a medicinal chemistry design set, experimental testing typically covers only a small fraction of candidates, and the perceived “hit rate” is often inferred from hundreds or even tens of compounds^16–18^. This practice makes conclusions sensitive to sampling variance and obscures whether a model provides value beyond expert design. Moreover, meaningful baseline comparisons, such as the intrinsic activity rate of an expert-designed compound set, are not always available in AI-enabled screening studies involving prospective prioritization, which can obscure the contribution of model guidance to active compound enrichment. In practice, what determines success is not model metrics, but whether a method can rank compounds so that the first batch of synthesized molecules are more likely to succeed than a rational baseline, and whether this enrichment remains controllable as users change the definition of hit and the decision threshold.

We previously introduced TAME-VS^19^ as a target-driven machine learning framework intended to lower the operational barrier for virtual screening by automating target expansion, compound retrieval, and model training starting from minimal user input. However, a platform-level contribution does not, by itself, establish practical utility. The field requires demonstrations that such workflows can (i) generalize to chemotypes that were not part of the bioactivity snapshot used for training, and (ii) prospectively enrich actives under synthesis constraints, relative to a rational medicinal chemistry baseline, rather than only improving retrospective metrics. Here, we address these two questions using aldo–keto reductase family 1 member C3^20^ (AKR1C3) as a challenging and clinically relevant model system. AKR1C3 is implicated in cancer progression and chemoresistance through its metabolic roles and detoxification of reactive intermediates, and inhibition of AKR1C3 has been proposed as a strategy to restore sensitivity to chemotherapeutics^21^. At the same time, AKR1C3 belongs to a closely related aldo–keto reductase (AKR) family^22,23^ with substantial sequence and functional similarity, creating realistic complications for data curation, selectivity, and model transfer. This combination makes AKR1C3 a stringent setting to evaluate whether a target-driven ML workflow can move beyond similarity-driven prioritization while remaining usable under practical constraints. Recently, a structure-based deep learning approach using the AtomNet model identified AKR1C3 inhibitors through virtual screening against a crystallographic binding site, followed by experimental validation and demonstration of synergistic effects with chemotherapeutic agents^24^. Such studies highlight the potential of AI-driven screening to expand chemical diversity and uncover biologically active scaffolds in this target class.

In this work, we apply TAME-VS to an end-to-end AKR1C3 discovery campaign designed explicitly around the two limitations above. First, we perform retrospective validation using inhibitor series published after the bioactivity snapshot used for training, providing a stringent test of generalization to chemotypes beyond familiar chemical neighborhoods. Second, we conduct prospective validation on newly designed derivatives across two distinct scaffolds, synthesizing and experimentally evaluating the full designed set to establish a rational baseline and quantify the model-added enrichment achieved by TAME-VS prioritization. Third, we demonstrate that prioritized inhibitors not only potently inhibit AKR1C3 enzymatic activity but also restore doxorubicin sensitivity in resistant breast cancer cells, linking computational prioritization to disease-relevant phenotypic impact. Finally, we advance the original terminal-based implementation to a publicly accessible online server with a graphical user interface, enabling reproducible application of the validated workflow by the broader community.

## Results

### Overview of the TAME-VS workflow for AKR1C3 hit identification

**Fig. 1:**
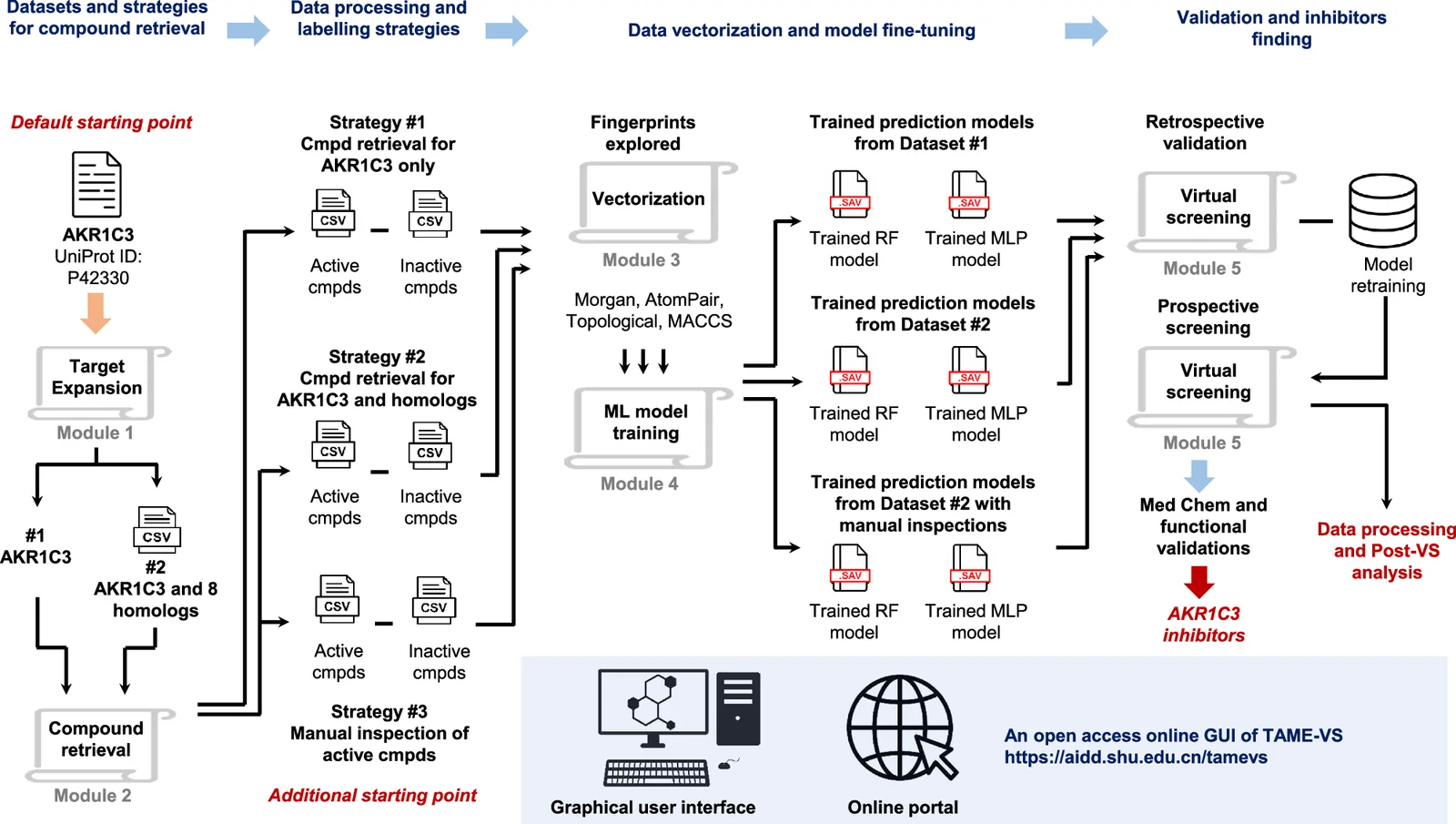
The schematic illustration of applying TAME-VS on AKR1C3 hit finding. Starting from the UniProt accession of AKR1C3, TAME-VS performs automated target expansion to identify homologous enzymes, followed by compound retrieval and activity-based labeling to construct training datasets. Three data-construction strategies are implemented, differing in target scope and curation level. Molecular representations are generated and used to train supervised classification models, which are subsequently evaluated by retrospective validation on unseen inhibitor series and by prospective prioritization of newly designed compounds. The workflow further supports iterative model updating and experimental feedback. An online server with a graphical user interface enables end-to-end execution of the pipeline.

Our previously developed TAME-VS platform is a modular, machine-learning-integrated framework that enables bespoke hit-identification campaigns for user-defined protein targets. Starting solely from the UniProt accession number of AKR1C3 (P42330), the platform automatically initiates target-related data acquisition (Fig. 1). Through the Target Expansion module, AKR1C3 and eight homologous enzymes sharing >40% sequence similarity were identified using BLAST-based similarity searches. These targets were subsequently processed through the Compound Retrieval module to extract reported bioactive molecules, which were curated and categorized into active or inactive ligands based on assay type and activity cutoff criteria. To construct high-quality training sets, we implemented three complementary strategies, exploring: (1) compounds associated with AKR1C3 alone; (2) compounds collected from AKR1C3 and its homologs, which is the default usage of the TAME-VS; and (3) the combined dataset supplemented with a manual inspection step to refine the active ligand list. Training molecules were vectorized using multiple fingerprinting schemes in the Vectorization module, followed by supervised classification in the ML Model Training module. Iterative exploration of fingerprint choices and algorithms yielded three ensembles of optimized random forest (RF) and multilayer perceptron (MLP) models corresponding to the three training-set strategies. Retrospective validation was then conducted using two series of established AKR1C3 inhibitors^25,26^ (bearing a pyrrole-amide scaffold and an aryl-isoxazole scaffold), confirming that the trained models could reliably identify preferred chemical scaffolds, including those structurally distinct relative to the training data. After incorporating these inhibitors into the training set, prospective virtual screening was performed with newly optimized compounds derived from chemotypes bearing a benzamide scaffold and a pyrrole-carboxamide core, leading to the synthesis and biological evaluation of prioritized candidates. This workflow ultimately yielded **S34-1035** and **S34-1040**, highly potent AKR1C3 inhibitors.

To broaden accessibility and facilitate interactive use beyond command-line execution, we further developed an open-access online implementation of TAME-VS (https://aidd.shu.edu.cn/tamevs), featuring an intuitive graphical user interface. This portal enables streamlined execution of the entire workflow—from target entry and data retrieval to model training and virtual screening—thus supporting wider adoption of the TAME-VS platform for early-stage hit discovery.

### Building the AKR1C3 chemical knowledge base and deriving robust predictive models

**Fig. 2:**
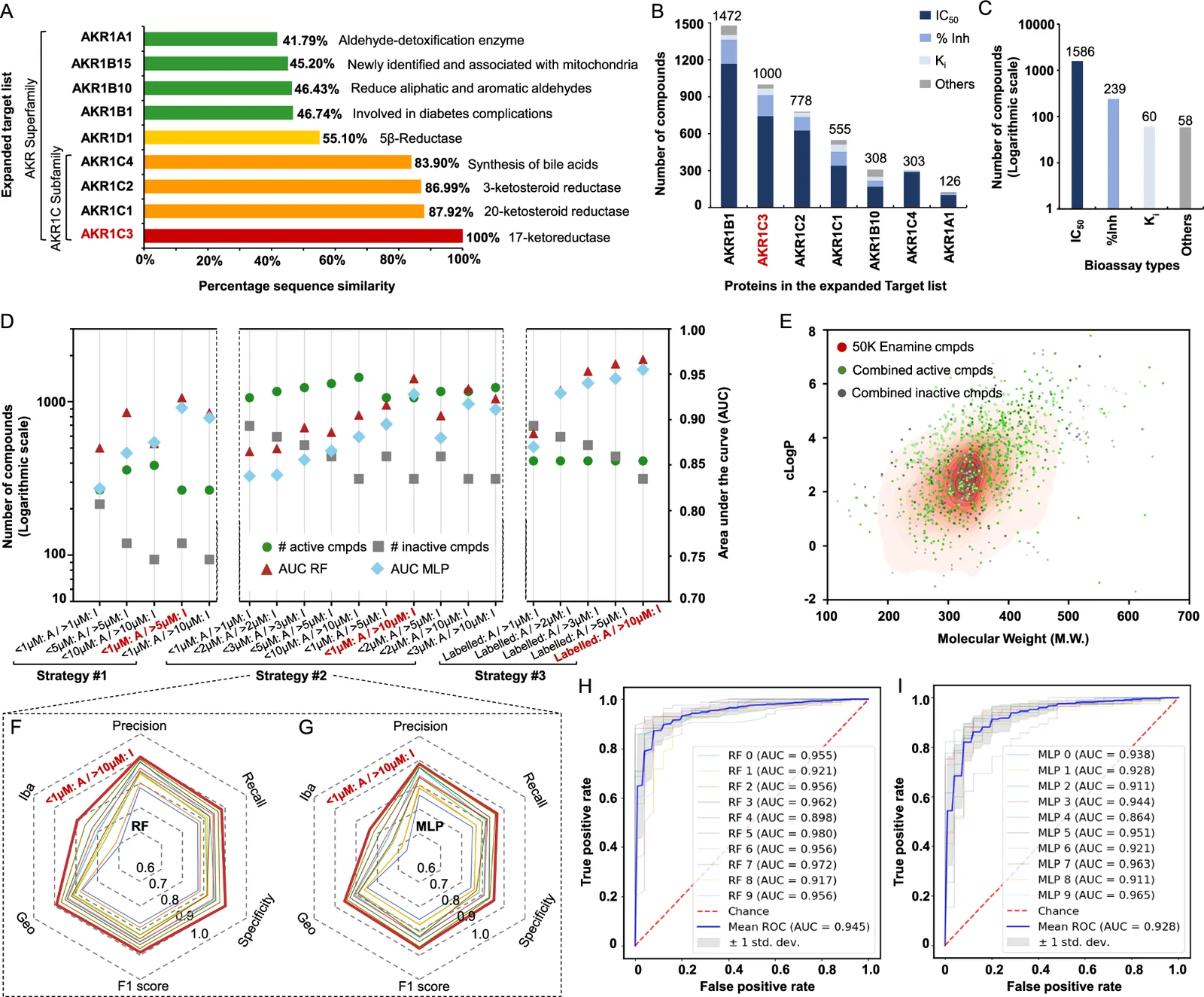
Cheminformatics data acquisition and model training strategies. **A** Target expansion results for AKR1C3, showing homologous aldo–keto reductase family members identified by sequence similarity and included for compound retrieval. **B** Number of bioactive compounds retrieved for AKR1C3 and each homologous target from the ChEMBL database. **C** Distribution of assay types across the retrieved bioactivity records. **D** Model performance (ROC–AUC) of random forest (RF) and multilayer perceptron (MLP) classifiers trained under different active–inactive cutoff definitions across three data-construction strategies. **E** Molecular weight versus cLogP distribution of active and inactive AKR1C3 compounds, shown in the context of 50,000 randomly selected drug-like Enamine compounds. Radar plots summarizing multiple classification metrics (precision, recall, specificity, F1 score, geometric mean, and index of balanced accuracy) for RF (**F**) and MLP (**G**) models under representative cutoff settings. Receiver operating characteristic (ROC) curves for RF (**H**) and MLP (**I**) models obtained by ten-fold stratified cross-validation under the strategy #2, with actives defined as IC_50_ < 1 µM and inactives defined as IC_50_ > 10 µM.

Using only the UniProt accession number of AKR1C3 (P42330) as input, the Target Expansion module identified eight homologous enzymes within the AKR superfamily sharing >40% sequence identity, including three closely related isoforms in the AKR1C subfamily—AKR1C1 (87.9%), AKR1C2 (87.0%), and AKR1C4 (83.9%) (Fig. 2A). Subsequent evaluation by the Compound Retrieval module showed that AKR1C3 and six of its homologs were associated with between 126 and 1479 compounds with reported bioactivity data (Fig. 2B and Supplementary Table 1). Across these targets, activities were reported in 23 assay formats, with IC_50_, percentage inhibition, and Kᵢ values being the most frequent (Supplementary Table 2). After merging the retrieved compounds, IC_50_ measurements, which represent functional inhibitory potency, accounted for 1586 entries, more than four times the combined number of all other assay types (Fig. 2C). We therefore focused on molecules with reported IC_50_ values for subsequent vectorization and classification model training.

As outlined in the overview of the workflow, three complementary strategies were implemented to construct training sets and systematically compare model behavior (Fig. 1). For each strategy, combinations of activity cutoffs for active and inactive labels were explored after converting molecules into Morgan fingerprints (Fig. 2D). In strategy #1, using IC_50_ < 1 µM to define actives and IC_50_ > 5 µM to define inactives yielded the highest area under the ROC curve (AUC) values of 0.924 and 0.913 for both RF and MLP models. In strategy #2, optimal performance (AUC values of 0.945 and 0.928 for RF and MLP models) was achieved when actives were defined as IC_50_ < 1 µM and inactives as IC_50_ > 10 µM, whereas in strategy #3, manually labeled actives combined with IC_50_ > 5 µM as the inactive cutoff produced the best-performing RF (AUC value of 0.966) and MLP (AUC value of 0.955) models. Across all three training-set strategies, a consistent trend emerged that larger potency gaps between the thresholds used to label active and inactive compounds produced models with superior discriminative performance (Fig. 2D). This behavior is expected, as widening the activity gap reduces ambiguity near the decision boundary and minimizes the influence of borderline compounds whose potency may be sensitive to assay variability. As a result, training sets derived from more stringent active–inactive separations yielded clearer structure–activity distinctions and correspondingly higher AUC values for both RF and MLP classifiers. Mapping molecular weight versus cLogP for all AKR1C3 actives and inactives showed substantial overlap in size and lipophilicity (Fig. 2E). When contextualized against 50,000 drug-like Enamine compounds, the collected ligands cluster within the general drug-like chemical space as desired while also extending beyond the classical rule-of-five region.

Using strategy #2 as an example, radar plots further illustrate model behavior across six additional performance metrics, including precision, recall, specificity, F1 score, geometric mean, and index of balanced accuracy (Iba) for both RF and MLP classifiers (Fig. 2F, G). The model trained with IC_50_ < 1 µM and IC_50_ > 10 µM consistently outperforms alternative cutoffs across these metrics, while strategies #1 and #3 also achieve solid performance (Supplementary Fig. S1A–D). Ten-fold cross-validation was applied throughout model training to assess generalization, and both RF and MLP models exhibited robust and reproducible ROC curves with narrow confidence intervals with standard deviation of 0.028, 0.025, and 0.015 for RF and 0.031, 0.029, and 0.023 for MLP, for three strategies, respectively. (Figs. 2H, I, and S1E–H). Notably, the ROC curves from strategy #1 exhibit wider confidence intervals relative to strategies #2 and #3, reflexive of the smaller dataset used in strategy #1, which naturally introduces higher variance during cross-validation. In contrast, strategies incorporating homolog-derived compounds or manually curated labels generate more stable models with tighter confidence bounds. In total, strategy #1 used 266 actives and 120 inactives, strategy #2 used 1066 actives and 315 inactives, and strategy #3 used 412 actives and 315 inactives, with their overlaps summarized by Venn diagrams (Supplementary Fig. S1I, J). While Morgan fingerprints were used as the default representation, TAME-VS also supports AtomPair, topological, and MACCS fingerprints. A comprehensive evaluation across the three strategies and four fingerprint types, each with ten-fold cross-validation for both RF (Supplementary Table 3 and Supplementary Fig. S1K) and MLP models (Supplementary Table 4 and Supplementary Fig. S1J), revealed that no statistically significant differences (Holm–Bonferroni step-down correction^27^) in predictive performance were observed between fingerprint types after correction for multiple comparisons (Supplementary Data 1), despite modest variations in mean metric values. These results indicate that model performance is primarily driven by training set composition and label definition rather than the specific fingerprint representation used.

### Retrospective validation confirms the ability of TAME-VS to recognize preferred AKR1C3 inhibitors with distinct scaffolds

**Fig. 3:**
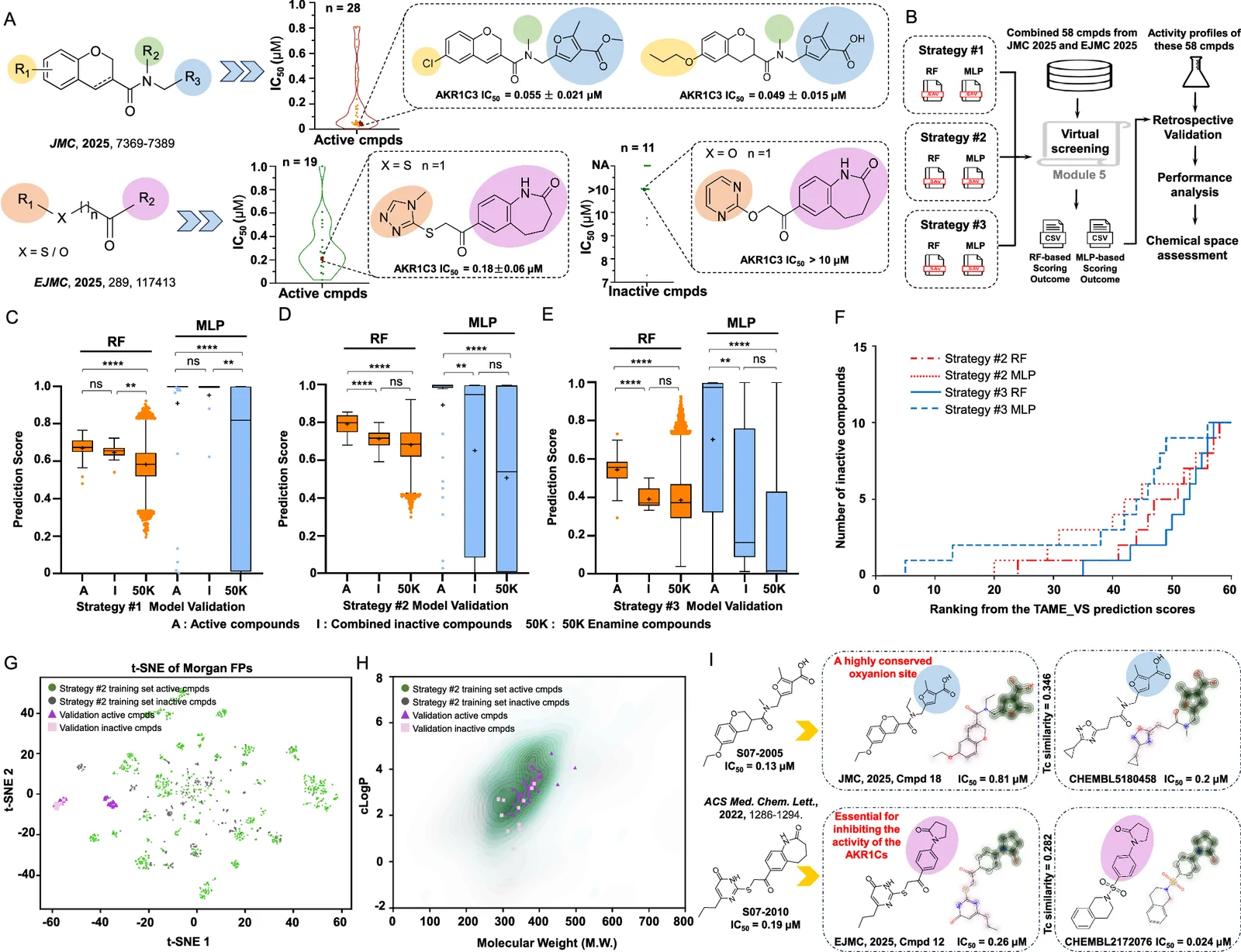
TAME-VS recognizes preferred chemicals with distinct scaffolds. **A** Chemical scaffolds and representative activity distributions of two externally reported AKR1C3 inhibitor series derived from S07-2005 and S07-2010. Violin plots summarize IC_50_ values of active and inactive derivatives, with representative compounds highlighted. **B** Schematic of retrospective validation workflow. RF and MLP models trained under three data-construction strategies were applied to score the combined validation set. Prediction score distributions for RF and MLP models trained under strategy #1 (**C**), strategy #2 (**D**), and strategy #3 (**E**). **F** Cumulative ranking curves showing the number of inactive compounds encountered as a function of rank when validation compounds are ordered by prediction score. **G** t-SNE visualization of Morgan fingerprint space for training-set and retrospective validation compounds. **H** Distribution of molecular weight and cLogP for training and validation compounds. **I** Structural comparison of representative validation compounds and training-set actives illustrating shared activity-relevant substructural motifs despite low overall Tanimoto similarity. In **C–E**, comparison between two groups was performed using an unpaired, one-tailed Student’s *t* test. For **C**: RF ns *P* = 0.1111, **P = 0.0077, and ****P < 0.0001; MLP ns *P* = 0.3014, **P = 0.0020, and ****P < 0.0001. For **D**: RF ns *P* = 0.1287, ****P < 0.0001; MLP ns *P* = 0.1439, **P = 0.0092, and ****P < 0.0001. For **E**: RF ns *P* = 0.4456, ****P < 0.0001; MLP ns *P* = 0.1656, **P = 0.0050, and ****P < 0.0001.

To further assess the generalizability of the trained classification models beyond machine-learning metrics, we conducted retrospective validations using our two recently reported series of potent AKR1C3 inhibitors whose structures were not present in the ChEMBL snapshot used during data retrieval (accessed 12/20/2024, ChEMBL35^28^), which contains publications only prior to 12/01/2024. These two series, derived from **S07-2005**^26,29^ and **S07-2010**^25,29^ and published in 2025, therefore represent completely unseen chemical matter with distinct scaffold architectures (Fig. 3A). **S07-2005** occupies the oxyanion site (OS), subpocket 1 (SP1), and subpocket 2 (SP2) of AKR1C3 (Supplementary Fig. 2A). Structural optimization was performed at three regions: (i) R_1_, the benzodihydropyran fragment, to enhance SP2 occupancy and modulate charge distribution; (ii) R_2_, the amide linkage, to probe substituent effects on potency and selectivity; and (iii) R_3_, the furan motif, to extend interactions within SP1. A total of 28 derivatives with IC_50_ < 1 µM were reported (Fig. 3A and Supplementary Table 5). Docking studies of **S07-2010** indicated binding primarily within SP2 and SP3 (Supplementary Fig. 2B). Modifications explored size tolerance at R_1_ and R_2_, linker length, and sulfur-to-oxygen substitutions. Nineteen derivatives displayed IC_50_ < 1 µM, whereas eleven exhibited IC_50_ > 10 µM (Fig. 3A and Supplementary Table 6). In total, 47 active and 11 inactive molecules were used as a retrospective validation set (Fig. 3B).

We applied RF and MLP models trained under the three data-construction strategies. In each case, 50K Enamine Diversity compounds were scored in parallel as a background distribution to approximate model response to unrelated drug-like compounds. Models derived from strategy #1 assigned significantly higher scores to the 58 validation molecules (0.6652 ± 0.0567 for RF and 0.9178 ± 0.2473 for MLP) relative to random Enamine compounds (0.5819 ± 0.0881 for RF and 0.5599 ± 0.4532 for MLP); however, these models did not statistically differentiate active (0.6696 ± 0.0586 for RF and 0.9096 ± 0.2694 for MLP) from inactive (0.6463 ± 0.0454 for RF and 0.9532 ± 0.1150 for MLP) validation molecules (Fig. 3C). In contrast, RF and MLP models trained under strategy #2, which is the default TAME-VS workflow, were able to score active molecules (0.7916 ± 0.0519 for RF and 0.8929 ± 0.2505 for MLP) significantly higher than inactive molecules (0.7125 ± 0.0559 for RF and 0.6508 ± 0.4552 for MLP) as well as the random background distribution (0.6816 ± 0.0903 for RF and 0.5072 ± 0.4479 for MLP) (Fig. 3D). Models generated using strategy #3, which incorporated manual curation of active ligands, produced similar qualitative behavior, successfully separating active (0.5426 ± 0.0826 for RF and 0.7012 ± 0.3917 for MLP) from inactive (0.3907 ± 0.0527 for RF and 0.3532 ± 0.3807 for MLP) molecules and from background compounds (0.3855 ± 0.1265 for RF and 0.2450 ± 0.3647 for MLP) (Fig. 3E). In both strategies #2 and #3, inactive validation compounds and random drug-like compounds were not separable from one another, confirming that the learned patterns are activity-related. To quantify ranking behavior, cumulative ranking curves were generated from model prediction scores (Figs. 3F and S2C). For both RF and MLP models derived from strategies #2 and #3, active compounds consistently accumulated at earlier ranks than inactive compounds. Using the RF model from strategy #3 as an example, 93.6% of active compounds appeared within the top 47 ranked molecules, whereas 72.7% of inactive compounds were positioned within the bottom 11 (Fig. 3F). These observations confirm that the trained models not only assign higher average scores to active molecules but also effectively prioritize them in a prospective ranking context.

To examine whether the separation arises from classical structural or physicochemical clustering, we visualized the chemical space using t-SNE embeddings. Active and inactive compounds in the training sets of strategies #2 and #3 are interspersed, reflecting the presence of multiple scaffold families with overlapping structural features (Fig. 3G). As expected, none of the validation molecules overlap with the training distribution because both series introduce distinct scaffolds. Within the validation set itself, the two chemical series appear as distinguishable clusters, but active and inactive members overlap considerably. Tanimoto similarity between retrospective validation compounds and the training set was assessed with an average similarity score of 0.137 (Supplementary Fig. S3). The highest similarity score of 0.963 was contributed by **S07-2010** itself. Analysis of molecular weight, cLogP, polar surface area, and other physicochemical descriptors revealed substantial overlap between training and validation sets, and no clear segregation between actives and inactives (Figs. 3H and S2D). These findings indicate that conventional structure- or property-based similarity cannot explain the activity separation observed in model predictions. Instead, the TAME-VS models captured recurring substructural patterns associated with AKR1C3 engagement (Fig. 3I). Although neither **S07-2005** nor **S07-2010** scaffolds (except for themselves) were represented in the training data, both contain key moieties, including oxygen-bearing motifs, which are required for anchoring within the oxyanion site and hydrophobic substituent patterns that are aligned with SP2/SP3. These key moieties were enriched in reported active ligands and therefore learned by the classification models. This enabled the models to prioritize potent derivatives despite their low Tanimoto similarity to any training compound, in contrast to traditional similarity-based methods. Collectively, these results demonstrate that TAME-VS can generalize learned activity-relevant features to distinct chemical frameworks and can effectively guide early-stage hit identification.

### Prospective validation of TAME-VS in prioritizing newly designed AKR1C3 inhibitors

**Fig. 4:**
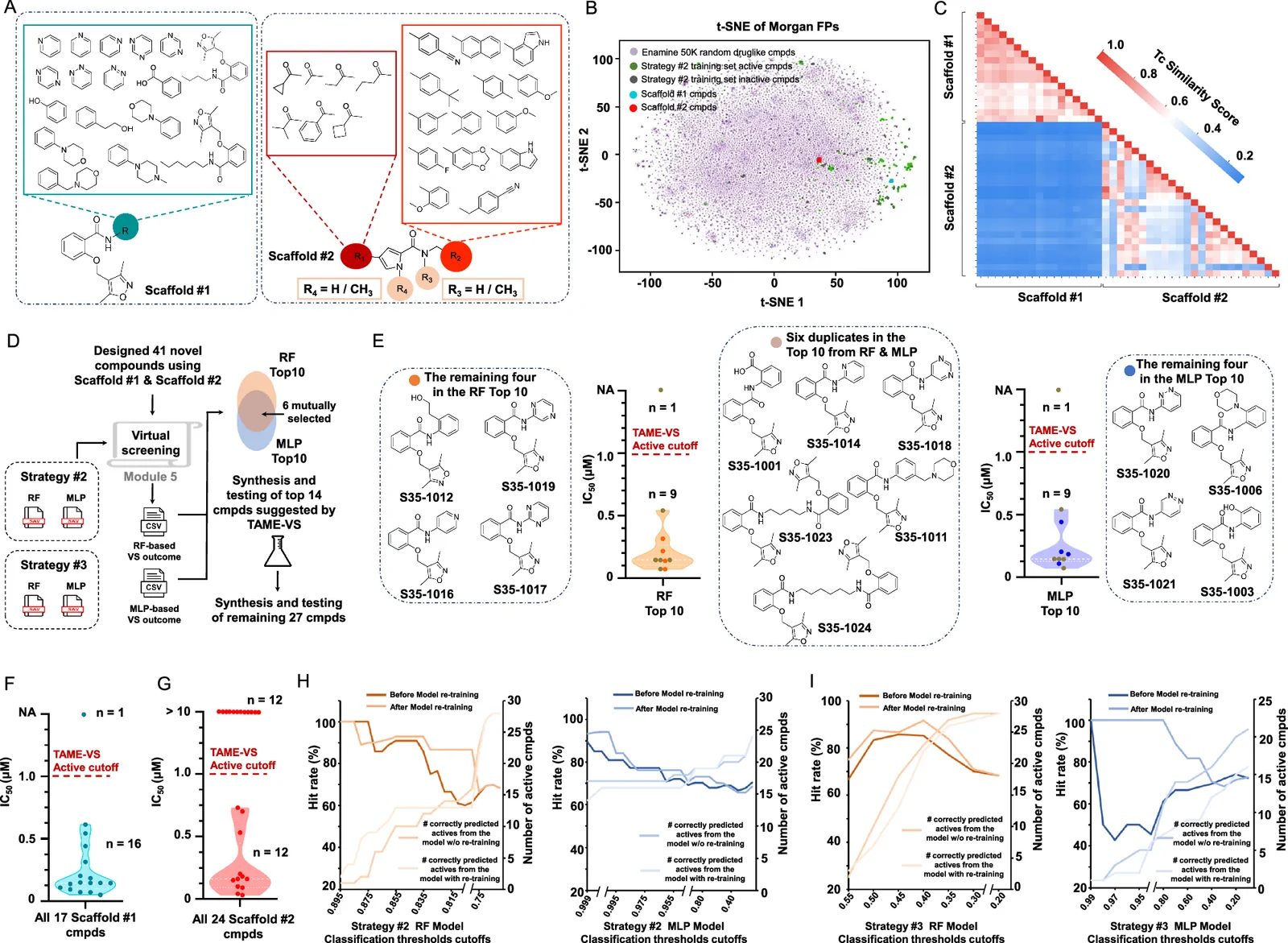
TAME-VS prioritizes derivatives designed for AKR1C3. **A** Derivatives design of two AKR1C3 inhibitor scaffolds. Scaffold #1 is a benzamide core bearing a (3,5-dimethylisoxazol-4-yl)methoxy substituent, and Scaffold #2 is a pyrrole-carboxamide core with four tunable positions (R1–R4). **B** t-SNE visualization of Morgan fingerprint space showing newly designed derivatives from Scaffold #1 and Scaffold #2 relative to AKR1C3 training-set actives and inactives and to 50,000 random drug-like Enamine compounds. **C** Pairwise Tanimoto similarity heatmap illustrating intra- and inter-scaffold structural similarity among newly designed derivatives from Scaffold #1 and Scaffold #2. **D** Prospective virtual screening and prioritization workflow. **E** Chemical structures of compounds prioritized by the RF and MLP models. Compounds uniquely selected by each model are shown at the sides, and compounds jointly prioritized by both models are shown in the center. IC_50_ distribution of 17 synthesized Scaffold #1 compounds (**F**), and 24 synthesized Scaffold #2 compounds (**G**) against AKR1C3, with the TAME-VS activity cutoff indicated. Hit-rate curves for RF and MLP models from strategies #2 (**H**) and #3 (**I**) showing the relationship between classification threshold, hit rate, and the number of correctly predicted active compounds before and after model retraining using additional validated data.

Encouraged by the retrospective validation results, we next performed a prospective validation to assess whether TAME-VS could prioritize active compounds from newly designed chemical series. Two scaffolds were selected for compound design (Fig. 4A): a benzamide scaffold bearing a (3,5-dimethylisoxazol-4-yl)methoxy substitution (Scaffold #1) and a pyrrole carboxamide scaffold (Scaffold #2). Scaffold #1 was derived from our previously identified hit molecule **S07-2008**^29^. This scaffold was selected based on its validated pharmacophore, in which a benzamide carbonyl anchors at the oxyanion site (Tyr55/His117/NADP⁺), while an isoxazolyl ether moiety occupies the SP1/SP2 hydrophobic subpockets, as confirmed by co-crystal structures with AKR1C3^30^. Notably, SP1 is a characteristic subpocket of AKR1C3, and interactions within this region are critical for achieving inhibitor selectivity. Importantly, this scaffold avoids the presence of a free carboxylic acid, thereby circumventing MCT1-dependent cellular uptake limitations associated with classical NSAID-derived inhibitors, while also eliminating off-target cyclooxygenase (COX) inhibition^20^. Scaffold #2 was derived from our previously identified hit molecule **S07-2001**^29^. It was selected as a structurally distinct, non-carboxylate alternative in which the pyrrole ring serves as a rigid central linker and the N-acyl carbonyl at R1 acts as a carboxylic acid bioisostere to engage the oxyanion pocket, thereby maintaining the essential anchor interaction while improving membrane permeability^20^. The complementary structural features of these two scaffolds were designed to ensure broad coverage of the AKR1C3 binding pocket topology, particularly the differential occupation of the SP1 cavity defined by Ser118, Phe306, Phe311, and Tyr319.

For Scaffold #1, the R-group library at the aniline position was deliberately constructed to span four chemically distinct subspaces: (i) nitrogen-containing heteroaromatics (pyridines, pyrimidines, pyridazines, and triazines), selected to improve aqueous solubility, introduce additional hydrogen-bond acceptors capable of contacting Asn167 or Ser118, and reduce cytochrome P450-mediated aromatic hydroxylation; (ii) substituted phenyl rings with varying substitution patterns (ortho >  meta > para preference), motivated by the known V-shaped binding conformation of this scaffold class in which ortho substitution promotes a dihedral twist enabling simultaneous occupancy of the SP1–SP2–SP3 subpockets; (iii) halogenated phenyl groups (F, Cl), selected as electron-withdrawing, metabolically blocking substituents with minimal steric impact; and (iv) morpholine- and piperazine-containing flexible chains, included to probe the effect of ionizable nitrogen atoms on cellular permeability and to reach the deeper SP3 region.

For Scaffold #2, the R1 position (N-acyl group) was varied across small cyclic ketones (cyclopropyl, cyclobutyl, isobutyl, and acetyl), since strained carbocycles confer favorable CH–π contacts and conformational rigidity that reduces entropic binding penalties. The R2 position (C-terminal aryl group) was diversified across large fused aromatic systems (naphthyl, biphenyl, and indolyl), methylenedioxy-fused phenyl groups (with and without fluorine), and polar-substituted benzyl ethers (methoxy, cyano), in order to systematically probe π–π stacking interactions with Phe306/Phe311 and differential occupancy of the wider SP1 subpocket unique to AKR1C3 (defined by Ser118 versus Phe118 in AKR1C2). The R3 and R4 methyl/hydrogen variants at the pyrrole 3- and 4-positions were included to evaluate the effect of local steric perturbation on the planarity and dihedral angles of the central linker, which in turn governs the spatial orientation of both terminal groups.

T-SNE analysis based on Morgan fingerprints showed AKR1C3 molecules have their preferred chemical space instead of randomly distributing across the general druglike chemical space (Fig. 4B). Derivatives of Scaffold #1 and Scaffold #2 form two separated clusters, reflecting their distinct structural patterns, while remaining within the preferred chemical space learned from AKR1C3 ligands. Pairwise Tanimoto similarity analysis^31^ further confirmed low mutual similarity between the two scaffolds, with cross-scaffold similarity scores below 0.235 (Fig. 4C).

All 41 newly designed compounds were evaluated using RF and MLP models trained under strategy #2, the default setting of TAME-VS. Among the top-10 compounds independently prioritized by the RF and MLP models, six compounds were mutually selected. Combining RF- and MLP-based rankings, 14 compounds were prioritized for synthesis and biological testing (Fig. 4D). Of these, 13 compounds exhibited IC_50_ values below 1 µM, corresponding to the predefined TAME-VS activity cutoff, yielding an overall hit rate of 92.8%. Individually, the RF and MLP models achieved hit rates of 90.0% (9/10). The only inactive compound among the mutually selected six was **S35-1025**, a symmetric molecule featuring a six-carbon linker connecting two pyrrole-carboxamide units (Fig. 4E). To assess whether the strong prospective performance was specific to strategy #2, we further examined predictions from strategy #3 models, after excluding compounds initially prioritized in strategy #2. Sixteen compounds were prioritzed for synthesis and biological testing (Supplementary Fig. S4A). Remarkably, two Scaffold #2 compounds **S34-1035** and **S34-1040** were identified to exhibit AKR1C3 inhibition IC_50_ values of 0.04 ± 0.01 and 0.03 ± 0.01 µM, respectively. To exclude the possibility that the high hit rate and potency arose solely from rational scaffold selection, the remaining 27 compounds were synthesized and tested to enable an unbiased assessment across the full designed chemical space (Fig. 4D, Tables 1 and 2). In total, 28 of the 41 compounds exhibited IC_50_ values below 1 µM, corresponding to a baseline hit rate of 68.3% if compounds were randomly selected (Fig. 4F, G). The baseline hit rate of 68.3% reflects effective expert-guided medicinal chemistry design within a focused chemical space. In this context, the observed increase to 92.8% reflects preferential ranking of active compounds within an already enriched chemical space, rather than discovery from a predominantly inactive background.

**Table 1:** Inhibitory activity of Scaffold #1 analogues against AKR1C3^a, b^

Scaffold #1			
Compounds	R	AKR1C3 IC_50_ (μM)	
**S35-1014**	2-pyridine		0.15 ± 0.08
**S35-1015**	3-pyridine		0.05 ± 0.02
**S35-1016**	4-pyridine		0.07 ± 0.01
**S35-1017**	2-pyrimidine		0.31 ± 0.25
**S35-1018**	5-pyrimidine		0.07 ± 0.05
**S35-1019**	2-pyrazine		0.14 ± 0.04
**S35-1020**	3-pyridazine		0.11 ± 0.04
**S35-1021**	4-pyridazine		0.18 ± 0.08
**S35-1023**	–		0.14 ± 0.08
**S35-1001**	2-benzoic acid		0.54 ± 0.17
**S35-1003**	2-phenol		0.20 ± 0.04
**S35-1005**	3-morpholinophenyl		0.09 ± 0.04
**S35-1006**	2-morpholinophenyl		0.44 ± 0.33
**S35-1008**	3-(4-methylpiperazin-1-yl)phenyl		0.15 ± 0.06
**S35-1011**	3-(morpholinomethyl)phenyl		0.14 ± 0.02
**S35-1012**	2-(2-hydroxyethyl)phenyl		0.61 ± 0.19
**S35-1024**	–		NA

^a^All tests were expressed as the mean ± SEM of three independent experiments.

^b^NA not active. No significant inhibition (>50%) was observed at the highest tested concentration (10 µM).

**Table 2:** Inhibitory activity of Scaffold #2 analogues against AKR1C3^a^

Scaffold #2						
Compounds	R1	R2	R3	*n*	X	AKR1C3 IC_50_ (μM)
**S34-1035**	H	Cyclopropyl	1-methyl-4-benzonitrile	1	NH	0.04 ± 0.01
**S34-1040**	H	Cyclopropyl	1-methyl-4-benzonitrile	1	N-CH_3_	0.03 ± 0.01
**S34-1010**	H	Methyl	1-methyl-4-benzonitrile	1	NH	0.20 ± 0.06
**S34-1022**	H	Methyl	Naphthalen-1-yl	1	NH	0.53 ± 0.24
**S34-1023**	H	Methyl	Naphthalen-2-yl	1	NH	0.70 ± 0.05
**S34-1024**	H	Methyl	4-methyl-1H-indole	1	NH	0.73 ± 0.31
**S34-1031**	H	Ethyl	1-methyl-4-benzonitrile	1	NH	0.16 ± 0.03
**S34-1032**	H	n-propyl	1-methyl-4-benzonitrile	1	NH	0.18 ± 0.01
**S34-1033**	H	i-propyl	1-methyl-4-benzonitrile	1	NH	0.16 ± 0.01
**S34-1034**	H	Phenyl	1-methyl-4-benzonitrile	1	NH	0.09 ± 0.03
**S34-1036**	H	Cyclobutyl	1-methyl-4-benzonitrile	1	NH	0.10 ± 0.02
**S34-1038**	H	Cyclopropyl	1-methyl-4-benzonitrile	2	NH	0.15 ± 0.13
**S34-1001**	H	Methyl	1-methyl-4-methylbenzene	1	NH	>10
**S34-1037**	Methyl	Methyl	1-methyl-4-benzonitrile	1	NH	>10
**S34-1004**	H	Methyl	1-methyl-4-methoxybenzene	1	NH	>10
**S34-1002**	H	Methyl	1-methyl-3-methylbenzene	1	NH	>10
**S34-1003**	H	Methyl	1-methyl-2-methylbenzene	1	NH	>10
**S34-1005**	H	Methyl	1-methyl-3-methoxybenzene	1	NH	>10
**S34-1006**	H	Methyl	1-methyl-2-methoxybenzene	1	NH	>10
**S34-1007**	H	Methyl	1-methyl-4-fluorobenzene	1	NH	>10
**S34-1021**	H	Methyl	5-methylbenzo[d][1,3]dioxole	1	NH	>10
**S34-1025**	H	Methyl	5-methyl-1H-indole	1	NH	>10
**S34-1030**	–	–	–	–	–	>10
**S34-1013**	H	Methyl	1-methyl-4-(tert-butyl)-benzene	1	NH	>10

^a^All tests were expressed as the mean ± SEM of three independent experiments.

With all compounds synthesized and evaluated, we further analyzed the relationship between hit rate, activity definition, and classification thresholds. In addition to models from strategy #2 (Supplementary Table 7), models from strategy #3 incorporating manual curation (Supplementary Table 8) were evaluated. Models retrained by incorporating the 58 retrospective validation compounds were also examined to reflect iterative discovery cycles. As the activity cutoff became more stringent, hit rates decreased for both RF and MLP models under strategies #2 and #3, regardless of retraining (Supplementary Fig. S4). For example, using strategy #2 models without retraining, hit rates declined from 77.8% and 89.5% (IC_50_ < 1000 nM) to 16.7% and 21.0% (IC_50_ < 100 nM) for RF and MLP models, respectively (Supplementary Fig. S4B, C). After retraining, hit rates increased to 92.8% and 94.1% for RF and MLP models under the same cutoffs (IC_50_ < 1000 nM). Similar trends were observed for strategy #3 (Supplementary Fig. S4D, E). As expected, hit rates decreased as the classification threshold cutoff was lowered: hit rates were highest at the most stringent threshold and declined progressively with decreasing thresholds (Fig. 4H, I). In parallel, the absolute number of correctly predicted compounds increased, accompanied by a reduction in overall hit rate. This behavior reflects the expected tradeoff between precision and recall, where stricter classification thresholds favor higher hit rates, whereas relaxed thresholds increase the recovery of active compounds at the cost of reduced hit rate. Model retraining consistently improved hit rates, with the most pronounced effect observed for the MLP model under strategy #3 (Fig. 4I). These results illustrate how iterative incorporation of newly validated compounds can progressively improve prioritization performance, reflecting realistic model-update cycles in early-stage discovery. Together, TAME-VS can prospectively prioritize newly designed compounds with elevated hit rates relative to rational design alone, while maintaining consistent behavior across different activity definitions, classification thresholds, and model retraining cycles.

### Experimental validation confirms that newly designed AKR1C3 inhibitors restore doxorubicin sensitivity in resistant breast cancer cells

The synthesis of the above-mentioned 17 Scaffold #1 compounds is outlined in Supplementary Fig. S5A, B. The detailed synthesis and structural characterizations are specified in Supplementary Methods and Supplementary Data 2. Initially, commercially available methyl benzoate (**1**) was first reacted with 4-(chloromethyl)-3,5-dimethylisoxazole (**2**) in acetonitrile at elevated temperature using K_2_CO_3_ as base, affording intermediate **3** bearing the isoxazole moiety. Subsequent hydrolysis of the aromatic ester under alkaline conditions yielded the corresponding carboxylic acid **4**. Amide coupling of acid **4** with a series of amines (R–NH_2_) was then performed employing HATU as the coupling reagent and DIPEA as the base in DMF, delivering the target Scaffold #1 analogues in 40–71% yield. For analogues **S35-1023** and **S35-1024**, intermediate **4** was coupled with 1,4-diaminobutane or 1,6-hexamethylenediamine under the same general amide-coupling conditions. In these cases, both amino termini of the diamine reacted with two equivalents of acid **4**, furnishing the corresponding symmetric dimeric products.

The synthesis of 24 Scaffold #2 analogues is summarized in Supplementary Fig. S5C. The detailed synthesis and structural characterizations are specified in Supplementary Methods and Supplementary Data 2. Commercially available methyl 1H-pyrrole-2-carboxylate (**5a**) was first subjected to Friedel-Crafts acylation with acetyl chloride in the presence of AlCl_3_ in anhydrous CH_2_Cl_2_, affording key intermediate methyl 4-acetyl-1H-pyrrole-2-carboxylate (**6a**). Subsequent hydrolysis of the methyl ester under basic conditions using aqueous LiOH yielded the corresponding carboxylic acid **7a**. Amide coupling of acid **7a** with 4-methylbenzylamine was then performed using HATU as the coupling reagent and DIPEA as the base in DMF, delivering the target Scaffold #2 core structure. The analogous intermediates **6b**–**6h** and **7b**–**7h** were prepared following the same general procedures, and were subsequently coupled with various amines to furnish the full series of Scaffold #2 derivatives. For analogue **S35-1030**, the target compound was obtained via a single-step routine amide condensation, as outlined in Supplementary Fig. S5D.

The compounds’ ability to inhibit the NADP^+^-dependent oxidation of (S)-(+)-1,2,3,4-tetrahydro-1-naphthol catalyzed by recombinant AKR1C3 was assessed, yielding IC_50_ values (Tables 1 and 2). Given that this study focuses on streamlining ML-integrated virtual screening and structural assessment for hit identification, a comprehensive medicinal chemistry structure–function analysis is beyond the scope of the current manuscript due to space limitations. **S34-1035** and **S34-1040** demonstrate the highest potency on inhibiting AKR1C3 with IC_50_ values of 0.04 ± 0.01 and 0.03 ± 0.01 µM, respectively (Table 2), and were prioritized for subsequent evaluation. To assess isoform selectivity within the closely related AKR1C family, we evaluated the inhibitory activity of **S34-1035** and **S34-1040** against AKR1C1, AKR1C2, and AKR1C4. Both compounds retained nanomolar potency toward AKR1C3 while exhibiting no detectable inhibition of the other AKR1C isoforms (IC_50_ > 100 μM), corresponding to selectivity ratios exceeding 2500-fold (Table 3). In comparison, the parent compound **S07-2001** displayed substantially weaker potency and selectivity (AKR1C3 IC_50_ = 2.08 ± 1.26 μM; selectivity > 48-fold).

**Table 3:** AKR1C isoform inhibitory activity and selectivity of S34-1035, S34-1040, and S07-2001

Compounds	inhibitory activity, IC_50_ (μM)^a^				IC_50_ ratio		
	1C1	1C2	1C3	1C4	1C1:1C3	1C2:1C3	1C4:1C3
S34-1035	>100	>100	0.04 ± 0.01	>100	>2500	>2500	>2500
S34-1040	>100	>100	0.03 ± 0.01	>100	>2500	>2500	>2500
S07-2001	>100	>100	2.08 ± 1.26	>100	>48.1	>48.1	>48.1

^a^The data are expressed as the mean ± SEM of three independent experiments.

**Fig. 5:**
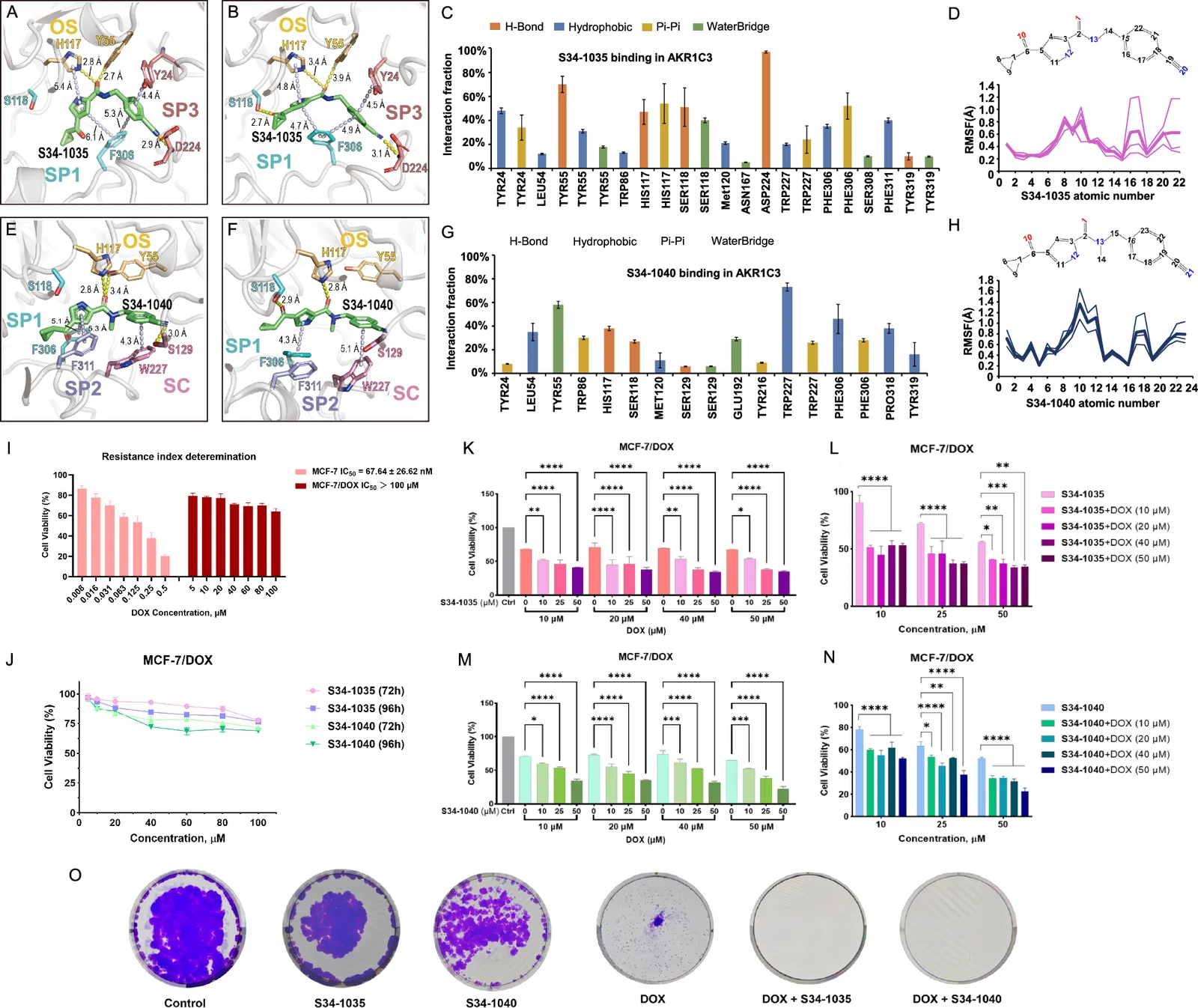
Experimental validation of potent AKR1C3 inhibitors and their chemosensitizing effects. Predicted binding poses of **S34-1035** within the AKR1C3 active site derived from molecular docking (**A**) and representative snapshots from molecular dynamics simulations (**B**). Key interacting residues are labeled. **C** Interaction fraction analysis for **S34-1035** during 100-ns molecular dynamics simulations, showing the frequency of hydrogen-bond, hydrophobic, pi–pi, and water-bridge interactions with AKR1C3 residues. Data are presented as mean ± SD from three independent MD simulation replicates. **D** Root-mean-square fluctuation (RMSF) of **S34-1035** ligand atoms during molecular dynamics simulations. Predicted binding poses of **S34-1040** within the AKR1C3 active site from docking (**E**) and molecular dynamics simulations (**F**). **G** Interaction fraction analysis for **S34-1040** during molecular dynamics simulations. Data are presented as mean ± SD from three independent MD simulation replicates. **H** RMSF profile of **S34-1040** ligand atoms over the simulation trajectory. **I** Evaluation of the resistance index of doxorubicin against MCF-7/DOX cells. **J**. Antiproliferative effects of **S34-1035** and **S34-1040** against MCF-7/DOX cells. **K**, **L** Dose-dependent effects of combined **S34-1035** and DOX treatment on MCF-7/DOX cell viability. **M**, **N** Dose-dependent effects of combined **S34-1040** and DOX treatment on MCF-7/DOX cell viability. **O** Representative clonogenic assay images showing long-term colony formation of MCF-7/DOX cells treated with vehicle control, **S34-1035** (10 μM) or **S34-1040** (10 μM) alone, DOX (25 μM) alone, or DOX (25 μM) in combination with **S34-1035** (10 μM) or **S34-1040** (10 μM). In **K**–**N**, comparison between two groups was performed using an unpaired, two-tailed Student’s *t* test. For **K**: 10 μM **P = 0.0045; 40 μM **P = 0.0064; 50 μM *P = 0.0158; ****P < 0.0001. For **L**: 50 μM *P = 0.0475, **P = 0.0054, ***P = 0.0007, **P = 0.0012; ****P < 0.0001. For **M**: 10 μM *P = 0.0181; 40 μM ***P = 0.0006; 50 μM ***P = 0.0004; ****P < 0.0001. For **N**: 25 μM *P = 0.0158, **P = 0.0076; ****P < 0.0001.

To elucidate the molecular basis underlying AKR1C3 inhibition, molecular modeling and simulations were performed using the previously reported crystal structure of AKR1C3 (**PDB ID: 7X3L**). Static molecular docking suggested that **S34-1035** adopts a well-defined binding pose within the active site, in which the nitrile group forms a polar contact with D224, while the carbonyl moiety engages in H-bond interactions with Y55 and H117. In addition, the phenyl and pyrrole rings establish stabilizing pi-stacking and hydrophobic interactions with Y24, H117, and F306, collectively anchoring the compound within the substrate-binding pocket (Fig. 5A). To assess the stability of these interactions under dynamic conditions, three independent 100-ns molecular dynamics (MD) simulations were conducted for each complex (Supplementary Fig. S6 and Supplementary Data 3–5). Interaction fraction analysis demonstrated that the key H-bond and hydrophobic contacts identified by docking were largely preserved throughout the simulation trajectory, indicating a stable binding mode for **S34-1035** (Fig. 5B, C). Notably, the MD simulations further revealed a conformational adaptability of the carbonyl group linked to the cyclopropane ring, which intermittently rotated to form an additional H-bond with S118. This interaction was accompanied by localized positional fluctuations, as reflected by elevated RMSF values for the corresponding ligand atoms, suggesting a degree of dynamic flexibility within the binding pocket (Fig. 5D). To further characterize ligand conformational stability, clustering analysis of MD trajectories based on ligand heavy-atom conformations was performed. The results revealed that a small number of dominant binding poses were consistently populated across independent simulations, with representative cluster centroids closely matching the docking-derived pose, indicating convergent and stable ligand binding (Supplementary Fig. S6). A comparable interaction pattern was observed for **S34-1040**. Docking and MD simulations revealed conserved polar interactions with Y55 and H117, along with hydrophobic contacts involving W227 and F306, which together stabilize the ligand within the AKR1C3 active site (Fig. 5E–G). Similar to **S34-1035**, the cyclopropane-associated carbonyl group of **S34-1040** exhibited rotational flexibility during MD simulations, enabling transient hydrogen-bond formation with S118 and resulting in modest ligand positional fluctuations (Fig. 5H). These results indicate that both inhibitors engage AKR1C3 through a conserved binding mode characterized by stable core interactions and localized conformational adaptability, providing a structural rationale for their high inhibitory potency.

AKR1C3 contributes to doxorubicin^32,33^ resistance by attenuating drug-induced oxidative stress and detoxifying reactive lipid peroxidation products, thereby dampening DNA damage signaling^25^. Pharmacological inhibition of AKR1C3 disrupts this protective metabolic barrier, leading to the restoration of ROS accumulation and DNA double-strand breaks upon doxorubicin treatment^25^. The doxorubicin-resistant cell line MCF-7/DOX was established in this study using a concentration-gradient method. Resistance assessment showed that the IC_50_ of doxorubicin against wild-type MCF-7 cells was 67.64 ± 26.64 nM, while the IC_50_ against MCF-7/DOX cells exceeded 100 µM, yielding a resistance index >1478.42 (Fig. 5I). We then evaluated the antiproliferative effects of two AKR1C3 inhibitors, **S34-1035** and **S34-1040**, as single agents on MCF-7/DOX cells (Fig. 5J). MTT assays (72 and 96 h) demonstrated that both compounds only showed weak antiproliferative activity even at concentrations up to 100 µM, with good cellular safety, indicating that AKR1C3 inhibitors alone cannot directly inhibit the growth of these tumor cells.

Next, we evaluated whether pharmacological inhibition of AKR1C3 could restore the sensitivity of resistant cells to doxorubicin. MCF-7/DOX cells were treated with **S34-1035** or **S34-1040** in combination with increasing concentrations of doxorubicin. In both cases, the combination treatments produced significantly stronger antiproliferative effects than doxorubicin alone across the tested concentration range (Fig. 5K–N). To further quantify the synergistic effect, we calculated the combination index (CI) using the Chou–Talalay method. The CI values for the combinations of **S34-1035** or **S34-1040** with doxorubicin ranged from 0.145 to 0.637, demonstrating strong synergy between the agents (Table 4). To control for the potential cytotoxicity of the inhibitors themselves, parallel experiments were conducted using matched concentrations of **S34-1035** or **S34-1040** as single agents. Neither compound alone produced a measurable inhibitory effect on MCF-7/DOX cell viability (Fig. 5K–N), and the growth suppression observed in the combination groups was significantly greater than that observed for either inhibitor alone. These results indicate that the enhanced antiproliferative activity arises from a true chemosensitizing effect rather than nonspecific cytotoxicity of the AKR1C3 inhibitors.

**Table 4:** Evaluation of the combined treatment with doxorubicin and compounds S34-1035 and S34-1040

S34-1035 (μM)	DOX (μM)	CI	S34-1040 (μM)	DOX (μM)	CI
10	10	0.183	10	10	0.314
	25	0.145		25	0.237
	40	0.190		40	0.356
	50	0.191		50	0.351
25	10	0.377	25	10	0.562
	25	0.376		25	0.344
	40	0.282		40	0.532
	50	0.281		50	0.233
50	10	0.637	50	10	0.368
	25	0.561		25	0.391
	40	0.498		40	0.325
	50	0.571		50	0.176

CI values were calculated using CompuSyn software at fixed concentrations. Synergy strength was classified by CI value: CI < 0.1, very strong; CI = 0.1–0.3, strong; CI = 0.3–0.7, synergy; CI = 0.7–0.85, moderate; and CI = 0.85–1.0, slight.

Finally, a colony formation assay was performed to evaluate the long-term impact of the combination strategy on cell proliferative capacity (Fig. 5O). The results showed that the combination of either AKR1C3 inhibitor with doxorubicin significantly suppressed the colony-forming ability of MCF-7/DOX cells. The inhibitor-alone groups showed no significant difference compared to the blank control, whereas doxorubicin alone at 10 µM failed to completely inhibit colony formation after 14 days of treatment, further highlighting the enhanced efficacy of the combination regimen.

### An online TAME-VS server enables end-to-end, user-friendly virtual screening workflows

**Fig. 6:**
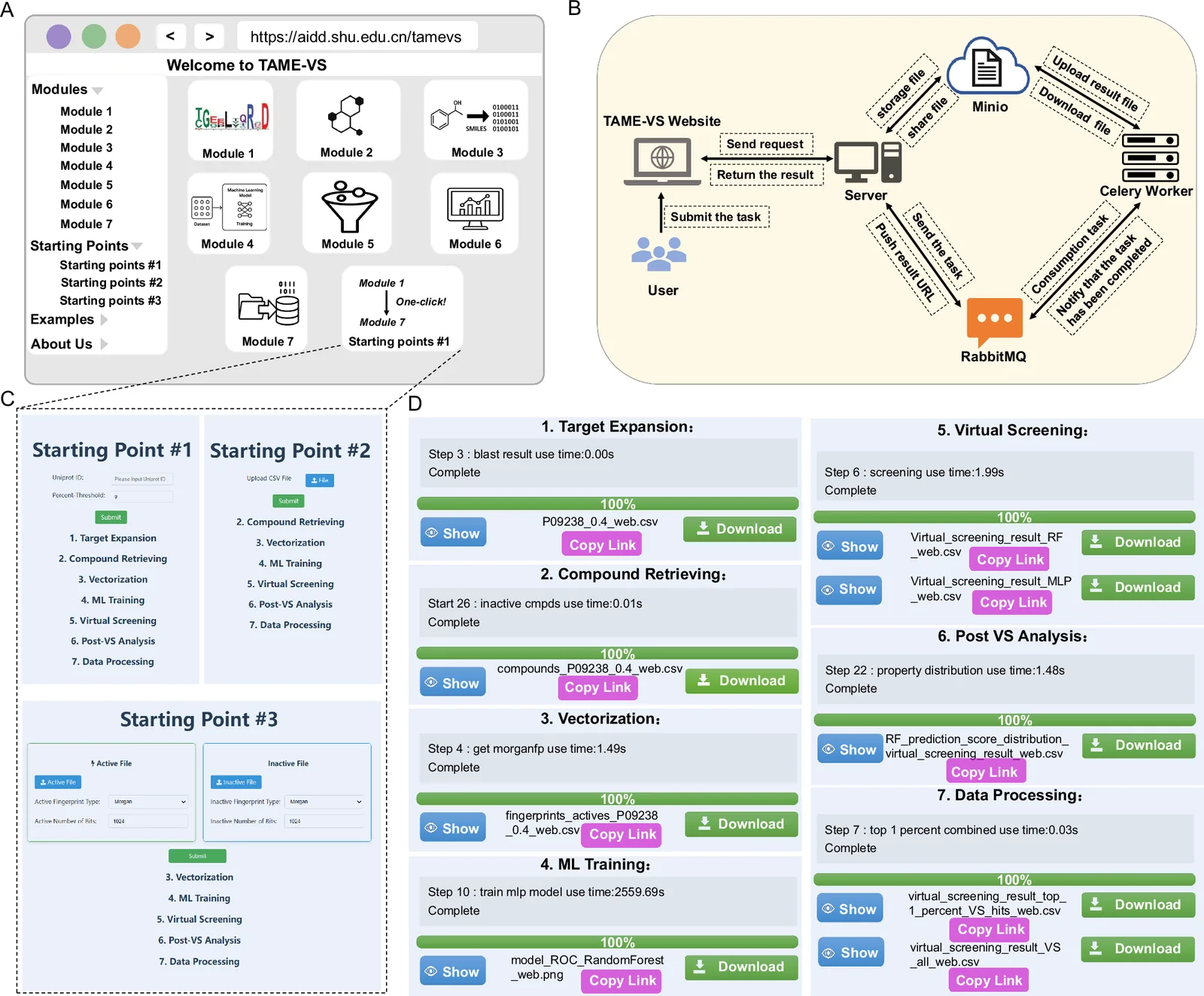
An online server of TAME-VS with a graphical user interface. **A** Overview of the TAME-VS web interface, showing the modular organization of the platform and available workflow components. **B** System architecture of the TAME-VS online server, illustrating the asynchronous execution framework. **C** Three alternative starting points supported by the web server: a target-driven workflow initiated from a UniProt accession number (starting point #1), a target-centric workflow using a user-supplied expanded target list for compound retrieval (starting point #2), and a compound-driven workflow allowing direct upload of curated active and inactive compound sets for customized model training (starting point #3). **D** Step-by-step execution view of the TAME-VS workflow within the GUI, showing real-time progress tracking, execution status of each module, and options for inspecting intermediate results. For each completed step, users can click the show button to preview the result file directly within the browser, click the download button to save the result file locally, or click the copy Link button to copy a temporary download URL (valid for 7 days) that can be used to retrieve result files from previously submitted tasks.

To facilitate accessibility and practical adoption of the TAME-VS framework, we developed an online server equipped with a graphic user interface (GUI) that integrates computational modules into a unified workflow (Fig. 6). The web server is publicly accessible at https://aidd.shu.edu.cn/tamevs and is designed to allow users to perform target expansion, data retrieval, model training, virtual screening, and post-screening analysis without requiring local software installation or command-line operations. This implementation extends our earlier terminal-based GitHub release to a fully web-accessible platform.

The GUI is organized into seven modular components corresponding to the core steps of the TAME-VS pipeline (Fig. 6A). Users can initiate analyses from three distinct starting points depending on data availability: (i) a target-driven workflow beginning with a UniProt accession number, enabling automated target expansion and compound retrieval; (ii) a target-centric workflow starting from user-supplied expanded target list for direct compound retrieval; or (iii) a compound-driven workflow allowing users to upload curated active and inactive compound sets for customized model training. This flexible design accommodates use cases ranging from early-stage target exploration to focused lead optimization. The execution status and intermediate outputs of each module are displayed in real time within the interface (Fig. 6B, C). The online server preserves consistency with the methodological framework described in this study. Default settings reproduce the strategy #2 configuration used in prospective validation, while advanced users retain the option to adjust key parameters, such as fingerprint types, activity cutoffs, and classification thresholds. In this way, the TAME-VS web server serves not only as a dissemination tool but also as a reproducible implementation of the platform evaluated in this work.

From a system architecture perspective, the TAME-VS server adopts a distributed and asynchronous design to ensure robustness and scalability (Fig. 6D). User requests submitted through the web interface are processed by a central server, which dispatches computational tasks to worker nodes via a message queue system (RabbitMQ^34^). Intermediate and final results are stored and managed using an object storage service (MinIO), ensuring an interaction between front-end operations and back-end computation.

## Discussion

A recurring challenge in machine learning for early-stage drug discovery is that successes reflect modifications within familiar chemical neighborhoods rather than generalization to unseen chemotypes. Under screening conditions, the most valuable models are those that capture activity-determining patterns that transfer across scaffold boundaries, rather than those that reproduce similarity search. This perspective complements structure-based AI screening efforts on the same target, where deep learning models trained on protein–ligand complexes have been used to identify new AKR1C3 inhibitors from large virtual libraries^24^. In those settings, model success is typically evaluated through the identification of active compounds from a predefined screening space. By contrast, the present work emphasizes whether learning-based models can recognize activity-determining patterns that transfer across chemically and temporally distinct compound sets, providing a more stringent test of scaffold-level generalization. A feature of our retrospective validation strategy is therefore the use of inhibitor series published after the bioactivity snapshot used during training, which provides a stringent test of novelty generalization. The ability of TAME-VS models to recognize preferred chemotypes in this setting suggests that supervised classifiers trained on heterogeneous historical data can learn functional motifs relevant to target engagement, which are patterns that may be distributed across substructures and not reducible to a single nearest-neighbor relationship. This view is consistent with the observation that active and inactive compounds overlap substantially in simple physicochemical property space and are not trivially separable by similarity metrics alone. Still, they can be differentiated by models trained on curated activity labels.

The observed improvements from homolog-augmented training highlight a second principle with broad relevance: that the bottleneck in drug discovery is often not the lack of algorithms but the lack of usable, well-labeled data. Although molecular fingerprints are widely used as descriptors in predictive modeling due to their interpretability and accessibility, their utility remains contingent on the availability of well-curated activity labels and appropriate model training strategies^12^. Target families frequently contain highly related proteins for which activity data are unevenly distributed. Restricting models to a single target may yield unstable decision boundaries and exaggerated sensitivity to dataset perculiarity. By incorporating homolog-derived ligands and explicitly exploring activity thresholds, TAME-VS adopts a pragmatic stance that, instead of insisting on assay homogeneity, it uses controlled label definitions to create a learnable contrast between actives and inactives and to tune the operational tradeoff between enrichment and coverage. Activity thresholds should be interpreted as operational definitions that enable model training rather than strict indicators of target exclusivity. While compounds with IC_50_ values in the micromolar range may exhibit off-target interactions, the use of separated active and inactive cutoffs introduces a controlled contrast that supports robust learning. Importantly, downstream selectivity profiling and phenotypic validation are required to establish target-specific biological relevance. This is reflected in the systematic dependence of performance on the active–inactive potency gap, and in the predictable shifts in hit rate as the classification threshold is adjusted. In this context, classification is not merely a modeling choice but a decision-theoretic interface. It enables users to choose between high-confidence enrichment (precision) and broader recovery (recall), depending on project constraints.

Prospective validation provides the most direct evidence of practical value because it forces models to operate under the same constraint that dominates real projects, which are limited synthesis and testing capacity. Importantly, our analysis does not treat model performance as competing with medicinal chemistry; instead, it quantifies how model-based prioritization can further enrich actives within a rationally designed chemical space. The baseline activity rate achieved by expert-guided scaffold selection underscores that domain knowledge remains essential. However, the added enrichment from TAME-VS prioritization demonstrates a complementary function that, given an expert-designed chemical space, the model helps decide which compounds to make first, thereby increasing experimental efficiency. This framing is important because it reflects how machine learning is most likely to contribute in practice that not by replacing expert design, but by improving allocation of experimental resources. The biological validation further elevates the contribution beyond hit finding. AKR1C3 is not merely an enzymatic target but a component of a broader resistance phenotype through its role in detoxifying reactive intermediates and attenuating oxidative stress. Existing AKR1C3 inhibitors span multiple chemotypes with strong enzymatic activity, yet practical limitations related to selectivity, cellular properties, or synthetic tractability remain in several cases (Supplementary Table 9). The compounds identified here, therefore, complement the current landscape by providing additional chemically tractable scaffolds with a balanced activity profile. The demonstration that potent inhibitors restore doxorubicin sensitivity and suppress long-term clonogenic survival establishes that the prioritized compounds are not only biochemically active but also functionally relevant in a clinically meaningful context. While the biochemical potency and isoform selectivity strongly support AKR1C3 engagement, we note that direct cellular target engagement or genetic perturbation studies would be required to establish definitive causality between enzyme inhibition and the observed phenotypic effects. Conceptually, this closes a key loop that is still missing from many AI-enabled screening studies, that computational prioritization must ultimately be judged by whether it changes biological outcomes in the intended direction. By placing enzyme inhibition, structural rationalization, and phenotypic reversal in a single workflow, this study illustrates an integrative template for validating ML-guided hit discovery.

Despite these advances, several opportunities remain for improving both generality and mechanistic fidelity. First, activity labels aggregated from public sources inherently reflect assay heterogeneity, experimental noise, and context-dependent pharmacology. Future versions of TAME-VS could incorporate assay-aware stratification, uncertainty quantification, or hierarchical label models to better reconcile heterogeneous measurements. Second, while homolog pooling improves robustness, it also raises the question of selectivity within closely related families, such as the AKR1C subfamily. Extending TAME-VS toward multi-task learning across isoforms, explicit counter-screen objectives, or joint optimization of potency and selectivity would make the platform more directly actionable for lead optimization. Third, the current classification-centric paradigm is well matched to early-stage enrichment, but later stages may benefit from hybrid approaches that combine classification for prioritization with regression or calibrated probability models for potency estimation and risk-aware decision-making. Fourth, while the prospective results demonstrate clear enrichment through model-guided prioritization, future studies should examine which functional groups or physicochemical features most strongly drive model decisions, and evaluate model behavior in less expert-biased or partially model-generated chemical spaces. Such analyses would provide deeper insight into whether structural novelty or scaffold hopping emerges when design constraints are relaxed, and would more directly assess the extent to which the models capture target-specific structure–activity features beyond reinforcement of expert priors.

The progress in ML-enabled drug discovery will increasingly depend on closing the loop between data, models, and experiments, and on building systems that are not only accurate but also deployable, reproducible, and aligned with how decisions are made in medicinal chemistry. The online TAME-VS server reflects this philosophy by making the validated pipeline executable by a wider community and enabling iterative model updates as new compounds are tested. We anticipate that continued integration of target-centric data acquisition, robust learning under label noise, and experimentally grounded validation will be essential for translating machine learning advances into practical gains in early-stage discovery.

## Methods

### Computational methods

#### Target input and target expansion

AKR1C3 was specified by UniProt accession P42330. Target expansion was performed using a BLAST-based similarity search^35^ implemented in the TAME-VS Target Expansion module. Homologous enzymes were retained if the sequence identity exceeded 40% (as computed by the BLASTp alignment, Biopython 1.83, E_VALUE_THRESH = 0.04, max hits = 500). The expanded target list was used downstream for compound retrieval. For the AKR family analysis in this manuscript, the target list used in the main modeling experiments corresponded to seven AKR family members^36–38^ plus AKR1C3: P42330 (AKR1C3), P17516 (AKR1C4), P52895 (AKR1C2), Q04828 (AKR1C1), O60218 (AKR1B10), P15121 (AKR1B1), and P14550 (AKR1A1).

#### Compound retrieval from ChEMBL and dataset construction

Compound activity data were retrieved by the Compound Retrieval module through automated queries to ChEMBL. The ChEMBL snapshot used in this work was accessed on 12/20/2024 (ChEMBL35). Retrieved records were restricted to target-associated small molecules with standardized activity annotations available in ChEMBL. Three retrieval strategies were implemented: strategy #1 (target-only): only P42330 (AKR1C3) was queried. Strategy #2 (homolog-informed; default in TAME-VS): each of the seven AKR family members listed above was queried individually, and activity records were pooled to construct a broader dataset incorporating homolog-derived information. Strategy #3 (curated homolog-informed): the pooled dataset from strategy #2 was refined through manual curation of high-quality activity records to generate a cleaner training set.

For each target, the most abundant experimental activity type available was used to define active and inactive compounds. Across the retrieved AKR datasets, IC_50_ measurements constituted the largest fraction of records and were therefore prioritized for model training and evaluation in this manuscript (additional assay types were retained only for descriptive analyses unless noted). Because different training-set strategies in the Results section evaluate alternative “potency-gap” definitions, we used the strategy-specific cutoff pairs (Fig. 2D) for the main model comparisons.

#### Handling of repeated measurements and conflicting annotations

When multiple activity entries existed for the same compound–target pair, records were aggregated using median after unit standardization, and compounds with conflicts that simultaneously meeting active and inactive criteria under the same strategy were excluded. Records with ambiguous qualifiers, such as “>” and “<,” were handled according to their numerical values relative to the defined activity thresholds (e.g., 1000 nM). Compounds were standardized prior to featurization using RDKit^39^. The final modeling dataset was de-duplicated by canonical SMILES (Fig. 2C).

#### Molecular featurization and fingerprint generation

Molecular fingerprints were computed in the vectorization module. Morgan fingerprints were used as the default representation with radius = 2 and 1024 bits, consistent with the standard TAME-VS configuration. Fingerprints were generated using RDKit (version: 2024.3.2). In addition to Morgan fingerprints, TAME-VS supports AtomPair, topological, and MACCS fingerprints. Where relevant, alternative fingerprint types were evaluated in parallel under identical cross-validation settings and are reported in Supplementary Tables 3 and 4. To control the family-wise error rate across the six pairwise comparisons within each strategy, the Holm–Bonferroni step-down correction (*α* = 0.05) was applied^27^. Beyond statistical significance, Cohen’s *d* was computed as a sample-size-independent measure of effect size.

#### Machine learning model training and internal validation

RF and MLP classifiers were trained using molecular fingerprints as input features and binary activity labels as outputs. For each strategy-specific dataset, compounds were split into a training set and a held-out test set at an 80:20 ratio. All cross-validation and hyperparameter selection steps were performed using the training split only. Class imbalance was addressed by random undersampling of the majority class within each fold of stratified 10-fold cross-validation to preserve the class ratio structure while preventing majority-class dominance. Python library Scikit-learn^40^ was applied here. Hyperparameters were optimized via grid search using accuracy as the selection criterion, consistent with the current implementation of the ML model training module. RF tuning parameters: n_estimators (50, 100, and 200), max_depth (4, 6, 10, and 12). MLP tuning parameters: hidden_layer_sizes ((50, 50, 50), (50, 50), (50)), activation (relu, tanh), alpha (1e-4, 1e-2). After selection, the best-performing hyperparameter configuration was retrained on the full training split and evaluated on the held-out test set. Model performance was assessed using AUROC as the primary metric, computed with *sklearn.metrics.roc_auc_score*. Additional metrics, including precision, recall, specificity, F1 score, geometric mean (Geo), and IBA, were also computed to characterize model behavior under class imbalance and to support multi-metric comparisons^41,42^.

#### Chemical-space visualization by t-SNE

Chemical-space embeddings were generated using t-distributed stochastic neighbor embedding (t-SNE) applied to 1024-bit Morgan fingerprints^41^. Dimensionality reduction was performed using scikit-learn *sklearn.manifold.TSNE* with default parameters. Training and validation compounds were plotted with distinct markers by origin and activity status, and training points were rendered at 60% opacity to reduce overplotting.

#### Hit-rate curves across classification thresholds and hit definitions

Hit-rate curves were generated to assess enrichment performance as a function of the classification threshold applied to predicted probabilities^1,2,43^. For each model (RF and MLP) under strategy #2 and strategy #3, predicted probabilities for the evaluation set were ranked in descending order. Hit rate was computed as the percentage of true actives among the compounds predicted active at each threshold cutoff. Thresholds were swept from 0.20 to 0.995. In parallel, the number of correctly predicted active compounds at each threshold was recorded and displayed on a secondary axis. To evaluate robustness to different definitions of “hit,” the activity cutoff was varied, for example, IC_50_ < 1000 nM vs. < 100 nM, and hit-rate curves were recalculated under each cutoff. Model performance before and after retraining was compared using identical threshold grids.

#### Molecular docking and molecular dynamics simulations

The molecular docking was performed using the Glide module in Schrödinger as previously described^44^. The binding pocket of AKR1C3 (PDB ID: 7X3L^45^) was defined based on the position of the previously reported ligand **S07044** in the cocrystal structure. The 3D conformations of compounds **S34-1035** and **S34-1040** was prepared using LigPrep. Glide SP, in standard precision mode, was used without constraints to generate binding poses. For each ligand, up to 20 docking poses were generated within the defined binding pocket of AKR1C3. Among these poses, the top 10 poses underwent the post-docking minimization.

Two docking-derived AKR1C3–ligand complex structures, corresponding to **S34-1035** and **S34-1040**, were prepared before modeling and simulations. The module of Protein Preparation in Schrödinger Maestro^46^ was applied to cap termini, repair residues, optimize H-bond assignments, and run restrained minimizations following default settings. The Schrödinger Desmond MD engine was used for simulations as previously described^47–49^. An orthorhombic water box was applied to bury prepared protein systems with a minimum distance of 10 Å to the edges from the protein. Water molecules were described using the SPC model. Na+ ions were placed to neutralize the total net charge. All simulations were performed following the OPLS4^50^ force field. The ensemble class of NPT was selected with the simulation temperature set to 300 K (Nose-Hoover chain) and the pressure set to 1.01325 bar (Martyna-Tobias-Klein). A set of default minimization steps pre-defined in the Desmond protocol was adopted to relax the MD system. Each MD simulation was performed for 100 ns with 3 replicates for the two protein–ligand systems, with one frame saved every 100 ps during the sampling phase. Hierarchical clustering was performed on three independent MD trajectories using TTClust 4.10.4^51^, a Python-based trajectory clustering program built on the MDTraj library.

### Experimental methods

#### General chemistry

Unless indicated otherwise, all chemicals used were of analytical grade and did not require further purification. The reactions were tracked using analytical TLC on silica gel GF254 plates, viewed under UV light at 254 and 365 nm wavelengths. The compounds described were purified by column chromatography using silica gel (300–400 mesh) from Qingdao Haiyang Chemical Co., Ltd. (China) and GF254 plates with 8‰ Sodium carboxymethyl cellulose (600–1000 mpa s) from Shanghai Macklin Biochemical Co., Ltd.(China). Purity confirmation was carried out using high-performance liquid chromatography (HPLC) on a Waters 2695 system with an Agilent Technologies 1260 infinity C_18_ reversed-phase column (4.60 mm × 150 mm × 5 μm), employing either methanol/water or acetonitrile/water solvent system with or without 1‰ triethylamine and a flow rate of 1.0 mL/min. All compounds were confirmed to have >95% purity by HPLC analysis. Melting points were measured on a YRT-3 apparatus (China). HR-MS data were collected using an Agilent LC-MS 6120 instrument with an ESI detector, operating in both positive and negative ion modes. ^1^H and ^13^C NMR spectra were recorded at 300/400/500 MHz and 75/101/126 MHz, respectively, on a Varian INOVA spectrometer using TMS as an internal standard in chloroform-*d* or DMSO-*d*_*6*_ at 300 K. Chemical shift values (δ) are given in parts per million (ppm), and coupling constants (J) in hertz (Hz). Data are presented as follows: chemical shift, multiplicity (s: singlet, d: doublet, t: triplet, q: quartet, m: multiplet), coupling constants, and integration. Detailed synthetic procedures and analytical data for each individual derivative are provided in the Supplementary Methods.

#### Expression and purification of AKR1Cs

His-tagged AKR1Cs constructs were cloned into the pET 30a vector and expressed in E. coli BL21(DE3) cells using 0.5 mM IPTG induction at 16 °C. The bacterial pellets were resuspended in Bacterial Active Protein Extraction Reagent, which is purchased from Beyotime Biotechnology (Shanghai, China). After centrifugation, the supernatant was subjected to nickel affinity chromatography. The eluted proteins were analyzed by SDS PAGE, concentrated via ultrafiltration, quantified using the BCA assay, and stored in Tris-HCl buffer (40 mM Tris-HCl, 200 mM NaCl, pH 8.0) with 20% glycerol at −80 °C.

#### Enzymatic inhibition assays against recombinant AKR1Cs

The inhibitory activities against AKR1Cs were assessed by monitoring the NADP+-dependent oxidation of (S)-(+)-1,2,3,4-tetrahydro-1-naphthol using a fluorescence-based assay. (S)-(+)-1,2,3,4-tetrahydro-1-naphthol was purchased from Daicel (Shanghai, China). Briefly, a stock solution of (S)-(+)-1,2,3,4-tetrahydro-1-naphthol and inhibitors was prepared in DMSO, followed by dilution with Tris-HCl buffer (40 mM Tris-HCl, 200 mM NaCl, pH 8.0). For the enzyme assay, reactions were assembled in a 96-well plate. Each well received 20 μL aliquots of the following components: (S)-(+)-1,2,3,4-tetrahydro-1-naphthol substrate, 200 μM NADP+, inhibitor at specified concentrations, and the purified recombinant enzyme, the latter being diluted in Tris-HCl buffer mentioned before. The final volume was 200 μL with a DMSO concentration of 2% (v/v). After the addition of enzymes to start the reaction, the plate was incubated at 37°C for 10 min. (S)-(+)-1,2,3,4-tetrahydro-1-naphthol concentrations for each AKR1C isoform matched their respective Km values (5.0 μM for AKR1C1, 54.8 μM for AKR1C2, 200.0 μM for AKR1C3, and 61.8 μM for AKR1C4). Then, fluorescence was measured at Ex340 nm/Em460 nm. IC_50_ values were determined by nonlinear regression analysis using GraphPad Prism 8.0 (GraphPad Software, Inc., CA, USA).

#### Cell cultures and reagents

The doxorubicin-resistant MCF-7 cell line (MCF-7/DOX) was generated in our laboratory. Dulbecco’s modified Eagle’s medium (DMEM), penicillin, and streptomycin solutions were purchased from KeyGen biotech (Nanjing, China). Fetal bovine serum (FBS) was purchased from Gibco (Invitrogen Corp., USA). 3-(4,5-dimethylthiazol)-2, 5-diphenyltetrazolium bromide (MTT) was purchased from Solarbio (Beijing, China). Doxorubicin hydrochloride was purchased from Sigma-Aldrich (Shanghai, China). 4% (w/v) paraformaldehyde and 0.1% crystal violet solution were purchased from Beyotime Biotechnology (Shanghai, China).

#### Development and maintenance of the doxorubicin-resistant MCF-7/DOX cell line

The doxorubicin-resistant MCF‑7/DOX cell line was established in‑house by continuous exposure of parental MCF‑7 cells to stepwise increasing concentrations of doxorubicin. Briefly, cells in the logarithmic growth phase were treated with doxorubicin starting from a low concentration, followed by recovery in drug‑free medium until they reached the logarithmic phase again. This cycle was repeated over approximately 9 months until a stable resistant population capable of proliferating under the target drug concentration was obtained. To maintain the resistant phenotype, MCF‑7/DOX cells were routinely cultured in medium supplemented with 2 μM doxorubicin. For all subsequent experiments, cells were passaged at least twice in doxorubicin‑free medium prior to use.

#### Cell viability assays in the doxorubicin-resistant MCF-7/DOX cell line

Cell viability was assessed by MTT assay using doxorubicin-resistant MCF-7/DOX cells cultured in DMEM with 10% FBS at 37 °C under 5% CO_2_. For single-agent testing, cells (6000/well) were treated with compound gradients for 72–96 h before MTT incubation (4 h) and DMSO solubilization. Viability was calculated as (treated OD_570_/control OD_570_) × 100%, and dose-response curves were fitted with GraphPad Prism 8.0. All source data are provided in Supplementary Data 6.

#### Drug combination assays

For combination studies, cells (4000/well) were pretreated with AKR1C3 inhibitor for 24 h, then co-treated with inhibitor and doxorubicin for 72 h. Viability was similarly measured, and synergy was analyzed using CompuSyn software: CI < 1 indicated synergy (CI < 0.1, very strong; 0.1–0.3, strong; 0.3–0.7, synergy; 0.7–0.85, moderate; 0.85–1.0, slight), while CI ≥ 1 indicated additivity or antagonism. All source data are provided in Supplementary Data 6.

#### Clonogenic assays

MCF‑7/DOX cells in the logarithmic growth phase were collected and seeded into 6‑well plates at a density of 1000 cells per well. After 24 h of incubation at 37 °C to allow cell attachment, the culture medium was replaced with drug‑containing medium, which was refreshed every 3 days. Following 14 days of culture, visible colonies formed. The medium was then discarded, and the cells were washed, fixed with 4% (w/v) paraformaldehyde for 30 min, and stained with 0.1% crystal violet solution for 20 min. After rinsing off residual stain with PBS, the colonies were photographed.

#### Statistical analysis

Statistical analysis was performed using GraphPad Prism 8.0. Unless noted otherwise, results are reported as the mean ± standard error of the mean (SEM) based on three independent experiments conducted in parallel. Group comparisons between treatment and control conditions were carried out using an unpaired two-tailed Student’s *t* test, with statistical significance set at *P* < 0.05. Differences among three or more groups were assessed using one-way ANOVA. *P* < 0.01 denoted statistical significance.

### Online server implementation

A publicly accessible online implementation of TAME-VS is available at https://aidd.shu.edu.cn/tamevs. The server was designed to enable end-to-end execution of the TAME-VS workflow without local installation or command-line usage, extending the earlier terminal-based GitHub release to a web-accessible platform.

#### System architecture

The TAME-VS web server is implemented with a Python-based backend built on FastAPI^52^ (FastAPI 0.115.14, Python 3.8.0), which exposes REST APIs for workflow configuration, task submission, and result retrieval, and coordinates communication among the execution components. To support computationally intensive screening and model training while maintaining responsive user interaction, the platform adopts an asynchronous task execution architecture using RabbitMQ^34^ (4.1.2) as the message broker and Celery (5.5.3) as the distributed task-queue framework. Upon task initiation from the web interface, the frontend first requests a temporary upload endpoint from the backend. The backend generates a unique file ID and returns a pre-signed upload URL to MinIO (7.2.10), enabling direct client-side upload of input files to the object storage service without routing large payloads through the application server. After the upload completes, the user submits task parameters together with the file ID, and the backend enqueues the job to RabbitMQ. Celery workers consume queued tasks and execute them asynchronously. During execution, intermediate artifacts and final outputs are written to MinIO, which serves as the centralized storage layer for large inputs/outputs and supports scalable file management. Upon completion, the worker uploads the result files to MinIO and signals task completion back to the backend through RabbitMQ. The backend then delivers real-time status updates and result links to the client via a WebSocket^53^ (13.1) connection, enabling progress visualization and immediate access to downloadable outputs. The TAME-VS online server was deployed on a Dell Precision 5860 workstation equipped with an Intel Xeon W7-2495X processor (24 cores, 48 threads, 45 MB cache, base power 225 W, maximum turbo frequency up to 4.8 GHz). The system was configured with 256 GB DDR5 ECC memory (4 × 64 GB RDIMM, 4800 MHz). Storage included a 2 TB M.2 PCIe NVMe solid-state drive for the operating system and active workloads, complemented by two 8 TB 3.5-inch SATA hard disk drives (7200 RPM) for data archiving. GPU acceleration was provided by an NVIDIA RTX A6000 graphics card with 48 GB GDDR6 memory. The server operated under Ubuntu Linux 22.04 LTS.

### Reporting summary

Further information on research design is available in the Nature Portfolio Reporting Summary linked to this article.

## Supplementary information

Transparent Peer Review File

Supplementary Information

Description of Additional Supplementary Files

Supplementary_Data_1

Supplementary_Data_2

Supplementary_Data_3

Supplementary_Data_4

Supplementary_Data_5

Supplementary_Data_6

Reporting Summary

## Data Availability

The newly developed TAME-VS online portal with a graphical user interface is available at https://aidd.shu.edu.cn/tamevs. The synthetic methods, chemical identities, purities (LC/MS), and spectroscopic analysis (^1^H and ^13^C NMR) for all compounds in this study are provided in the Supplementary Information and Supplementary Data files.

## Code availability

The previously published terminal version of the TAME-VS^19^ is accessible at https://github.com/bymgood/Target-driven-ML-enabled-VS and has been archived on Zenodo with https://doi.org/10.5281/zenodo.20422709.
