# Supplementary material for: Target-driven machine learning–enabled virtual screening (TAME-VS) enables prioritization of AKR1C3 inhibitors
Source: Commun Chem. 2026 Jun 15;9:311. doi: 10.1038/s42004-026-02092-6 (PMC13631243; doi:10.1038/s42004-026-02092-6)
Supplement: Supplementary file 1 [file 42004_2026_2092_MOESM10_ESM.pdf]

nature portfolio

Corresponding author(s): Yuemin Bian, Haopeng Sun, Xin Gao

Last updated by author(s): Apr 29, 2026

Reporting Summary

Nature Portfolio wishes to improve the reproducibility of the work that we publish. This form provides structure for consistency and transparency in reporting. For further information on Nature Portfolio policies, see our Editorial Policies and the Editorial Policy Checklist.

Statistics

For all statistical analyses, confirm that the following items are present in the figure legend, table legend, main text, or Methods section.

n/a	Confirmed	
☐	☒	The exact sample size (*n*) for each experimental group/condition, given as a discrete number and unit of measurement
☐	☒	A statement on whether measurements were taken from distinct samples or whether the same sample was measured repeatedly
☐	☒	The statistical test(s) used AND whether they are one- or two-sided<br>*Only common tests should be described solely by name; describe more complex techniques in the Methods section.*
☒	☐	A description of all covariates tested
☒	☐	A description of any assumptions or corrections, such as tests of normality and adjustment for multiple comparisons
☐	☒	A full description of the statistical parameters including central tendency (e.g. means) or other basic estimates (e.g. regression coefficient) AND variation (e.g. standard deviation) or associated estimates of uncertainty (e.g. confidence intervals)
☒	☐	For null hypothesis testing, the test statistic (e.g. *F*, *t*, *r*) with confidence intervals, effect sizes, degrees of freedom and *P* value noted<br>*Give P values as exact values whenever suitable.*
☒	☐	For Bayesian analysis, information on the choice of priors and Markov chain Monte Carlo settings
☒	☐	For hierarchical and complex designs, identification of the appropriate level for tests and full reporting of outcomes
☒	☐	Estimates of effect sizes (e.g. Cohen's *d*, Pearson's *r*), indicating how they were calculated

Our web collection on statistics for biologists contains articles on many of the points above.

Software and code

Policy information about availability of computer code

Data collection	1. Compounds data: ChEMBL (https://www.ebi.ac.uk/chembl/),chembl_webresource_client 0.10.8, Python 3.8.0<br>2. AKR1C3 PDB ID: Protein Data Bank (https://www.rcsb.org/)<br>3. Enzymatic Inhibition Assay: Reactions were assembled in a 96-well plate and fluorescence was measured at Ex340 nm / Em460 nm.<br>4. Cell Viability Assay: Cell viability was assessed by MTT reagent was sourced from Sigma-Aldrich (Shanghai, China) using doxorubicin-resistant MCF-7/DOX cells cultured in DMEM with 10% FBS at 37 °C under 5% CO_2.<br>5. Drug Combination Assay: Cells (4000/well) were pretreated with AKR1C3 inhibitor for 24 h, then co-treated with inhibitor and doxorubicin for 72 h.
Data analysis	1. Scientific Computing Libraries: pandas 1.3.5, rdkit 2024.3.2, scikit-learn 1.0.2, scipy 1.7.3, Python 3.8.0<br>2. Data Visualization: Microsoft 365, GraphPad Prism 8.0, seaborn 0.11.2, matplotlib 3.5.3<br>3. Molecular docking and Molecular Dynamics Simulations: Schrödinger Maestro 14.2, PyMOL 3.0.0, ChemDraw 22.0.0<br>4. Drug Synergy Analysis: CompuSyn 3.0.1 software

For manuscripts utilizing custom algorithms or software that are central to the research but not yet described in published literature, software must be made available to editors and reviewers. We strongly encourage code deposition in a community repository (e.g. GitHub). See the Nature Portfolio guidelines for submitting code & software for further information.

Data

Policy information about availability of data

All manuscripts must include a data availability statement. This statement should provide the following information, where applicable:

- Accession codes, unique identifiers, or web links for publicly available datasets
- A description of any restrictions on data availability
- For clinical datasets or third party data, please ensure that the statement adheres to our policy

1. Compounds data of training set can be found through ChEMBL (https://www.ebiac.uk/chemb/).
2. AKR1C3 PDB ID can be found through Protein Data Bank (https://www.rcsb.org/).
3. Compounds data of retrospective validation can be found from two papers published in JMC 2025(https://doi.org/10.1021/acs.jmedchem.4c03035) and EJMC 2025(https://doi.org/10.1016/j.ejmech.2025.117413).
4. Synthetic methods, chemical identities, purities (LC/MS), and spectroscopic analysis (1H and 13C NMR) for 41 novel compounds are provided in Supplementary Information.

Research involving human participants, their data, or biological material

Policy information about studies with human participants or human data. See also policy information about sex, gender (identity/presentation), and sexual orientation and race, ethnicity and racism.

Reporting on sex and gender	N/A
Reporting on race, ethnicity, or other socially relevant groupings	N/A
Population characteristics	N/A
Recruitment	N/A
Ethics oversight	N/A

Note that full information on the approval of the study protocol must also be provided in the manuscript.

Field-specific reporting

Please select the one below that is the best fit for your research. If you are not sure, read the appropriate sections before making your selection.

☒ Life sciences ☐ Behavioural & social sciences ☐ Ecological, evolutionary & environmental sciences

For a reference copy of the document with all sections, see nature.com/documents/nr-reporting-summary-flat.pdf

Life sciences study design

All studies must disclose on these points even when the disclosure is negative.

Sample size	1. In Model Training: For Strategy #1, there were 266 active (IC50 < 1000 nM)compounds and 120 inactive (IC50 > 5000 nM) compounds; for Strategy #2, there were 1066 active (IC50 < 1000 nM) compounds and 315 inactive (IC50 > 10000 nM)compounds; and for Strategy #3, there were 412 active compounds (labelled) and 315 inactive (IC50 > 10000 nM) compounds.<br>2. Enzymatic Inhibition Assay: At least two independent experiments were performed.
Data exclusions	Duplicate compounds from ChemBL database were removed to ensure uniqueness based on the molecule chembl id.
Replication	To verify the reproducibility of our experimental resuts, we conducted 10-cross validation method for the training of RF/MLP model classifier.
Randomization	No randomization was performed.
Blinding	Blinding was not relevantto our study, since the goal of this study is to develop a computational pipeline to effectively design and discover AKR1C3 inhibitor using machine learning methods.

Reporting for specific materials, systems and methods

We require information from authors about some types of materials, experimental systems and methods used in many studies. Here, indicate whether each material, system or method listed is relevant to your study. If you are not sure if a list item applies to your research, read the appropriate section before selecting a response.

Materials & experimental systems

n/a	Involved in the study
☒	☐ Antibodies
☐	☒ Eukaryotic cell lines
☒	☐ Palaeontology and archaeology
☒	☐ Animals and other organisms
☒	☐ Clinical data
☒	☐ Dual use research of concern
☒	☐ Plants

Methods

n/a	Involved in the study
☒	☐ ChIP-seq
☒	☐ Flow cytometry
☒	☐ MRI-based neuroimaging

Eukaryotic cell lines

Policy information about cell lines and Sex and Gender in Research

Cell line source(s)	1. MCF-7 cell line was purchased from the Cell Culture Center at the Institution of Basic Medical Sciences,Chinese Academy of Medical Science(Shanghai,China).<br>2. MCF-7/ADR was established by ourseleves.<br>3. E.coli BL21(DE3) cells obtained from DeTABIO (Nanjing,China).
Authentication	None of the used cell lines were authenticated by us.<br>The MCF-7/ADR cell line was generated in our lab by continuous exposure of MCF-7 cell line to increasing concertration of 2μM DOX over 9 months. Successful construction of MCF-7/ADR was vertified by comparision of IC50.
Mycoplasma contamination	All cell lines tested negative for mycoplasma contamination.
Commonly misidentified lines (See ICLAC register)	No commonly misidentified cell lines were used in this study.

Plants

Seed stocks	N/A
Novel plant genotypes	N/A
Authentication	N/A
