# Supplementary material for: Target-driven machine learning–enabled virtual screening (TAME-VS) enables prioritization of AKR1C3 inhibitors
Source: Commun Chem. 2026 Jun 15;9:311. doi: 10.1038/s42004-026-02092-6 (PMC13631243; doi:10.1038/s42004-026-02092-6)
Supplement: Supplementary file 2 [file 42004_2026_2092_MOESM1_ESM.pdf]

nature portfolio

Peer Review File

Target-driven machine learning–enabled virtual screening (TAME-VS) enables prioritization of AKR1C3 inhibitors

Corresponding Author: Professor Yuemin Bian

This file contains all reviewer reports in order by version, followed by all author rebuttals in order by version.

Attachments originally included by the reviewers as part of their assessment can be found at the end of this file.

Version 0:

Reviewer comments:

Reviewer #1

(Remarks to the Author)
Nature journals explicitly discourage priority, or novelty claims that cannot be robustly verified. The framing of the manuscript, beginning with the title and reinforced throughout the narrative, implies prospective discovery of novel scaffold cores for AKR1C3. This implication is not supported by the data. While compounds S34 1035 and S34 1040 are described as “newly designed” and part of a “prospective validation,” the authors explicitly state that “structurally related compounds corresponding to both scaffolds can be found dispersed across the training set” and that the designed compounds “occupy a chemical space proximal to AKR1C3 training set molecules.” Moreover, an independent analysis of ChEMBL35 performed by the reviewers surfaced close analogs of the same core scaffold exemplified in the “prospective validation” section of the manuscript. Specifically, compound S07-2001 (CHEMBL5197742) not only shares the same scaffold (pyrrole carboxamide) but differs only by few atoms at the two ends of the molecule from S34 1035 and S34 1040 (methyl instead of cyclopropyl on one end and methyl sulfonamide instead of a nitrile on the other end). S07-2001 is reported to have 2 micromolar IC50 AKR1C3 activity. I surmise it was excluded from the training set due to the imposed, arbitrary 1–5 µM activity threshold rather than chemical novelty. However, the scaffold and close analogs with AKR1C3 activity were in fact known before the study was done (S07-2001 was added to CHEMBL in 2022, ACS Med. Chem. Lett. 2022, 13, 1286–1294). Although the authors do not explicitly claim novelty of the scaffold, the manuscript title and prospective framing convey an impression of novel scaffold discovery that is misleading and factually incorrect. The results instead demonstrate merely successful prioritization and enrichment within a well established chemical space, and the manuscript would benefit from making this clear in the title and text to distinguish procedural prospectiveness from genuine chemical or scaffold novelty. As the TAME-VS platform was already retrospectively validated on a selection of ten diverse protein targets in the past by the authors (see reference 18), I see little added value in publishing yet another example of what amounts to demonstrating the ability to surface very close analogs of known chemical matter in a virtual screening hit finding campaign.

In addition to the major concern above I have the following comments:

When introducing ligand-based (especially fingerprint-based) virtual screening , cite a well-recognized and relevant review on the topic, e.g., Muegge & Mukherjee Expert Opin Drug Discov 11:136 (2016).

The authors make claims about gains in classification accuracy and overall model quality on lines 175-177. Those claims will need to be supported by statistical analyses of paired difference distributions to establish probability of superiority when comparing the models. I recommend A. Nicholls’ paper as reference (J Comput Aided Mol Des 30:103–126 (2016)) to review relevant methodology.

Reviewer #2

(Remarks to the Author)
I co-reviewed this manuscript with one of the reviewers who provided the listed reports. This is part of the Communications Chemistry initiative to facilitate training in peer review and to provide appropriate recognition for Early Career Researchers

who co-review manuscripts.

Reviewer #3

(Remarks to the Author)
Qiao et al. present the application of a target-driven machine learning–enabled virtual screening framework (TAME-VS) to the discovery of AKR1C3 inhibitors. The study integrates ML embedded VS workflow, prospective prioritization under synthesis constraints, biochemical validation, and phenotypic reversal of doxorubicin resistance. Overall, the work is well organized, logically structured, and addresses two practical questions in ML-enabled drug discovery: scaffold generalization and prospective enrichment efficiency. The prospective validation and full synthesis of the designed set are particularly commendable, as this level of experimental completeness is still uncommon in AI-enabled screening studies. The following are specific comments and questions to share.

1. The study is a combination of retrospective and prospective validations. It would be helpful to assess and report the maximum and average Tanimoto similarity between retrospective validation compounds and the training set. A quantitative statement/discussion confirming scaffold-level independence would strengthen the generalization claim.
2. Docking was performed using Glide SP and followed by 100 ns MD. While standard, The MD analysis is descriptive rather than quantitative. RMSD evolution, hydrogen-bond occupancy statistics, etc. should be supplied.
3. Given the high sequence identity among AKR1C isoforms, a brief discussion on selectivity would be valuable. Discussing structural determinants of potential selectivity based on pocket differences would strengthen translational relevance.
4. For statistics in Figure 3C and Figures 5K-N, reporting exact p-values alongside significance thresholds would improve transparency.
5. Regarding the web server deployed, would it be possible to have the AKR1C3 included as an example?
6. Through assessing the TAME-VS online portal, the reviewer is curious about whether the outcome can be persisted for re-visit later. A minor suggestion is about potentially adding an user login that finished VS runs can be persisted for a certain amount of time to enable the re-visit of the outcome.
7. The reviewer agree that an open source online portal of TAME-VS will benefit a larger community of experimental drug discovery scientists. Currently, a 50K library is the default option. Is there computational resource limitation for large-scale screening? The capacity and the limitation of the computational resources that have been used to support the TAME-VS at the authors' end should be specified.

Reviewer #4

(Remarks to the Author)
This manuscript describes an ML-guided virtual screening workflow (TAME-VS) applied to the identification of AKR1C3 inhibitors. The integration of ML-based prioritization with prospective compound synthesis and biochemical/cellular testing is a strength. Inclusion of homolog-derived ligands during model training represents a practical strategy for expanding sparse activity datasets, and the development of an open-source GUI-based pipeline could facilitate broader adoption in early-stage drug discovery. However, several aspects of the modeling assumptions and experimental validation limit the strength of the conclusions. In particular, the biological interpretation of the activity thresholds used for model training, the mechanistic attribution of the observed cellular phenotype to AKR1C3 inhibition, and the presentation and interpretation of the prospective validation require further clarification.

Activity thresholds used for model training

A central methodological feature of this study is the use of binary classification labels based on IC_{50} thresholds, where compounds with IC_{50} < 1 µM are defined as active and compounds above 10 µM are inactive. While such thresholds are commonly used in ligand-based modeling, the biological interpretation of a 1 µM cutoff deserves further discussion. At concentrations near 1 µM, most small molecules will interact with multiple cellular targets unless they are highly optimized and selective. As a result, compounds labeled as "active" in the training set may not be acting exclusively through AKR1C3. This raises the possibility that the model may be learning chemical features associated with general enzyme inhibition or promiscuous binding motifs rather than features specific to AKR1C3 engagement. Given that the study ultimately links inhibitor activity to a cellular phenotype (restoration of doxorubicin sensitivity), the authors should more clearly justify the choice of the 1 µM activity threshold.

Link between biochemical inhibition and cellular phenotype

The manuscript reports that two novel compounds restore doxorubicin sensitivity in a resistant breast cancer cell line. While this result is potentially interesting, the experiments presented do not demonstrate that the observed cellular phenotype arises from on-target inhibition of AKR1C3. The current evidence relies on biochemical inhibition of recombinant AKR1C3 combined with cell viability and clonogenic assays. However, these data do not establish that the compounds engage AKR1C3 in cells or that the observed chemosensitization is mediated specifically through this target. Given the modest potency threshold used in the training data and the possibility of off-target effects, additional validation would strengthen the mechanistic conclusions. For example, experiments demonstrating cellular target engagement, genetic perturbation of AKR1C3, or selectivity profiling within the AKR1C enzyme family would help confirm that the phenotype arises from AKR1C3 inhibition rather than alternative mechanisms.

Interpretation of the prospective validation

Because the full set of 41 compounds was ultimately synthesized and shows a high baseline hit rate (28/41 active), the design space already appears strongly enriched for active molecules. Under these conditions, the practical impact of model-based prioritization is difficult to interpret, as most compounds within the design set are already active.

Analysis of false positives

The reported 92.8% hit rate is based on only 14 prioritized compounds and should therefore be interpreted cautiously. Given the small sample size and the already high baseline activity rate across the full design set (28/41, 68.3%), it is unclear whether the observed enrichment reflects a robust advantage of model-based prioritization or simply ranking within an already highly enriched chemical series.

In summary, the study presents an interesting workflow for ML-guided screening. However, the manuscript would benefit from clearer discussion of the activity thresholds used for model training, stronger mechanistic evidence linking the compounds to AKR1C3-dependent cellular effects, and clarification of the prospective validation strategy and model retraining results. Addressing these points would strengthen the interpretation of the work and its relevance for ML-driven drug discovery.

Version 1:

Reviewer comments:

Reviewer #1

(Remarks to the Author)
The authors have substantially revised the manuscript in response to the reviewers' comments. In particular, the revisions directly and convincingly address the major conceptual and framing concerns i had raised previously regarding claims of novelty, scaffold discovery, and the interpretation of "prospective" validation. The current version is significantly improved in clarity, statistical rigor, and positioning relative to prior work, and it now presents a balanced, accurate account of the contribution. In its revised form, the manuscript no longer overstates chemical or scaffold novelty and instead focuses appropriately on model-guided prioritization within a medicinal chemistry–defined design space, coupled with an experimentally closed-loop validation. The level of experimental follow-up, transparency around human-driven decisions, and rigor of the statistical analyses are now appropriate for publication in Communications Chemistry.

Reviewer #2

(Remarks to the Author)
I co-reviewed this manuscript with one of the reviewers who provided the listed reports. This is part of the Communications Chemistry initiative to facilitate training in peer review and to provide appropriate recognition for Early Career Researchers who co-review manuscripts.

The revised manuscript represents a substantial improvement over the original submission. The authors have clearly addressed earlier concerns regarding novelty and scope, particularly by removing any implication of scaffold discovery and reframing the work around model-guided prioritization within a defined medicinal-chemistry space. The inclusion of relevant reference compounds and closely related AKR1C3 studies provides appropriate context for the experimental results, and the expanded discussion of evaluation metrics in the main text moves the analysis beyond recall-centric claims. In addition, the explicit description of human-driven scaffold and substituent selection is a welcome change, as it improves transparency and avoids overstating the role of the machine-learning models in the overall decision-making process.

One remaining conceptual point concerns how best to benchmark and interpret the contribution of the machine-learning models themselves. While the prospective results clearly demonstrate enrichment through prioritization, it remains challenging to assess to what extent the models are learning target-specific structure–activity features that are distinct from the medicinal-chemistry bias introduced by the human-defined design space. The analogs explored are, as expected, constructed from components related to the training data, which complicates claims that the models are generating newly created decision-making "equity" rather than reinforcing expert priors. This does not invalidate the results, but it does suggest that the primary value demonstrated here is effective ranking within a constrained, expert-designed chemical space rather than emergent chemical novelty.

As an additional suggestion, the authors may wish to frame this limitation more explicitly and note directions for future work. For example, further analysis of which functional groups or physicochemical features drive model prioritization, or evaluation of model behavior in less human-biased or partially model-generated chemical spaces, could provide deeper insight into whether true scaffold hopping or structural novelty emerges when design constraints are relaxed. Such discussion would strengthen the conceptual clarity of the study without changing its scope. With these considerations acknowledged, I believe the revised manuscript presents a clear, balanced, and experimentally well-supported contribution and is suitable for acceptance in its current revised form.

Reviewer #3

(Remarks to the Author)
The authors have revised the manuscript accordingly to address comments and concerns raised in the initial review particularly on reproduceable MD simulations and expanded experimental validation of isoform selectivity. The reviewer has no additional comments to add. The responses are thorough and the manuscript is suitable for publication in its current form.

Reviewer #4

(Remarks to the Author)
The authors have addressed major comments raised in the initial review. The revised manuscript provides additional clarification regarding the model activity thresholds, the interpretation of the validation and its limitations. Additional discussion of false positives and the statistical interpretation of the reported enrichment strengthens the manuscript.

Some mechanistic questions regarding on-target cellular activity and selectivity remain for future study, but the authors have adjusted claims and clarified scope. The revision presents a useful workflow for virtual screening with prospective experimental validation and will be of interest to drug discovery folks.

Thus, I think the manuscript is suitable for publication in Communications Chemistry.

Open Access This Peer Review File is licensed under a Creative Commons Attribution 4.0 International License, which permits use, sharing, adaptation, distribution and reproduction in any medium or format, as long as you give appropriate credit to the original author(s) and the source, provide a link to the Creative Commons license, and indicate if changes were made.
In cases where reviewers are anonymous, credit should be given to 'Anonymous Referee' and the source.
The images or other third party material in this Peer Review File are included in the article's Creative Commons license, unless indicated otherwise in a credit line to the material. If material is not included in the article's Creative Commons license and your intended use is not permitted by statutory regulation or exceeds the permitted use, you will need to obtain permission directly from the copyright holder.
To view a copy of this license, visit https://creativecommons.org/licenses/by/4.0/

Detailed response to the reviewers

We thank the reviewers for their interest regarding our manuscript, and we appreciate the very helpful suggestions. We have comprehensively addressed each of the reviewers' comments below and have provided a revised manuscript.

Reviewer #1

Comment #1: Nature journals explicitly discourage priority, or novelty claims that cannot be robustly verified. The framing of the manuscript, beginning with the title and reinforced throughout the narrative, implies prospective discovery of novel scaffold cores for AKR1C3. This implication is not supported by the data. While compounds S34 1035 and S34 1040 are described as "newly designed" and part of a "prospective validation," the authors explicitly state that "structurally related compounds corresponding to both scaffolds can be found dispersed across the training set" and that the designed compounds "occupy a chemical space proximal to AKR1C3 training set molecules." Moreover, an independent analysis of ChEMBL35 performed by the reviewers surfaced close analogs of the same core scaffold exemplified in the "prospective validation" section of the manuscript. Specifically, compound S07-2001 (CHEMBL5197742) not only shares the same scaffold (pyrrole carboxamide) but differs only by few atoms at the two ends of the molecule from S34 1035 and S34 1040 (methyl instead of cyclopropyl on one end and methyl sulfonamide instead of a nitrile on the other end). S07-2001 is reported to have 2 micromolar IC50 AKR1C3 activity. I surmise it was excluded from the training set due to the imposed, arbitrary 1–5 μM activity threshold rather than chemical novelty. However, the scaffold and close analogs with AKR1C3 activity were in fact known before the study was done (S07-2001 was added to CHEMBL in 2022, ACS Med. Chem. Lett. 2022, 13, 1286−1294). Although the authors do not explicitly claim novelty of the scaffold, the manuscript title and prospective framing convey an impression of novel scaffold discovery that is misleading and factually incorrect. The results instead demonstrate merely successful prioritization and enrichment within a well established chemical space, and the manuscript would benefit from making this clear in the title and text to distinguish procedural prospectiveness from genuine chemical or scaffold novelty. As the TAME-VS platform was already retrospectively validated on a selection of ten diverse protein targets in the past by the authors (see reference 18), I see little added value in publishing yet another example of what amounts to demonstrating the ability to surface very close analogs of known chemical matter in a virtual screening hit finding campaign.

Thank you for this detailed and thoughtful assessment. We appreciate the reviewer's careful examination of the chemical space and the positioning of the work, and we agree that the framing of novelty should be precise and fully supported by the data. In particular, we agree that the chemical scaffolds explored in this study are not entirely unprecedented in a strict cheminformatics sense. As correctly noted, we previously reported a set of AKR1C3 inhibitors, including **S07-2001** (*ACS Med. Chem. Lett.* 2022, 13, 1286–1294), which served as validated starting points for subsequent optimization. In the revised manuscript, we have explicitly

clarified the origin of both Scaffold #1 and Scaffold #2 (**Line 257-271** in **Results**), as well as the design rationale underlying the construction of the 41 prospective compounds (**Line 272-291** in **Results**). These compounds should therefore be interpreted as systematic expansions and refinements of validated chemotypes rather than de novo scaffold discovery.

To avoid any potential misinterpretation, we have revised the title and corresponding text to remove any implication of scaffold-level novelty and to more accurately reflect the scope of the work. Specifically, phrases suggesting novel scaffold discovery or novel chemotypes have been removed or replaced with wording that emphasizes model-guided prioritization within a defined chemical space. The title has been revised to focus on target-driven machine learning–enabled prioritization and prospective validation, rather than scaffold novelty. In the Abstract and main text, descriptions of the designed compounds have been clarified as "newly designed derivatives" or "optimized compounds derived from validated chemotypes," and we explicitly state that both scaffolds originate from previously reported hits. These changes ensure that the manuscript accurately distinguishes procedural prospectiveness from chemical novelty.

At the same time, we would like to clarify that this study demonstrates how a target-driven machine learning framework can be used to guide compound prioritization and improve experimental decision-making under realistic medicinal chemistry constraints. In this context, **S07-2001** represents a functionally validated but suboptimal starting point, with limitations in potency and selectivity. Building upon this scaffold, the present study identifies **S34-1035** and **S34-1040**, which exhibit substantially improved biochemical potency (IC_{50} = 0.04 ± 0.01 μM and 0.03 ± 0.01 μM, respectively) and markedly enhanced subtype selectivity within the AKR1C family (both >2500-fold over AKR1C1, AKR1C2, and AKR1C4), compared to the parent compound **S07-2001** (IC_{50} = 2.08 ± 1.26 μM; selectivity >48-fold). In the revision, the selectivity profile within the closely related AKR1C family was conducted (new **Table 3**, **Line 373-378**). These results indicate that model-guided prioritization can effectively navigate a chemically constrained design space to identify compounds with improved activity profiles.

Regarding the comment that structurally related compounds are present in the training set or public databases, we would like to clarify that the training set did not contain structurally diversified analogs of the **S07-2001**-derived pyrrole carboxamide scaffold, and therefore did not provide sufficient coverage to enable trivial memorization or direct interpolation. As such, the model was not trained on this specific chemotype, and its ability to prioritize optimized derivatives within this scaffold family reflects learned activity-relevant features rather than direct memorization of closely related analogs.

We also respectfully note that while the TAME-VS framework has been retrospectively validated, this does not diminish the value of its application in a fully prospective, experimentally closed-loop setting. The field requires demonstrations that such workflows can (i) generalize to chemotypes that were not part of the bioactivity snapshot used for training, and (ii) prospectively enrich actives under synthesis constraints, relative to a rational medicinal chemistry baseline, rather than only improving retrospective metrics. In the present study, the platform is used to support a complete hit-to-lead prioritization campaign, including data retrieval, model training, retrospective validation using temporally separated datasets, prospective ranking within a designed compound set, and full synthesis and biological evaluation of all 41 compounds. This design enables a direct comparison between the intrinsic activity distribution of the design

space and the subset prioritized by the model, thereby quantifying the added value of model-guided prioritization under synthesis constraints. Compared to our previous retrospective validation across multiple targets, the present study establishes a fully closed-loop, target-specific application that integrates design, prioritization, synthesis, and biological validation within a single coherent framework.

We have implemented a series of revisions throughout the manuscript to address concerns and to improve clarity and rigor. These include:

1. Revision of the title and narrative framing.
   We have revised the title and corresponding text to remove any implication of scaffold-level novelty.

2. Incorporation of the established reference for ligand-based virtual screening.
   LBVS was better introduced with the relevant reference of Muegge & Mukherjee *Expert Opin Drug Discov* 11:136 (2016). **Line 47-51** in **Introduction** and **Line 468-470** in **Discussion**.

3. Careful statistical analyses following Nicholls' methodology.
   We applied the Holm–Bonferroni step-down correction (α = 0.05) and computed Cohen's d as a sample-size-independent measure of effect size following A. Nicholls' paper as reference (*J Comput Aided Mol Des* 30:103–126 (2016)). The text on **Line 175-177** has been revised accordingly to ensure claims are appropriately supported by the statistical evidence. In **Methods**, **Line 582-586**, the section of "Molecular featurization and fingerprint generation" was updated to specify this statistical analysis.

4. Comparison with closely related AKR1C3 studies.
   The manuscript should cite and compare its methodology and results to A closely related AKR1C3 AIDD study, *ACS Medicinal Chemistry Letters* 15.8 (2024): 1269-1278, was cited and compared. **Line 89-93** in **Introduction** and **Line 467-474** in **Discussion**.

5. Benchmarking against reported inhibitors.
   Supplementary Table 9 was compiled in revision with representative published AKR1C3 inhibitors, their reported biochemical activities, and their key limitations. We have revised the manuscript to discuss this prior landscape more explicitly and to clarify the motivation for synthesizing new analogs in this chemical space.

6. Clarification of human-driven design decisions.
   We clarified the rationale behind selecting the two scaffolds and their corresponding substitution strategies (**Line 257-271** in **Results),** and their corresponding substitution strategies (**Line 272-291** in **Results**).

7. Expansion of computational validation including replicate MD simulations.
   We have expanded the MD analysis to improve both quantitative rigor and reproducibility. Specifically, the simulations were extended from a single trajectory to three independent replicate simulations (100 ns each) for both **S34-1035** and **S34-1040**.

8. Subtype selectivity profiling of prioritized compounds.
   We continued experimental selectivity profiling of prioritized compounds to validate AKR1C3 preference within the AKR1C family (new **Table 3**). **Line 373-378** in **Results**.

9. Explicit reporting of statistical significance values.
   In the revised manuscript, legends of **Figure 3** and **Figure 5** were updated to have exact p-values specified.

10. Further development of the TAME-VS web server to enhance reproducibility and accessibility.

    (1) We incorporated AKR1C3 as a dedicated example on our web server. We provided download links (highlighted in blue at the top of the page) for all result files associated with this example. They can be downloaded at any time for reference or reproduction purposes.

    (2) We implemented a result persistence mechanism. Once a submitted job is completed and the result files are generated, a link will be remained valid for re-visit for 7 days.

    (3) In addition to the default Enamine Diversity 50K library for screening, we have now included the Enamine Screening 4M library as an additional option.

Etc.

Together, these revisions aim to present the work more accurately as an experimentally validated, target-driven framework for model-guided prioritization in early-stage drug discovery. We hope that this revised presentation clarifies the intended scope of the study and addresses the reviewer's concerns regarding novelty and contribution.

Comment #2: When introducing ligand-based (especially fingerprint-based) virtual screening , cite a well-recognized and relevant review on the topic, e.g., Muegge & Mukherjee Expert Opin Drug Discov 11:136 (2016).

Thank you for highlighting the importance of incorporating a well-recognized review when introducing ligand-based virtual screening. We appreciate the suggestion and update the Introduction section accordingly:

> **Line 47**: *Molecular fingerprints, particularly two-dimensional representations, remain among the most widely used descriptors in ligand-based virtual screening due to their efficiency and strong empirical performance, including in identifying compounds with novel scaffolds, and are often integrated with complementary information such as pharmacophore or structural features in modern workflows[12].*

Modifications are made in Discussion as well to emphasize the utility remains contingent on the availability of well-curated activity labels:

> **Line 485-487**: *Although molecular fingerprints are widely used as descriptors in predictive modeling due to their interpretability and accessibility, their utility remains contingent on the availability of well-curated activity labels and appropriate model training strategies[12].*

Changes are highlighted in the revised manuscript.

Comment #3: The authors make claims about gains in classification accuracy and overall model quality on lines 175-177. Those claims will need to be supported by statistical analyses of paired difference distributions to establish probability of superiority when comparing the models. I recommend A. Nicholls' paper as reference (J Comput Aided Mol Des 30:103–126 (2016)) to review relevant methodology.

Thank you for pointing out that statistical analyses of paired difference are critical to make claims about overall model qualities. We agree on it and appreciate your recommendation of Nicholls' paper as a reference.

This part originally described the performance comparison of avaialble of fingerprint types. **Supplementary Figure 1** and **Supplementary Tables 3 and 4** denote the metrics (AUC, Precision, Recall, Specificity, F1 score, Geometric mean, and index of balanced accuracy) for the quantitative evaluation. Here, according to A. Nicholls' papers (*J Comput Aided Mol Des 30:103–126 (2016)*) and (*J Comput Aided Mol Des 28:887–918 (2014)*), we performed rigorous statistical analyses to re-assess our claims regarding model performance using different fingerprint types.

To control the Family-Wise Error Rate (FWER) across the six pairwise comparisons within each strategy, we applied the Holm-Bonferroni step-down correction ($\alpha = 0.05$). Beyond statistical significance, we also computed Cohen's d as a sample-size-independent measure of effect size, and estimated the probability of superiority $P(A > B) \approx 0.25|d| + 0.5$ as specified in *J Comput Aided Mol Des 30:103-126 (2016)*. Using the analysis on AUCs of RF models as an example (**Response Table 1**), while slight variations in mean AUC were observed (ranging from 0.914 to 0.967 across strategies), no statistically significant differences were detected between any pair of fingerprints after Holm-Bonferroni correction for multiple comparisons.

Response Table 1. Holm–Bonferroni step-down correction pairwise comparisons for AUC of trained RF models under different fingerprint types.

Strategy	Metric	FP_A	FP_B	Mean_A	Mean_B	Δ(A-B)	t	df	p_value	Cohen's_d	P(A>B)	HB_threshold	Significant	Effect_size
Strategy #1	AUC	Atompair	MACCS	0.914	0.926	-0.012	-0.785	12.6	0.4469	-0.351	0.588	0.008	FALSE	small
Strategy #1	AUC	Morgan	Atompair	0.924	0.914	0.01	0.606	15.3	0.5532	0.271	0.568	0.01	FALSE	small
Strategy #1	AUC	Atompair	Topological	0.914	0.921	-0.007	-0.394	17.1	0.6986	-0.176	0.544	0.0125	FALSE	negligible
Strategy #1	AUC	Topological	MACCS	0.921	0.926	-0.005	-0.392	14.3	0.7007	-0.175	0.544	0.016	FALSE	negligible
Strategy #1	AUC	Morgan	Topological	0.924	0.921	0.003	0.212	17.2	0.8349	0.095	0.524	0.025	FALSE	negligible
Strategy #1	AUC	Morgan	MACCS	0.924	0.926	-0.002	-0.184	16.3	0.8564	-0.082	0.521	0.05	FALSE	negligible
Strategy #2	AUC	Morgan	MACCS	0.945	0.926	0.019	1.633	17.9	0.12	0.73	0.683	0.008	FALSE	medium
Strategy #2	AUC	Topological	MACCS	0.942	0.926	0.016	1.559	15.7	0.1389	0.697	0.674	0.01	FALSE	medium
Strategy #2	AUC	Morgan	Atompair	0.945	0.934	0.011	1.024	17.9	0.3195	0.458	0.614	0.0125	FALSE	small
Strategy #2	AUC	Atompair	Topological	0.934	0.942	-0.008	-0.866	17	0.3984	-0.387	0.597	0.016	FALSE	small
Strategy #2	AUC	Atompair	MACCS	0.934	0.926	0.008	0.713	17.6	0.4851	0.319	0.58	0.025	FALSE	small
Strategy #2	AUC	Morgan	Topological	0.945	0.942	0.003	0.308	16.4	0.762	0.138	0.534	0.05	FALSE	negligible
Strategy #3	AUC	Topological	MACCS	0.967	0.965	0.002	0.335	15.1	0.7421	0.15	0.537	0.008	FALSE	negligible
Strategy #3	AUC	Atompair	Topological	0.965	0.967	-0.002	-0.297	17.7	0.7696	-0.133	0.533	0.01	FALSE	negligible
Strategy #3	AUC	Morgan	MACCS	0.966	0.965	0.001	0.175	15.7	0.863	0.078	0.52	0.0125	FALSE	negligible
Strategy #3	AUC	Morgan	Atompair	0.966	0.965	0.001	0.154	17.9	0.8792	0.069	0.517	0.016	FALSE	negligible
Strategy #3	AUC	Morgan	Topological	0.966	0.967	-0.001	-0.144	17.9	0.887	-0.064	0.516	0.025	FALSE	negligible
Strategy #3	AUC	Atompair	MACCS	0.965	0.965	0	0	16.3	1	0	0.5	0.05	FALSE	negligible

The analysis for all metrics of both RF and MLP models continues and attached as a separate **supplementary data file Pairwise_fingerprint_comparisons_for_all_metrics**. No statistically significant differences were detected. The progressive improvement in model performance from Strategy #1 to Strategy #3 reflects the effect of training set composition rather than fingerprint choice. The text on **lines 175-177** has been revised accordingly to ensure that these claims are appropriately supported by the statistical evidence presented above:

> **Line 175**: *While Morgan fingerprints were used as the default representation, TAME-VS also supports AtomPair, topological, and MACCS fingerprints. A comprehensive evaluation across the three strategies and four fingerprint types, each with ten-fold cross-validation for both RF (**Supplementary Table 3** and **Supplementary Figure 1K**) and MLP models (**Supplementary Table 4** and **Supplementary Figure 1J**), revealed that no statistically significant differences (Holm–Bonferroni step-down correction) in predictive performance were observed between fingerprint types after correction for multiple comparisons (**Supplementary data file**), despite modest variations in mean metric values. These results indicate that model performance is primarily driven by training set composition and label definition rather than the specific fingerprint representation used.*

In Methods, the section of "Molecular featurization and fingerprint generation" was updated to specify this statistical analysis:

> **Line 583**: *To control the Family-Wise Error Rate (FWER) across the six pairwise comparisons within each strategy, the Holm–Bonferroni step-down correction ($\alpha = 0.05$) was applied[27]. Beyond statistical significance, Cohen's d was computed as a sample-size-independent measure of effect size.*

Changes are highlighted in the revised manuscript.

Reviewer #2

Comment #1: The authors describe a set of machine-learning models integrated into a previously published, publicly accessible web platform for exploring analogs of AKR1C inhibitors. Their approach relies on traditional ML methods trained on small-molecule activity data across AKR1C isoforms, similar to strategies reported in recent literature. They manually curate two potent scaffold families and define a synthetically accessible set of substituents, which are then used to illustrate the platform's capabilities. Model performance is demonstrated primarily through recall-based evaluation. A small number of predicted compounds are synthesized and tested in biological assays, and the authors propose these as potential leads. Overall, the work represents a conventional virtual-screening workflow paired with limited experimental validation.
The manuscript does not meet the methodological or conceptual standards required for publication in Nature Communications. The machine-learning components lack sufficient benchmarking, quantitative evaluation, and comparison against established baselines, making it difficult to assess the robustness or generalizability of the proposed approach. In addition, the work does not adequately address potency or efficacy benchmarks that are standard in this research area. Finally, the methodological contribution is not sufficiently novel or differentiated from existing literature to justify publication in a high-impact journal.

Thank you for your detailed assessment of the manuscript. We would like to clarify the intended scope, the methodological contribution, and the extent of experimental validation in the present study. We also note that the manuscript is currently under consideration for ***Communications Chemistry*** but not ***Nature Communications***, and we have revised the presentation with this

journal's scope in mind.

Although the workflow integrates established components such as ligand-based machine learning, virtual screening, and medicinal chemistry design, the contribution of this study does not lie in presenting a new standalone algorithm. Rather, it lies in the systematic design and validation of a target-driven framework to address two practical questions in ML-enabled early-stage drug discovery: whether models can generalize beyond familiar chemical neighborhoods to temporally and chemically novel scaffolds, and whether model-guided prioritization can improve experimental decision-making under realistic synthesis constraints. To address these questions, we combined temporally separated retrospective validation, prospective prioritization within a medicinal chemistry design space, full synthesis of the designed compound set, and experimental comparison against the intrinsic activity baseline of that design space. We believe that this integrated evaluation framework goes beyond a conventional virtual-screening workflow.

The reviewer states that model performance is demonstrated primarily through recall-based evaluation. We would like to clarify that in the manuscript, model performance has been assessed using a set of metrics, including ROC-AUC, precision, recall, specificity, F1 score, geometric mean, and index of balanced accuracy, as presented in **Figure 2**, **Supplementary Tables 3 and 4**, and **Supplementary Figure 1**. During the revision, we have further strengthened the quantitative evaluation by incorporating statistical analyses based on paired difference distributions, including Holm–Bonferroni correction, Cohen's d, and probability of superiority following Nicholls' methodology (**Supplementary Data file**). We have revised the Results and Methods sections accordingly to make this more explicit.

The reviewer raises an important point regarding the extent of experimental validation. We would like to clarify that the present study includes decent experimental follow-up. In the present study, all 41 designed compounds were synthesized (**Supplementary Figure S5**) and fully characterized, including NMR and LC–MS data provided in the **Supplementary Information page 22 - page 108**. Their biochemical activities against AKR1C3 were systematically evaluated (**Tables 1, 2, 3, and 4**), and the most promising compounds were further subjected to selectivity profiling within the AKR1C family, molecular modeling and triplicate MD simulations, cell-based viability assays, synergy evaluation with doxorubicin (**Figure 5I-N, and Table 4**), and long-term clonogenic assays (**Figure 5O**). In our view, this level of experimental validation is appropriate to support a typical Medicinal Chemistry campaign.

We also note that the prospective validation was intentionally designed to be more informative than a standard hit-identification exercise. Because the full designed set of 41 compounds was synthesized and tested, the study provides a direct comparison between model-guided prioritization and the baseline activity distribution of the expert-designed chemical space. We agree that the high baseline hit rate indicates that the design space was already enriched by medicinal chemistry knowledge, and we have revised the manuscript to clarify that the value of TAME-VS in this context lies in preferential ranking of active compounds and improved decision-making efficiency under synthesis constraints, rather than discovery from a predominantly inactive background. We believe that this framing more accurately reflects the practical role of machine learning in early-stage discovery.

To better position the manuscript relative to prior literature, we have also added discussion of closely related AKR1C3 studies, including the recent ACS Medicinal Chemistry Letters paper using the AtomNet model as the reviewer suggested. As revised, the manuscript now makes clearer that the prior study and ours are complementary rather than redundant. The earlier work focuses on structure-based deep learning for hit identification from a screening library, whereas our work emphasizes target-driven ligand-based learning, scaffold-level generalization, and enrichment over a medicinal chemistry baseline within a constrained synthesis setting. We have also added a comparative summary of representative published AKR1C3 inhibitors and their limitations to better contextualize the motivation for the newly synthesized compounds.

Finally, we would like to clarify again that this manuscript is submitted to ***Communications Chemistry***. We believe the study aligns well with the scope of Communications Chemistry, which values chemical discovery, methodological rigor, and experimentally validated workflows. The manuscript presents an AI-enabled virtual screening framework, an experimentally grounded AKR1C3 inhibitor discovery campaign, and a publicly accessible online portal that enables broader use of the validated workflow. We have revised the manuscript to make these contributions more explicit and hope that the updated version better reflects the intended positioning and significance of the work.

Your specific comments and suggestions are carefully addressed as follows.

Comment #2: Include and discuss closely related prior work.
The manuscript should cite and compare its methodology and results to the closely related study ACS Med. Chem. Lett. (DOI: 10.1021/acsmedchemlett.4c00150). This earlier work employs a similar machine-learning strategy for AKR1C3 inhibitor discovery, and a direct comparison would help clarify what is novel in the present study and how its performance differs.

Thank you for suggesting a direct comparison with this closely related study on AI accelerated AKR1C3 inhibitor finding. This study applies a structure-based deep learning model (AtomNet) to perform virtual screening against AKR1C3, identifying a novel inhibitor scaffold (compound 4) with submicromolar enzymatic potency and demonstrated antiproliferative and chemo-sensitizing effects in cancer cell models. We agree and appreciate the suggestion to cite and compare.

We have revised the manuscript to clarify the relationship between that study and our work. While both studies leverage machine learning for AKR1C3 inhibitor discovery, they differ in methodological emphasis and evaluation framework. The prior study focuses on structure-based screening for hit identification, whereas our work adopts a target-driven, ligand-based learning strategy and explicitly evaluates (i) generalization to temporally and chemically novel scaffolds and (ii) model-guided enrichment relative to a rational medicinal chemistry baseline under synthesis constraints. Sentences has been inserted to the **Discussion** section:

> **Line 472**: *Under screening conditions, the most valuable models are those that capture activity-determining patterns that transfer across scaffold boundaries, rather than those reproduce similarity search. This perspective complements structure-based AI screening efforts on the same target, where deep learning models trained on protein–ligand*

complexes have been used to identify new AKR1C3 inhibitors from large virtual libraries[24]. In those settings, model success is typically evaluated through the identification of active compounds from a predefined screening space. By contrast, the present work emphasizes whether learning-based models can recognize activity-determining patterns that transfer across chemically and temporally distinct compound sets, providing a more stringent test of scaffold-level generalization.

A brief acknowledgement of this closely related study was also added to the **Introduction**:

Line 89: *Recently, a structure-based deep learning approach using the AtomNet model identified AKR1C3 inhibitors through virtual screening against a crystallographic binding site, followed by experimental validation and demonstration of synergistic effects with chemotherapeutic agents[24]. Such studies highlight the potential of AI-driven screening to expand chemical diversity and uncover biologically active scaffolds in this target class.*

Changes are highlighted in the revised manuscript.

Comment #3: Strengthen experimental benchmarking with established reference compounds.

The biological validation would be more impactful if benchmark AKR1C3 inhibitors were included alongside the newly synthesized compounds.

What are the strongest published AKR1C3 inhibitors, and how do the authors' compounds perform relative to them in the same assays?

What specific limitations of existing inhibitors motivate the synthesis of new analogs in this chemical space?

Providing this context would help justify the experimental direction and clarify the contribution of the new compounds.

Thank you for this helpful suggestion. We agree that benchmarking against established AKR1C3 inhibitors is important for contextualizing the contribution of the newly synthesized compounds. In response, we have compiled representative published AKR1C3 inhibitors, their reported biochemical activities, and their key limitations in a newly drafted **Supplementary Table 9**. This comparison shows that although several prior compounds achieve strong enzymatic potency, many are associated with limitations such as poor subtype selectivity, poor cellular permeability, metabolic instability, high cytotoxicity, or synthetic complexity.

Supplementary Table 9. Representative reported AKR1C3 inhibitors, their biochemical activities, and key limitations relevant to inhibitor development.

Type	Compound structure	Assay type and Value	Limits	Reference
Natural Product Origin	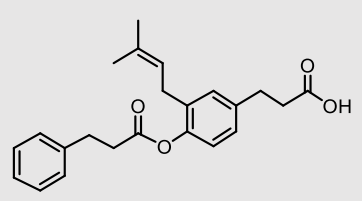	AKR1C3 IC_{50} = 110 nM	Metabolically unstable; synthetically challenging	*Chem.-Biol. Interact.* 2015, 234, 339−348.
	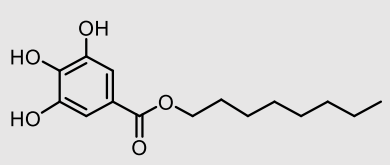	AKR1C3 IC_{50} = 2.56 ± 0.2 μM		*Chem.-Biol. Interact.* 2016, 253,60−65.

	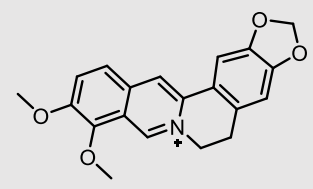	AKR1C3 IC_{50} = 4.08 μM		*Asian J. Androl.* 2016, 18 (4), 607−612.
Organic Ruthenium Complexes	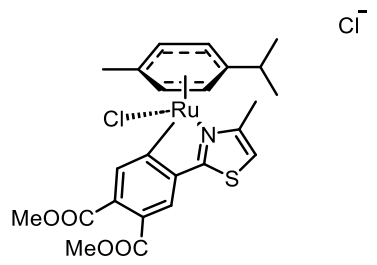	Ki = 560 nM	Poor subtype selectivity; high cytotoxicity	*Chem.-Biol. Interact.* 2015, 234, 349−359.
Off-Label Use Compounds	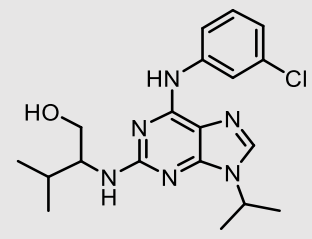	AKR1C3 IC_{50} = 6.58 μM	Weak AKR1C3 activity (μM); severe off-target effects	*Biochem. Pharmacol.* 2018, 156,22−31.
	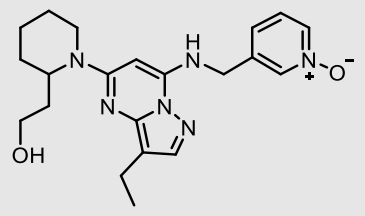	AKR1C3 IC_{50} = 0.23 μM		*Arch.Toxicol.* 2018, 92 (9), 2845−2857.
	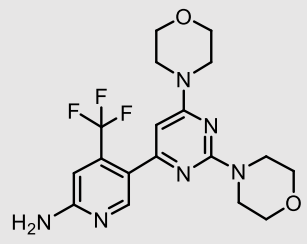	AKR1C3 IC_{50} = 9.5 μM		*Chem.-Biol. Interact.* 2019, 302, 101−107.
	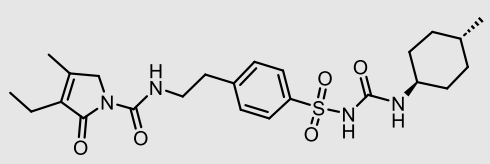	AKR1C3 IC_{50} = 0.85 ± 0.04 μM		*Chem.-Biol. Interact.* 2015, 240, 310−315.
Long-Chain Polyunsaturated Fatty Acids	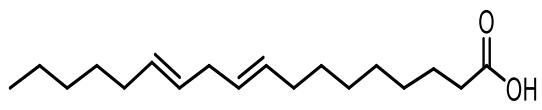	AKR1C3 IC_{50} (when substrate is S-tetralol) = 0.69 μM	Poor subtype selectivity; rapid in vivo metabolism	*J. Biochem.* 2017, 162 (5), 371−379.
Catalytic Substrate Skeletons	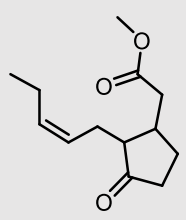	AKR1C3 K_is = 153 ± 28 μM/L	Weak AKR1C3 activity (μM); poor subtype selectivity	*Cancer Res.* 2009, 69 (11), 4769−4775.
NSAID Ana-logues	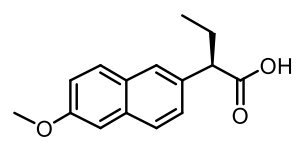	AKR1C3 IC_{50} = 0.11 ± 0.01 μM	Poor cellular permeability	*J. Med. Chem.* 2016, 59 (16), 7431−7444.
	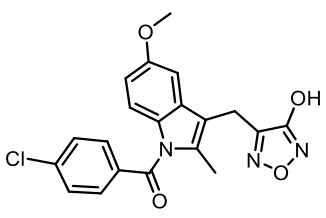	AKR1C3 IC_{50} = 0.30 ± 0.02 μM		*Cancer Cell* 2013, 23, 332−346.
	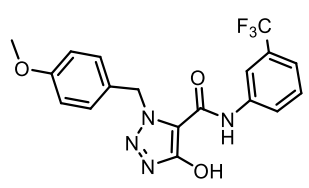	AKR1C3 IC_{50} = 0.31 ± 0.005 μM		*Eur. J. Med. Chem.* 2017, 139, 936−946.
Other AKR1C3 Inhibitors	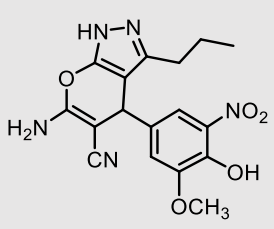	AKR1C3 IC_{50} = 0.160 ± 0.028 μM	Poor subtype selectivity; conformation-al changes at the binding	*Bioorg. Med. Chem.* 2018, 26 (22), 5934−5943.
	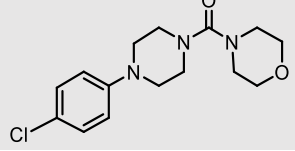	AKR1C3 IC_{50} = 0.11 μM		*Bioorg. Med. Chem.* 2014, 22 (3), 967−977.

	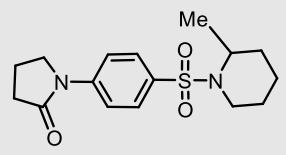	AKR1C3 IC_{50} = 0.094 μM	site	*Eur. J. Med. Chem.* 2013, 62, 738−744.
Bifunctional Inhibitors	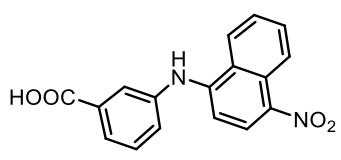	AKR1C3 IC_{50} = 0.086 ± 0.011 μM	Poor subtype selectivity; complex molecular optimization	*Bioorg. Med. Chem. Lett.* 2012, 22 (10), 3492−3497.

We have revised the manuscript to discuss this prior landscape more explicitly and to clarify the motivation for synthesizing new analogs in this chemical space. Our goal was not only to obtain potent AKR1C3 inhibitors, but to identify compounds with a more favorable balance of potency, selectivity, and cellular relevance. In this context, **S34-1035** and **S34-1040** compare favorably with strong published AKR1C3 inhibitors in biochemical potency, while also demonstrating strong selectivity within the AKR1C family (new **Table 3.** Selectivity profiles within the AKR1C family of the selected compounds were further assessed during the revision. Detailed discussions can be found with the responses to the Reviewer 3 Comment 3 and Reviewer 4 Comment 2.) and robust chemosensitizing effects in resistant breast cancer cells. We believe this addition better positions the present work relative to the existing AKR1C3 inhibitor literature.

> **Line 513**: *Existing AKR1C3 inhibitors span multiple chemotypes with strong enzymatic activity, yet practical limitations related to selectivity, cellular properties, or synthetic tractability remain in several cases (**Supplementary Table 9**). The compounds identified here therefore complement the current landscape by providing additional chemically tractable scaffolds with a balanced activity profile.*

Comment #4: Clarify human-driven decisions in the machine-learning workflow.
The manuscript should more fully describe any manual steps in the modeling pipeline, particularly the rationale for selecting the two scaffold families and the chosen substituent set. Since these decisions shape the model's chemical search space, transparency is essential for reproducibility and for evaluating the generality of the approach.

Thank you for raising the concern that human-driven decisions should be clarified. We agree that further specifying manual steps will increase the transparency and benefit the assessment of reproducibility and the generality of the approach. We revised the section of “Prospective validation of TAME-VS in prioritizing newly designed AKR1C3 inhibitors” section in the Results to clarify the rationale behind selecting the two scaffolds and their corresponding substitution strategies.

> For the scaffold #1, **Line 257**: *Scaffold #1 was derived from our previously identified hit molecule **S07-2008**. This scaffold was selected based on its validated pharmacophore, in which a benzamide carbonyl anchors at the oxyanion site (Tyr55 / His117 / NADP$^+$), while an isoxazolyl ether moiety occupies the SP1/SP2 hydrophobic subpockets, as confirmed by co-crystal structures with AKR1C3. Notably, SP1 is a characteristic subpocket of AKR1C3, and interactions within this region are critical for achieving inhibitor selectivity.*

Importantly, this scaffold avoids the presence of a free carboxylic acid, thereby circumventing MCT1-dependent cellular uptake limitations associated with classical NSAID-derived inhibitors, while also eliminating off-target cyclooxygenase (COX) inhibition.

For the scaffold #2, **Line 265**: *Scaffold #2 was derived from our previously identified hit molecule **S07-2001**[29]. It was selected as a structurally distinct, non-carboxylate alternative in which the pyrrole ring serves as a rigid central linker and the N-acyl carbonyl at R1 acts as a carboxylic acid bioisostere to engage the oxyanion pocket, thereby maintaining the essential anchor interaction while improving membrane permeability. The complementary structural features of these two scaffolds were designed to ensure broad coverage of the AKR1C3 binding pocket topology, particularly the differential occupation of the SP1 cavity defined by Ser118, Phe306, Phe311, and Tyr319.*

For Scaffold #1, 17 derivatives were proposed by systematically varying the amide-linked aromatic ring to modulate steric bulk, electronic properties, and hydrogen-bonding capacity. For Scaffold #2, four substitution positions (R1-R3 and X) on the pyrrole-carboxamide core were explored to design 24 derivatives, enabling combinatorial modulation of sterics and polarity.

Line 272: *For Scaffold #1, the R-group library at the aniline position was deliberately constructed to span four chemically distinct subspaces: (i) nitrogen-containing heteroaromatics (pyridines, pyrimidines, pyridazines, triazines), selected to improve aqueous solubility, introduce additional hydrogen-bond acceptors capable of contacting Asn167 or Ser118, and reduce cytochrome P450-mediated aromatic hydroxylation; (ii) substituted phenyl rings with varying substitution patterns (ortho > meta > para preference), motivated by the known V-shaped binding conformation of this scaffold class in which ortho substitution promotes a dihedral twist enabling simultaneous occupancy of the SP1–SP2–SP3 subpockets; (iii) halogenated phenyl groups (F, Cl), selected as electron-withdrawing, metabolically blocking substituents with minimal steric impact; and (iv) morpholine- and piperazine-containing flexible chains, included to probe the effect of ionizable nitrogen atoms on cellular permeability and to reach the deeper SP3 region.*

For Scaffold #2, the R1 position (N-acyl group) was varied across small cyclic ketones (cyclopropyl, cyclobutyl, isobutyl, acetyl), since strained carbocycles confer favorable CH–π contacts and conformational rigidity that reduces entropic binding penalties. The R2 position (C-terminal aryl group) was diversified across large fused aromatic systems (naphthyl, biphenyl, indolyl), methylenedioxy-fused phenyl groups (with and without fluorine), and polar-substituted benzyl ethers (methoxy, cyano), in order to systematically probe π–π stacking interactions with Phe306/Phe311 and differential occupancy of the wider SP1 subpocket unique to AKR1C3 (defined by Ser118 versus Phe118 in AKR1C2). The R3 and R4 methyl/hydrogen variants at the pyrrole 3- and 4-positions were included to evaluate the effect of local steric perturbation on the planarity and dihedral angles of the central linker, which in turn governs the spatial orientation of both terminal groups.

Changes are highlighted in the revised manuscript.

Comment #5: Revise and expand machine-learning performance metrics.

The current reliance on recall alone is insufficient for evaluating model quality or for supporting compound triage from large chemical libraries. Recall can be trivially maximized by predicting every compound as a hit.
Metrics such as F1 score, Matthews correlation coefficient (MCC), precision, and ROC-AUC are standard in cheminformatics and should be included to provide a balanced assessment of model performance. These additions would bring the evaluation in line with industry and academic best practices.

Thank you for emphasizing the importance of using multiple evaluation metrics for assessing model performance. We fully agree that reliance on a single metric such as recall alone would be insufficient for evaluating classification models or supporting compound prioritization.

In the current manuscript, model performance has already been evaluated using a comprehensive set of metrics, including ROC-AUC, precision, recall, specificity, F1 score, geometric mean (Geo), and index of balanced accuracy (IBA), as presented in **Figure 2**, **Supplementary Tables 3 and 4** and **Supplementary Figure 1**. These metrics collectively provide a balanced assessment of model performance, capturing discrimination ability, precision–recall trade-offs, and robustness to class imbalance.

To avoid any potential misunderstanding, we have revised the corresponding text in the Results section to explicitly highlight the use of these multiple metrics and clarify that model evaluation is not based on recall alone. We believe that this clarification aligns the presentation with current best practices in cheminformatics and addresses your concern.

Comment #6: Reconsider the article structure for the intended journal.
As written, the manuscript does not align with the expectations for Nature Communications, which typically requires a substantial conceptual advance, methodological innovation, or broad scientific impact. The current work is more consistent with a traditional virtual- screening study supported by limited experimental follow-up.

Thank you for your comment regarding article structure and scope. We would like to clarify that the manuscript is currently under consideration for ***Communications Chemistry*** **(**but not ***Nature Communications*****)**, and we have revised the presentation with this journal's scope in mind. We believe the study aligns well with ***Communications Chemistry***, which emphasizes chemical discovery, methodology, and experimentally validated workflows.

Regarding experimental validation, we respectfully note that the manuscript includes extensive experimental follow-up beyond initial virtual screening. Specifically, all involved 41 compounds were synthesized (**Supplementary Figure S5**) and fully characterized (including NMR and LC–MS, **Supplementary information page 22 - page 108**), and their biochemical activities against AKR1C3 were systematically evaluated (**Tables 1, 2, and 3**). In addition, selected compounds were further assessed in cell-based assays, demonstrating restoration of doxorubicin sensitivity and suppression of long-term clonogenic growth (**Figure 5I-N, and Table 4**). A colony formation assay was performed to evaluate the long-term impact of the combination strategy on cell

proliferative capacity (**Figure 5O**). These experiments were designed to provide both quantitative evaluation of model-guided enrichment and functional validation in a biologically relevant context.

To improve clarity, we have revised the manuscript to more explicitly highlight the scope of experimental validation and its role in supporting the computational findings. We hope this revision better reflects the depth of experimental work and the intended positioning of the study within ***Communications Chemistry***.

Reviewer #3

Qiao et al. present the application of a target-driven machine learning–enabled virtual screening framework (TAME-VS) to the discovery of AKR1C3 inhibitors. The study integrates ML embedded VS workflow, prospective prioritization under synthesis constraints, biochemical validation, and phenotypic reversal of doxorubicin resistance. Overall, the work is well organized, logically structured, and addresses two practical questions in ML-enabled drug discovery: scaffold generalization and prospective enrichment efficiency. The prospective validation and full synthesis of the designed set are particularly commendable, as this level of experimental completeness is still uncommon in AI-enabled screening studies. The following are specific comments and questions to share.

Thank you for the positive and encouraging assessment of our work, as well as for the constructive and thoughtful comments. We particularly appreciate the recognition of the prospective validation and complete synthesis of the designed compound set.

We have carefully addressed all specific comments, including those related to quantitative evaluation, subtype selectivity, statistical analysis, and the online server implementation. Detailed responses and corresponding revisions are provided below for each point.

Comment #1: The study is a combination of retrospective and prospective validations. It would be helpful to assess and report the maximum and average Tanimoto similarity between retrospective validation compounds and the training set. A quantitative statement/discussion confirming scaffold-level independence would strengthen the generalization claim.

Thank you for pointing out the importance of quantitatively state the similarity between the retrospective validation compounds and the training set. Original, a systematic similarity comparison was conducted (**Supplementary Figure 3**). Heatmaps were generated to depict Tanimoto similarity scores between active compounds in the strategy #2 training set and retrospective validation compounds, calculated using Morgan molecular fingerprints. we visualized the chemical space using t-SNE embeddings (**Figure 3G**). None of the validation molecules overlap with the training distribution because both series introduce genuinely novel scaffolds. Within the validation set itself, the two chemical series appear as distinguishable clusters, but active and inactive members overlap considerably.

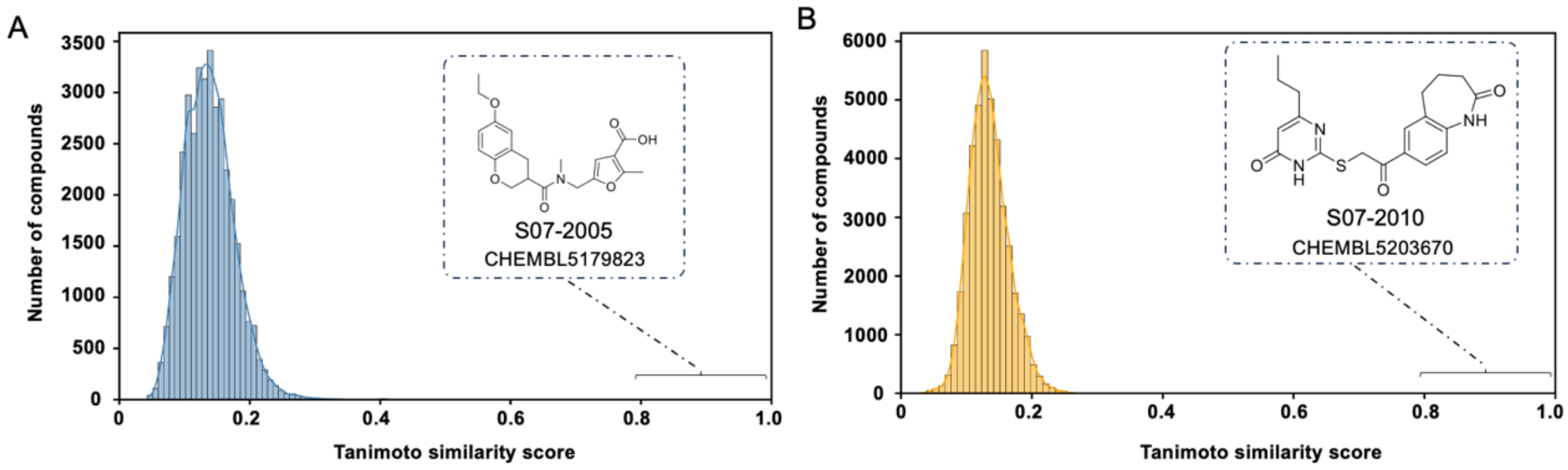

Response Figure 1. similarities between the actives in Strategy #2 and retrospective validation compounds. A. Distribution of Tanimoto similarity scores between 28 compounds from JMC 2025 and the training set compounds used in Strategy #2 with a maximum similarity score of 0.8814 and a mean of 0.1383. **B.** Distribution of Tanimoto similarity scores between 30 compounds from EJMC 2025 and the training set compounds used in Strategy #2 with a maximum score of 0.9636 and a minimum of 0.1350.

Authors agree that although data presented visually illustrates the similarity between retrospective validation compounds and the training set, direct maximum and average similarity numbers will strengthen the generalization claim (**Response Figure 1**). The maximum Tanimoto similarity observed was 0.9636, contributed by **S07-2010**, the parent compound itself, and the average Tanimoto similarity was 0.137. Corresponding changes has been updated in the main text.

> **Line 234**: *As expected, none of the validation molecules overlap with the training distribution because both series introduce genuinely novel scaffolds. Within the validation set itself, the two chemical series appear as distinguishable clusters, but active and inactive members overlap considerably. Tanimoto similarity between retrospective validation compounds and the training set was assessed with an average similarity score of 0.137 (**Supplementary Fig. S3**). The highest similarity score of 0.9636 was contributed by a **S07-2010** analogue (the parent compound).*

The **Supplementary Figure 3** was also updated to include distributions of similarity scores.

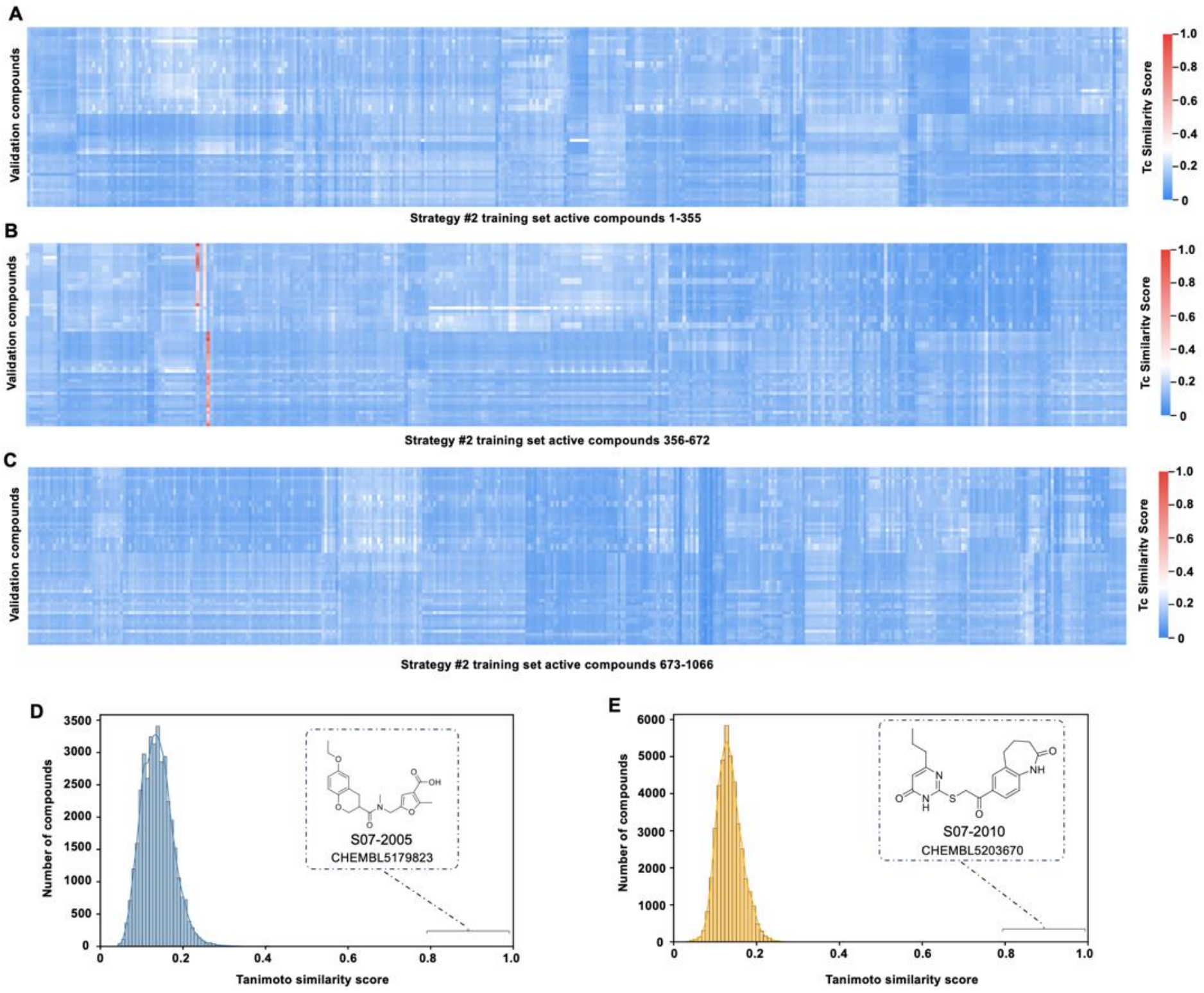

Supplementary Figure 3. Similarities between the actives in Strategy #2 and retrospective validation compounds. **A-C**. Heatmaps depict Tanimoto similarity scores between active compounds in the strategy #2 training set and retrospective validation compounds, calculated using molecular fingerprints. Rows represent training actives and columns represent validation compounds, with color scale indicating similarity magnitude. **D**. Distribution of Tanimoto similarity scores between 28 compounds from JMC 2025 and the training set compounds used in Strategy #2 with a maximum similarity score of 0.8814 and a mean of 0.1383. **E**. Distribution of Tanimoto similarity scores between 30 compounds from EJMC 2025 and the training set compounds used in Strategy #2 with a maximum score of 0.9636 and a minimum of 0.1350.

Comment #2: Docking was performed using Glide SP and followed by 100 ns MD. While standard, The MD analysis is descriptive rather than quantitative. RMSD evolution, hydrogen-bond occupancy statistics, etc. should be supplied.

Thank you for this helpful suggestion regarding the quantitative analysis of molecular dynamics (MD) simulations.

In the revised manuscript, we have expanded the MD analysis to improve both quantitative rigor and reproducibility. Specifically, the simulations were extended from a single trajectory to three independent replicate simulations (100 ns each) for each compound. RMSD evolution profiles are now provided to assess the stability and convergence of the protein–ligand complexes across independent trajectories (new **Supplementary Figure 6 A-D**).

In addition, hydrogen-bond interaction analysis, which was previously presented as interaction fractions in **Figure 5C** and **5G**, has been updated to incorporate results from all three replicate simulations, providing a more robust and statistically representative assessment of interaction

persistence.

Together, these analyses demonstrate stable protein-ligand interactions across independent simulations and reveal dominant conformational states used to derive representative binding poses for structural interpretation.

To further characterize ligand binding stability beyond pairwise interaction metrics, we performed clustering analysis of MD trajectories based on ligand heavy-atom conformations (new **Supplementary Figure 6 E-J**). The clustering results reveal that the dominant binding poses are consistently populated across independent simulations, with the top clusters accounting for the majority of sampled conformations, supporting the stability and reproducibility of the ligand binding orientation within the AKR1C3 active site.

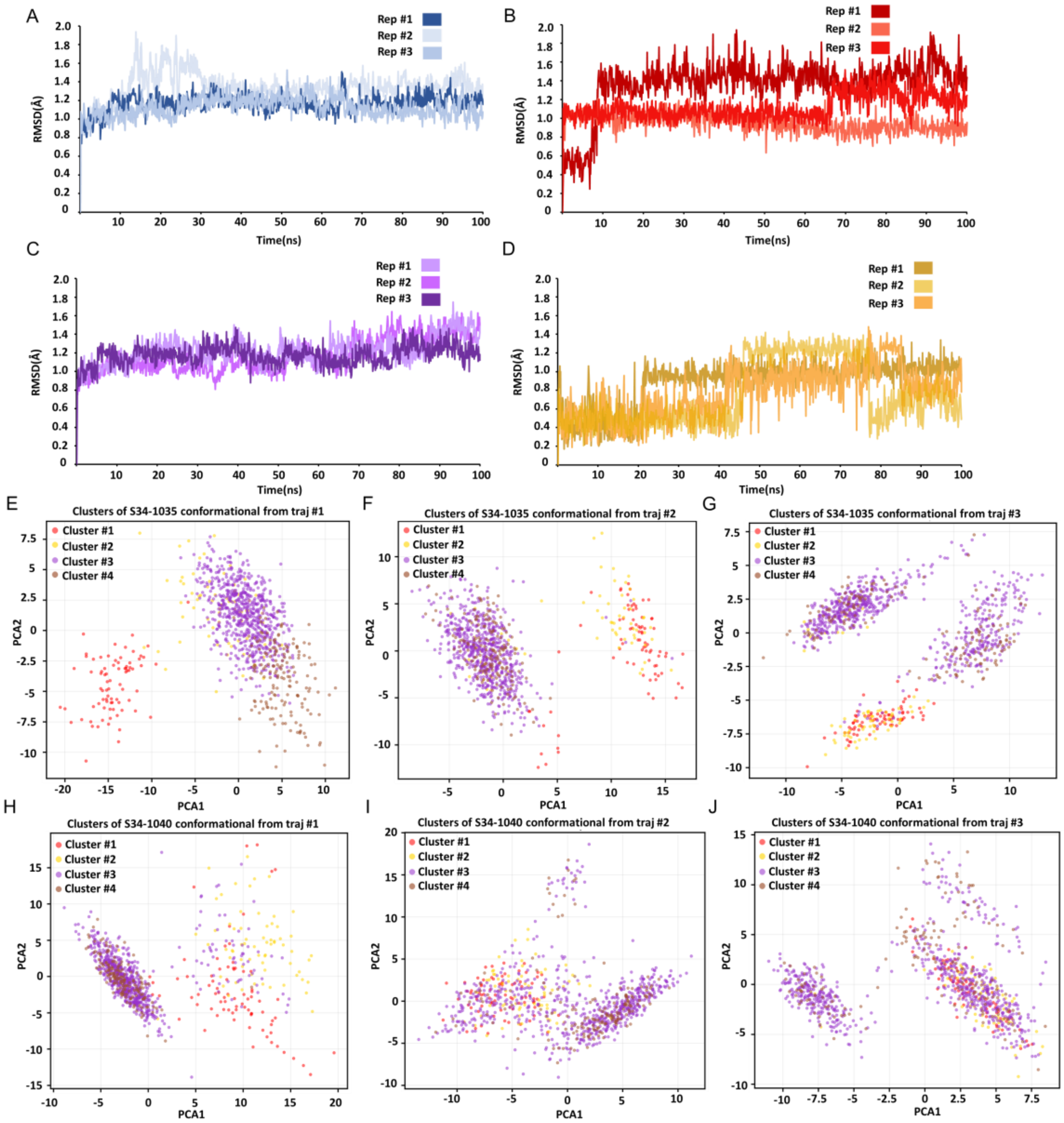

Supplementary Figure 6. Molecular dynamics stability and conformational clustering analysis of S34-1035 and S34-1040 in complex with AKR1C3. A-D. Root-mean-square deviation (RMSD) profiles from three independent 100 ns MD simulations. (**A**) Cα RMSD of AKR1C3 in complex with **S34-1035**. (**B**) Ligand RMSD of **S34-1035** relative to its initial bound

conformation. (**C**) Cα RMSD of AKR1C3 in complex with **S34-1040**. (**D**) Ligand RMSD of **S34-1040** relative to its initial bound conformation. **E-J**. Principal component analysis PCA-based conformational clustering of ligand binding modes. (**E-G**) PCA projections of **S34-1035** conformations from three independent trajectories (traj #1–#3). (**H-J**) PCA projections of **S34-1040** conformations from three independent trajectories (traj #1–#3). Each point represents a simulation frame projected onto the first two principal components (PCA1 and PCA2). Frames are colored according to cluster assignments derived from hierarchical clustering of ligand heavy-atom RMSD matrices: Cluster #1 (red), Cluster #2 (yellow), Cluster #3 (purple), and Cluster #4 (brown).

These additions ensure that the MD analysis is no longer purely descriptive but supported by quantitative and reproducible metrics. The corresponding figures and descriptions have been revised accordingly in the Results section.

Comment #3: Given the high sequence identity among AKR1C isoforms, a brief discussion on selectivity would be valuable. Discussing structural determinants of potential selectivity based on pocket differences would strengthen translational relevance.

Thank you for pointing out the importance of assessing the protein subtype selectivity. In TAME-VS, the target expansion module incorporates homologous proteins to enrich training data, based on the premise that ligands of related targets can provide transferable activity-relevant features for early-stage hit identification. While this strategy improves model robustness under data-limited conditions, it does not explicitly enforce isoform selectivity. In the present study, selectivity was addressed at the design stage through medicinal chemistry considerations informed by structural and mechanistic knowledge of AKR1C3. Accordingly, all 41 candidate compounds were designed with attention to subtype-specific interactions. In this revision, we continued experimental selectivity profiling of prioritized compounds to validate AKR1C3 preference within the AKR1C family.

In the revised manuscript, we present two optimized compounds (**S34-1035** and **S34-1040**) derived from the lead scaffold of **S07-2001**, which exhibit nanomolar potency against AKR1C3 (IC_{50} = 0.04 ± 0.01 μM and 0.03 ± 0.01 μM, respectively) with exceptional selectivity over the other AKR1C isoforms (AKR1C1, AKR1C2, and AKR1C4 all >100 μM), yielding selectivity ratios exceeding 2500-fold (new **Table 3**). This degree of isoform selectivity substantially reduces the likelihood that the observed chemosensitization phenotype arises from off-target inhibition of related AKR1C family members.

Table 3. AKR1C Isoform Inhibitory Activity and Selectivity of Compounds.

Compounds	inhibitory activity, IC_{50} (μM)[a]				IC_{50} ratio		
	1C1	1C2	1C3	1C4	1C1:1C3	1C2:1C3	1C4:1C3
**S34-1035**	>100	>100	0.04 ± 0.01	>100	>2500	>2500	>2500
**S34-1040**	>100	>100	0.03 ± 0.01	>100	>2500	>2500	>2500
**S07-2001**	>100	>100	2.08 ± 1.26	>100	>48.1	>48.1	>48.1

[a]The data are expressed as the mean ± SEM of three independent experiments.

Notably, compared to the lead compound **S07-2001** (AKR1C3 IC_{50} = 2.08 ± 1.26 μM, selectivity ratio >48-fold), the optimized derivatives **S34-1035** and **S34-1040** demonstrate a ~50- to 70-fold improvement in AKR1C3 potency as well as a ~50-fold enhancement in isoform selectivity. This structure-activity progression, combined with the previously reported cellular validation of **S07-2001** in drug-resistant cancer cell lines (*ACS Med. Chem. Lett.* 2022, 13, 1286–1294), provides mechanistic evidence suggesting that the observed chemosensitization effect is preserved and amplified upon structural optimization specifically directed at AKR1C3 engagement. Corresponding changes have been made in the main text Results and Methods sections.

> **Line 373**: *To assess isoform selectivity within the closely related AKR1C family, we evaluated the inhibitory activity of* ***S34-1035*** *and* ***S34-1040*** *against AKR1C1, AKR1C2, and AKR1C4. Both compounds retained nanomolar potency toward AKR1C3 while exhibiting no detectable inhibition of the other AKR1C isoforms (IC_{50} > 100 μM), corresponding to selectivity ratios exceeding 2500-fold (new* ***Table 3****). In comparison, the parent compound* ***S07-2001*** *displayed substantially weaker potency and selectivity (AKR1C3 IC_{50} = 2.08 ± 1.26 μM; selectivity > 48-fold).*
>
> **Line 655**: *The section of Expression and Purification of AKR1Cs.*
>
> **Line 662**: *The section of Enzymatic inhibition assays against recombinant AKR1Cs.*

Comment #4: For statistics in Figure 3C and Figures 5K-N, reporting exact p-values alongside significance thresholds would improve transparency.

Thank you for pointing out that exact p-values alongside significance thresholds should be reported. In the revised manuscript, legends of Figure 3 and Figure 5 were updated to have exact p-values specified.

> **Line 927**: *In C-E, comparison between two groups was performed using an unpaired, two-tailed Student's t-test. For C: RF ns P = 0.1111, **P = 0.0077, ****P < 0.0001; MLP ns P = 0.3014, **P = 0.0020, ****P < 0.0001. For D: RF ns P = 0.1287, ****P < 0.0001; MLP ns P = 0.1439, **P = 0.0092, ****P < 0.0001. For E: RF ns P = 0.4456, ****P < 0.0001; MLP ns P = 0.1656, **P = 0.0050, ****P < 0.0001.*
>
> **Line 965**: *In K-N, comparison between two groups was performed using an unpaired, two-tailed Student's t-test. For K: 10μM **P = 0.0045; 40μM **P = 0.0064; 50μM *P = 0.0158; ****P < 0.0001. For L: 50μM *P = 0.0475, **P = 0.0054, ***P = 0.0007, **P = 0.0012; ****P < 0.0001. For M: 10μM *P = 0.0181; 40μM ***P = 0.0006; 50μM ***P = 0.0004; ****P < 0.0001. For N: 25μM *P = 0.0158, **P = 0.0076; ****P < 0.0001.*

Comment #5: Regarding the web server deployed, would it be possible to have the AKR1C3 included as an example?

Thank you for this valuable suggestion. We have now incorporated AKR1C3 as a dedicated example on our web server. Users can access Example2: P42330 (AKR1C3) directly via the following link: https://aidd.shu.edu.cn/tamevs/example2.

On this page, we have also provided download links (highlighted in blue at the top of the page) for all result files associated with this example, including the complete output generated by our pipeline. These resources are freely accessible and can be downloaded at any time for reference or reproduction purposes. We hope this addition addresses your suggestion and enhances the usability of our web server for the community.

Comment #6: Through assessing the TAME-VS online portal, the reviewer is curious about whether the outcome can be persisted for re-visit later. A minor suggestion is about potentially adding an user login that finished VS runs can be persisted for a certain amount of time to enable the re-visit of the outcome.

Thank you for this thoughtful and valuable suggestion. In the revised manuscript, we have implemented a result persistence mechanism to address this need. Once a submitted job is completed and the result files are generated, a pink "Copy Link" button will appear on the results page (**Figure 6D**). By clicking this button, users can instantly copy a dedicated download link for their result files with a single click. This link remains valid for 7 days, during which users can re-access and download their results at any time, without needing to resubmit the job or visit our website again. Simply pasting the copied link directly into a browser's address bar and pressing Enter will initiate the download.

Comment #7: The reviewer agree that an open source online portal of TAME-VS will benefit a larger community of experimental drug discovery scientists. Currently, a 50K library is the default option. Is there computational resource limitation for large-scale screening? The capacity and the limitation of the computational resources that have been used to support the TAME-VS at the authors' end should be specified.

Thank you for this insightful suggestion. In the revised manuscript, we have expanded the available screening library options on the TAME-VS portal. In addition to the default Enamine Diversity 50K library, we have now included the Enamine Screening 4M library as an additional option, enabling users to perform relatively larger scale virtual screenings directly through our web server. Furthermore, users can upload their own customized compound libraries tailored to their specific research needs through the designed “Screening Set File” button on the submission page. In the future, we plan to continuously expand the variety and coverage of available libraries to better support diverse virtual screening scenarios and to maximize the accessibility and utility of TAME-VS for the broader community.

Regarding the computational resources underpinning our web server, the TAME-VS platform is currently supported by a server equipped with 24 CPU cores, 256 GB RAM, and an NVIDIA RTX A6000 GPU. For the 50K library, a typical screening job can be completed within approximately 10 seconds, while the 4M library requires approximately 12 minutes under standard settings. We specified computational resources in the Methods.

> **Line 735**: *The TAME-VS online server was deployed on a Dell Precision 5860 workstation equipped with an Intel Xeon W7-2495X processor (24 cores, 48 threads, 45 MB cache, base power 225 W, maximum turbo frequency up to 4.8 GHz). The system was configured with 256 GB DDR5 ECC memory (4 × 64 GB RDIMM, 4800 MHz). Storage included a 2*

TB M.2 PCIe NVMe solid-state drive for the operating system and active workloads, complemented by two 8 TB 3.5-inch SATA hard disk drives (7200 RPM) for data archiving. GPU acceleration was provided by an NVIDIA RTX A6000 graphics card with 48 GB GDDR6 memory. The server operated under Ubuntu Linux 22.04 LTS.

Reviewer #4

This manuscript describes an ML-guided virtual screening workflow (TAME-VS) applied to the identification of AKR1C3 inhibitors. The integration of ML-based prioritization with prospective compound synthesis and biochemical/cellular testing is a strength. Inclusion of homolog-derived ligands during model training represents a practical strategy for expanding sparse activity datasets, and the development of an open-source GUI-based pipeline could facilitate broader adoption in early-stage drug discovery. However, several aspects of the modeling assumptions and experimental validation limit the strength of the conclusions. In particular, the biological interpretation of the activity thresholds used for model training, the mechanistic attribution of the observed cellular phenotype to AKR1C3 inhibition, and the presentation and interpretation of the prospective validation require further clarification.

Thank you for the positive and encouraging assessment of our work, as well as for the constructive and thoughtful comments. We have carefully addressed all specific comments, including those related to clarifying activity thresholds used for model training, the link between biochemical inhibition and cellular phenotype, the interpretation of prospective validation, and the analysis of false positive.

Comment #1: Activity thresholds used for model training

A central methodological feature of this study is the use of binary classification labels based on IC_{50} thresholds, where compounds with IC_{50} < 1 μM are defined as active and compounds above 10 μM are inactive. While such thresholds are commonly used in ligand-based modeling, the biological interpretation of a 1 μM cutoff deserves further discussion. At concentrations near 1 μM, most small molecules will interact with multiple cellular targets unless they are highly optimized and selective. As a result, compounds labeled as "active" in the training set may not be acting exclusively through AKR1C3. This raises the possibility that the model may be learning chemical features associated with general enzyme inhibition or promiscuous binding motifs rather than features specific to AKR1C3 engagement. Given that the study ultimately links inhibitor activity to a cellular phenotype (restoration of doxorubicin sensitivity), the authors should more clearly justify the choice of the 1 μM activity threshold.

Thank you for this insightful comment regarding the interpretation of activity thresholds and their potential implications for biological specificity. We agree that an IC_{50} threshold of 1 μM does not imply exclusive target engagement at the cellular level, and that compounds within this potency range may exhibit interactions with multiple targets. In the context of this study, the activity threshold is used as an operational criterion for defining a learnable contrast between active and inactive compounds, rather than as a strict indicator of target-specific

binding. Importantly, the use of a separated inactive threshold (IC_{50} > 10 µM) introduces a potency gap that reduces ambiguity from borderline compounds and improves the robustness of classification.

We further note that the goal of the model at this stage is to capture activity-associated chemical patterns relevant to AKR1C3 engagement within available data, rather than to encode absolute selectivity at the training stage. In practice, subtype selectivity was addressed at the design stage through medicinal chemistry considerations informed by structural and mechanistic knowledge of AKR1C3. Accordingly, all 41 candidate compounds were designed with attention to subtype-specific interactions. In this revision, we further performed experimental selectivity profiling of prioritized compounds, confirming preference for AKR1C3 within the AKR1C family. The part of selectivity will be discussed in more details with your second comment below.

Potential ambiguity arising from heterogeneous activity labels is therefore resolved through downstream validation. In this study, the prioritized compounds were experimentally confirmed to exhibit strong isoform selectivity and demonstrated functional relevance by restoring doxorubicin sensitivity in resistant cancer cells. These results support that the learned patterns are not dominated by general promiscuous inhibition but are associated with biologically meaningful AKR1C3 modulation. To clarify this point, we have added a discussion in the revised manuscript in the Discussion section to explicitly describe the rationale for the activity threshold and its role in early-stage virtual screening.

> **Line 493**: *Activity thresholds should be interpreted as operational definitions that enable model training rather than strict indicators of target exclusivity. While compounds with IC_{50} values in the micromolar range may exhibit off-target interactions, the use of separated active and inactive cutoffs introduces a controlled contrast that supports robust learning. Importantly, downstream selectivity profiling and phenotypic validation are required to establish target-specific biological relevance.*

Comment #2: Link between biochemical inhibition and cellular phenotype

The manuscript reports that two novel compounds restore doxorubicin sensitivity in a resistant breast cancer cell line. While this result is potentially interesting, the experiments presented do not demonstrate that the observed cellular phenotype arises from on-target inhibition of AKR1C3. The current evidence relies on biochemical inhibition of recombinant AKR1C3 combined with cell viability and clonogenic assays. However, these data do not establish that the compounds engage AKR1C3 in cells or that the observed chemosensitization is mediated specifically through this target. Given the modest potency threshold used in the training data and the possibility of off-target effects, additional validation would strengthen the mechanistic conclusions. For example, experiments demonstrating cellular target engagement, genetic perturbation of AKR1C3, or selectivity profiling within the AKR1C enzyme family would help confirm that the phenotype arises from AKR1C3 inhibition rather than alternative mechanisms.

We thank the reviewer for this important and insightful comment. We agree that establishing a mechanistic link between biochemical inhibition of AKR1C3 and the observed cellular phenotype is critical for interpreting the chemosensitization results.

In response, we have performed selectivity profiling of the prioritized compounds within the AKR1C enzyme family. As shown in the revised manuscript, **S34-1035** and **S34-1040** exhibit nanomolar potency against AKR1C3 (IC_{50} = 0.04 ± 0.01 μM and 0.03 ± 0.01 μM, respectively), while showing no detectable inhibition of closely related isoforms AKR1C1, AKR1C2, and AKR1C4 (IC_{50} > 100 μM), corresponding to selectivity ratios exceeding 2500-fold (new **Table 3**).

Table 3. AKR1C isoform inhibitory activity and selectivity of S34-1035, S34-1040, and S07-2001.

Compounds	inhibitory activity, IC_{50} (μM)[a]				IC_{50} ratio		
	1C1	1C2	1C3	1C4	1C1:1C3	1C2:1C3	1C4:1C3
**S34-1035**	>100	>100	0.04 ± 0.01	>100	>2500	>2500	>2500
**S34-1040**	>100	>100	0.03 ± 0.01	>100	>2500	>2500	>2500
**S07-2001**	>100	>100	2.08 ± 1.26	>100	>48.1	>48.1	>48.1

[a]The data are expressed as the mean ± SEM of three independent experiments.

This high degree of isoform selectivity substantially reduces the likelihood that the observed cellular phenotype arises from inhibition of other AKR1C family members. In addition, the optimized compounds show marked improvements in both potency and selectivity relative to the parent scaffold **S07-2001**, which has previously been validated in drug-resistant cancer cell models. The consistent structure–activity progression observed across this series supports a target-driven optimization process centered on AKR1C3 engagement.

We agree that additional approaches, such as direct cellular target engagement assays or genetic perturbation of AKR1C3, would further strengthen mechanistic attribution. While these experiments are beyond the scope of the current study, we have revised the Discussion section to explicitly acknowledge this limitation and to clarify that the current results provide strong supporting evidence, rather than definitive proof, that the observed chemosensitization is mediated through AKR1C3 inhibition. We revised the Results and Discussion to reflect the selectivity profiling we conducted.

> **Line 373**: *To assess isoform selectivity within the closely related AKR1C family, we evaluated the inhibitory activity of **S34-1035** and **S34-1040** against AKR1C1, AKR1C2, and AKR1C4. Both compounds retained nanomolar potency toward AKR1C3 while exhibiting no detectable inhibition of the other AKR1C isoforms (IC_{50} > 100 μM), corresponding to selectivity ratios exceeding 2500-fold (new **Table 3**). In comparison, the parent compound **S07-2001** displayed substantially weaker potency and selectivity (AKR1C3 IC_{50} = 2.08 ± 1.26 μM; selectivity > 48-fold).*

> **Line 520**: *While the biochemical potency and isoform selectivity strongly support AKR1C3 engagement, we note that direct cellular target engagement or genetic perturbation studies would be required to establish definitive causality between enzyme inhibition and the observed phenotypic effects.*

Comment #3: Interpretation of the prospective validation

Because the full set of 41 compounds was ultimately synthesized and shows a high baseline hit rate (28/41 active), the design space already appears strongly enriched for active molecules. Under these conditions, the practical impact of model-based prioritization is difficult to interpret, as most compounds within the design set are already active.

We thank the reviewer for this thoughtful comment regarding the interpretation of prospective validation in the context of a high baseline activity rate. The designed chemical space is already enriched for active compounds (28/41, 68.3%), reflecting effective medicinal chemistry design. Under such conditions, the role of model-based prioritization is not to identify actives from a largely inactive background, but rather to preferentially rank active compounds earlier within a constrained and feasible synthesis space.

The experimental design of this study enables an unbiased assessment of this effect, as all 41 designed compounds were synthesized and evaluated. This allows direct comparison between the intrinsic activity distribution of the design space and the subset prioritized by the model. Within this context, the observed increase from 68.3% to 92.8% reflects enrichment through prioritization rather than discovery, and therefore should be interpreted as an improvement in decision-making efficiency under synthesis constraints.

Comment #4: Analysis of false positives

The reported 92.8% hit rate is based on only 14 prioritized compounds and should therefore be interpreted cautiously. Given the small sample size and the already high baseline activity rate across the full design set (28/41, 68.3%), it is unclear whether the observed enrichment reflects a robust advantage of model-based prioritization or simply ranking within an already highly enriched chemical series.

We thank the reviewer for this important comment regarding the interpretation of hit rate and the potential impact of sample size and baseline activity. We agree that the number of prioritized compounds (14) is relatively small and that the baseline activity rate within the designed chemical space (28/41, 68.3%) is already high, reflecting effective medicinal chemistry design. In this context, the reported hit rate of 92.8% should not be interpreted as an absolute measure of model performance, but rather as an indication of enrichment within a constrained and already active chemical series.

Importantly, the design of this study enables a direct and unbiased comparison between model-guided prioritization and the intrinsic activity distribution of the full compound set, as all 41 designed compounds were synthesized and experimentally evaluated. This allows us to assess whether the model preferentially ranks active compounds earlier within a feasible synthesis space, which more closely reflects real-world decision-making in early-stage discovery.

We have revised the manuscript:

> **Line 316***: In this context, the observed increase to 92.8% reflects preferential ranking of active compounds within an already enriched chemical space, rather than discovery from a predominantly inactive background.*

To clarify this point and to present the results in terms of enrichment over a rational baseline, rather than emphasizing the absolute hit rate. We also note that the prioritization of the most

potent compounds, including **S34-1035** and **S34-1040**, further supports the practical utility of the model in guiding experimental resource allocation.

Detailed response to the reviewers

We thank the reviewers for their thorough and constructive re-evaluation of the revised manuscript. We are pleased that the revisions have been well received, and we are grateful for the continued engagement and valuable feedback that has helped strengthen the work. We have addressed the remaining comments below and provide a further revised manuscript accordingly.

Reviewer #1

Comment #1: The authors have substantially revised the manuscript in response to the reviewers' comments. In particular, the revisions directly and convincingly address the major conceptual and framing concerns had raised previously regarding claims of novelty, scaffold discovery, and the interpretation of "prospective" validation. The current version is significantly improved in clarity, statistical rigor, and positioning relative to prior work, and it now presents a balanced, accurate account of the contribution. In its revised form, the manuscript no longer overstates chemical or scaffold novelty and instead focuses appropriately on model-guided prioritization within a medicinal chemistry–defined design space, coupled with an experimentally closed-loop validation. The level of experimental follow-up, transparency around human-driven decisions, and rigor of the statistical analyses are now appropriate for publication in Communications Chemistry.

Thank you for the thorough and generous re-evaluation of our revised manuscript. We are pleased that the revisions have effectively addressed the earlier conceptual and framing concerns, and we appreciate the reviewer's recognition of the improvements in clarity, statistical rigor, and positioning.

Reviewer #2

Comment #1: The revised manuscript represents a substantial improvement over the original submission. The authors have clearly addressed earlier concerns regarding novelty and scope, particularly by removing any implication of scaffold discovery and reframing the work around model-guided prioritization within a defined medicinal-chemistry space. The inclusion of relevant reference compounds and closely related AKR1C3 studies provides appropriate context for the experimental results, and the expanded discussion of evaluation metrics in the main text moves the analysis beyond recall-centric claims. In addition, the explicit description of human-driven scaffold and substituent selection is a welcome change, as it improves transparency and avoids overstating the role of the machine-learning models in the overall decision-making process.

Thank you for the careful and detailed re-evaluation of our revised manuscript. We appreciate the reviewer's thorough engagement and constructive assessment throughout the review process.

Comment #2: One remaining conceptual point concerns how best to benchmark and interpret the contribution of the machine-learning models themselves. While the prospective results clearly demonstrate enrichment through prioritization, it remains challenging to assess to what extent the models are learning target-specific structure–activity features that are distinct from the medicinal-chemistry bias introduced by the human-defined design space. The analogs explored are, as expected, constructed from components related to the training data, which complicates claims that the models are generating newly created decision-making "equity" rather than reinforcing expert priors. This does not invalidate the results, but it does suggest that the primary value demonstrated here is effective ranking within a constrained, expert-designed chemical space rather than emergent chemical novelty.

Thank you for this insightful point. We agree with the reviewer's assessment. Within the current study design, it is inherently difficult to decouple target-specific structure–activity relationship learning from the medicinal-chemistry bias introduced by the human-defined design space, and we acknowledge that the analogs explored were constructed from scaffolds and substituents selected through expert-guided reasoning closely related to the training data. As the reviewer notes, this does not invalidate the prospective enrichment results, but it does mean that the primary value demonstrated here is effective compound prioritization within a constrained, expert-designed chemical space rather than the emergence of structural novelty beyond expert priors. We have revised the Discussion section to make this distinction more explicit. Specifically, we rephrased following at:

> ***Line 505:*** *However, the added enrichment from TAME-VS prioritization demonstrates a complementary function that given an expert-designed chemical space, the model helps decide which compounds to make first, thereby increasing experimental efficiency.*

Comment #3: As an additional suggestion, the authors may wish to frame this limitation more explicitly and note directions for future work. For example, further analysis of which functional groups or physicochemical features drive model prioritization, or evaluation of model behavior in less human-biased or partially model-generated chemical spaces, could provide deeper insight into whether true scaffold hopping or structural novelty emerges when design constraints are relaxed. Such discussion would strengthen the conceptual clarity of the study without changing its scope. With these considerations acknowledged, I believe the revised manuscript presents a clear, balanced, and experimentally well-supported contribution and is suitable for acceptance in its current revised form.

Thank you for the constructive suggestion. We agree that further analysis of functional groups and physicochemical features that drive the model prioritization is of great value as a future direction to note. Related discussion is inserted at:

> ***Line 535:*** *Fourth, while the prospective results demonstrate clear enrichment through model-guided prioritization, future studies should examine which functional groups or physicochemical features most strongly drive model decisions, and evaluate model behavior in less expert-biased or partially model-generated chemical spaces. Such*

analyses would provide deeper insight into whether structural novelty or scaffold hopping emerges when design constraints are relaxed, and would more directly assess the extent to which the models capture target-specific structure–activity features beyond reinforcement of expert priors.

Reviewer #3

Comment #1: The authors have revised the manuscript accordingly to address comments and concerns raised in the initial review particularly on reproduceable MD simulations and expanded experimental validation of isoform selectivity. The reviewer has no additional comments to add. The responses are thorough and the manuscript is suitable for publication in its current form.

Thank you for the careful re-evaluation of our revised manuscript and for confirming that the concerns regarding reproducibility of MD simulations and expanded isoform selectivity validation have been satisfactorily addressed. We are grateful for the reviewer's time, expertise, and constructive engagement throughout the review process.

Reviewer #4

Comment #1: The authors have addressed major comments raised in the initial review. The revised manuscript provides additional clarification regarding the model activity thresholds, the interpretation of the validation and its limitations. Additional discussion of false positives and the statistical interpretation of the reported enrichment strengthens the manuscript. Some mechanistic questions regarding on-target cellular activity and selectivity remain for future study, but the authors have adjusted claims and clarified scope. The revision presents a useful workflow for virtual screening with prospective experimental validation and will be of interest to drug discovery folks. Thus, I think the manuscript is suitable for publication in Communications Chemistry.

Thank you for the positive and encouraging assessment of our work, as well as for the constructive and thoughtful comments throughout the review process. We are pleased that the additional clarification on activity thresholds, validation interpretation, false positive analysis, and statistical enrichment has strengthened the manuscript to the reviewer's satisfaction.

Review: Target-driven machine learning–enabled virtual screening (TAME-VS) identifies novel AKR1C3 inhibitors

Recommendation: Reject.

The authors describe a set of machine-learning models integrated into a previously published, publicly accessible web platform for exploring analogs of AKR1C inhibitors. Their approach relies on traditional ML methods trained on small-molecule activity data across AKR1C isoforms, similar to strategies reported in recent literature. They manually curate two potent scaffold families and define a synthetically accessible set of substituents, which are then used to illustrate the platform's capabilities. Model performance is demonstrated primarily through recall-based evaluation. A small number of predicted compounds are synthesized and tested in biological assays, and the authors propose these as potential leads. Overall, the work represents a conventional virtual-screening workflow paired with limited experimental validation.

The manuscript does not meet the methodological or conceptual standards required for publication in Nature Communications. The machine-learning components lack sufficient benchmarking, quantitative evaluation, and comparison against established baselines, making it difficult to assess the robustness or generalizability of the proposed approach. In addition, the work does not adequately address potency or efficacy benchmarks that are standard in this research area. Finally, the methodological contribution is not sufficiently novel or differentiated from existing literature to justify publication in a high-impact journal.

Suggested Revisions Prior to Submission to Other Journals

- **Include and discuss closely related prior work.**
  The manuscript should cite and compare its methodology and results to the closely related study ACS Med. Chem. Lett. (DOI: 10.1021/acsmedchemlett.4c00150). This earlier work employs a similar machine-learning strategy for AKR1C3 inhibitor discovery, and a direct comparison would help clarify what is novel in the present study and how its performance differs.
- **Strengthen experimental benchmarking with established reference compounds.**
  The biological validation would be more impactful if benchmark AKR1C3 inhibitors were included alongside the newly synthesized compounds.
  - What are the strongest published AKR1C3 inhibitors, and how do the authors' compounds perform relative to them in the same assays?
  - What specific limitations of existing inhibitors motivate the synthesis of new analogs in this chemical space?
    Providing this context would help justify the experimental direction and clarify the contribution of the new compounds.
- **Clarify human-driven decisions in the machine-learning workflow.**

The manuscript should more fully describe any manual steps in the modeling pipeline, particularly the rationale for selecting the two scaffold families and the chosen substituent set. Since these decisions shape the model's chemical search space, transparency is essential for reproducibility and for evaluating the generality of the approach.

- **Revise and expand machine-learning performance metrics.**
  The current reliance on recall alone is insufficient for evaluating model quality or for supporting compound triage from large chemical libraries. Recall can be trivially maximized by predicting every compound as a hit.
  Metrics such as F1 score, Matthews correlation coefficient (MCC), precision, and ROC-AUC are standard in cheminformatics and should be included to provide a balanced assessment of model performance. These additions would bring the evaluation in line with industry and academic best practices.
- **Reconsider the article structure for the intended journal.**
  As written, the manuscript does not align with the expectations for Nature Communications, which typically requires a substantial conceptual advance, methodological innovation, or broad scientific impact. The current work is more consistent with a traditional virtual-screening study supported by limited experimental follow-up.
