# Supplementary material for: Target-driven machine learning–enabled virtual screening (TAME-VS) enables prioritization of AKR1C3 inhibitors
Source: Commun Chem. 2026 Jun 15;9:311. doi: 10.1038/s42004-026-02092-6 (PMC13631243; doi:10.1038/s42004-026-02092-6)
Supplement: Supplementary file 3 [file 42004_2026_2092_MOESM2_ESM.pdf]

Supporting Information:

Target-driven machine learning–enabled virtual screening (TAME-VS) enables prioritization of AKR1C3 inhibitors

Longjiang Qiao[1,2,†], Xinyu Li[3,†], Shuaishuai Xing[3], Qianwen Guan[3], Kun Ning[1,2], Weijie Han[1,2], Luyu Zhang[1], Anlan Dai[1], Weiqi Yang[1], Yinhang Liu[4,5], Jason J. Kwon[6], Xin Gao[1,7]*, Haopeng Sun[3,8]*, Yuemin Bian[1,2,9]*

[1]Digital Medical Research Institute, School of Medicine, Shanghai University, Shanghai, China

[2]Department of Pharmaceutical Science, School of Medicine, Shanghai University, Shanghai, China

[3]School of Pharmacy, China Pharmaceutical University, Nanjing, China

[4]Department of Bioengineering, University of California, Berkeley, Berkeley, CA, USA

[5]Department of Bioengineering and Therapeutic Sciences, University of California, San Francisco, San Francisco, CA, USA

[6]Cancer Program, Broad Institute of MIT and Harvard, Cambridge, MA, USA

[7]Shanghai Universal Medical Diagnostic Imaging Center, Shanghai, China

[8]State Key Laboratory of Natural Medicines, China Pharmaceutical University, Nanjing, China

[9]Shanghai Engineering Research Center of Organ Repair, Joint International Research Laboratory of Biomaterials and Biotechnology in Organ Repair (Ministry of Education), Shanghai, China

[†]These authors contributed equally to this work.

*To whom correspondence should be addressed: Xin Gao (george.ssmu@163.com), Haopeng Sun (sunhaopeng@cpu.edu.cn), Yuemin Bian (yuemin@shu.edu.cn).

Table of Contents

Supplementary Tables

1. Example compounds obtained for each target with different types of biological activities……………………………………………………………………………………………3
2. Number of compounds obtained for each target with different types of biological tests……………………………………………………………………………………………4
3. Performance metrics of RF classifiers across three training strategies and fingerprint representations at the optimal activity cutoff……………………………………………...5
4. Performance metrics of MLP classifiers across three training strategies and fingerprint representations at the optimal activity cutoff…………………………………6
5. RF and MLP prediction scores across three training strategies of 28 compounds from JMC 2025……………………………………………………………………………………7
6. RF and MLP prediction scores across three training strategies of 30 compounds from EJMC 2025…………………………………………………………………………………..8
7. Prediction scores and ranking of newly designed scaffold #1 compounds by RF and MLP models before and after model retraining under strategies #2 and #3…………..9
8. Prediction scores and ranking of newly designed scaffold #2 compounds by RF and MLP models before and after model retraining under strategies #2 and #3………...10
9. Representative reported AKR1C3 inhibitors, their biochemical activities, and key limitations relevant to inhibitor development…………………...……………...……….11

Supplementary Figures

1. Performance evaluation of TAME-VS models under training strategies #1 and #3…13
2. Structural basis and physicochemical diversity of retrospective validation compounds……………………………………………………………………………………14
3. Similarities between the actives in Strategy #2 and retrospective validation compounds……………………………………………………………………………………16
4. Dependence of hit rates on activity cutoffs before and after model retraining………17
5. Synthetic Routes to Scaffold #1 and Scaffold #2 Analogues…………………………18
6. Molecular dynamics stability and conformational clustering analysis of **S34-1035** and **S34-1040** in complex with AKR1C3……………………………………………………..20

Supplementary Methods

1. Synthesis of Scaffold #1 analogues…………………………………………………………22
2. Synthesis of Scaffold #2 analogues…………………………………………………………28

Supplementary Tables

Supplementary Table 1. Example compounds obtained for each target with different types of biological activities.

Assay type	AKR1B1	AKR1C3	AKR1C2	AKR1C1	AKR1B10	AKR1C4	AKR1A1
IC_{50}	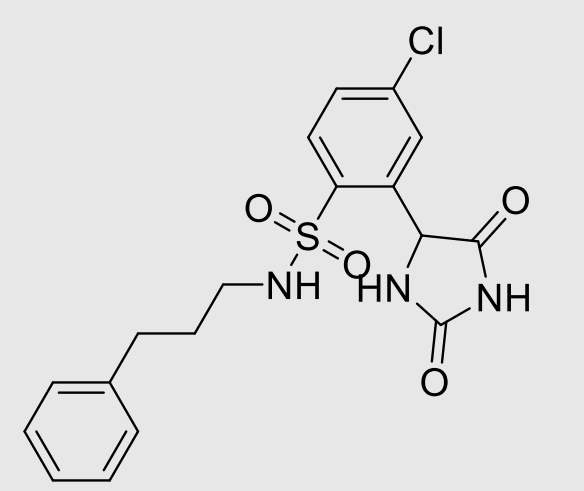<br>IC_{50} = 0.1 μM<br>1169 molecules with IC_{50}	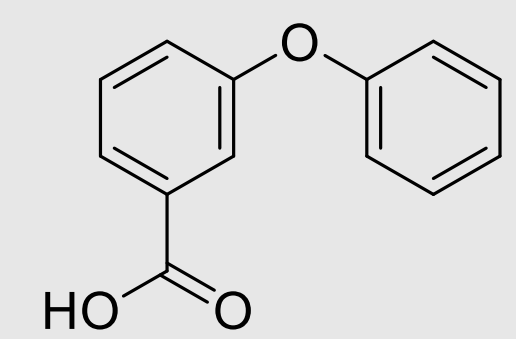<br>IC_{50} = 0.68 μM<br>743 molecules with IC_{50}	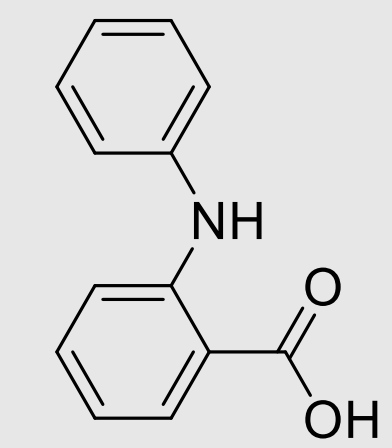<br>IC_{50} = 0.44 μM<br>628 molecules with IC_{50}	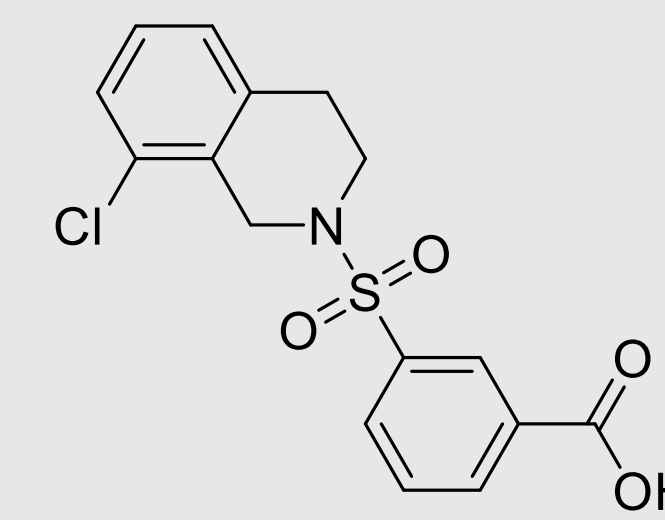<br>IC_{50} = 0.74 μM<br>340 molecules with IC_{50}	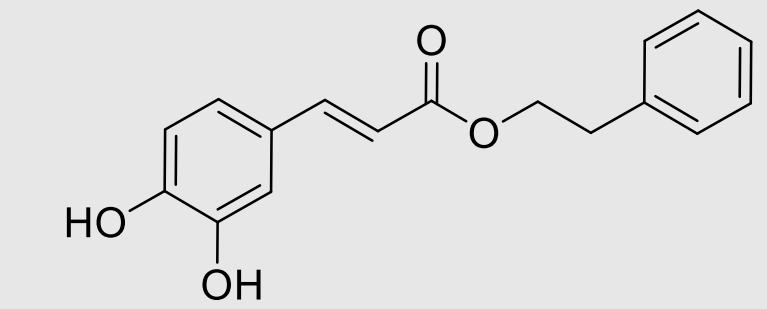<br>IC_{50} = 0.08 μM<br>170 molecules with IC_{50}	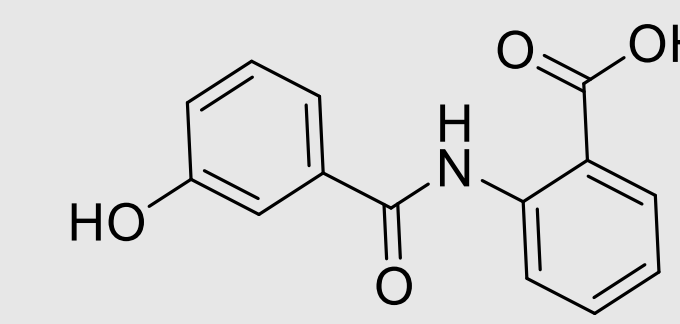<br>IC_{50} = 100 μM<br>289 molecules with IC_{50}	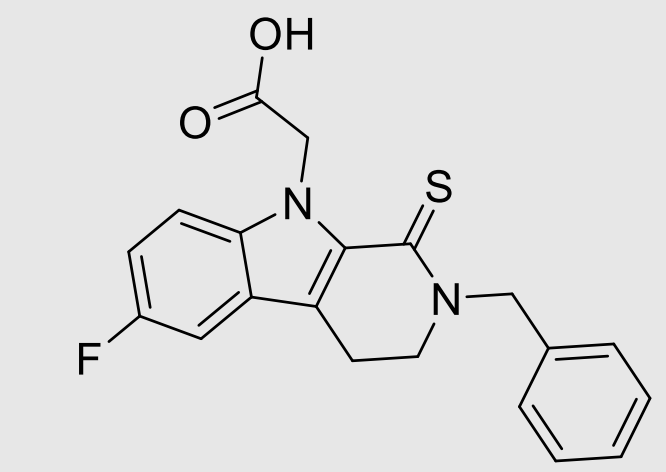<br>IC_{50} = 19 μM<br>102 molecules with IC_{50}
% Inh	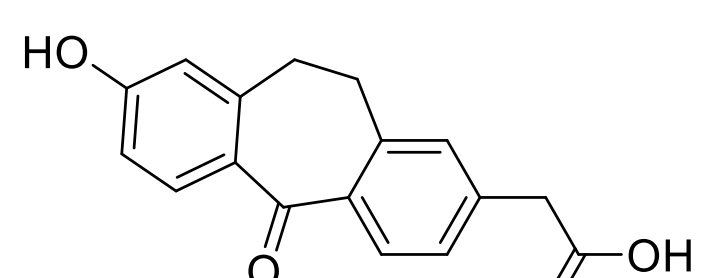<br>% Inh = 96<br>197 molecules with % Inh	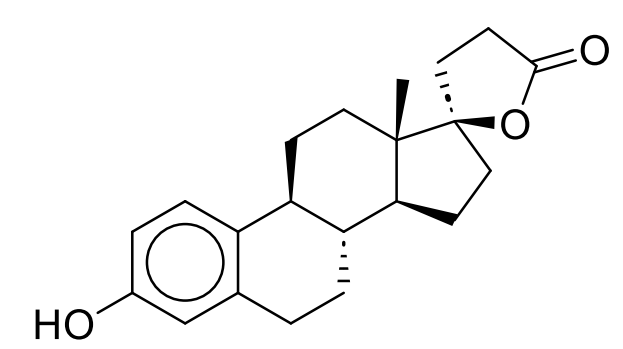<br>% Inh = 47<br>172 molecules with % Inh	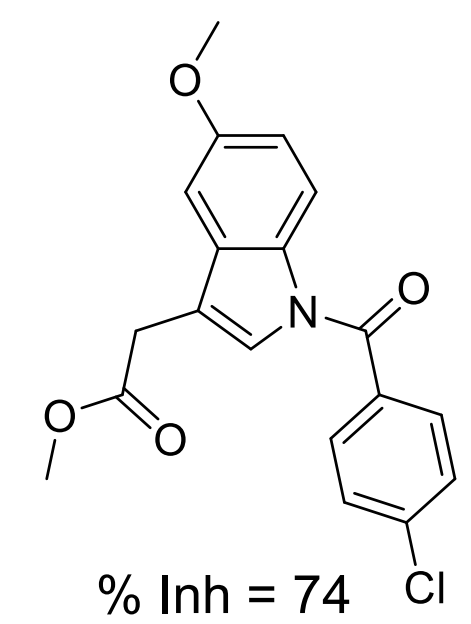<br>% Inh = 74<br>109 molecules with % Inh	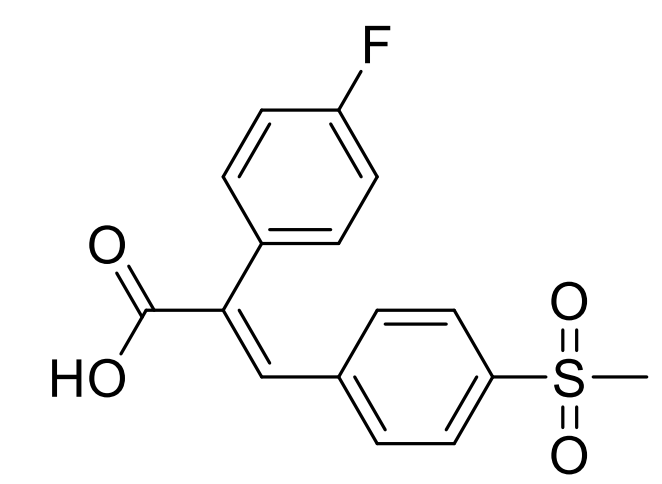<br>% Inh = 86.1<br>114 molecules with % Inh	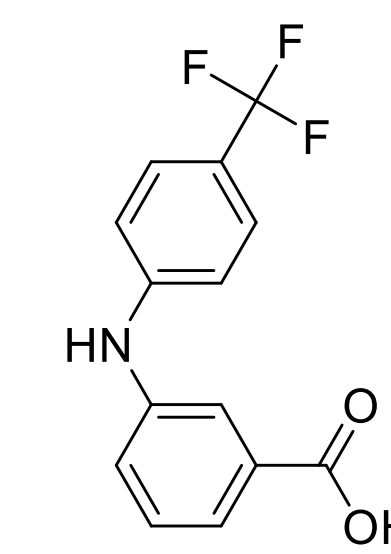<br>% Inh = 40<br>50 molecules with % Inh	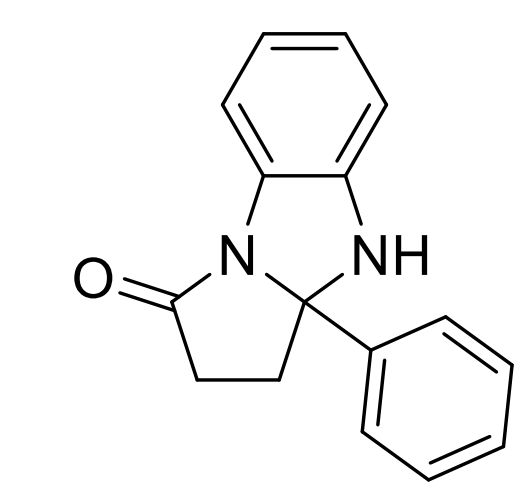<br>% Inh = 48<br>8 molecules with % Inh	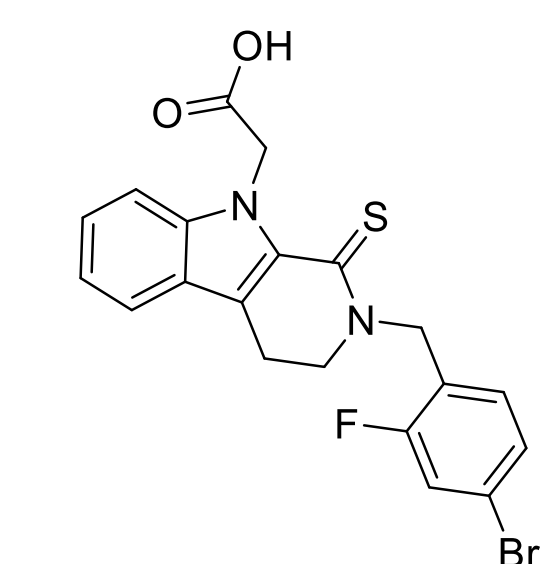<br>% Inh = 45<br>12 molecules with % Inh
K_i	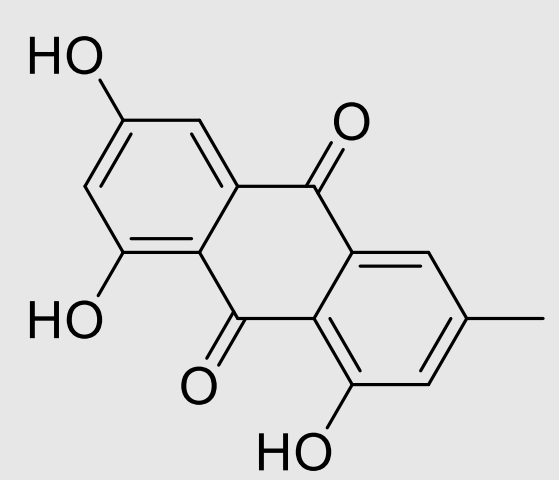<br>K_i = 2.113 μM<br>36 molecules with K_i	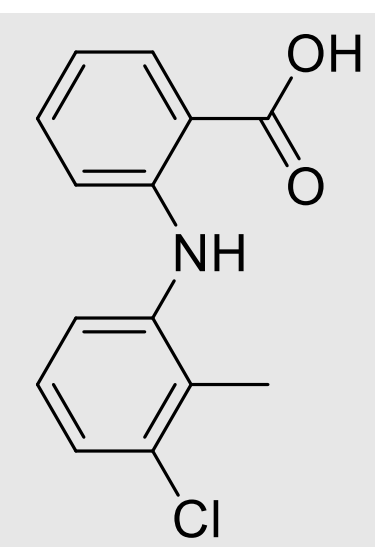<br>K_i = 8 nM<br>52 molecules with K_i	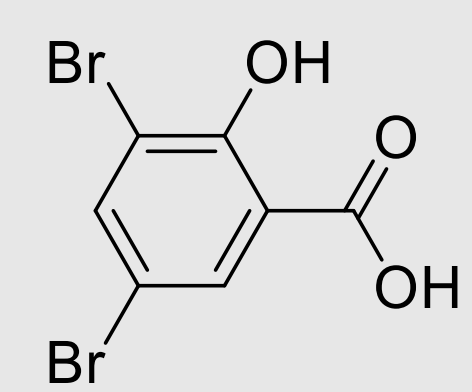<br>K_i = 0.082 μM<br>35 molecules with K_i	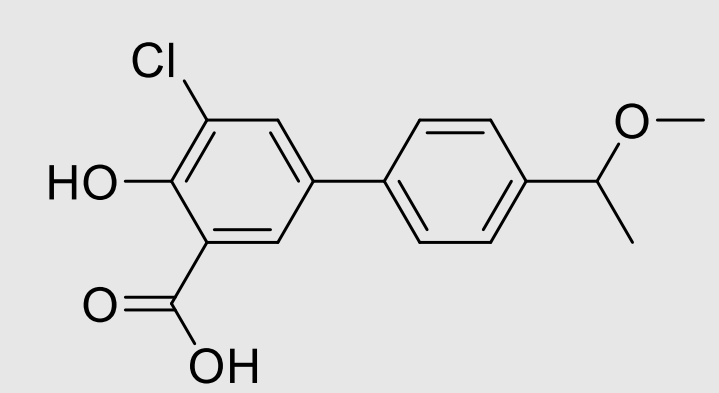<br>K_i = 0.34 μM<br>59 molecules with K_i	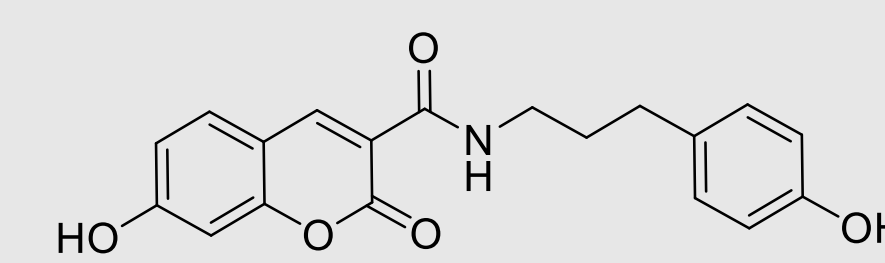<br>K_i = 2.3 nM<br>30 molecules with K_i	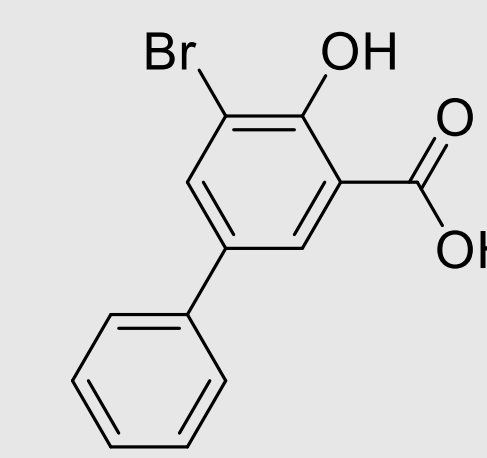<br>K_i = 18.2 μM<br>5 molecules with K_i	0 molecules with K_i
Others	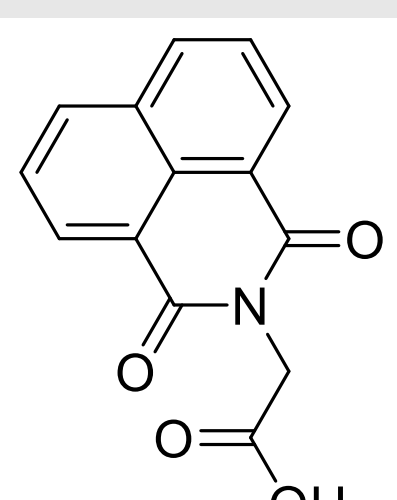<br>ED_{50} = 0.000001 mg/kg<br>70 molecules with other types	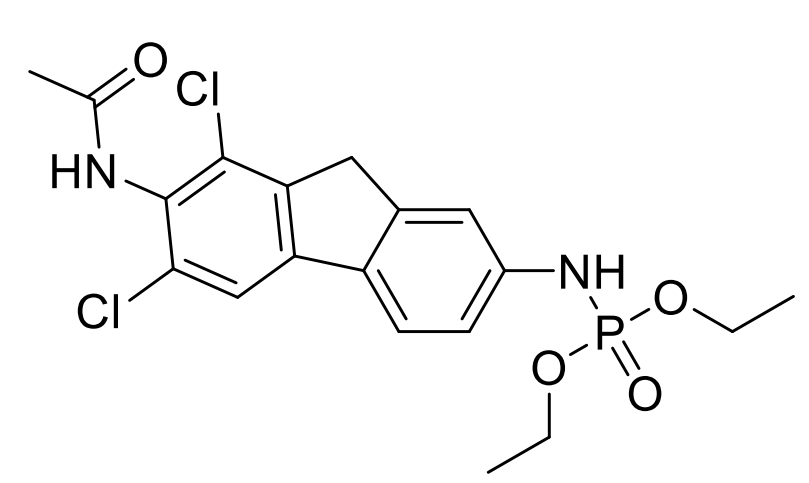<br>Activity = 84%<br>33 molecules with other types	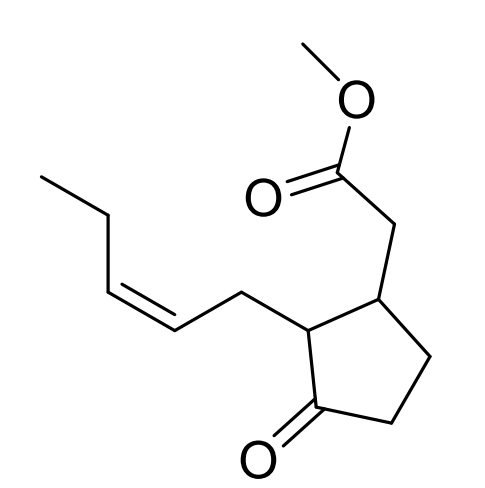<br>Kis = 49 μM/L<br>6 molecules with other types	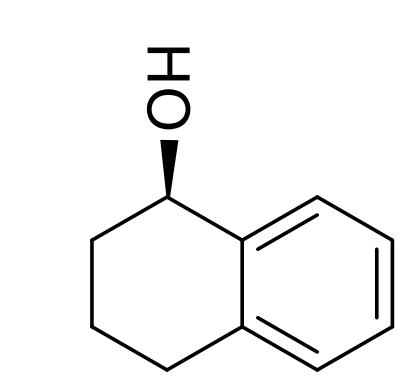<br>K_{cat} / k_m = 0.05 /μM/min<br>42 molecules with other types	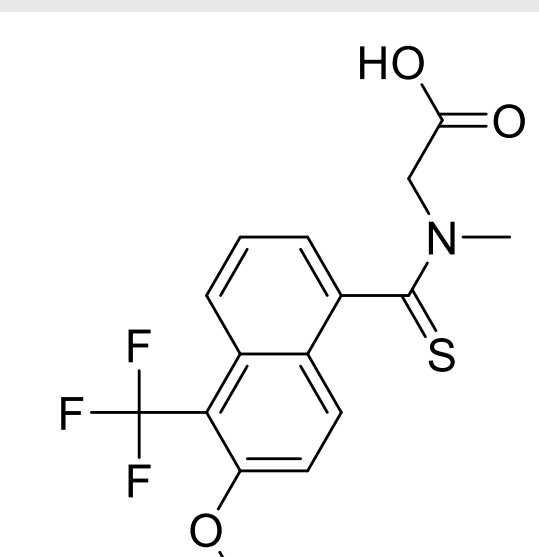<br>Activity = 50%<br>58 molecules with other types	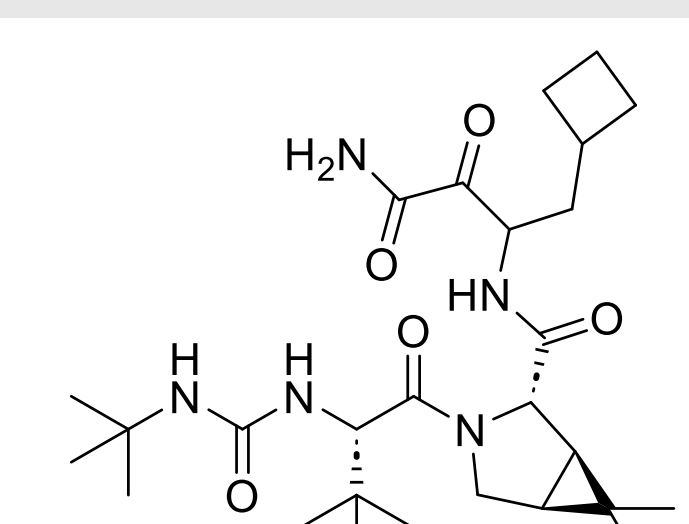<br>Compound recovery<br>1 molecules with other types	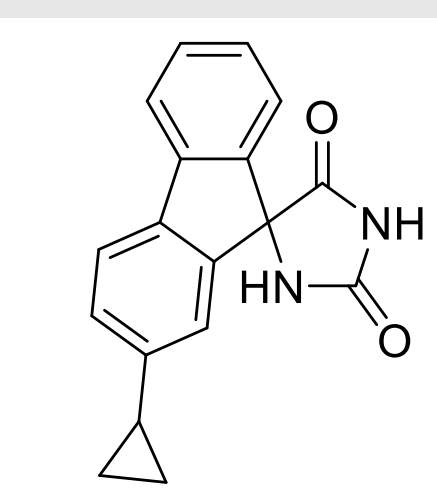<br>V_{max} = 13 μmol/min<br>12 molecules with other types

Supplementary Table 2. Number of compounds obtained for each target with different types of biological tests.

Bioassay types	AKR1B1	AKR1C3	AKR1C2	AKR1C1	AKR1B10	AKR1C4	AKR1A1
IC_{50}	1169	743	628	340	170	289	102
% Inh	197	172	109	114	50	8	12
K_i	36	52	35	59	30	5	0
Km	14	1	1	7	12	0	3
Ratio IC_{50}	12	16	0	0	3	0	0
k cat/Km	7	0	0	0	0	0	0
Activity	7	14	0	7	1	0	2
k cat	6	0	0	0	0	0	0
Kcat	5	0	0	0	16	0	1
K	4	0	0	0	0	0	0
Ratio Ki	3	0	0	12	7	0	0
Kcat/Km	3	0	0	7	12	0	0
Kis	2	0	1	1	0	0	0
Kii	2	0	0	0	0	0	0
Vmax	2	1	1	0	0	0	2
FC	1	0	1	0	4	0	0
Drug metabolism	1	0	0	0	0	0	0
ED_{50}	1	0	0	0	0	0	0
Compound recovery	0	0	1	0	0	1	0
Vmax/Km	0	1	1	0	0	0	1
Ratio	0	0	0	8	0	0	0
Selectivity ratio	0	0	0	0	3	0	0
Tm	0	0	0	0	0	0	3

Supplementary Table 3. Performance metrics of RF classifiers across three training strategies and fingerprint representations at the optimal activity cutoff.

RF	Type of FPs	AUC	Precision	Recall	Specificity	F1 score	Geometric mean	Index of balanced accuracy
Strategy #1	Morgan	0.924 ± 0.028	0.873 ± 0.048	0.838 ± 0.065	0.876 ± 0.055	0.842 ± 0.063	0.852 ± 0.056	0.726 ± 0.098
	Atompair	0.914 ± 0.044	0.882 ± 0.042	0.851 ± 0.056	0.887 ± 0.048	0.855 ± 0.054	0.865 ± 0.047	0.747 ± 0.083
	Topological	0.921 ± 0.035	0.877 ± 0.041	0.848 ± 0.050	0.880 ± 0.045	0.851 ± 0.048	0.861 ± 0.043	0.740 ± 0.076
	MACCS	0.926 ± 0.020	0.877 ± 0.038	0.854 ± 0.053	0.868 ± 0.042	0.857 ± 0.051	0.857 ± 0.041	0.736 ± 0.073
Strategy #2	Morgan	0.945 ± 0.025	0.906 ± 0.019	0.884 ± 0.024	0.901 ± 0.035	0.889 ± 0.022	0.892 ± 0.026	0.795 ± 0.045
	Atompair	0.934 ± 0.023	0.888 ± 0.024	0.862 ± 0.025	0.870 ± 0.059	0.869 ± 0.024	0.865 ± 0.038	0.749 ± 0.063
	Topological	0.942 ± 0.018	0.898 ± 0.014	0.873 ± 0.025	0.887 ± 0.033	0.879 ± 0.022	0.879 ± 0.021	0.772 ± 0.036
	MACCS	0.926 ± 0.027	0.882 ± 0.023	0.856 ± 0.025	0.854 ± 0.061	0.862 ± 0.023	0.853 ± 0.038	0.730 ± 0.062
Strategy #3	Morgan	0.966 ± 0.015	0.919 ± 0.038	0.916 ± 0.040	0.913 ± 0.039	0.916 ± 0.039	0.913 ± 0.039	0.836 ± 0.071
	Atompair	0.965 ± 0.014	0.918 ± 0.022	0.916 ± 0.022	0.909 ± 0.025	0.916 ± 0.022	0.911 ± 0.024	0.832 ± 0.042
	Topological	0.967 ± 0.016	0.926 ± 0.024	0.923 ± 0.025	0.925 ± 0.025	0.923 ± 0.024	0.923 ± 0.024	0.852 ± 0.045
	MACCS	0.965 ± 0.010	0.904 ± 0.018	0.902 ± 0.019	0.897 ± 0.022	0.902 ± 0.019	0.898 ± 0.020	0.808 ± 0.035

Supplementary Table 4. Performance metrics of MLP classifiers across three training strategies and fingerprint representations at the optimal activity cutoff.

MLP	Type of FPs	AUC	Precision	Recall	Specificity	F1 score	Geometric mean	Index of balanced accuracy
Strategy #1	Morgan	0.913 ± 0.031	0.859 ± 0.035	0.835 ± 0.052	0.848 ± 0.024	0.838 ± 0.049	0.839 ± 0.035	0.704 ± 0.060
	Atompair	0.885 ± 0.048	0.841 ± 0.049	0.815 ± 0.063	0.829 ± 0.063	0.819 ± 0.061	0.818 ± 0.060	0.673 ± 0.099
	Topological	0.891 ± 0.032	0.863 ± 0.044	0.838 ± 0.049	0.865 ± 0.059	0.842 ± 0.047	0.849 ± 0.050	0.721 ± 0.083
	MACCS	0.895 ± 0.033	0.860 ± 0.053	0.848 ± 0.062	0.839 ± 0.055	0.850 ± 0.060	0.842 ± 0.057	0.713 ± 0.098
Strategy #2	Morgan	0.928 ± 0.029	0.886 ± 0.025	0.866 ± 0.024	0.851 ± 0.070	0.871 ± 0.024	0.856 ± 0.046	0.736 ± 0.071
	Atompair	0.914 ± 0.026	0.881 ± 0.023	0.853 ± 0.031	0.855 ± 0.049	0.860 ± 0.028	0.854 ± 0.036	0.730 ± 0.059
	Topological	0.929 ± 0.019	0.890 ± 0.012	0.864 ± 0.023	0.870 ± 0.040	0.870 ± 0.020	0.866 ± 0.021	0.750 ± 0.034
	MACCS	0.903 ± 0.036	0.865 ± 0.028	0.839 ± 0.035	0.823 ± 0.051	0.846 ± 0.032	0.830 ± 0.041	0.692 ± 0.068
Strategy #3	Morgan	0.955 ± 0.023	0.899 ± 0.029	0.897 ± 0.028	0.891 ± 0.033	0.897 ± 0.028	0.893 ± 0.030	0.799 ± 0.053
	Atompair	0.960 ± 0.019	0.900 ± 0.030	0.897 ± 0.032	0.889 ± 0.032	0.896 ± 0.032	0.891 ± 0.032	0.796 ± 0.057
	Topological	0.959 ± 0.017	0.902 ± 0.016	0.900 ± 0.017	0.895 ± 0.019	0.900 ± 0.017	0.897 ± 0.018	0.805 ± 0.031
	MACCS	0.944 ± 0.024	0.898 ± 0.031	0.895 ± 0.032	0.891 ± 0.030	0.895 ± 0.032	0.892 ± 0.030	0.797 ± 0.054

Supplementary Table 5. RF and MLP prediction scores across three training strategies of 28 compounds from JMC 2025.

JMC, 2025, 7369-7389	AKR1C3 IC_{50} (μM)	Strategy #1				Strategy #2				Strategy #3			
		RF prediction score	RF rank	MLP prediction score	MLP rank	RF prediction score	RF rank	MLP prediction score	MLP rank	RF prediction score	RF rank	MLP prediction score	MLP rank
Cmpd 1	0.16 ± 0.04	0.68430	17	0.999902502	7	0.84895	4	0.999865706	11	0.69859	2	0.995974025	10
Cmpd 2	0.11 ± 0.02	0.64296	25	0.999508551	24	0.82003	21	0.998828888	17	0.57741	14	0.121338597	21
Cmpd 3	0.16 ± 0.03	0.66024	22	0.999713957	21	0.83328	15	0.999192025	16	0.58263	10	0.015884425	26
Cmpd 4	0.48 ± 0.10	0.73143	7	0.99981601	15	0.79813	24	0.984722547	22	0.57844	13	0.996034551	9
Cmpd 5	0.38 ± 0.06	0.76482	1	0.999921596	2	0.84429	7	0.999990204	8	0.73027	1	0.999993711	1
Cmpd 6	0.049 ± 0.015	0.67918	18	0.999919005	3	0.83638	14	0.999996899	5	0.69602	3	0.999965009	2
Cmpd 7	0.042 ± 0.018	0.66255	20	0.999906531	6	0.83027	18	0.9998376	12	0.68795	4	0.999953631	3
Cmpd 8	0.18 ± 0.05	0.74970	2	0.999924603	1	0.83255	16	0.99997519	10	0.58508	9	0.999892831	4
Cmpd 9	0.19 ± 0.06	0.74472	3	0.999900484	8	0.81841	22	0.99998355	9	0.56508	17	0.99287337	12
Cmpd 10	0.052 ± 0.012	0.71698	11	0.99984136	13	0.83902	11	0.999999088	4	0.54205	22	0.99855443	8
Cmpd 11	0.058 ± 0.015	0.72044	10	0.999912638	4	0.84336	8	0.999999456	3	0.57050	15	0.99217566	13
Cmpd 12	0.055 ± 0.021	0.71093	12	0.999791909	17	0.84857	5	0.999999616	1	0.53559	26	0.903246571	16
Cmpd 13	0.068 ± 0.024	0.72426	9	0.999842559	12	0.84989	3	0.999999561	2	0.56624	16	0.792054555	18
Cmpd 14	0.049 ± 0.015	0.68889	16	0.999823616	14	0.85430	1	0.999995212	6	0.53826	24	0.999248085	5
Cmpd 15	0.054 ± 0.020	0.69834	14	0.999882242	10	0.84458	6	0.999994072	7	0.55826	20	0.994742037	11
Cmpd 18	0.81 ± 0.31	0.64951	24	0.999759084	18	0.79068	25	0.982254831	23	0.58118	11	0.043186045	23
Cmpd 19	0.71 ± 0.24	0.63961	26	0.999758029	19	0.74611	28	0.488658827	25	0.60118	6	0.024022178	24
Cmpd 20	0.65 ± 0.15	0.58132	28	0.99950168	25	0.76585	27	0.452214723	26	0.56334	18	0.004057203	27
Cmpd 21	0.24 ± 0.048	0.63515	27	0.998296681	27	0.82751	19	0.987714739	21	0.52541	27	0.001956864	28
Cmpd 22	0.096 ± 0.030	0.69148	15	0.999907708	5	0.82403	20	0.9997066	14	0.64447	5	0.885980713	17
Cmpd 23	0.80 ± 0.11	0.65128	23	0.133763512	28	0.83751	13	0.065883334	28	0.59957	7	0.057033535	22
Cmpd 26	0.13 ± 0.06	0.66065	21	0.999849784	11	0.78435	26	0.739866058	24	0.49849	28	0.018047334	25
Cmpd 27	0.034 ± 0.01	0.67104	19	0.999094698	26	0.81224	23	0.312485993	27	0.55879	19	0.17899325	20
Cmpd 29	0.024±0.01	0.73469	5	0.999805517	16	0.85023	2	0.999561672	15	0.53773	25	0.999051277	6
Cmpd 30	0.005±0.001	0.73468	6	0.999882748	9	0.83756	12	0.998271008	18	0.58054	12	0.980322714	14
Cmpd 31	0.036±0.01	0.73689	4	0.999739157	20	0.84278	9	0.999715903	13	0.55454	21	0.937236428	15
Cmpd 32	0.037±0.02	0.73125	8	0.999687835	22	0.83115	17	0.994037289	19	0.59628	8	0.584505246	19
Cmpd 33	0.046± 0.02	0.70680	13	0.999658295	23	0.83916	10	0.992309197	20	0.54140	23	0.998883166	7

Supplementary Table 6. RF and MLP prediction scores across three training strategies of 30 compounds from EJMC 2025.

EJMC, 2025, 289, 117413	AKR1C3 IC_{50} (μM)	Strategy #1				Strategy #2				Strategy #3			
		RF prediction score	RF rank	MLP prediction score	MLP rank	RF prediction score	RF rank	MLP prediction score	MLP rank	RF prediction score	RF rank	MLP prediction score	MLP rank
Cmpd 1	0.61 ± 0.25	0.68163	5	0.999749185	9	0.79048	3	0.999952988	7	0.50306	7	0.991553343	10
Cmpd 2	0.37 ± 0.08	0.66992	11	0.993827004	18	0.74833	12	0.992583293	15	0.44931	17	0.864397275	13
Cmpd 3	0.14 ± 0.08	0.67309	7	0.99961567	10	0.79699	2	0.99944132	9	0.50822	6	0.999328487	4
Cmpd 4	0.22 ± 0.05	0.69380	3	0.999918985	1	0.76799	5	0.99999984	1	0.49938	9	0.999830085	3
Cmpd 5	0.22 ± 0.05	0.69189	4	0.999916416	2	0.76780	6	0.999987171	4	0.49615	10	0.994719854	8
Cmpd 6	0.20 ± 0.32	0.66692	12	0.999784524	8	0.78742	4	0.999994719	2	0.47722	12	0.973217915	12
Cmpd 7	0.27 ± 0.14	0.62335	22	0.998880429	14	0.74925	10	0.999990533	3	0.50983	5	0.994889469	7
Cmpd 9	0.03 ± 0.01	0.67201	8	0.990236878	21	0.72953	17	0.990300429	17	0.47410	13	0.316382973	22
Cmpd 12	0.26 ± 0.03	0.48116	30	0.996210786	17	0.68140	26	0.996477286	14	0.52120	4	0.989561033	11
Cmpd 13	0.51 ± 0.04	0.67504	6	0.979754094	22	0.74516	14	0.978240462	20	0.61025	1	0.862496931	14
Cmpd 14	0.54 ± 0.13	0.66216	14	0.977706249	23	0.74855	11	0.999348824	10	0.59025	2	0.994496578	9
Cmpd 15	0.31 ± 0.05	0.57790	26	0.004460145	30	0.70802	21	0.02811153	28	0.45207	16	0.440033212	18
Cmpd 16	0.48 ± 0.04	0.56338	27	0.016785959	29	0.70784	22	0.403529579	25	0.48271	11	0.600765663	17
Cmpd 17	0.08 ± 0.05	0.60372	24	0.965863497	24	0.67972	27	0.613756711	24	0.38756	20	0.36910452	20
Cmpd 19	0.09 ± 0.02	0.66267	13	0.999510837	12	0.74418	15	0.997854765	12	0.45720	14	0.713941186	16
Cmpd 23	0.26 ± 0.13	0.69440	2	0.999908545	3	0.75860	8	0.999977841	5	0.57493	3	0.999953427	1
Cmpd 29	0.15 ± 0.06	0.65854	16	0.999821555	6	0.75313	9	0.999974466	6	0.44035	19	0.997459397	6
Cmpd 34	1.03 ± 0.11	0.59902	25	0.060943671	28	0.70038	25	0.978022615	21	0.38239	23	0.321187222	21
Cmpd 38	0.18±0.06	0.51829	29	0.639728559	26	0.70857	20	0.99232816	16	0.29304	30	0.016602597	29
Cmpd 11	9.47 ± 1.35	0.64989	19	0.880214596	25	0.76378	7	0.921129567	23	0.44584	18	0.759666183	15
Cmpd 18	NA	0.63008	21	0.623283486	27	0.59113	30	0.000125142	30	0.35779	26	0.086845347	26
Cmpd 20	7.30 ± 1.27	0.54110	28	0.998327072	16	0.66132	29	0.94801301	22	0.35688	27	0.064973709	28
Cmpd 21	＞10	0.67121	10	0.999786119	7	0.74544	13	0.978898307	19	0.36207	25	0.371852288	19
Cmpd 24	NA	0.66199	15	0.99990278	4	0.74385	16	0.997985553	11	0.45282	15	0.997617627	5
Cmpd 28	＞10	0.72355	1	0.999887127	5	0.70652	23	0.246889129	26	0.37027	24	0.112738995	25
Cmpd 30	＞10	0.60611	23	0.99956318	11	0.72539	18	0.084132531	27	0.38428	22	0.228701639	23
Cmpd 32	NA	0.65420	18	0.992763601	20	0.70304	24	0.983839557	18	0.38697	21	0.086710303	27
Cmpd 33	＞10	0.64485	20	0.992953979	19	0.67931	28	0.000728533	29	0.33304	29	0.012230135	30
Cmpd 37	9.76 ± 1.16	0.65439	17	0.999445232	13	0.71684	19	0.99684293	13	0.34689	28	0.164046662	24
Cmpd 39	NA	0.67193	9	0.998827131	15	0.80067	1	0.999750489	8	0.50056	8	0.999928817	2

Supplementary Table 7. Prediction scores and ranking of newly designed Scaffold #1 compounds by RF and MLP models before and after model retraining under strategies #2 and #3.

Sun group's code	Strategy #2								Strategy #3							
	before model-retraining				after model-retraining				before model-retraining				after model-retraining			
	RF prediction score	RF rank	MLP prediction score	MLP rank	RF prediction score	RF rank	MLP prediction score	MLP rank	RF prediction score	RF rank	MLP prediction score	MLP rank	RF prediction score	RF rank	MLP prediction score	MLP rank
S35-1014	0.86197	9	0.999999865	8	0.88347	6	0.999986781	8	0.53177	3	0.991455631	1	0.61102	1	0.949509184	3
S35-1015	0.83683	13	0.999997045	15	0.86478	12	0.999294162	14	0.46970	14	0.24287683	12	0.44871	13	0.096174303	12
S35-1016	0.87488	3	0.999974091	16	0.89584	2	0.997626023	16	0.57164	2	0.750173951	9	0.51588	6	0.839422684	6
S35-1017	0.86320	8	0.999999373	13	0.88104	9	0.999648728	12	0.50048	9	0.228337517	13	0.48392	11	0.008302591	16
S35-1018	0.85627	10	0.999999892	7	0.85837	13	0.999986099	9	0.49586	11	0.319254179	10	0.44758	14	0.294647283	10
S35-1019	0.87300	4	0.999999559	12	0.87915	10	0.999926653	11	0.52702	4	0.860986286	7	0.50040	7	0.463970803	7
S35-1020	0.85597	11	0.999999983	4	0.88490	5	0.999996123	4	0.50875	6	0.87279679	5	0.51606	5	0.300680834	9
S35-1021	0.82840	14	0.999999743	10	0.85706	14	0.999943738	10	0.51413	5	0.865749668	6	0.47849	12	0.417162958	8
S35-1023	0.87172	5	0.999999811	9	0.88185	7	0.999994795	5	0.49597	10	0.312610108	11	0.48937	9	0.04711587	14
S35-1001	0.88064	2	0.999999995	2	0.89193	3	0.999999306	1	0.39771	16	0.000540704	17	0.30973	17	0.000094657	17
S35-1003	0.83746	12	0.999999959	5	0.89060	4	0.999991307	7	0.50138	8	0.188107534	15	0.43376	15	0.116100205	11
S35-1005	0.70858	16	0.999999705	11	0.71176	16	0.999444189	13	0.50182	7	0.952648361	3	0.59308	2	0.992861179	1
S35-1006	0.78160	15	0.999999988	3	0.77109	15	0.999996422	3	0.58428	1	0.033816761	16	0.53267	4	0.080891829	13
S35-1008	0.66275	17	0.999361435	17	0.70601	17	0.927548004	17	0.48660	12	0.893193459	4	0.56382	3	0.945145031	4
S35-1011	0.87007	7	0.999999998	1	0.86969	11	0.999994679	6	0.42164	15	0.976661921	2	0.49026	8	0.979311375	2
S35-1012	0.89613	1	0.999997598	14	0.89699	1	0.998631684	15	0.37320	17	0.758250943	8	0.41037	16	0.92748876	5
S35-1024	0.87172	6	0.999999942	6	0.88185	8	0.9999988	2	0.47669	13	0.191920812	14	0.48937	10	0.032169302	15

Supplementary Table 8. Prediction scores and ranking of newly designed Scaffold #2 compounds by RF and MLP models before and after model retraining under strategies #2 and #3.

Sun group's code	Strategy #2								Strategy #3							
	before model-retraining				after model-retraining				before model-retraining				after model-retraining			
	RF prediction score	RF rank	MLP prediction score	MLP rank	RF prediction score	RF rank	MLP prediction score	MLP rank	RF prediction score	RF rank	MLP prediction score	MLP rank	RF prediction score	RF rank	MLP prediction score	MLP rank
S34-1035	0.78623	16	0.054740183	22	0.76640	15	0.162396109	17	0.45922	5	0.80766951	11	0.48476	2	0.421422736	8
S34-1040	0.77727	18	0.055993225	21	0.77269	9	0.543164261	12	0.54208	2	0.523595158	13	0.41899	7	0.044826057	13
S34-1010	0.79017	14	0.867480452	10	0.75865	17	0.89882176	8	0.39809	13	0.019943188	18	0.38165	13	0.001314386	19
S34-1022	0.80705	10	0.064342867	20	0.77651	6	0.055705341	20	0.36385	18	0.966744146	6	0.35243	15	0.799463249	2
S34-1023	0.82439	4	0.999813551	2	0.77352	8	0.999325425	1	0.32501	21	0.015383524	20	0.28411	22	0.000758691	21
S34-1024	0.75477	20	0.118183315	16	0.70044	22	0.006455156	23	0.47023	4	0.860846827	9	0.44215	5	0.69599791	4
S34-1031	0.73329	22	0.187692899	15	0.73654	20	0.147977906	18	0.43415	9	0.039897158	16	0.38534	12	0.043967053	14
S34-1032	0.73738	21	0.107837903	17	0.73994	19	0.048566745	21	0.43652	8	0.006097156	21	0.41923	6	0.007653312	16
S34-1033	0.80351	12	0.957098133	8	0.77231	10	0.872415396	9	0.42082	11	0.474654051	14	0.40617	8	0.06218716	12
S34-1034	0.78940	15	0.000388608	24	0.76575	16	0.002446538	24	0.42222	10	0.97814085	2	0.38557	11	0.949347903	1
S34-1036	0.71359	23	0.212744847	14	0.68547	23	0.171214471	16	0.45922	6	0.782587743	12	0.48476	3	0.272284505	11
S34-1038	0.79554	13	0.541137797	12	0.77596	7	0.585227164	11	0.45742	7	0.023713432	17	0.44712	4	0.00499415	17
S34-1001	0.81472	8	0.962565069	7	0.76792	12	0.939146269	6	0.37817	16	0.976135293	3	0.34439	16	0.787281588	3
S34-1037	0.83931	1	0.999927423	1	0.83242	1	0.995535559	2	0.38801	15	0.002818047	24	0.39339	10	0.000599676	22
S34-1004	0.82206	7	0.98986838	5	0.76753	13	0.989379289	4	0.32175	23	0.005240944	23	0.26418	24	0.000447901	23
S34-1002	0.83262	2	0.996348964	4	0.79458	3	0.982355045	5	0.32290	22	0.975544425	4	0.33952	17	0.623743793	5
S34-1003	0.82424	5	0.315694589	13	0.78394	4	0.360586989	15	0.33944	19	0.983763276	1	0.33797	18	0.489432328	7
S34-1005	0.83198	3	0.790852755	11	0.76918	11	0.632840515	10	0.28631	24	0.846256734	10	0.28221	23	0.406233946	9
S34-1006	0.80516	11	0.067880315	19	0.75017	18	0.084856231	19	0.36634	17	0.954125381	7	0.33337	19	0.490320084	6
S34-1007	0.82305	6	0.998151638	3	0.79979	2	0.995196755	3	0.39654	14	0.005714505	22	0.33020	20	0.000372845	24
S34-1021	0.76832	19	0.001490698	23	0.76671	14	0.010178438	22	0.51884	3	0.973551045	5	0.37379	14	0.397286643	10
S34-1025	0.78106	17	0.926584657	9	0.72584	21	0.475936667	14	0.40785	12	0.07985683	15	0.39749	9	0.002479702	18
S34-1030	0.64601	24	0.076023925	18	0.64095	24	0.519336842	13	0.61503	1	0.930780253	8	0.65487	1	0.039050152	15
S34-1013	0.81367	9	0.964278512	6	0.77713	5	0.929593358	7	0.32623	20	0.015841071	19	0.30962	21	0.001064575	20

Supplementary Table 9. Representative reported AKR1C3 inhibitors, their biochemical activities, and key limitations relevant to inhibitor development.

Type	Compound structure	Assay type and Value	Limits	Reference
Natural Product Origin	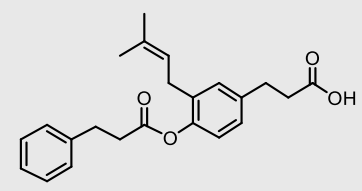	AKR1C3 IC_{50} = 110 nM	Metabolically unstable; synthetically challenging	*Chem.-Biol. Interact.* 2015, 234, 339−348.
	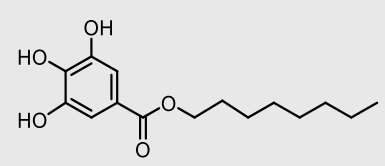	AKR1C3 IC_{50} = 2.56 ± 0.2 μM		*Chem.-Biol. Interact.* 2016, 253,60−65.
	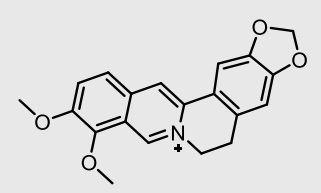	AKR1C3 IC_{50} = 4.08 μM		*Asian J. Androl.* 2016, 18 (4), 607−612.
Organic Ruthenium Complexes	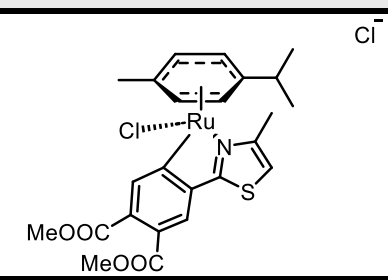	Ki = 560 nM	Poor subtype selectivity; high cytotoxicity	*Chem.-Biol. Interact.* 2015, 234, 349−359.
Off-Label Use Compounds	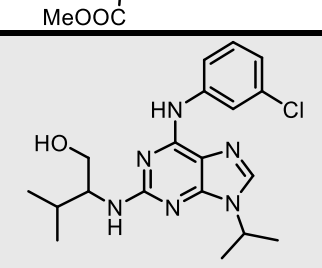	AKR1C3 IC_{50} = 6.58 μM	Weak AKR1C3 activity (μM); severe off-target effects	*Biochem. Pharmacol.* 2018, 156,22−31.
	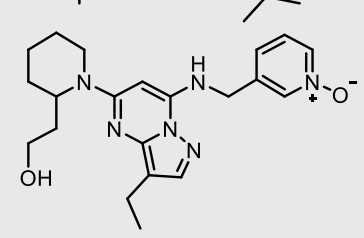	AKR1C3 IC_{50} = 0.23 μM		*Arch.Toxicol.* 2018, 92 (9), 2845−2857.
	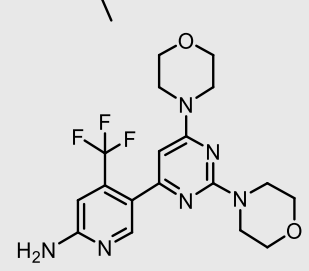	AKR1C3 IC_{50} = 9.5 μM		*Chem.-Biol. Interact.* 2019, 302, 101−107.
	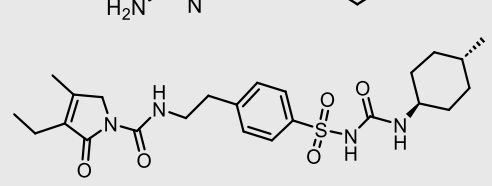	AKR1C3 IC_{50} = 0.85 ± 0.04 μM		*Chem.-Biol. Interact.* 2015, 240, 310−315.
Long-Chain Polyunsaturated Fatty Acids	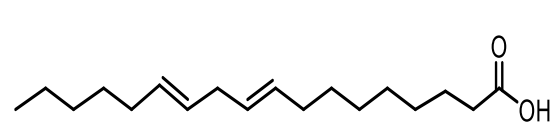	AKR1C3 IC_{50} (when substrate is S-tetralol) = 0.69 μM	Poor subtype selectivity; rapid in vivo metabolism	*J. Biochem.* 2017, 162 (5), 371−379.
Catalytic Substrate Skeletons	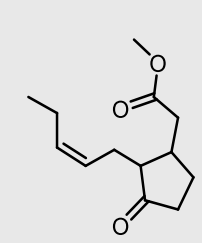	AKR1C3 K_{is} = 153 ± 28 μM/L	Weak AKR1C3 activity (μM); poor subtype selectivity	*Cancer Res.* 2009, 69 (11), 4769−4775.
NSAID Analogues	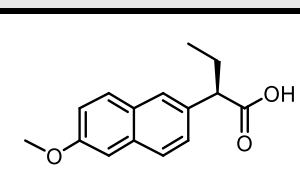	AKR1C3 IC_{50} = 0.11 ± 0.01 μM	Poor cellular permeability	*J. Med. Chem.* 2016, 59 (16), 7431−7444.
	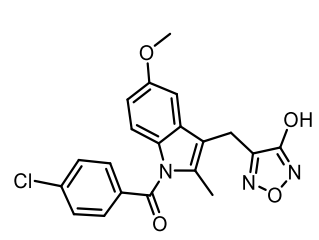	AKR1C3 IC_{50} = 0.30 ± 0.02 μM		*Cancer Cell* 2013, 23, 332−346.
	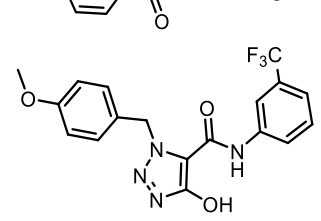	AKR1C3 IC_{50} = 0.31 ± 0.005 μM		*Eur. J. Med. Chem.* 2017, 139, 936−946.

Other AKR1C3 Inhibitors	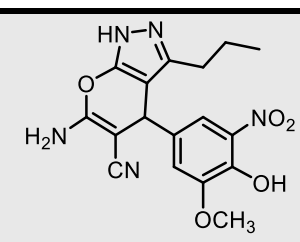	AKR1C3 IC_{50} = 0.160 ± 0.028 μM	Poor subtype selectivity; conformation-al changes at the binding site	*Bioorg. Med. Chem.* 2018, 26 (22), 5934−5943.
	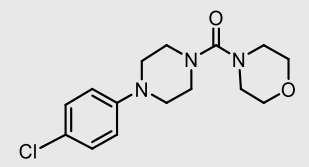	AKR1C3 IC_{50} = 0.11 μM		*Bioorg. Med. Chem.* 2014, 22 (3), 967−977.
	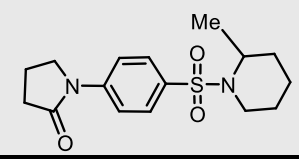	AKR1C3 IC_{50} = 0.094 μM		*Eur. J. Med. Chem.* 2013, 62, 738−744.
Bifunctional Inhibitors	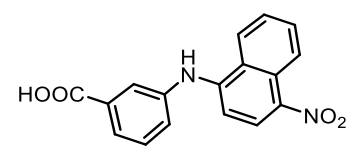	AKR1C3 IC_{50} = 0.086 ± 0.011 μM	Poor subtype selectivity; complex molecular optimization	*Bioorg. Med. Chem. Lett.* 2012, 22 (10), 3492−3497.

Supplementary Figures

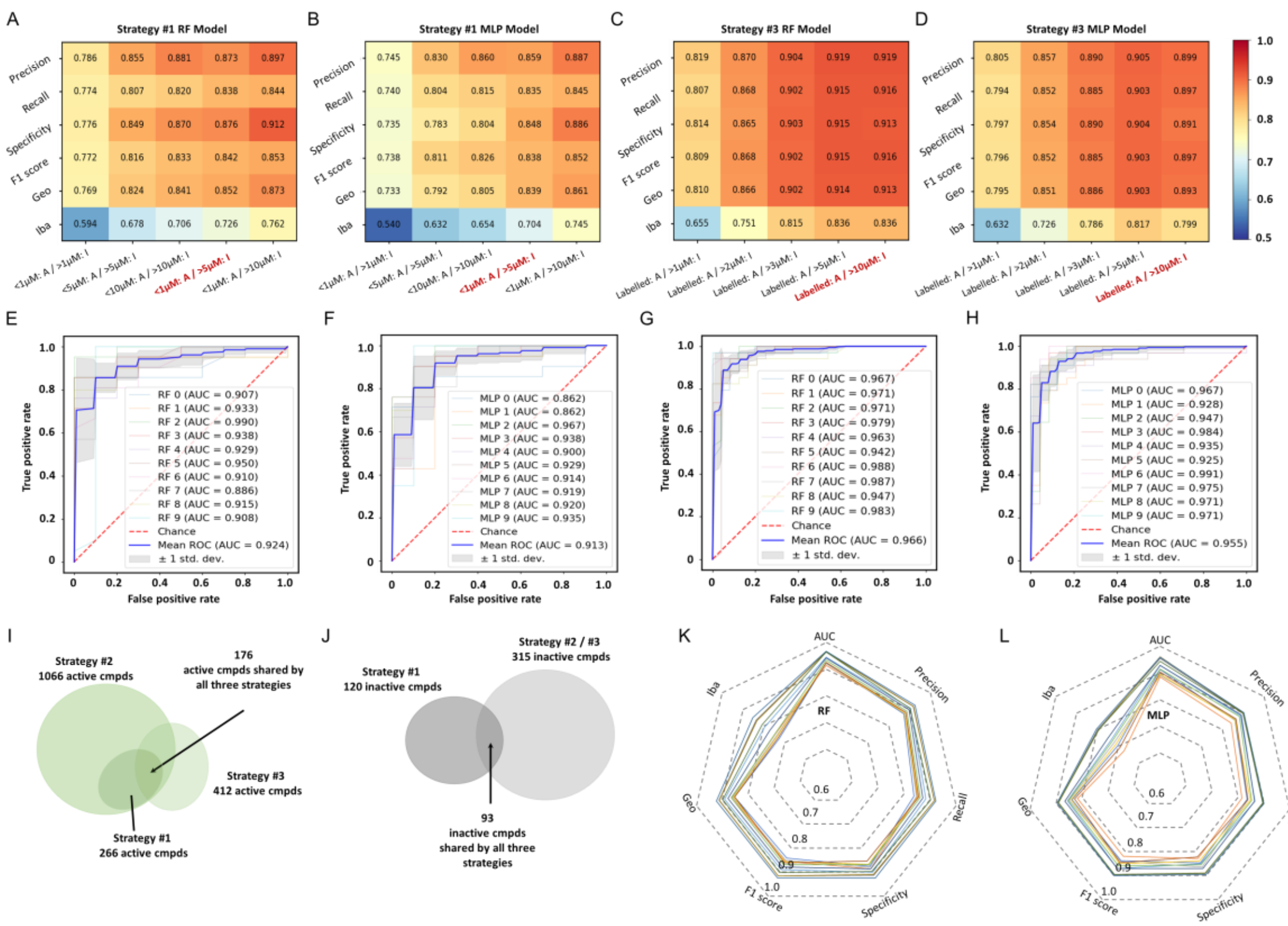

Supplementary Figure 1. Performance evaluation of TAME-VS models under training strategies #1 and #3. A-D. Heatmaps summarizing the AUC values for RF and MLP models trained under strategy #1 (AKR1C3-only dataset) and strategy #3 (combined homolog-derived dataset with manual curation), evaluated across different active–inactive activity cutoff combinations. Warmer colors indicate higher AUROC values. **E-H**. ROC curves for RF and MLP models trained under strategies #1 and #3 using the optimal cutoff combinations identified in panels **A-D**. Shaded regions indicate variability across ten-fold stratified cross-validation. **I, J**. Venn diagrams showing the overlap of active and inactive compounds used in training strategies #1, #2, and #3. **K, L**. Radar plots comparing multiple classification performance metrics—including precision, recall, specificity, F1 score, geometric mean, and index of balanced accuracy—for RF (**K**) and MLP (**L**) models trained under strategies #1 and #3.

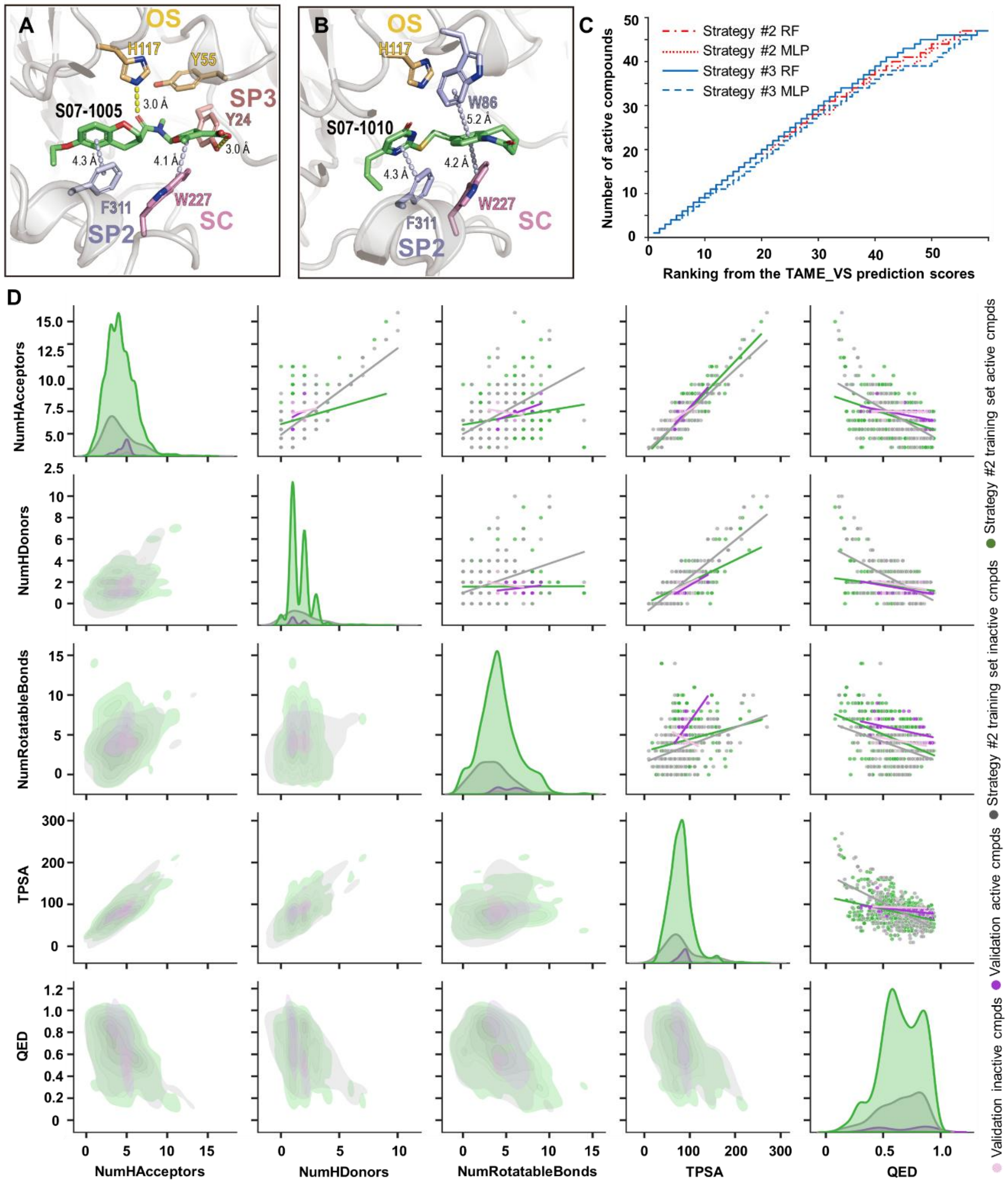

Supplementary Figure 2. Structural basis and physicochemical diversity of retrospective validation compounds. Predicted binding mode of the original hit compounds **S07-2005** (**A**) and **S07-2010** (**B**) in the AKR1C3 active site. Key protein–ligand interactions are illustrated. **C.** Cumulative ranking curves showing the number of active compounds encountered as a function of rank when validation compounds are ordered by prediction score. **D**. Pairwise distributions of key physicochemical descriptors for compounds in the strategy #2 training set and external validation set. Diagonal panels show kernel density estimates of individual properties, whereas

off-diagonal panels depict pairwise relationships between descriptors.

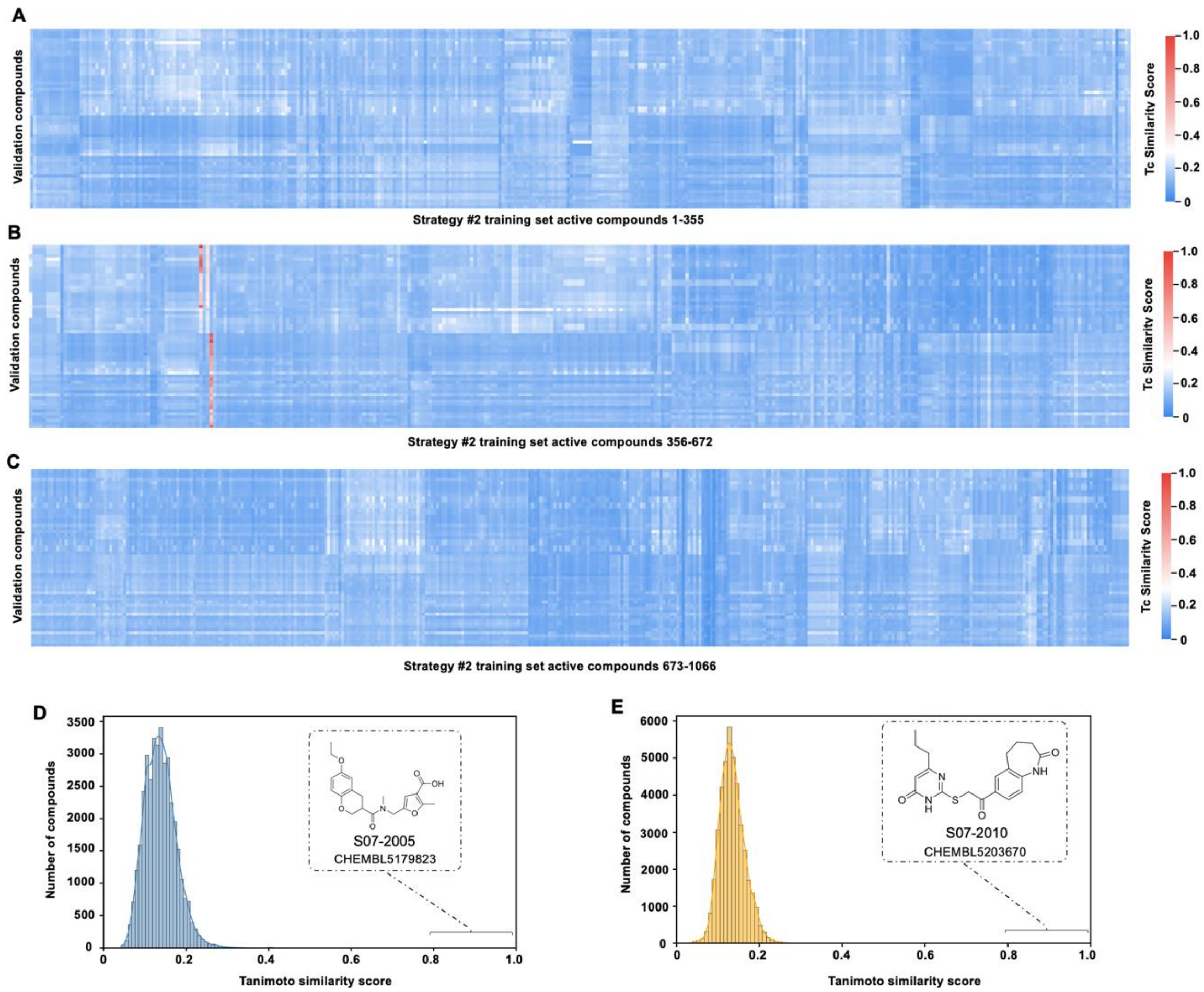

Supplementary Figure 3. Similarities between the actives in Strategy #2 and retrospective validation compounds. A-C. Heatmaps depict Tanimoto similarity scores between active compounds in the strategy #2 training set and retrospective validation compounds, calculated using molecular fingerprints. Rows represent training actives and columns represent validation compounds, with color scale indicating similarity magnitude. **D**. Distribution of Tanimoto similarity scores between 28 compounds from JMC 2025 and the training set compounds used in Strategy #2 with a maximum similarity score of 0.8814 and a mean of 0.1383. **E**. Distribution of Tanimoto similarity scores between 30 compounds from EJMC 2025 and the training set compounds used in Strategy #2 with a maximum score of 0.9636 and a minimum of 0.1350.

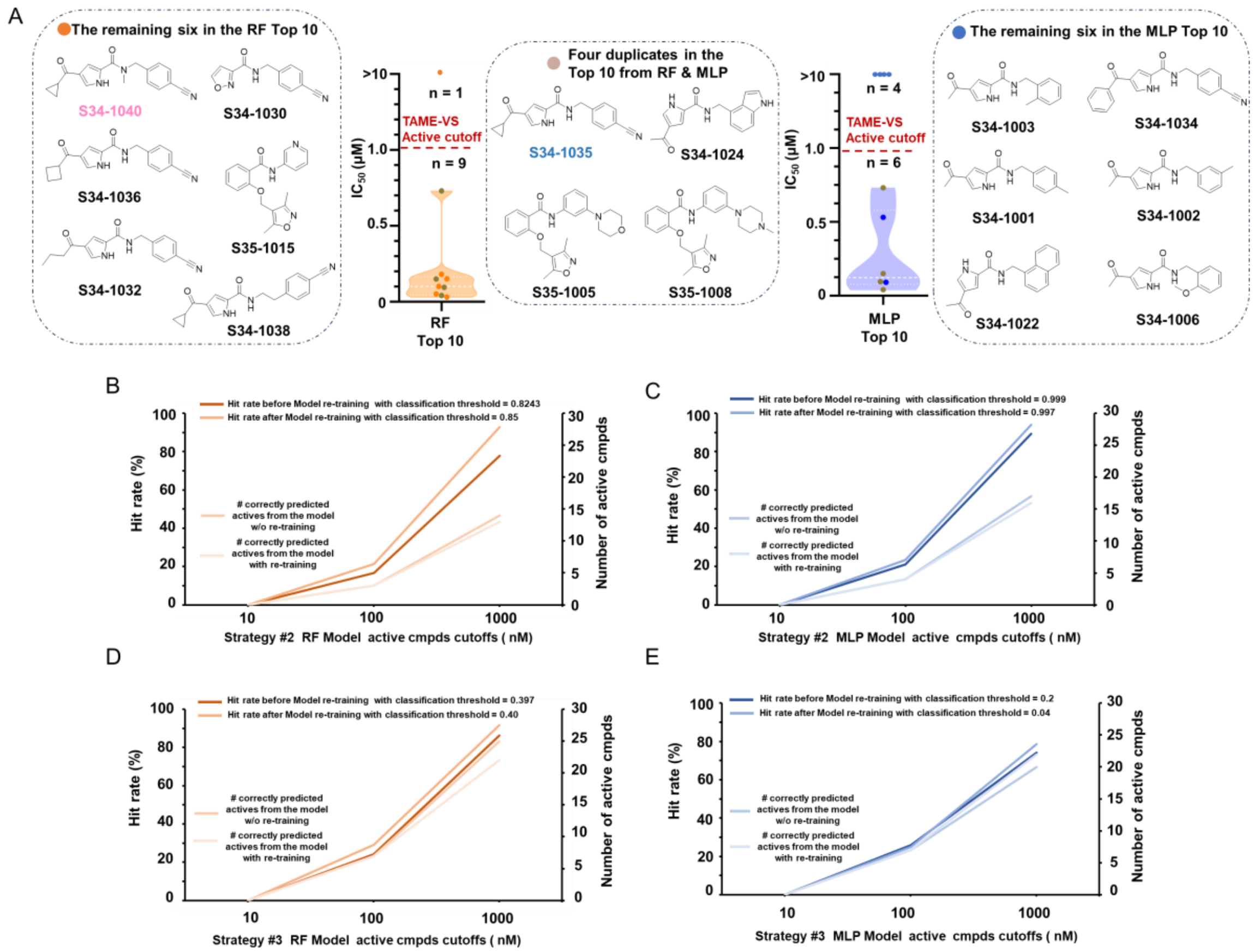

Supplementary Figure 4. Dependence of hit rates on activity cutoffs before and after model retraining. A. Prospective prioritization using Strategy #3 models after retraining, after excluding all compounds previously prioritized under Strategy #2. Chemical structures of the resulting top-10 RF-ranked (left) and top-10 MLP-ranked (right) compounds are shown, with compounds duplicated in the top-10 lists from both models highlighted (center). Hit rates of the RF (**B**) and MLP (**C**) models trained under Strategy #2 evaluated across different activity cutoffs (IC_{50} thresholds) with and without model retraining. Hit rates of the RF (**D**) and MLP (**E**) models trained under Strategy #3 evaluated across different activity cutoffs (IC_{50} thresholds) with and without model retraining. Hit rates are calculated as the fraction of correctly predicted active compounds among prioritized candidates at a fixed classification threshold.

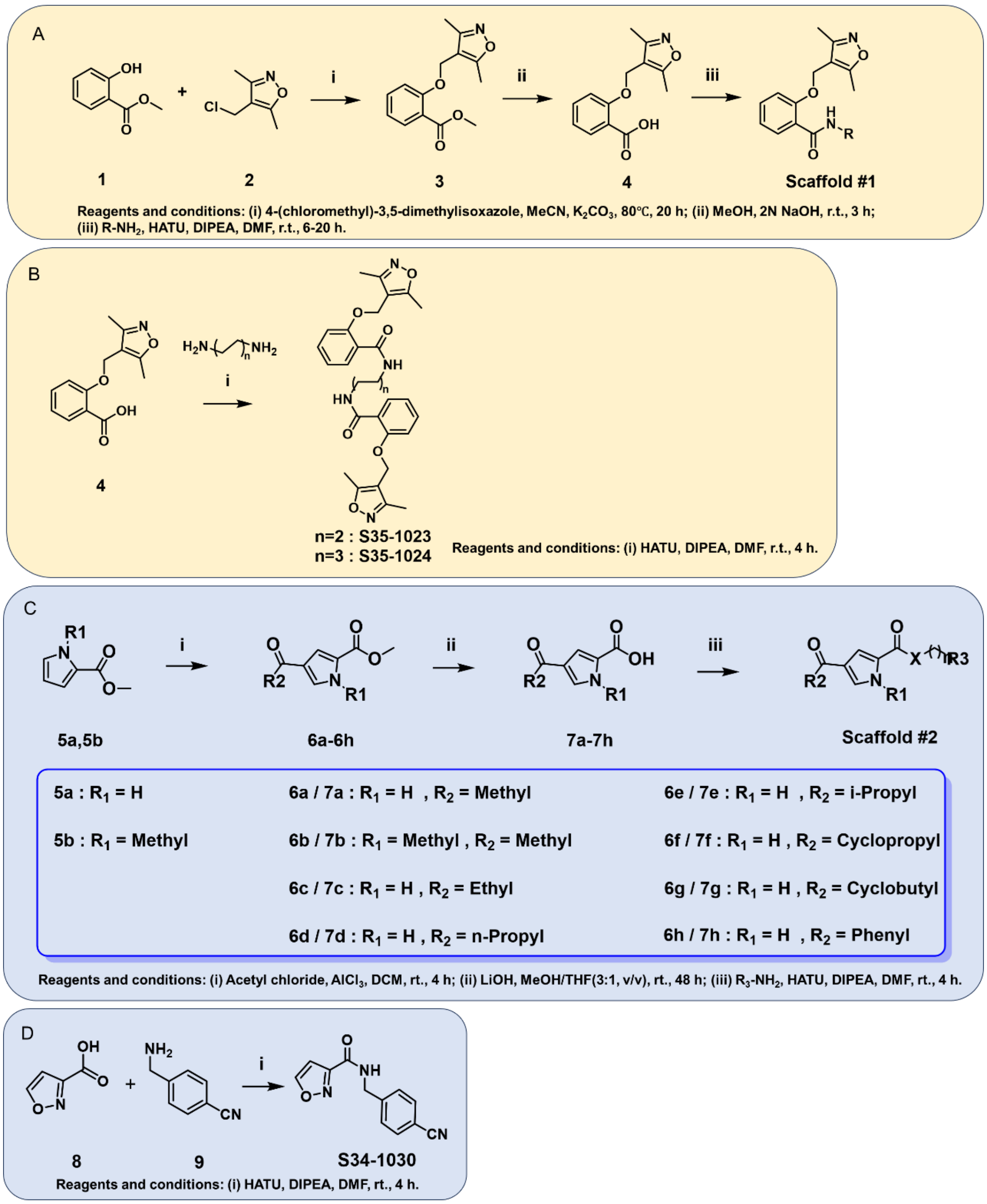

Supplementary Figure 5. Synthetic Routes to Scaffold #1 and Scaffold #2 Analogues. A. Synthesis of Scaffold #1 analogues. Starting from methyl salicylate (**1**) and 4-(chloromethyl)-3,5-dimethylisoxazole (**2**), Intermediate **3** is formed via nucleophilic substitution. Subsequent hydrolysis of the methyl ester yields acid **4**, which undergoes amide coupling with various amines (R–NH_2) using HATU/DIPEA to afford Scaffold #1 derivatives. **B.** Preparation of macrocyclic analogues **S35-1023** and **S35-1024**. Compound **4** reacts with diamines (n = 2 or 3) under HATU/DIPEA conditions to form bis-amide macrocycles through intramolecular cyclization. **C.**

Synthesis of Scaffold #2 analogues. Methyl pyrrole-2-carboxylates (**5a**/**5b**) are acylated with acetyl chloride to yield methyl esters **6a-6h**. Hydrolysis gives corresponding carboxylic acids **7a–7h**, which are then coupled with diverse amines (R_3-NH_2) via HATU-mediated amide bond formation to generate Scaffold #2 analogues. Structural variations include R_1= H or methyl, and R_2 = methyl, ethyl, n-propyl, i-propyl, cyclopropyl, cyclobutyl or phenyl. **D.** Synthesis of compound **S34-1030**. Oxazole-5-carboxylic acid (**8**) is coupled with 4-cyanobenzylamine (**9**) using HATU/DIPEA to yield the amide-linked analogue **S34-1030**.

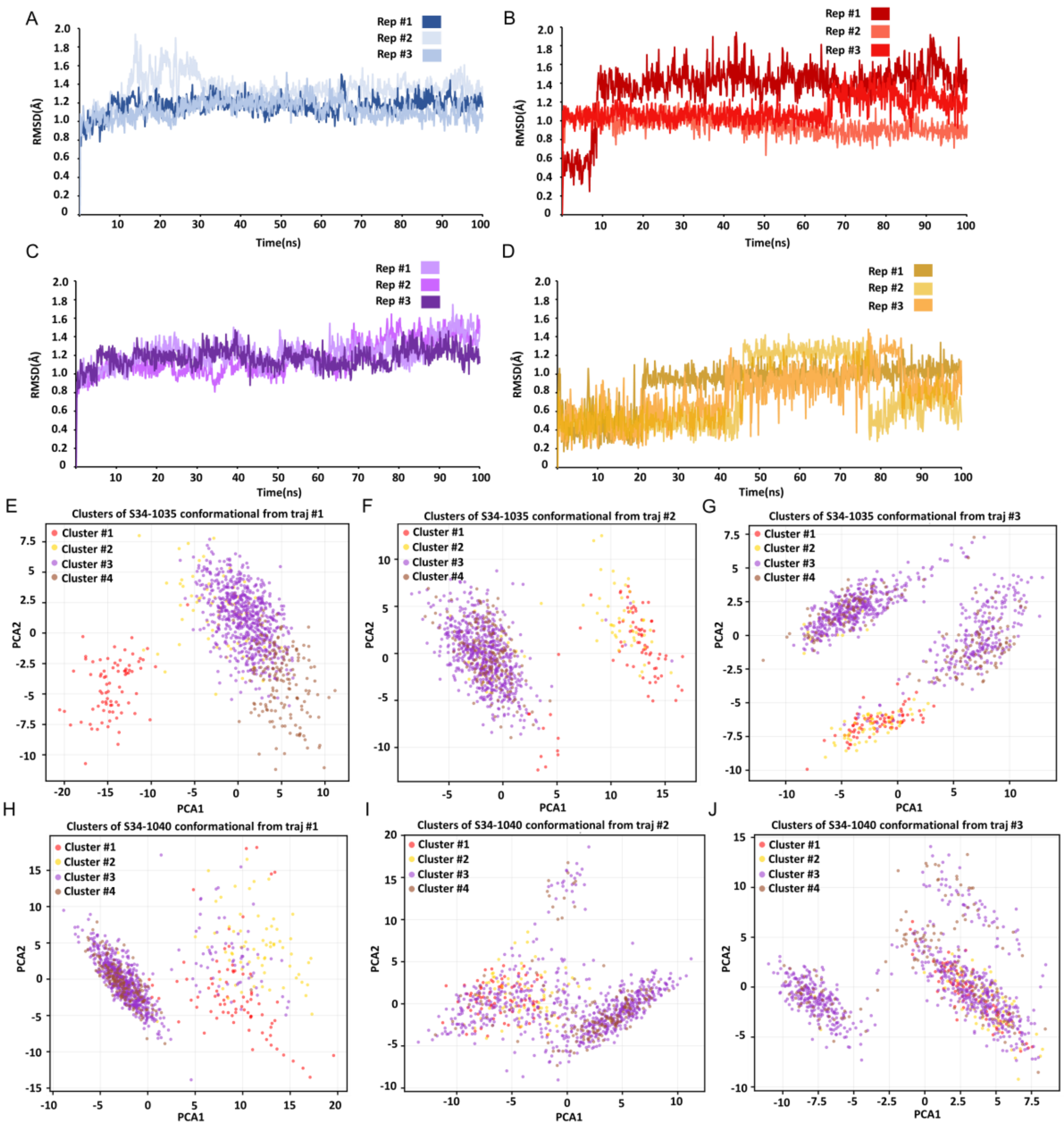

Supplementary Figure 6. Molecular dynamics stability and conformational clustering analysis of S34-1035 and S34-1040 in complex with AKR1C3. A-D. Root-mean-square deviation (RMSD) profiles from three independent 100 ns MD simulations. (**A**) Cα RMSD of AKR1C3 in complex with **S34-1035**. (**B**) Ligand RMSD of **S34-1035** relative to its initial bound conformation. (**C**) Cα RMSD of AKR1C3 in complex with **S34-1040**. (**D**) Ligand RMSD of **S34-1040** relative to its initial bound conformation. **E-J**. Principal component analysis PCA-based conformational clustering of ligand binding modes. (**E-G**) PCA projections of **S34-1035** conformations from three independent trajectories (traj #1-#3). (**H-J**) PCA projections of **S34-1040** conformations from three independent trajectories (traj #1-#3). Each point represents a

simulation frame projected onto the first two principal components (PCA1 and PCA2). Frames are colored according to cluster assignments derived from hierarchical clustering of ligand heavy-atom RMSD matrices: Cluster #1 (red), Cluster #2 (yellow), Cluster #3 (purple), and Cluster #4 (brown).

Supplementary Methods

Synthesis of Scaffold #1 analogues

***Synthesis of methyl 2-((3,5-dimethylisoxazol-4-yl)methoxy)benzoate** (**3**).* Methyl benzoate **1** (140 μL, 1.0 mmol) and 4-(Chloromethyl)-3,5-dimethylisoxazole **2** (125 μL, 1.0 mmol) were dissolved in anhydrous acetonitrile (6.0 mL). Potassium carbonate (207 mg, 1.5 mmol) was added to the reaction mixture, and the resulting suspension was heated to 80 °C and stirred for 20 h under an inert atmosphere. After cooling to ambient temperature, the mixture was slowly quenched with water to induce complete precipitation. The solid was collected by filtration, washed with water (100 mL), and dried under reduced pressure to afford crude product **3**, which was used directly in the subsequent step without further purification (120 mg, 46% yield). ^{1}H NMR (300 MHz, DMSO-d_6) δ 7.67 (dd, J = 7.7, 1.8 Hz, 1H), 7.59 (ddd, J = 9.1, 7.4, 1.8 Hz, 1H), 7.31 (d, J = 8.1 Hz, 1H), 7.08 (td, J = 7.6, 0.8 Hz, 1H), 5.02 (s, 2H), 3.77 (s, 3H), 2.43 (s, 3H), 2.27 (s, 3H).

Synthesis of **2-((3,5-Dimethylisoxazol-4-yl)methoxy)benzoic acid** *(**4**).* Intermediate **3** (1 g, 0.46 mmol) was dissolved in a mixed solvent of tetrahydrofuran and methanol (THF/MeOH, v/v = 3/1, 5 mL). A 2 N aqueous NaOH solution was added dropwise to the mixture at room temperature. The reaction was allowed to proceed for 3 h. Upon completion, THF and MeOH were removed under reduced pressure. The resulting aqueous residue was then carefully acidified by dropwise addition of 2 N HCl solution until the pH fell below 5. The precipitated white solid was collected by vacuum filtration, yielding compound **4** (86 mg, 100% yield). ^{1}H NMR (300 MHz, DMSO-d_6) δ 7.65 (dd, J = 7.7, 1.8 Hz, 1H), 7.58 (ddd, J = 9.1, 7.4, 1.8 Hz, 1H), 7.32 (d, J = 8.1 Hz, 1H), 7.09 (td, J = 7.6, 0.8 Hz, 1H), 5.02 (s, 2H), 2.41 (s, 3H), 2.23 (s, 3H).

Synthesis of Scaffold #1 analogues. Intermediate **4** (1.2 equiv) was dissolved in anhydrous DMF (5.0 mL). To this solution were added sequentially DIPEA (2.5 equiv) and HATU (3.0 equiv). The mixture was stirred for 30 minutes at room temperature to allow activation. Then, various amines with different R groups (1.0 equiv) were added. After the reaction was complete, the mixture was rapidly poured into ice-cold water (50 mL) and extracted with ethyl acetate (3 × 30 mL). The combined organic extracts were washed three times with saturated brine, dried over anhydrous sodium sulfate, and concentrated under reduced pressure to give the crude product. Purification by column chromatography afforded the desired compounds as white or yellow solids in 40–71% yield.

***2-(2-((3,5-dimethylisoxazol-4-yl)methoxy)benzamido)benzoic acid** (**S35-1001**).* According to the general procedure described above, compound **S35-1001** was synthesized as a white solid with a yield of 71%, m.p. 112−113 °C. ^{1}H NMR (300 MHz, DMSO-d_6) 11.88 (s, 1H), 8.70 (d, J = 8.4 Hz, 1H), 8.01 (dd, J = 7.9, 1.7 Hz, 1H), 7.83 (dd, J = 7.7, 1.8 Hz, 1H), 7.69 – 7.54 (m, 2H), 7.33 (d, J = 8.4 Hz, 1H), 7.17 (dt, J = 15.7, 7.5 Hz, 2H), 5.18 (s, 2H), 2.38 (s, 3H), 2.12 (s, 3H). ^{13}C NMR

(151 MHz, DMSO) δ 169.3, 168.1, 164.5, 159.8, 155.7, 141.0, 134.1, 133.2, 131.6, 131.0, 125.4, 123.3, 121.9, 121.0, 118.0, 114.8, 110.3, 60.5, 40.4, 40.3, 40.2, 40.0, 39.9, 39.7, 39.6, 11.1, 10.0. HPLC (75% methanol in water with 1‰ $C_6H_{15}N$): t_R = 1.828 min, 100%. ESI-HRMS (*m*/*z*): $[M + H]^+$ calcd for $C_{20}H_{18}N_2O_5$, 367.1288, found, 367.1289.

2-((3,5-dimethylisoxazol-4-yl)methoxy)-N-(2-hydroxyphenyl)benzamide (S35-1003). According to the general procedure described above, compound **S35-1003** was synthesized as a white solid with a yield of 71%, m.p. 126−128 °C. ^1H NMR (300 MHz, DMSO-*d₆*): δ 10.10 (s, 1H), 9.83 (s, 1H), 8.31 (d, *J* = 8.0 Hz, 1H), 8.08 (dd, *J* = 7.8, 1.9 Hz, 1H), 7.62 (ddd, *J* = 8.9, 7.2, 1.9 Hz, 1H), 7.40 (d, *J* = 8.3 Hz, 1H), 7.23 – 7.13 (m, 1H), 6.96 – 6.87 (m, 2H), 6.84 – 6.75 (m, 1H), 5.21 (s, 2H), 2.44 (s, 3H), 2.21 (s, 3H). ^{13}C NMR (75 MHz, DMSO) δ 168.4, 164.6, 160.1, 155.7, 152.0, 140.2, 132.4, 130.2, 129.7, 126.0, 121.6, 114.1, 111.2, 110.8, 110.5, 106.2, 66.5, 60.3, 48.9, 40.8, 40.5, 40.2, 39.9, 39.7, 39.4, 39.1, 11.1, 10.1. HPLC (75% methanol in water with 1‰ $C_6H_{15}N$): t_R = 4.908 min, 97.420%. ESI-HRMS (*m*/*z*): $[M + H]^+$ calcd for $C_{19}H_{18}N_2O_4$, 339.1399, found, 339.1340.

2-((3,5-dimethylisoxazol-4-yl)methoxy)-N-(3-morpholinophenyl)benzamide (S35-1005). According to the general procedure described above, compound **S35-1005** was synthesized as a white solid with a yield of 69%, m.p. 150−153 °C. ^1H NMR (300 MHz, DMSO-d_6) δ 10.02 (s, 1H), 7.67 (dd, *J* = 7.6, 1.8 Hz, 1H), 7.55 (td, *J* = 8.0, 7.3, 1.8 Hz, 1H), 7.35 (d, *J* = 8.4 Hz, 1H), 7.22 – 7.04 (m, 4H), 6.69 (dt, *J* = 8.0, 1.9 Hz, 1H), 5.08 (s, 2H), 3.76 (t, *J* = 4.8 Hz, 4H), 3.05 (dd, *J* = 6.0, 3.6 Hz, 4H), 2.43 (s, 3H), 2.22 (s, 3H). ^{13}C NMR (75 MHz, DMSO) δ 168.4, 164.6, 160.1, 155.7, 152.0, 140.2, 132.4, 130.2, 129.7, 126.0, 121.6, 114.1, 111.2, 110.8, 110.5, 106.2, 66.5, 60.3, 48.9, 11.1, 10.1. HPLC (75% methanol in water): t_R = 5.797 min, 98.954%. ESI-HRMS (*m*/*z*): $[M + H]^+$ calcd for $C_{23}H_{25}N_3O_4$, 408.1918, found, 408.1919.

2-((3,5-dimethylisoxazol-4-yl)methoxy)-N-(2-morpholinophenyl)benzamide (S35-1006). According to the general procedure described above, compound **S35-1006** was synthesized as a white solid with a yield of 54%, m.p. 163−165 °C. ^1H NMR (300 MHz, DMSO-d_6) δ 10.15 (s, 1H), 8.32 (dd, *J* = 7.7, 1.9 Hz, 1H), 7.97 (dd, *J* = 7.8, 1.8 Hz, 1H), 7.61 (ddd, *J* = 8.9, 7.3, 1.9 Hz, 1H), 7.48 (dd, *J* = 8.4, 1.0 Hz, 1H), 7.25 – 7.09 (m, 4H), 5.26 (s, 2H), 3.63 – 3.51 (m, 4H), 2.69 (t, *J* = 4.5 Hz, 4H), 2.30 (s, 3H), 2.02 (s, 3H). ^{13}C NMR (101 MHz, DMSO) δ 168.4, 163.1, 159.9, 156.1, 142.6, 133.6, 133.4, 131.7, 125.0, 124.7, 124.5, 122.8, 120.9, 120.6, 117.0, 110.4, 66.8, 62.2, 52.3, 40.6, 40.4, 40.2, 40.0, 39.8, 39.6, 39.4, 11.0, 9.8. HPLC (75% methanol in water with 1‰ $C_6H_{15}N$): t_R = 6.382 min, 99.359%. ESI-HRMS (*m*/*z*): $[M + H]^+$ calcd for $C_{23}H_{25}N_3O_4$, 408.1918, found,408.1919.

2-((3,5-dimethylisoxazol-4-yl)methoxy)-N-(3-(4-methylpiperazin-1-yl)phenyl)benzamide (S35-1008). According to the general procedure described above, compound **S35-1008** was

synthesized as a yellow solid with a yield of 58%, m.p. 154−155 °C. ^{1}H NMR (300 MHz, DMSO-d_6) δ 10.00 (s, 1H), 7.68 (dd, *J* = 7.6, 1.8 Hz, 1H), 7.56 (td, *J* = 7.9, 7.4, 1.8 Hz, 1H), 7.35 (d, *J* = 8.3 Hz, 1H), 7.20 – 7.02 (m, 4H), 6.68 (dt, *J* = 7.8, 2.0 Hz, 1H), 5.09 (s, 2H), 3.09 (t, *J* = 5.0 Hz, 4H), 2.48 (d, *J* = 4.7 Hz, 4H), 2.43 (s, 3H), 2.24 (d, *J* = 10.1 Hz, 6H). ^{13}C NMR (101 MHz, DMSO) δ 168.4, 164.6, 160.1, 155.7, 151.9, 140.1, 132.4, 130.2, 129.7, 125.9, 121.6, 114.1, 111.4, 110.5, 110.4, 106.5, 60.3, 55.0, 48.4, 46.2, 40.5, 40.3, 40.1, 39.9, 39.7, 39.5, 39.3, 11.1, 10.1. HPLC (75% methanol in water with 1‰ $C_6H_{15}N$): t_R = 7.500 min, 100%. ESI-HRMS (*m*/*z*): [M + H]$^+$ calcd for $C_{19}H_{18}N_2O_4$, 421.2235, found, 421.2234.

2-((3,5-dimethylisoxazol-4-yl)methoxy)-N-(3-(morpholinomethyl)phenyl)benzamide(S35-1011). According to the general procedure described above, compound **S35-1011** was synthesized as a white solid with a yield of 66%, m.p. 159−160 °C. ^{1}H NMR (300 MHz, DMSO-d_6) δ 10.14 (s, 1H), 7.62 (dd, *J* = 7.6, 1.8 Hz, 1H), 7.57 – 7.48 (m, 3H), 7.34 (dd, *J* = 8.4, 1.0 Hz, 1H), 7.26 (t, *J* = 7.7 Hz, 1H), 7.11 (td, *J* = 7.5, 0.9 Hz, 1H), 7.01 (dt, *J* = 7.6, 1.3 Hz, 1H), 5.06 (s, 2H), 3.59 (t, *J* = 4.6 Hz, 4H), 2.41 (s, 3H), 2.36 (d, *J* = 4.8 Hz, 4H), 2.20 (s, 3H). ^{13}C NMR (75 MHz, DMSO) δ 168.3, 165.0, 160.1, 155.6, 139.4, 139.0, 132.2, 129.9, 129.0, 126.5, 124.6, 121.5, 120.2, 118.4, 114.2, 110.5, 66.6, 63.0, 60.3, 53.6, 40.8, 40.5, 40.2, 40.0, 39.7, 39.4, 39.1, 11.1, 10.1. HPLC (75% methanol in water with 1‰ $C_6H_{15}N$): t_R = 4.560 min, 95.725%. ESI-HRMS (*m*/*z*): [M + H]$^+$ calcd for $C_{24}H_{27}N_3O_4$, 422.2074, found, 422.2075.

2-((3,5-dimethylisoxazol-4-yl)methoxy)-N-(2-(2-hydroxyethyl)phenyl)benzamide (S35-1012). According to the general procedure described above, compound **S35-1012** was synthesized as a white solid with a yield of 52%, m.p. 146−148 °C. ^{1}H NMR (300 MHz, DMSO-d_6) δ 9.74 (s, 1H), 7.72 (dd, *J* = 7.7, 1.8 Hz, 1H), 7.63 (dd, *J* = 8.0, 1.4 Hz, 1H), 7.50 (ddd, *J* = 8.9, 7.3, 1.8 Hz, 1H), 7.27 (d, *J* = 8.3 Hz, 1H), 7.23 – 7.13 (m, 2H), 7.12 – 7.02 (m, 2H), 5.08 (s, 2H), 4.83 (t, *J* = 4.5 Hz, 1H), 3.45 (td, *J* = 6.3, 4.4 Hz, 2H), 2.51 (t, *J* = 6.4 Hz, 2H), 2.37 (s, 3H), 2.15 (s, 3H). ^{13}C NMR (75 MHz, DMSO) δ 168.4, 164.5, 160.1, 155.8, 136.9, 133.4, 132.6, 130.8, 130.6, 126.8, 125.5, 125.3, 124.9, 121.6, 114.2, 110.4, 62.0, 60.5, 40.8, 40.5, 40.2, 40.0, 39.7, 39.4, 39.1, 34.6, 11.2, 10.1. HPLC (75% methanol in water): t_R = 2.720 min, 97.323%. ESI-HRMS (*m*/*z*): [M + H] $^+$ calcd for $C_{21}H_{22}N_2O_4$, 367.1652, found, 367.1653.

2-((3,5-dimethylisoxazol-4-yl)methoxy)-N-(pyridin-2-yl)benzamide (S35-1014). According to the general procedure described above, compound **S35-1014** was synthesized as a white solid with a yield of 59%, m.p. 142−143 °C. ^{1}H NMR (300 MHz, DMSO-d_6) δ 10.37 (s, 1H), 8.77 – 8.70 (m, 1H), 8.29 (d, *J* = 4.8 Hz, 1H), 8.11 (d, *J* = 8.4 Hz, 1H), 7.64 – 7.49 (m, 2H), 7.36 (q, *J* = 8.5, 6.6 Hz, 2H), 7.12 (t, *J* = 7.5 Hz, 1H), 5.06 (s, 2H), 2.39 (s, 3H), 2.18 (s, 3H). ^{13}C NMR (75 MHz, DMSO) δ 168.1, 165.8, 160.1, 155.6, 144.9, 141.3, 136.2, 132.3, 129.8, 126.5, 126.5, 124.2, 121.5, 114.3, 110.5, 60.3, 40.8, 40.5, 40.2, 39.9, 39.7, 39.4, 39.1, 11.1, 10.0. HPLC (75% methanol in water with

1‰ $C_6H_{15}N$): t_R = 4.460 min, 99.788%. ESI-HRMS (*m/z*): $[M + H]^+$ calcd for $C_{18}H_{17}N_3O_3$, 324.1343, found, 324.1343.

2-((3,5-dimethylisoxazol-4-yl)methoxy)-N-(pyridin-3-yl)benzamide (S35-1015). According to the general procedure described above, compound **S35-1015** was synthesized as a white solid with a yield of 53%, m.p. 145−146 °C. ^{1}H NMR (300 MHz, DMSO-*d6*) δ 10.50 (s, 1H), 8.37 – 8.21 (m, 2H), 7.95 (dd, *J* = 7.8, 1.8 Hz, 1H), 7.86 (td, *J* = 7.8, 2.0 Hz, 1H), 7.65 (ddd, *J* = 8.9, 7.3, 1.9 Hz, 1H), 7.46 (d, *J* = 8.3 Hz, 1H), 7.19 (dt, *J* = 11.8, 6.2 Hz, 2H), 5.22 (s, 2H), 2.49 (s, 3H), 2.30 (s, 3H). ^{13}C NMR (101 MHz, DMSO) δ 168.8, 164.2, 160.0, 156.4, 152.0, 148.7, 138.8, 133.6, 131.2, 123.5, 122.0, 120.3, 114.7, 114.2, 110.2, 60.8, 40.6, 40.4, 40.2, 40.0, 39.8, 39.6, 39.4, 11.2, 10.1. HPLC (75% methanol in water with 1‰ $C_6H_{15}N$): t_R = 6.472 min, 99.936%. ESI-HRMS (*m/z*): $[M + H]^+$ calcd for $C_{18}H_{17}N_3O_3$, 324.1343, found, 324.1344.

2-((3,5-dimethylisoxazol-4-yl)methoxy)-N-(pyridin-4-yl)benzamide (S35-1016). According to the general procedure described above, compound **S35-1016** was synthesized as a white solid with a yield of 63%, m.p. 144−146 °C. ^{1}H NMR (300 MHz, DMSO-*d6*) δ 11.39 (s, 1H), 8.96 – 8.90 (m, 2H), 8.21 – 8.16 (m, 2H), 7.92 (dd, *J* = 9.4, 6.8 Hz, 2H), 7.70 (d, *J* = 8.2 Hz, 1H), 7.47 (t, *J* = 7.4 Hz, 1H), 5.38 (s, 2H), 2.71 (s, 3H), 2.49 (s, 3H). ^{13}C NMR (75 MHz, DMSO) δ 168.2, 167.1, 160.0, 155.7, 149.9, 146.7, 132.9, 129.7, 126.0, 121.6, 114.3, 110.5, 60.3, 40.8, 40.5, 40.2, 39.9, 39.7, 39.4, 39.1, 11.1, 10.0. HPLC (75% methanol in water): t_R = 4.118 min, 98.753%. ESI-HRMS (*m/z*): $[M + H]^+$ calcd for $C_{18}H_{17}N_3O_3$, 324.1343, found, 324.1345.

2-((3,5-dimethylisoxazol-4-yl)methoxy)-N-(pyrimidin-2-yl)benzamide (S35-1017). According to the general procedure described above, compound **S35-1017** was synthesized as a white solid with a yield of 40%, m.p. 149−150 °C. ^{1}H NMR (300 MHz, DMSO-*d6*) δ 12.68 (s, 1H), 8.77 (dd, *J* = 4.4, 1.4 Hz, 1H), 8.55 (dd, *J* = 8.4, 1.4 Hz, 1H), 7.63 (dd, *J* = 7.6, 1.8 Hz, 1H), 7.56 – 7.48 (m, 2H), 7.25 (d, *J* = 8.3 Hz, 1H), 7.04 (td, *J* = 7.5, 1.0 Hz, 1H), 4.98 (s, 2H), 2.41 (s, 3H), 2.24 (s, 3H). ^{13}C NMR (75 MHz, DMSO) δ 168.1, 160.2, 159.6, 153.1, 140.5, 137.8, 134.9, 132.7, 130.4, 122.3, 121.7, 115.2, 113.1, 110.2, 60.7, 40.8, 40.5, 40.2, 40.0, 39.7, 39.4, 39.1, 11.1, 10.1. HPLC (75% methanol in water): t_R = 4.552 min, 95.822%. ESI-HRMS (*m/z*): $[M + Na]^+$ calcd for $C_{17}H_{16}N_4O_3$, 347.1115, found, 347.1116.

2-((3,5-dimethylisoxazol-4-yl)methoxy)-N-(pyrimidin-5-yl)benzamide (S35-1018). According to the general procedure described above, compound **S35-1018** was synthesized as a white solid with a yield of 59%, m.p. 142−144 °C. ^{1}H NMR (300 MHz, DMSO-*d6*) δ 10.54 (s, 1H), 9.02 (s, 2H), 8.91 (s, 1H), 7.63 – 7.51 (m, 2H), 7.34 (d, *J* = 8.3 Hz, 1H), 7.13 (td, *J* = 7.5, 0.9 Hz, 1H), 5.06 (s, 2H), 2.38 (s, 3H), 2.17 (s, 3H). ^{13}C NMR (75 MHz, DMSO) δ 168.1, 166.1, 160.0, 155.6, 153.8, 147.6, 134.9, 132.6, 129.8, 126.0, 121.6, 114.4, 110.5, 60.4, 40.8, 40.5, 40.2, 40.0, 39.7, 39.4, 39.1, 11.1, 10.0. HPLC (75% methanol in water with 1‰ $C_6H_{15}N$): t_R = 4.055 min, 100%. ESI-HRMS (*m/z*):

[M + H] + calcd for $C_{17}H_{16}N_4O_3$, 325.1295, found, 325.1296.

2-((3,5-dimethylisoxazol-4-yl)methoxy)-N-(pyrazin-2-yl)benzamide (S35-1019). According to the general procedure described above, compound **S35-1019** was synthesized as a white solid with a yield of 47%, m.p. 141−142 °C. ^{1}H NMR (300 MHz, DMSO-d_6) δ 8.71 (s, 1H), 8.49 (d, *J* = 8.5 Hz, 1H), 7.53 (d, *J* = 36.7 Hz, 4H), 7.21 (d, *J* = 8.5 Hz, 1H), 7.10 – 6.86 (m, 1H), 4.94 (s, 2H), 2.20 (s, 3H). ^{13}C NMR (75 MHz, DMSO) δ 168.1, 160.2, 159.6, 153.1, 140.5, 137.8, 134.9, 132.7, 130.4, 122.3, 121.7, 115.2, 113.1, 110.2, 60.7, 40.8, 40.5, 40.2, 40.0, 39.7, 39.4, 39.1, 11.1, 10.1. HPLC (75% methanol in water): t_R = 4.550 min, 99.063%. ESI-HRMS (*m/z*): [M + Na] + calcd for $C_{17}H_{16}N_4O_3$, 347.1115, found, 347.1099.

2-((3,5-dimethylisoxazol-4-yl)methoxy)-N-(pyridazin-3-yl)benzamide (S35-1020). According to the general procedure described above, compound **S35-1020** was synthesized as a white solid with a yield of 52%, m.p. 149−150 °C. ^{1}H NMR (300 MHz, DMSO-d_6) δ 10.94 (s, 1H), 8.99 (d, *J* = 4.7 Hz, 1H), 8.43 (d, *J* = 9.0 Hz, 1H), 7.85 (d, *J* = 7.6 Hz, 1H), 7.72 (dd, *J* = 9.1, 4.7 Hz, 1H), 7.63 (t, *J* = 7.9 Hz, 1H), 7.42 (d, *J* = 8.4 Hz, 1H), 7.17 (t, *J* = 7.5 Hz, 1H), 5.17 (s, 2H), 2.44 (s, 3H), 2.23 (s, 3H). ^{13}C NMR (75 MHz, DMSO) δ 168.7, 165.4, 160.0, 156.4, 155.7, 149.2, 133.8, 131.0, 129.1, 123.5, 121.9, 118.9, 114.6, 110.2, 60.7, 40.8, 40.5, 40.2, 40.0, 39.7, 39.4, 39.1, 11.2, 10.1. HPLC (75% methanol in water): t_R = 3.983 min, 99.671 %. ESI-HRMS (*m/z*): [M + H] + calcd for $C_{17}H_{16}N_4O_3$, 325.1295, found, 325.1295.

2-((3,5-dimethylisoxazol-4-yl)methoxy)-N-(pyridazin-4-yl)benzamide (S35-1021). According to the general procedure described above, compound **S35-1021** was synthesized as a white solid with a yield of 56%, m.p. 149−151 °C. ^{1}H NMR (300 MHz, DMSO-d_6) δ 11.21 (s, 1H), 9.27 (d, *J* = 2.7 Hz, 1H), 9.21 (d, *J* = 6.2 Hz, 1H), 8.26 (dd, *J* = 6.2, 2.7 Hz, 1H), 7.60 (t, *J* = 7.6 Hz, 2H), 7.38 (d, *J* = 8.3 Hz, 1H), 7.15 (t, *J* = 7.4 Hz, 1H), 5.05 (s, 2H), 2.38 (s, 3H), 2.16 (s, 3H). ^{13}C NMR (75 MHz, DMSO) δ 168.2, 167.5, 160.0, 155.7, 150.7, 143.9, 140.8, 133.2, 129.8, 125.5, 121.6, 115.1, 114.4, 110.4, 60.3, 40.8, 40.5, 40.2, 39.9, 39.7, 39.4, 39.1, 11.1, 10.0. HPLC (75% methanol in water with 1‰ $C_6H_{15}N$): t_R = 3.920 min, 100%. ESI-HRMS (*m/z*): [M + H] + calcd for $C_{17}H_{16}N_4O_3$, 325.1295, found, 325.1296.

N,N'-(butane-1,4-diyl)bis(2-((3,5-dimethylisoxazol-4-yl)methoxy)benzamide)(S35-1023). According to the general procedure described above, compound **S35-1023** was synthesized as a white solid with a yield of 48%, m.p. 177−179 °C. ^{1}H NMR (300 MHz, DMSO) δ 7.98 (t, *J* = 5.6 Hz, 1H), 7.58 (dd, *J* = 7.6, 1.8 Hz, 1H), 7.53 – 7.40 (m, 1H), 7.22 (d, *J* = 8.3 Hz, 1H), 7.09 – 6.98 (m, 1H), 5.02 (s, 2H), 3.13 (d, *J* = 5.7 Hz, 2H), 2.40 (s, 3H), 2.20 (s, 3H), 1.30 (s, 2H). ^{13}C NMR (75 MHz, DMSO) δ 168.2, 165.8, 160.1, 155.8, 131.9, 130.3, 125.6, 121.4, 114.1, 110.5, 60.1, 40.8, 40.5, 40.2, 39.9, 39.7, 39.4, 39.1, 38.8, 26.7, 11.1, 10.1. HPLC (75% methanol in water): t_R = 4.562 min, 96.742%. ESI-HRMS (*m/z*): [M + H] + calcd for $C_{30}H_{34}N_4O_6$, 547.2551, found, 547.2551.

***N,N'-(hexane-1,6-diyl)bis(2-((3,5-dimethylisoxazol-4-yl)methoxy)benzamide)* (*S35-1024*).** According to the general procedure described above, compound **S35-1024** was synthesized as a white solid with a yield of 51%, m.p. 179−180 °C. ^{1}H NMR (300 MHz, DMSO-d6) δ 8.01 (t, *J* = 5.4 Hz, 2H), 7.62 (dd, *J* = 7.6, 1.8 Hz, 2H), 7.52 – 7.44 (m, 2H), 7.25 (d, *J* = 8.3 Hz, 2H), 7.06 (t, *J* = 7.5 Hz, 2H), 5.04 (s, 4H), 3.18 (q, *J* = 6.6 Hz, 4H), 2.43 (s, 6H), 2.23 (s, 6H), 1.34 (s, 4H), 1.14 (s, 4H). ^{13}C NMR (75 MHz, $CDCl_3$) δ 173.0, 170.4, 164.8, 160.6, 136.7, 135.0, 130.2, 126.2, 118.8, 115.2, 64.9, 60.2, 45.5, 45.3, 45.0, 44.7, 44.4, 44.1, 43.9, 34.0, 31.2, 15.8, 14.9. HPLC (75% methanol in water with 1‰ $C_6H_{15}N$): t_R = 5.763 min, 98.145%. ESI-HRMS (*m/z*): [M + H] $^+$ calcd for $C_{32}H_{38}N_4O_6$, 575.2864, found, 575.2863.

Synthesis of Scaffold #2 analogues

Synthesis of methyl 4-acetyl-1H-pyrrole-2-carboxylate (6a). A mixture of 1H-pyrrole-2-carboxylate **5a** (1 g, 7.99 mmol) and anhydrous $AlCl_3$ (2.66 g, 19.98 mmol) in anhydrous CH_2Cl_2 (10 mL) was placed in a round-bottom flask under nitrogen. A solution of acetyl chloride (1.57 g, 19.98 mmol) in anhydrous CH_2Cl_2 (10 mL) was added dropwise via a dropping funnel while cooling in an ice bath. After complete addition, stirring was continued for 5 min in the ice bath, then the reaction was allowed to warm to room temperature and stirred for 4 h. The mixture was quenched by pouring into ice-water, and the pH was adjusted to 7 with saturated $NaHCO_3$ (aq). The mixture was filtered through a diatomaceous earth, and the filtrate was extracted with CH_2Cl_2 (3 × 30 mL). The combined organic layers were washed with brine, dried over Na_2SO_4, and concentrated in vacuo. The residue was purified by column chromatography (petroleum ether/ethyl acetate = 2:1, v/v) to give intermediate **6a** as a white solid (960 mg, 72 %). The intermediates **6b−6h** were synthesized in the general procedure. ^{1}H NMR (400 MHz, Chloroform-*d*) δ 7.58 (dd, *J* = 3.3, 1.6 Hz, 1H), 7.31 (dd, *J* = 2.5, 1.6 Hz, 1H), 3.91 (s, 3H), 2.47 (s, 3H).

Synthesis of 4-acetyl-1H-pyrrole-2-carboxylic acid (7a). **6a** (500 mg, 2.99 mmol) was placed in a 50ml round-bottom flask. Methanol (9 mL) and tetrahydrofuran (3 mL) were added to aid dissolution. A 2 N lithium hydroxide aqueous solution (9 mL) was added dropwise under stirring until the reaction mixture turned from clear to cloudy. The reaction was then stirred at room temperature for 48 hours. Upon completion, most of the solvent was removed under reduced pressure. The pH of the residue was adjusted to 1–2 using 2 N hydrochloric acid, resulting in the precipitation of a white solid. After standing for 10 minutes, the solid was collected by vacuum filtration. The filter cake was dried under an infrared lamp to afford white or brown solid in 94% yield. The intermediates **7b−7h** were synthesized in the general procedure. ^{1}H NMR (300 MHz, DMSO-*d*6) δ 12.24 (s, 1H), 7.56 (dd, *J* = 3.3, 1.7 Hz, 1H), 6.93 (t, *J* = 2.0 Hz, 1H), 2.23 (s, 3H).

Synthesis of Scaffold #2 analogues. The intermediate acid (1.0 equiv) and HATU (1.5 equiv) were placed in a 25 mL round-bottom flask. Anhydrous DMF (3 mL) was added, and DIPEA (1.5 equiv) was introduced dropwise under stirring. The mixture was stirred at room temperature for 30 min, after which a solution of amine (1.5 equiv) in anhydrous DMF (3 mL) was added dropwise. Stirring was continued at room temperature for 4 h. Upon completion, water (10 mL) was added, and the mixture was stirred for an additional 10 min. The resulting mixture was extracted with ethyl acetate (3 × 30 mL). The combined organic layers were washed with saturated brine, dried over Na_2SO_4, and concentrated under reduced pressure to afford the crude product. Purification by column chromatography on silica gel (dichloromethane/methanol = 10:1, v/v) furnished the *Scaffold #2* series compounds in 55-86% yield .

4-acetyl-N-(4-methylbenzyl)-1H-pyrrole-2-carboxamide (S34-1001). According to the general

procedure described above, compound **S34-1001** was synthesized as a white solid with a yield of 60% , m.p. 132-134 °C. ^{1}H NMR (300 MHz, DMSO-d_6) δ 12.17 (s, 1H), 8.81 (t, *J* = 6.1 Hz, 1H), 7.65 (s, 1H), 7.25 (s, 1H), 7.17 (d, *J* = 8.8 Hz, 4H), 4.39 (d, *J* = 6.0 Hz, 2H), 2.35 (s, 3H), 2.28 (s, 3H). ^{13}C NMR (126 MHz, DMSO) δ 193.0, 160.6, 137.1, 136.2, 129.3, 128.3, 127.7, 127.2, 126.4, 109.8, 42.2, 40.5, 40.3, 40.2, 40.0, 39.8, 39.7, 39.5, 27.5, 21.1. HPLC (50% acetonitrile in water): t_R = 5.275 min, 99.418%. ESI-HRMS (*m*/*z*): [M + H]$^+$ calcd for $C_{15}H_{16}N_2O_2$, 257.1285, found, 257.1285.

4-acetyl-N-(3-methylbenzyl)-1H-pyrrole-2-carboxamide (S34-1002). According to the general procedure described above, compound **S34-1002** was synthesized as a white solid with a yield of 58%, m.p. 130-132 °C. ^{1}H NMR (500 MHz, DMSO) δ 12.12 (s, 1H), 8.77 (t, *J* = 6.1 Hz, 1H), 7.62 (d, *J* = 1.7 Hz, 1H), 7.24 (d, *J* = 1.5 Hz, 1H), 7.20 (t, *J* = 7.5 Hz, 1H), 7.12 – 7.02 (m, 3H), 4.39 (d, *J* = 6.0 Hz, 2H), 2.33 (s, 2H), 2.28 (s, 2H). ^{13}C NMR (126 MHz, DMSO) δ 193.0, 160.6, 140.0, 137.8, 128.7, 128.3, 128.2, 127.9, 127.2, 126.4, 124.8, 109.8, 42.4, 40.5, 40.3, 40.2, 40.0, 39.8, 39.7, 39.5, 27.5, 21.5. HPLC (50% acetonitrile in water): t_R = 5.576 min, 100.000%. ESI-HRMS (*m*/*z*): [M + H]$^+$ calcd for $C_{15}H_{16}N_2O_2$, 257.1285, found, 257.1286.

4-acetyl-N-(2-methylbenzyl)-1H-pyrrole-2-carboxamide (S34-1003). According to the general procedure described above, compound **S34-1003** was synthesized as a white solid with a yield of 62%, m.p. 131-133 °C. ^{1}H NMR (400 MHz, DMSO-d_6) δ 12.14 (s, 1H), 8.66 (t, *J* = 5.8 Hz, 1H), 7.63 (dd, *J* = 3.3, 1.6 Hz, 1H), 7.28 (dd, *J* = 2.5, 1.6 Hz, 1H), 7.22 (t, *J* = 4.0 Hz, 1H), 7.15 (d, *J* = 3.0 Hz, 3H), 4.41 (d, *J* = 5.8 Hz, 2H), 2.33 (s, 3H), 2.30 (s, 3H). ^{13}C NMR (126 MHz, DMSO) δ 193.1, 160.6, 137.6, 136.0, 130.4, 128.2, 127.9, 127.3, 127.2, 126.4, 126.2, 109.9, 40.5, 40.3, 40.1, 40.0, 39.8, 39.6, 39.5, 27.5, 19.1. HPLC (50%acetonitrile in water): t_R = 5.432 min, 100.000%. ESI-HRMS (*m*/*z*): [M + H]$^+$ calcd for $C_{15}H_{16}N_2O_2$, 257.1285, found, 257.1284.

4-acetyl-N-(4-methoxybenzyl)-1H-pyrrole-2-carboxamide (S34-1004). According to the general procedure described above, compound **S34-1004** was synthesized as a white solid with a yield of 71%, m.p. 136-138 °C. ^{1}H NMR (300 MHz, DMSO-d_6) δ 12.12 (s, 1H), 8.75 (t, *J* = 5.4 Hz, 1H), 7.63 (s, 1H), 7.24 (d, *J* = 7.0 Hz, 3H), 6.90 (d, *J* = 8.1 Hz, 2H), 4.37 (d, *J* = 5.9 Hz, 2H), 3.74 (s, 3H), 2.35 (s, 3H). ^{13}C NMR (101 MHz, DMSO) δ 193.1, 160.5, 158.6, 132.1, 129.1, 128.3, 127.2, 126.4, 114.1, 109.8, 55.5, 41.9, 40.6, 40.4, 40.2, 39.9, 39.7, 39.5, 39.3, 27.5. HPLC (50% acetonitrile in water): t_R = 4.367 min, 100.000%. ESI-HRMS (*m*/*z*): [M + H]$^+$ calcd for $C_{15}H_{16}N_2O_3$: 273.1234, found 273.1234.

4-acetyl-N-(3-methoxybenzyl)-1H-pyrrole-2-carboxamide (S34-1005). According to the general procedure described above, compound **S34-1005** was synthesized as a white solid with a yield of 67%, m.p. 134-136 °C. ^{1}H NMR (300 MHz, DMSO-d_6) δ 12.14 (s, 1H), 8.79 (t, *J* = 6.1 Hz, 1H), 7.63

(dd, *J* = 3.3, 1.6 Hz, 1H), 7.28 – 7.19 (m, 2H), 6.90 – 6.77 (m, 3H), 4.40 (d, *J* = 6.0 Hz, 2H), 3.72 (s, 3H), 2.33 (s, 3H). ^{13}C NMR (101 MHz, DMSO) δ 193.1, 160.7, 159.8, 141.7, 129.8, 128.2, 127.3, 126.4, 119.8, 113.4, 112.5, 109.8, 55.4, 42.4, 40.6, 40.4, 40.2, 40.0, 39.7, 39.5, 39.3, 27.5. HPLC (50% acetonitrile in water): t_R = 4.462 min, 100.000%. ESI-HRMS (*m/z*): [M + H]$^+$ calcd for $C_{15}H_{16}N_2O_3$: 273.1234, found 273.1231.

4-acetyl-N-(2-methoxybenzyl)-1H-pyrrole-2-carboxamide (S34-1006). According to the general procedure described above, compound **S34-1006** was synthesized as a white solid with a yield of 86%, m.p. 138-140 °C. ^{1}H NMR (300 MHz, DMSO-*d₆*) δ 12.12 (s, 1H), 8.61 (t, *J* = 5.7 Hz, 1H), 7.63 (dd, *J* = 3.4, 1.6 Hz, 1H), 7.30 – 7.19 (m, 2H), 7.16 (dd, *J* = 7.7, 1.7 Hz, 1H), 6.98 (d, *J* = 8.1 Hz, 1H), 6.94 – 6.86 (m, 1H), 4.39 (d, *J* = 5.9 Hz, 2H), 3.82 (s, 3H), 2.34 (s, 3H). ^{13}C NMR (101 MHz, DMSO) δ 193.1, 160.8, 157.0, 128.4, 128.3, 127.8, 127.3, 127.2, 126.4, 120.6, 110.9, 109.8, 55.8, 40.6, 40.4, 40.2, 40.0, 39.8, 39.6, 39.3, 37.5, 27.5. HPLC (50% acetonitrile in water): t_R = 5.057 min, 100.000%. ESI-HRMS (*m/z*): [M + H]$^+$ calcd for $C_{15}H_{16}N_2O_3$: 273.1234, found 273.1234.

4-acetyl-N-(4-fluorobenzyl)-1H-pyrrole-2-carboxamide (S34-1007). According to the general procedure described above, compound **S34-1007** was synthesized as a white solid with a yield of 71%, m.p. 127-129 °C. ^{1}H NMR (300 MHz, DMSO-*d₆*) δ 12.22 (s, 1H), 8.95 (t, *J* = 6.2 Hz, 1H), 7.66 – 7.59 (m, 1H), 7.33 (dd, *J* = 8.3, 5.5 Hz, 2H), 7.24 (s, 1H), 7.14 (t, *J* = 8.7 Hz, 2H), 4.40 (d, *J* = 5.9 Hz, 2H), 2.33 (s, 3H). ^{13}C NMR (75 MHz, DMSO) δ 193.1, 160.6, 136.4, 136.4, 129.7, 129.6, 128.2, 127.2, 126.4, 115.6, 115.3, 110.2, 41.7, 40.8, 40.5, 40.2, 39.9, 39.7, 39.4, 39.1, 27.5. HPLC (50% acetonitrile in water): t_R = 4.688 min, 100.000%. ESI-HRMS (*m/z*): [M + H]$^+$ calcd for $C_{14}H_{13}FN_2O$: 261.1034, found 261.1031.

4-acetyl-N-(4-cyanobenzyl)-1H-pyrrole-2-carboxamide (S34-1010). According to the general procedure described above, compound **S34-1010** was synthesized as a white solid with a yield of 59%, m.p. 143-145 °C. ^{1}H NMR (300 MHz, DMSO-*d₆*) δ 12.16 (s, 1H), 8.92 (t, *J* = 6.1 Hz, 1H), 7.82 (d, *J* = 7.9 Hz, 2H), 7.66 (s, 1H), 7.50 (d, *J* = 7.9 Hz, 2H), 7.27 (s, 1H), 4.52 (d, *J* = 6.0 Hz, 2H), 2.36 (s, 3H). ^{13}C NMR (75 MHz, DMSO) δ 193.1, 160.9, 146.1, 132.8, 128.4, 127.9, 127.5, 126.5, 119.4, 110.0, 110.0, 42.2, 40.8, 40.5, 40.2, 39.9, 39.6, 39.4, 39.1, 27.5. HPLC (50% acetonitrile in water): t_R = 4.004 min, 98.933%. ESI-HRMS (*m/z*): [M + H]$^+$ calcd for $C_{15}H_{13}N_3O_2$: 268.1081, found 268.1079.

4-acetyl-N-(4-(tert-butyl)benzyl)-1H-pyrrole-2-carboxamide (S34-1013). According to the general procedure described above, compound **S34-1013** was synthesized as a white solid with a yield of 62%, m.p. 126-128 °C. ^{1}H NMR (300 MHz, DMSO-*d₆*) δ 12.13 (s, 1H), 8.76 (t, *J* = 5.7 Hz, 1H), 7.66 – 7.58 (m, 1H), 7.34 (d, *J* = 8.1 Hz, 2H), 7.22 (d, *J* = 8.3 Hz, 3H), 4.38 (d, *J* = 5.8 Hz, 2H), 2.33 (s, 3H), 1.26 (s, 9H). ^{13}C NMR (75 MHz, DMSO) δ 193.1, 160.6, 149.6, 137.1, 128.3, 127.6, 127.2, 126.4, 125.5, 109.8, 42.2, 40.8, 40.5, 40.2, 39.9, 39.7, 39.4, 39.1, 34.6, 31.6, 27.5. HPLC

(50% acetonitrile in water): t_R = 9.927 min, 99.901%. ESI-HRMS (*m/z*): $[M + H]^+$ calcd for $C_{18}H_{22}N_2O_2$: 299.1754, found 299.1754.

4-acetyl-N-(benzo[d][1,3]dioxol-5-ylmethyl)-1H-pyrrole-2-carboxamide (S34-1021). According to the general procedure described above, compound **S34-1021** was synthesized as a white solid with a yield of 64%, m.p. 150-152 °C. ^{1}H NMR (300 MHz, DMSO-d_6) δ 12.16 (s, 1H), 8.77 (t, *J* = 6.1 Hz, 1H), 7.63 (s, 1H), 7.23 (s, 1H), 6.89 – 6.82 (m, 2H), 6.77 (dd, *J* = 8.0, 1.6 Hz, 1H), 5.98 (s, 2H), 4.33 (d, *J* = 5.9 Hz, 2H), 2.34 (s, 3H). ^{13}C NMR (75 MHz, DMSO) δ 193.1, 160.6, 147.7, 146.5, 134.0, 128.2, 127.2, 126.4, 120.9, 109.8, 108.5, 108.4, 101.3, 42.3, 40.8, 40.5, 40.2, 39.9, 39.7, 39.4, 39.1, 27.5. HPLC (50% acetonitrile in water): t_R = 4.252 min, 100.000%. ESI-HRMS (*m/z*): $[M + H]^+$ calcd for $C_{15}H_{14}N_2O_4$: 287.1026, found 287.1022.

4-acetyl-N-(naphthalen-1-ylmethyl)-1H-pyrrole-2-carboxamide (S34-1022). According to the general procedure described above, compound **S34-1022** was synthesized as a white solid with a yield of 73%, m.p. 97-99 °C. ^{1}H NMR (300 MHz, DMSO-d_6) δ 12.19 (s, 1H), 8.84 (t, *J* = 5.7 Hz, 1H), 8.19 (d, *J* = 8.2 Hz, 1H), 8.01 – 7.92 (m, 1H), 7.92 – 7.83 (m, 1H), 7.65 (d, *J* = 1.6 Hz, 1H), 7.60 – 7.47 (m, 4H), 7.30 (d, *J* = 1.6 Hz, 1H), 4.93 (d, *J* = 5.7 Hz, 2H), 2.35 (s, 3H). ^{13}C NMR (75 MHz, DMSO) δ 193.1, 160.6, 135.1, 133.8, 131.3, 129.0, 128.1, 128.0, 127.3, 126.7, 126.5, 126.3, 125.9, 124.0, 110.0, 40.7, 40.6, 40.5, 40.2, 39.9, 39.6, 39.4, 39.1, 27.5. HPLC (50% acetonitrile in water): t_R = 6.383 min, 100.000%. ESI-HRMS (*m/z*): $[M + H]^+$ calcd for $C_{18}H_{16}N_2O_2$: 293.1285, found 293.1286.

4-acetyl-N-(naphthalen-2-ylmethyl)-1H-pyrrole-2-carboxamide (S34-1023). According to the general procedure described above, compound **S34-1023** was synthesized as a white solid with a yield of 83%, m.p. 95-97 °C. ^{1}H NMR (300 MHz, DMSO-d_6) δ 12.18 (s, 1H), 8.92 (t, *J* = 6.1 Hz, 1H), 7.91 (d, *J* = 8.5 Hz, 3H), 7.80 (s, 1H), 7.67 (dd, *J* = 3.3, 1.6 Hz, 1H), 7.56 – 7.44 (m, 3H), 7.31 (t, *J* = 2.0 Hz, 1H), 4.63 (d, *J* = 6.0 Hz, 2H), 2.37 (s, 3H). ^{13}C NMR (75 MHz, DMSO) δ 193.1, 160.8, 137.7, 133.3, 132.6, 128.4, 128.2, 128.0, 127.3, 126.7, 126.5, 126.4, 126.1, 125.7, 109.9, 42.6, 40.8, 40.5, 40.2, 40.0, 39.7, 39.4, 39.1, 27.5. HPLC (50% acetonitrile in water): t_R = 6.464 min, 98.565%. ESI-HRMS (*m/z*): $[M + H]^+$ calcd for $C_{18}H_{16}N_2O_2$: 293.1285, found 293.1283.

N-((1H-indol-4-yl)methyl)-4-acetyl-1H-pyrrole-2-carboxamide (S34-1024). According to the general procedure described above, compound **S34-1024** was synthesized as a white solid with a yield of 74%, m.p. 164-166 °C. ^{1}H NMR (300 MHz, DMSO-d_6) δ 12.17 (s, 1H), 11.15 (s, 1H), 8.78 (s, 1H), 7.65 (s, 1H), 7.42 – 7.21 (m, 3H), 7.00 (dd, *J* = 31.0, 7.5 Hz, 2H), 6.60 (s, 1H), 4.73 (d, *J* = 6.0 Hz, 2H), 2.36 (s, 3H). ^{13}C NMR (101 MHz, DMSO) δ 193.1, 160.5, 136.3, 130.9, 128.4, 127.1, 126.6, 126.4, 125.4, 121.2, 117.9, 110.8, 109.8, 100.0, 41.0, 40.5, 40.3, 40.1, 39.9, 39.7, 39.5, 39.3, 27.5. HPLC (50% acetonitrile in water): t_R = 3.986 min, 100.000%. ESI-HRMS (*m/z*): $[M + H]^+$ calcd for $C_{16}H_{15}N_3O_2$: 282.1237, found 282.1239.

N-((1H-indol-6-yl)methyl)-4-acetyl-1H-pyrrole-2-carboxamide (S34-1025). According to the general procedure described above, compound **S34-1025** was synthesized as a white solid with a yield of 85%, m.p. 163-165 °C. ^{1}H NMR (400 MHz, DMSO-d_6) δ 12.13 (s, 1H), 11.03 (s, 1H), 8.75 (t, *J* = 5.96 Hz, 1H), 7.62 (dd, *J* = 3.3, 1.6 Hz, 1H), 7.49 – 7.44 (m, 1H), 7.36 – 7.30 (m, 2H), 7.25 (dd, *J* = 2.6, 1.6 Hz, 1H), 7.05 (dd, *J* = 8.3, 1.6 Hz, 1H), 6.38 (ddd, *J* = 3.0, 2.0, 0.9 Hz, 1H), 4.50 (d, *J* = 6.0 Hz, 2H), 2.33 (s, 3H). ^{13}C NMR (126 MHz, DMSO) δ 193.1, 160.5, 135.5, 130.3, 128.5, 128.0, 127.1, 126.4, 126.0, 121.5, 119.2, 111.6, 109.7, 101.4, 43.0, 40.4, 40.3, 40.1, 39.9, 39.8, 39.6, 39.4, 27.5. HPLC (50% acetonitrile in water): t_R = 4.016 min, 100.000%. ESI-HRMS (*m/z*): [M - H]$^-$ calcd for $C_{16}H_{15}N_3O_2$: 280.1091, found 280.1089.

N-(4-cyanobenzyl)isoxazole-3-carboxamide (S34-1030). According to the general procedure described above, compound **S34-1030** was synthesized as a white solid with a yield of 67%, m.p. 97-99 °C. ^{1}H NMR (400 MHz, DMSO-d_6) δ 9.49 (t, *J* = 6.2 Hz, 1H), 9.12 (d, *J* = 1.7 Hz, 1H), 7.87 – 7.75 (m, 2H), 7.51 (d, *J* = 8.0 Hz, 2H), 6.93 (d, *J* = 1.7 Hz, 1H), 4.53 (d, *J* = 6.1 Hz, 2H). ^{13}C NMR (101 MHz, DMSO) δ 162.1, 159.2, 158.2, 145.2, 132.8, 128.6, 119.3, 110.2, 104.8, 42.6, 40.6, 40.4, 40.2, 40.0, 39.7, 39.5, 39.3. HPLC (50% acetonitrile in water): t_R = 5.159 min, 99.515%. ESI-HRMS (*m/z*): [M + H]$^+$ calcd for $C_{12}H_9N_3O_2$: 228.0768, found 228.0770.

N-(4-cyanobenzyl)-4-propionyl-1H-pyrrole-2-carboxamide (S34-1031). According to the general procedure described above, compound **S34-1031** was synthesized as a white solid with a yield of 58%, m.p. 128-130 °C. ^{1}H NMR (300 MHz, DMSO) δ 12.13 (s, 1H), 8.90 (t, *J* = 6.1 Hz, 1H), 7.80 (d, *J* = 8.2 Hz, 2H), 7.62 (d, *J* = 1.5 Hz, 1H), 7.48 (d, *J* = 8.0 Hz, 2H), 7.27 (d, *J* = 1.6 Hz, 1H), 4.50 (d, *J* = 6.0 Hz, 2H), 2.76 (q, *J* = 7.2 Hz, 2H), 1.04 (t, *J* = 7.3 Hz, 3H). ^{13}C NMR (75 MHz, DMSO) δ 196.0, 160.9, 146.1, 132.8, 128.4, 127.8, 126.8, 125.8, 119.4, 110.0, 110.0, 42.2, 40.8, 40.5, 40.2, 40.0, 39.7, 39.4, 39.1, 32.2, 9.0. HPLC (50% acetonitrile in water): t_R = 4.833 min, 100.000%. ESI-HRMS (*m/z*): [M + H]$^+$ calcd for $C_{16}H_{15}N_3O_2$: 282.1237, found 282.1236.

4-butyryl-N-(4-cyanobenzyl)-1H-pyrrole-2-carboxamide (S34-1032). According to the general procedure described above, compound **S34-1032** was synthesized as a white solid with a yield of 66%, m.p. 127-129 °C. ^{1}H NMR (300 MHz, DMSO) δ 12.13 (s, 1H), 8.89 (t, *J* = 6.1 Hz, 1H), 7.80 (d, *J* = 7.9 Hz, 2H), 7.63 (d, *J* = 1.5 Hz, 1H), 7.48 (d, *J* = 7.9 Hz, 2H), 7.27 (d, *J* = 1.6 Hz, 1H), 2.70 (t, *J* = 7.2 Hz, 2H), 1.68 – 1.50 (m, 2H), 0.89 (t, *J* = 7.4 Hz, 3H). ^{13}C NMR (75 MHz, DMSO) δ 195.6, 160.9, 146.1, 132.8, 128.4, 127.9, 126.9, 126.2, 119.4, 110.1, 110.0, 42.2, 40.8, 40.5, 40.2, 40.0, 39.7, 39.4, 39.1, 18.3, 14.2. HPLC (50% acetonitrile in water): t_R = 5.833 min, 99.613%. ESI-HRMS (*m/z*): [M + H]$^+$ calcd for $C_{17}H_{17}N_3O_2$: 296.1394, found 296.1390.

N-(4-cyanobenzyl)-4-isobutyryl-1H-pyrrole-2-carboxamide (S34-1033). According to the general procedure described above, compound **S34-1033** was synthesized as a white solid with a yield of 65%, m.p. 126-128 °C.^{1}H NMR (300 MHz, DMSO-d_6) δ 9.44 (t, *J* = 5.9 Hz, 1H), 8.06 (d, *J*

= 7.9 Hz, 2H), 7.91 (s, 1H), 7.76 (d, *J* = 7.9 Hz, 2H), 7.59 (s, 1H), 4.76 (d, *J* = 6.0 Hz, 2H), 1.32 (d, *J* = 6.8 Hz, 7H). ^{13}C NMR (101 MHz, DMSO) δ 199.5, 160.8, 146.2, 132.7, 128.5, 128.1, 126.8, 124.8, 119.4, 110.9, 109.9, 42.2, 40.6, 40.4, 40.1, 39.9, 39.7, 39.5, 39.3, 36.4, 19.9. HPLC (50% acetonitrile in water): t_R = 5.788 min, 99.101%. ESI-HRMS (*m/z*): [M + H]$^+$ calcd for $C_{17}H_{17}N_3O_2$: 296.1394, found 296.1389.

4-benzoyl-N-(4-cyanobenzyl)-1H-pyrrole-2-carboxamide (S34-1034). According to the general procedure described above, compound **S34-1034** was synthesized as a white solid with a yield of 76%, m.p. 130-132 °C. ^{1}H NMR (300 MHz, DMSO) δ 12.35 (s, 1H), 9.02 (t, *J* = 6.1 Hz, 1H), 7.80 (t, *J* = 7.6 Hz, 4H), 7.61 (d, *J* = 7.1 Hz, 1H), 7.52 (dd, *J* = 14.8, 7.6 Hz, 4H), 7.44 (s, 1H), 7.39 (s, 1H), 4.53 (d, *J* = 6.0 Hz, 2H). ^{13}C NMR (126 MHz, DMSO) δ 189.8, 160.7, 146.1, 139.6, 132.8, 132.2, 129.0, 128.9, 128.5, 128.3, 128.0, 124.5, 119.4, 112.1, 110.0, 42.3, 40.5, 40.3, 40.1, 40.0, 39.8, 39.6, 39.5. HPLC (50% acetonitrile in water): t_R = 7.211 min, 97.724%. ESI-HRMS (*m/z*): [M + H]$^+$ calcd for $C_{20}H_{15}N_3O_2$: 330.1237, found 330.1231.

4-(cyclopropanecarbonyl)-N-(4-methylbenzyl)-1H-pyrrole-2-carboxamide(S34-1035). According to the general procedure described above, compound **S34-1035** was synthesized as a white solid with a yield of 76%, m.p. 135-137 °C. ^{1}H NMR (300 MHz, DMSO-d_6) δ 12.20 (s, 1H), 8.95 (t, *J* = 6.1 Hz, 1H), 7.80 (dd, *J* = 6.3, 2.0 Hz, 3H), 7.49 (d, *J* = 8.0 Hz, 2H), 7.30 (s, 1H), 4.51 (d, *J* = 6.0 Hz, 2H), 0.89 (ddd, *J* = 10.9, 6.2, 2.6 Hz, 4H). ^{13}C NMR (75 MHz, DMSO) δ 194.8, 160.9, 146.1, 132.8, 128.5, 128.0, 127.1, 126.8, 119.4, 110.0, 109.9, 42.2, 40.8, 40.5, 40.2, 40.0, 39.7, 39.4, 39.1, 17.6, 10.3. HPLC (50% acetonitrile in water): t_R = 4.986 min, 100.000%. ESI-HRMS (*m/z*): [M + H]$^+$ calcd for $C_{17}H_{15}N_3O_2$: 294.1237, found 294.1234.

N-(4-cyanobenzyl)-4-(cyclobutanecarbonyl)-1H-pyrrole-2-carboxamide(S34-1036). According to the general procedure described above, compound **S34-1036** was synthesized as a white solid with a yield of 75%, m.p. 136-138 °C. ^{1}H NMR (300 MHz, DMSO-d_6) δ 12.15 (s, 1H), 8.92 (t, *J* = 6.39 Hz, 1H), 7.82 (d, *J* = 8.0 Hz, 2H), 7.55 – 7.48 (m, 3H), 7.27 (d, *J* = 1.5 Hz, 1H), 4.52 (d, *J* = 6.0 Hz, 2H), 2.20 (q, *J* = 9.2 Hz, 5H), 1.99 (d, *J* = 9.1 Hz, 1H), 1.78 (d, *J* = 9.8 Hz, 1H). ^{13}C NMR (126 MHz, DMSO) δ 196.2, 160.8, 146.1, 132.7, 128.5, 127.9, 126.7, 124.3, 119.4, 110.3, 110.0, 42.4, 42.2, 40.5, 40.3, 40.2, 40.0, 39.8, 39.7, 39.5, 24.9, 18.2. HPLC (50% acetonitrile in water): t_R = 6.327 min, 97.518%. ESI-HRMS (*m/z*): [M + H]$^+$ calcd for $C_{18}H_{17}N_3O_2$: 308.1394, found 308.1391.

4-acetyl-N-(4-cyanobenzyl)-1-methyl-1H-pyrrole-2-carboxamide (S34-1037). According to the general procedure described above, compound **S34-1037** was synthesized as a white solid with a yield of 55%, m.p. 135-137 °C. ^{1}H NMR (300 MHz, DMSO-d_6) δ 8.89 (t, *J* = 6.0 Hz, 1H), 7.84 – 7.71 (m, 3H), 7.48 (d, *J* = 7.9 Hz, 2H), 7.29 (d, *J* = 1.8 Hz, 1H), 4.46 (d, *J* = 6.0 Hz, 2H), 3.87 (s, 3H), 2.32 (s, 3H). ^{13}C NMR (75 MHz, DMSO) δ 192.4, 161.4, 146.1, 133.1, 132.8, 128.4, 127.2, 123.8, 119.4, 112.6, 109.9, 42.2, 40.8, 40.5, 40.2, 40.0, 39.7, 39.4, 39.1, 37.4, 27.4. HPLC (50%

acetonitrile in water): t_R = 4.918 min, 100.000%. ESI-HRMS (*m/z*): $[M + H]^+$ calcd for $C_{16}H_{15}N_3O$: 282.1237, found 282.1246.

N-(4-cyanophenethyl)-4-(cyclopropanecarbonyl)-1H-pyrrole-2-carboxamide(S34-1038). According to the general procedure described above, compound **S34-1038** was synthesized as a white solid with a yield of 58%, m.p. 131-133 °C. ^{1}H NMR (400 MHz, DMSO) δ 12.08 (s, 1H), 8.35 (t, *J* = 5.7 Hz, 1H), 7.85 – 7.68 (m, 3H), 7.54 – 7.35 (m, 2H), 7.18 (d, *J* = 1.6 Hz, 1H), 3.48 (q, *J* = 6.7 Hz, 2H), 2.91 (t, *J* = 6.9 Hz, 2H), 2.60 – 2.53 (m, 1H), 0.95 – 0.78 (m, 4H). ^{13}C NMR (101 MHz, DMSO) δ 193.0, 163.1, 163.0, 161.7, 161.6, 160.7, 159.3, 159.1, 131.4, 131.3, 131.3, 131.2, 127.9, 127.4, 126.4, 123.1, 123.0, 122.9, 122.9, 111.9, 111.9, 111.7, 111.6, 110.0, 104.4, 104.1, 103.9, 40.6, 40.4, 40.2, 40.0, 39.7, 39.5, 39.3, 36.0, 35.9, 27.5. HPLC (50% acetonitrile in water): t_R = 5.364 min, 99.574%. ESI-HRMS (*m/z*): $[M + H]^+$ calcd for $C_{16}H_{15}N_6O$: 308.1394, found 308.1394.

N-(4-cyanobenzyl)-4-(cyclopropanecarbonyl)-N-methyl-1H-pyrrole-2-carboxamide (S34-1040). According to the general procedure described above, compound **S34-1040** was synthesized as a white solid with a yield of 72%, m.p. 135-137 °C.^{1}H NMR (300 MHz, DMSO-*d*$_6$) δ 12.21 (s, 1H), 7.89 – 7.79 (m, 4H), 7.46 (d, *J* = 8.2 Hz, 2H), 4.81 (s, 2H), 3.21 (s, 3H), 0.89 (d, *J* = 5.9 Hz, 3H). ^{13}C NMR (101 MHz, DMSO) δ 194.9, 144.1, 133.0, 128.6, 127.0, 126.7, 126.3, 119.3, 110.4, 40.6, 40.4, 40.2, 40.0, 39.8, 39.5, 39.3, 17.6, 10.3. HPLC (50% acetonitrile in water): t_R = 5.771 min, 98.326%. ESI-HRMS (*m/z*): $[M + H]^+$ calcd for $C_{18}H_{17}N_3O_2$: 308.1394, found 308.1395.
