# Supplementary material for: Target-driven machine learning–enabled virtual screening (TAME-VS) enables prioritization of AKR1C3 inhibitors
Source: Commun Chem. 2026 Jun 15;9:311. doi: 10.1038/s42004-026-02092-6 (PMC13631243; doi:10.1038/s42004-026-02092-6)
Supplement: Supplementary file 4 [file 42004_2026_2092_MOESM3_ESM.pdf]

Description of Additional Supplementary Files:

Supplementary Data 1: Pairwise statistical comparisons of molecular fingerprint types across classification metrics with Holm– Bonferroni correction.

Supplementary Data 2: Spectroscopic characterization of 41 synthesized compounds (^{1}H NMR, ^{13}C NMR, HPLC, and HRMS).

Supplementary Data 3: Source data for Supplementary Figure 6A-D, containing RMSD data from three independent replicates for S34-1035 and S34-1040.

Supplementary Data 4: Source data for Figure 5D and Figure 5H, containing RMSF data from three independent replicates for S34-1035 and S34-1040.

Supplementary Data 5: Source data for Figure 5C and Figure 5G, containing interaction fraction data from three independent replicates for S34-1035 and S34-1040.

Supplementary Data 6: Source data for Figure 5I–N, containing cell viability data from MCF-7 and MCF-7/DOX cells and cell viability measurements from combination treatment of S34-1035 or S34-1040 with DOX at varying concentrations.
