# Supplementary material for: Target-driven machine learning–enabled virtual screening (TAME-VS) enables prioritization of AKR1C3 inhibitors
Source: Commun Chem. 2026 Jun 15;9:311. doi: 10.1038/s42004-026-02092-6 (PMC13631243; doi:10.1038/s42004-026-02092-6)
Supplement: Supplementary file 5 [file 42004_2026_2092_MOESM4_ESM.zip › Supplementary_data_1_Pairwise_fingerprint_comparisons_for_all_metrics.docx]

This file serves as supplementary statistical data in support of **Supplementary Tables 3 and 4**, provided in response to **Reviewer #1 Comment #3**. Following the methodology recommended by Nicholls (*J Comput Aided Mol Des* 30:103–126, 2016; *J Comput Aided Mol Des* 28:887–918, 2014), we performed rigorous pairwise comparisons of seven classification metrics — AUC, Precision, Recall, Specificity, F1 Score, Geometric Mean, and Index of Balanced Accuracy (IBA) — across four molecular fingerprint types (Morgan, Atompair, Topological, and MACCS) for both Random Forest (RF) and Multilayer Perceptron (MLP) models trained under three strategies. Family-Wise Error Rate (FWER) was controlled using Holm–Bonferroni step-down correction (α = 0.05) across six pairwise comparisons per strategy. Effect size is reported as Cohen’s d and probability of superiority P(A > B). **Across all 14 tables, no statistically significant differences were detected between any fingerprint pair after multiple-comparison correction, indicating that fingerprint choice does not significantly affect classification performance within each training strategy.**

**Response Table 1. Holm–Bonferroni step-down correction pairwise comparisons for AUC of trained RF models under different fingerprint types.**

| Strategy | Metric | FP_A | FP_B | Mean_A | Mean_B | Δ(A-B) | t | df | p_value | Cohen's_d | P(A>B) | HB_threshold | Significant | Effect_size |
| --- | --- | --- | --- | --- | --- | --- | --- | --- | --- | --- | --- | --- | --- | --- |
| Strategy #1 | AUC | Atompair | MACCS | 0.914 | 0.926 | -0.012 | -0.785 | 12.6 | 0.4469 | -0.351 | 0.588 | 0.008 | FALSE | small |
| Strategy #1 | AUC | Morgan | Atompair | 0.924 | 0.914 | 0.01 | 0.606 | 15.3 | 0.5532 | 0.271 | 0.568 | 0.01 | FALSE | small |
| Strategy #1 | AUC | Atompair | Topological | 0.914 | 0.921 | -0.007 | -0.394 | 17.1 | 0.6986 | -0.176 | 0.544 | 0.0125 | FALSE | negligible |
| Strategy #1 | AUC | Topological | MACCS | 0.921 | 0.926 | -0.005 | -0.392 | 14.3 | 0.7007 | -0.175 | 0.544 | 0.016 | FALSE | negligible |
| Strategy #1 | AUC | Morgan | Topological | 0.924 | 0.921 | 0.003 | 0.212 | 17.2 | 0.8349 | 0.095 | 0.524 | 0.025 | FALSE | negligible |
| Strategy #1 | AUC | Morgan | MACCS | 0.924 | 0.926 | -0.002 | -0.184 | 16.3 | 0.8564 | -0.082 | 0.521 | 0.05 | FALSE | negligible |
| Strategy #2 | AUC | Morgan | MACCS | 0.945 | 0.926 | 0.019 | 1.633 | 17.9 | 0.12 | 0.73 | 0.683 | 0.008 | FALSE | medium |
| Strategy #2 | AUC | Topological | MACCS | 0.942 | 0.926 | 0.016 | 1.559 | 15.7 | 0.1389 | 0.697 | 0.674 | 0.01 | FALSE | medium |
| Strategy #2 | AUC | Morgan | Atompair | 0.945 | 0.934 | 0.011 | 1.024 | 17.9 | 0.3195 | 0.458 | 0.614 | 0.0125 | FALSE | small |
| Strategy #2 | AUC | Atompair | Topological | 0.934 | 0.942 | -0.008 | -0.866 | 17 | 0.3984 | -0.387 | 0.597 | 0.016 | FALSE | small |
| Strategy #2 | AUC | Atompair | MACCS | 0.934 | 0.926 | 0.008 | 0.713 | 17.6 | 0.4851 | 0.319 | 0.58 | 0.025 | FALSE | small |
| Strategy #2 | AUC | Morgan | Topological | 0.945 | 0.942 | 0.003 | 0.308 | 16.4 | 0.762 | 0.138 | 0.534 | 0.05 | FALSE | negligible |
| Strategy #3 | AUC | Topological | MACCS | 0.967 | 0.965 | 0.002 | 0.335 | 15.1 | 0.7421 | 0.15 | 0.537 | 0.008 | FALSE | negligible |
| Strategy #3 | AUC | Atompair | Topological | 0.965 | 0.967 | -0.002 | -0.297 | 17.7 | 0.7696 | -0.133 | 0.533 | 0.01 | FALSE | negligible |
| Strategy #3 | AUC | Morgan | MACCS | 0.966 | 0.965 | 0.001 | 0.175 | 15.7 | 0.863 | 0.078 | 0.52 | 0.0125 | FALSE | negligible |
| Strategy #3 | AUC | Morgan | Atompair | 0.966 | 0.965 | 0.001 | 0.154 | 17.9 | 0.8792 | 0.069 | 0.517 | 0.016 | FALSE | negligible |
| Strategy #3 | AUC | Morgan | Topological | 0.966 | 0.967 | -0.001 | -0.144 | 17.9 | 0.887 | -0.064 | 0.516 | 0.025 | FALSE | negligible |
| Strategy #3 | AUC | Atompair | MACCS | 0.965 | 0.965 | 0 | 0 | 16.3 | 1 | 0 | 0.5 | 0.05 | FALSE | negligible |

**Response Table 2. Holm–Bonferroni step-down correction pairwise comparisons for Precision of trained RF models under different fingerprint types.**

| Strategy | Metric | FP_A | FP_B | Mean_A | Mean_B | Δ(A-B) | t | df | p_value | Cohen's_d | P(A>B) | HB_threshold | Significant | Effect_size |
| --- | --- | --- | --- | --- | --- | --- | --- | --- | --- | --- | --- | --- | --- | --- |
| Strategy #1 | Precision | Morgan | Atompair | 0.873 | 0.882 | -0.009 | -0.446 | 17.7 | 0.6609 | -0.2 | 0.55 | 0.008 | FALSE | small |
| Strategy #1 | Precision | Atompair | MACCS | 0.882 | 0.877 | 0.005 | 0.279 | 17.8 | 0.7833 | 0.125 | 0.531 | 0.01 | FALSE | negligible |
| Strategy #1 | Precision | Atompair | Topological | 0.882 | 0.877 | 0.005 | 0.269 | 18 | 0.7907 | 0.12 | 0.53 | 0.0125 | FALSE | negligible |
| Strategy #1 | Precision | Morgan | MACCS | 0.873 | 0.877 | -0.004 | -0.207 | 17.1 | 0.8388 | -0.092 | 0.523 | 0.016 | FALSE | negligible |
| Strategy #1 | Precision | Morgan | Topological | 0.873 | 0.877 | -0.004 | -0.2 | 17.6 | 0.8435 | -0.09 | 0.522 | 0.025 | FALSE | negligible |
| Strategy #1 | Precision | Topological | MACCS | 0.877 | 0.877 | 0 | 0 | 17.9 | 1 | 0 | 0.5 | 0.05 | FALSE | negligible |
| Strategy #2 | Precision | Morgan | MACCS | 0.906 | 0.882 | 0.024 | 2.544 | 17.4 | 0.0207 | 1.138 | 0.784 | 0.008 | FALSE | large |
| Strategy #2 | Precision | Topological | MACCS | 0.898 | 0.882 | 0.016 | 1.879 | 14.9 | 0.08 | 0.84 | 0.71 | 0.01 | FALSE | large |
| Strategy #2 | Precision | Morgan | Atompair | 0.906 | 0.888 | 0.018 | 1.86 | 17.1 | 0.0802 | 0.832 | 0.708 | 0.0125 | FALSE | large |
| Strategy #2 | Precision | Atompair | Topological | 0.888 | 0.898 | -0.01 | -1.138 | 14.5 | 0.2735 | -0.509 | 0.627 | 0.016 | FALSE | medium |
| Strategy #2 | Precision | Morgan | Topological | 0.906 | 0.898 | 0.008 | 1.072 | 16.5 | 0.2991 | 0.479 | 0.62 | 0.025 | FALSE | small |
| Strategy #2 | Precision | Atompair | MACCS | 0.888 | 0.882 | 0.006 | 0.571 | 18 | 0.5752 | 0.255 | 0.564 | 0.05 | FALSE | small |
| Strategy #3 | Precision | Topological | MACCS | 0.926 | 0.904 | 0.022 | 2.319 | 16.7 | 0.0334 | 1.037 | 0.759 | 0.008 | FALSE | large |
| Strategy #3 | Precision | Atompair | MACCS | 0.918 | 0.904 | 0.014 | 1.557 | 17.3 | 0.1374 | 0.697 | 0.674 | 0.01 | FALSE | medium |
| Strategy #3 | Precision | Morgan | MACCS | 0.919 | 0.904 | 0.015 | 1.128 | 12.8 | 0.2799 | 0.505 | 0.626 | 0.0125 | FALSE | medium |
| Strategy #3 | Precision | Atompair | Topological | 0.918 | 0.926 | -0.008 | -0.777 | 17.9 | 0.4473 | -0.347 | 0.587 | 0.016 | FALSE | small |
| Strategy #3 | Precision | Morgan | Topological | 0.919 | 0.926 | -0.007 | -0.493 | 15.2 | 0.6294 | -0.22 | 0.555 | 0.025 | FALSE | small |
| Strategy #3 | Precision | Morgan | Atompair | 0.919 | 0.918 | 0.001 | 0.072 | 14.4 | 0.9436 | 0.032 | 0.508 | 0.05 | FALSE | negligible |

**Response Table 3. Holm–Bonferroni step-down correction pairwise comparisons for Recall of trained RF models under different fingerprint types.**

| Strategy | Metric | FP_A | FP_B | Mean_A | Mean_B | Δ(A-B) | t | df | p_value | Cohen's_d | P(A>B) | HB_threshold | Significant | Effect_size |
| --- | --- | --- | --- | --- | --- | --- | --- | --- | --- | --- | --- | --- | --- | --- |
| Strategy #1 | Recall | Morgan | MACCS | 0.838 | 0.854 | -0.016 | -0.603 | 17.3 | 0.5542 | -0.27 | 0.567 | 0.008 | FALSE | small |
| Strategy #1 | Recall | Morgan | Atompair | 0.838 | 0.851 | -0.013 | -0.479 | 17.6 | 0.6377 | -0.214 | 0.554 | 0.01 | FALSE | small |
| Strategy #1 | Recall | Morgan | Topological | 0.838 | 0.848 | -0.01 | -0.386 | 16.9 | 0.7046 | -0.172 | 0.543 | 0.0125 | FALSE | negligible |
| Strategy #1 | Recall | Topological | MACCS | 0.848 | 0.854 | -0.006 | -0.26 | 17.9 | 0.7975 | -0.116 | 0.529 | 0.016 | FALSE | negligible |
| Strategy #1 | Recall | Atompair | Topological | 0.851 | 0.848 | 0.003 | 0.126 | 17.8 | 0.9009 | 0.057 | 0.514 | 0.025 | FALSE | negligible |
| Strategy #1 | Recall | Atompair | MACCS | 0.851 | 0.854 | -0.003 | -0.123 | 17.9 | 0.9034 | -0.055 | 0.514 | 0.05 | FALSE | negligible |
| Strategy #2 | Recall | Morgan | MACCS | 0.884 | 0.856 | 0.028 | 2.555 | 18 | 0.0199 | 1.143 | 0.786 | 0.008 | FALSE | large |
| Strategy #2 | Recall | Morgan | Atompair | 0.884 | 0.862 | 0.022 | 2.007 | 18 | 0.06 | 0.898 | 0.724 | 0.01 | FALSE | large |
| Strategy #2 | Recall | Topological | MACCS | 0.873 | 0.856 | 0.017 | 1.521 | 18 | 0.1458 | 0.68 | 0.67 | 0.0125 | FALSE | medium |
| Strategy #2 | Recall | Morgan | Topological | 0.884 | 0.873 | 0.011 | 1.004 | 18 | 0.3288 | 0.449 | 0.612 | 0.016 | FALSE | small |
| Strategy #2 | Recall | Atompair | Topological | 0.862 | 0.873 | -0.011 | -0.984 | 18 | 0.3382 | -0.44 | 0.61 | 0.025 | FALSE | small |
| Strategy #2 | Recall | Atompair | MACCS | 0.862 | 0.856 | 0.006 | 0.537 | 18 | 0.5981 | 0.24 | 0.56 | 0.05 | FALSE | small |
| Strategy #3 | Recall | Topological | MACCS | 0.923 | 0.902 | 0.021 | 2.115 | 16.8 | 0.0497 | 0.946 | 0.736 | 0.008 | FALSE | large |
| Strategy #3 | Recall | Atompair | MACCS | 0.916 | 0.902 | 0.014 | 1.523 | 17.6 | 0.1455 | 0.681 | 0.67 | 0.01 | FALSE | medium |
| Strategy #3 | Recall | Morgan | MACCS | 0.916 | 0.902 | 0.014 | 1 | 12.9 | 0.3359 | 0.447 | 0.612 | 0.0125 | FALSE | small |
| Strategy #3 | Recall | Atompair | Topological | 0.916 | 0.923 | -0.007 | -0.665 | 17.7 | 0.5148 | -0.297 | 0.574 | 0.016 | FALSE | small |
| Strategy #3 | Recall | Morgan | Topological | 0.916 | 0.923 | -0.007 | -0.469 | 15.1 | 0.6456 | -0.21 | 0.552 | 0.025 | FALSE | small |
| Strategy #3 | Recall | Morgan | Atompair | 0.916 | 0.916 | 0 | 0 | 14 | 1 | 0 | 0.5 | 0.05 | FALSE | negligible |

**Response Table 4. Holm–Bonferroni step-down correction pairwise comparisons for Specificity of trained RF models under different fingerprint types.**

| Strategy | Metric | FP_A | FP_B | Mean_A | Mean_B | Δ(A-B) | t | df | p_value | Cohen's_d | P(A>B) | HB_threshold | Significant | Effect_size |
| --- | --- | --- | --- | --- | --- | --- | --- | --- | --- | --- | --- | --- | --- | --- |
| Strategy #1 | Specificity | Atompair | MACCS | 0.887 | 0.868 | 0.019 | 0.942 | 17.7 | 0.3589 | 0.421 | 0.605 | 0.008 | FALSE | small |
| Strategy #1 | Specificity | Topological | MACCS | 0.88 | 0.868 | 0.012 | 0.616 | 17.9 | 0.5453 | 0.276 | 0.569 | 0.01 | FALSE | small |
| Strategy #1 | Specificity | Morgan | Atompair | 0.876 | 0.887 | -0.011 | -0.477 | 17.7 | 0.6395 | -0.213 | 0.553 | 0.0125 | FALSE | small |
| Strategy #1 | Specificity | Morgan | MACCS | 0.876 | 0.868 | 0.008 | 0.366 | 16.8 | 0.7192 | 0.163 | 0.541 | 0.016 | FALSE | negligible |
| Strategy #1 | Specificity | Atompair | Topological | 0.887 | 0.88 | 0.007 | 0.336 | 17.9 | 0.7404 | 0.15 | 0.538 | 0.025 | FALSE | negligible |
| Strategy #1 | Specificity | Morgan | Topological | 0.876 | 0.88 | -0.004 | -0.178 | 17.3 | 0.8608 | -0.08 | 0.52 | 0.05 | FALSE | negligible |
| Strategy #2 | Specificity | Morgan | MACCS | 0.901 | 0.854 | 0.047 | 2.113 | 14.3 | 0.0525 | 0.945 | 0.736 | 0.008 | FALSE | large |
| Strategy #2 | Specificity | Topological | MACCS | 0.887 | 0.854 | 0.033 | 1.505 | 13.9 | 0.1549 | 0.673 | 0.668 | 0.01 | FALSE | medium |
| Strategy #2 | Specificity | Morgan | Atompair | 0.901 | 0.87 | 0.031 | 1.429 | 14.6 | 0.174 | 0.639 | 0.66 | 0.0125 | FALSE | medium |
| Strategy #2 | Specificity | Morgan | Topological | 0.901 | 0.887 | 0.014 | 0.92 | 17.9 | 0.3696 | 0.412 | 0.603 | 0.016 | FALSE | small |
| Strategy #2 | Specificity | Atompair | Topological | 0.87 | 0.887 | -0.017 | -0.795 | 14.1 | 0.4396 | -0.356 | 0.589 | 0.025 | FALSE | small |
| Strategy #2 | Specificity | Atompair | MACCS | 0.87 | 0.854 | 0.016 | 0.596 | 18 | 0.5585 | 0.267 | 0.567 | 0.05 | FALSE | small |
| Strategy #3 | Specificity | Topological | MACCS | 0.925 | 0.897 | 0.028 | 2.659 | 17.7 | 0.0161 | 1.189 | 0.797 | 0.008 | FALSE | large |
| Strategy #3 | Specificity | Atompair | Topological | 0.909 | 0.925 | -0.016 | -1.431 | 18 | 0.1695 | -0.64 | 0.66 | 0.01 | FALSE | medium |
| Strategy #3 | Specificity | Atompair | MACCS | 0.909 | 0.897 | 0.012 | 1.14 | 17.7 | 0.2697 | 0.51 | 0.627 | 0.0125 | FALSE | medium |
| Strategy #3 | Specificity | Morgan | MACCS | 0.913 | 0.897 | 0.016 | 1.13 | 14.2 | 0.2772 | 0.505 | 0.626 | 0.016 | FALSE | medium |
| Strategy #3 | Specificity | Morgan | Topological | 0.913 | 0.925 | -0.012 | -0.819 | 15.3 | 0.4253 | -0.366 | 0.592 | 0.025 | FALSE | small |
| Strategy #3 | Specificity | Morgan | Atompair | 0.913 | 0.909 | 0.004 | 0.273 | 15.3 | 0.7885 | 0.122 | 0.531 | 0.05 | FALSE | negligible |

**Response Table 5. Holm–Bonferroni step-down correction pairwise comparisons for F1 Score of trained RF models under different fingerprint types.**

| Strategy | Metric | FP_A | FP_B | Mean_A | Mean_B | Δ(A-B) | t | df | p_value | Cohen's_d | P(A>B) | HB_threshold | Significant | Effect_size |
| --- | --- | --- | --- | --- | --- | --- | --- | --- | --- | --- | --- | --- | --- | --- |
| Strategy #1 | F1 | Morgan | MACCS | 0.842 | 0.857 | -0.015 | -0.585 | 17.3 | 0.566 | -0.262 | 0.565 | 0.008 | FALSE | small |
| Strategy #1 | F1 | Morgan | Atompair | 0.842 | 0.855 | -0.013 | -0.495 | 17.6 | 0.6264 | -0.222 | 0.555 | 0.01 | FALSE | small |
| Strategy #1 | F1 | Morgan | Topological | 0.842 | 0.851 | -0.009 | -0.359 | 16.8 | 0.7238 | -0.161 | 0.54 | 0.0125 | FALSE | negligible |
| Strategy #1 | F1 | Topological | MACCS | 0.851 | 0.857 | -0.006 | -0.271 | 17.9 | 0.7896 | -0.121 | 0.53 | 0.016 | FALSE | negligible |
| Strategy #1 | F1 | Atompair | Topological | 0.855 | 0.851 | 0.004 | 0.175 | 17.8 | 0.863 | 0.078 | 0.52 | 0.025 | FALSE | negligible |
| Strategy #1 | F1 | Atompair | MACCS | 0.855 | 0.857 | -0.002 | -0.085 | 17.9 | 0.9331 | -0.038 | 0.51 | 0.05 | FALSE | negligible |
| Strategy #2 | F1 | Morgan | MACCS | 0.889 | 0.862 | 0.027 | 2.683 | 18 | 0.0152 | 1.2 | 0.8 | 0.008 | FALSE | large |
| Strategy #2 | F1 | Morgan | Atompair | 0.889 | 0.869 | 0.02 | 1.943 | 17.9 | 0.068 | 0.869 | 0.717 | 0.01 | FALSE | large |
| Strategy #2 | F1 | Topological | MACCS | 0.879 | 0.862 | 0.017 | 1.689 | 18 | 0.1085 | 0.755 | 0.689 | 0.0125 | FALSE | medium |
| Strategy #2 | F1 | Morgan | Topological | 0.889 | 0.879 | 0.01 | 1.016 | 18 | 0.3229 | 0.455 | 0.614 | 0.016 | FALSE | small |
| Strategy #2 | F1 | Atompair | Topological | 0.869 | 0.879 | -0.01 | -0.971 | 17.9 | 0.3444 | -0.434 | 0.609 | 0.025 | FALSE | small |
| Strategy #2 | F1 | Atompair | MACCS | 0.869 | 0.862 | 0.007 | 0.666 | 18 | 0.5139 | 0.298 | 0.574 | 0.05 | FALSE | small |
| Strategy #3 | F1 | Topological | MACCS | 0.923 | 0.902 | 0.021 | 2.169 | 17.1 | 0.0444 | 0.97 | 0.743 | 0.008 | FALSE | large |
| Strategy #3 | F1 | Atompair | MACCS | 0.916 | 0.902 | 0.014 | 1.523 | 17.6 | 0.1455 | 0.681 | 0.67 | 0.01 | FALSE | medium |
| Strategy #3 | F1 | Morgan | MACCS | 0.916 | 0.902 | 0.014 | 1.021 | 13 | 0.326 | 0.456 | 0.614 | 0.0125 | FALSE | small |
| Strategy #3 | F1 | Atompair | Topological | 0.916 | 0.923 | -0.007 | -0.68 | 17.9 | 0.5053 | -0.304 | 0.576 | 0.016 | FALSE | small |
| Strategy #3 | F1 | Morgan | Topological | 0.916 | 0.923 | -0.007 | -0.483 | 15 | 0.6358 | -0.216 | 0.554 | 0.025 | FALSE | small |
| Strategy #3 | F1 | Morgan | Atompair | 0.916 | 0.916 | 0 | 0 | 14.2 | 1 | 0 | 0.5 | 0.05 | FALSE | negligible |

**Response Table 6. Holm–Bonferroni step-down correction pairwise comparisons for Geometric Mean of trained RF models under different fingerprint types.**

| Strategy | Metric | FP_A | FP_B | Mean_A | Mean_B | Δ(A-B) | t | df | p_value | Cohen's_d | P(A>B) | HB_threshold | Significant | Effect_size |
| --- | --- | --- | --- | --- | --- | --- | --- | --- | --- | --- | --- | --- | --- | --- |
| Strategy #1 | GM | Morgan | Atompair | 0.852 | 0.865 | -0.013 | -0.562 | 17.5 | 0.5811 | -0.251 | 0.563 | 0.008 | FALSE | small |
| Strategy #1 | GM | Atompair | MACCS | 0.865 | 0.857 | 0.008 | 0.406 | 17.7 | 0.6899 | 0.181 | 0.545 | 0.01 | FALSE | negligible |
| Strategy #1 | GM | Morgan | Topological | 0.852 | 0.861 | -0.009 | -0.403 | 16.9 | 0.6919 | -0.18 | 0.545 | 0.0125 | FALSE | negligible |
| Strategy #1 | GM | Morgan | MACCS | 0.852 | 0.857 | -0.005 | -0.228 | 16.5 | 0.8226 | -0.102 | 0.525 | 0.016 | FALSE | negligible |
| Strategy #1 | GM | Topological | MACCS | 0.861 | 0.857 | 0.004 | 0.213 | 18 | 0.8338 | 0.095 | 0.524 | 0.025 | FALSE | negligible |
| Strategy #1 | GM | Atompair | Topological | 0.865 | 0.861 | 0.004 | 0.199 | 17.9 | 0.8448 | 0.089 | 0.522 | 0.05 | FALSE | negligible |
| Strategy #2 | GM | Morgan | MACCS | 0.892 | 0.853 | 0.039 | 2.679 | 15.9 | 0.0165 | 1.198 | 0.799 | 0.008 | FALSE | large |
| Strategy #2 | GM | Topological | MACCS | 0.879 | 0.853 | 0.026 | 1.894 | 14 | 0.0791 | 0.847 | 0.712 | 0.01 | FALSE | large |
| Strategy #2 | GM | Morgan | Atompair | 0.892 | 0.865 | 0.027 | 1.854 | 15.9 | 0.0823 | 0.829 | 0.707 | 0.0125 | FALSE | large |
| Strategy #2 | GM | Morgan | Topological | 0.892 | 0.879 | 0.013 | 1.23 | 17.2 | 0.2352 | 0.55 | 0.638 | 0.016 | FALSE | medium |
| Strategy #2 | GM | Atompair | Topological | 0.865 | 0.879 | -0.014 | -1.02 | 14 | 0.3251 | -0.456 | 0.614 | 0.025 | FALSE | small |
| Strategy #2 | GM | Atompair | MACCS | 0.865 | 0.853 | 0.012 | 0.706 | 18 | 0.4891 | 0.316 | 0.579 | 0.05 | FALSE | small |
| Strategy #3 | GM | Topological | MACCS | 0.923 | 0.898 | 0.025 | 2.531 | 17.4 | 0.0213 | 1.132 | 0.783 | 0.008 | FALSE | large |
| Strategy #3 | GM | Atompair | MACCS | 0.911 | 0.898 | 0.013 | 1.316 | 17.4 | 0.2053 | 0.588 | 0.647 | 0.01 | FALSE | medium |
| Strategy #3 | GM | Atompair | Topological | 0.911 | 0.923 | -0.012 | -1.118 | 18 | 0.2783 | -0.5 | 0.625 | 0.0125 | FALSE | medium |
| Strategy #3 | GM | Morgan | MACCS | 0.913 | 0.898 | 0.015 | 1.082 | 13.4 | 0.2982 | 0.484 | 0.621 | 0.016 | FALSE | small |
| Strategy #3 | GM | Morgan | Topological | 0.913 | 0.923 | -0.01 | -0.691 | 15 | 0.5004 | -0.309 | 0.577 | 0.025 | FALSE | small |
| Strategy #3 | GM | Morgan | Atompair | 0.913 | 0.911 | 0.002 | 0.138 | 15 | 0.892 | 0.062 | 0.515 | 0.05 | FALSE | negligible |

**Response Table 7. Holm–Bonferroni step-down correction pairwise comparisons for IBA of trained RF models under different fingerprint types.**

| Strategy | Metric | FP_A | FP_B | Mean_A | Mean_B | Δ(A-B) | t | df | p_value | Cohen's_d | P(A>B) | HB_threshold | Significant | Effect_size |
| --- | --- | --- | --- | --- | --- | --- | --- | --- | --- | --- | --- | --- | --- | --- |
| Strategy #1 | IBA | Morgan | Atompair | 0.726 | 0.747 | -0.021 | -0.517 | 17.5 | 0.6116 | -0.231 | 0.558 | 0.008 | FALSE | small |
| Strategy #1 | IBA | Morgan | Topological | 0.726 | 0.74 | -0.014 | -0.357 | 16.9 | 0.7255 | -0.16 | 0.54 | 0.01 | FALSE | negligible |
| Strategy #1 | IBA | Atompair | MACCS | 0.747 | 0.736 | 0.011 | 0.315 | 17.7 | 0.7567 | 0.141 | 0.535 | 0.0125 | FALSE | negligible |
| Strategy #1 | IBA | Morgan | MACCS | 0.726 | 0.736 | -0.01 | -0.259 | 16.6 | 0.799 | -0.116 | 0.529 | 0.016 | FALSE | negligible |
| Strategy #1 | IBA | Atompair | Topological | 0.747 | 0.74 | 0.007 | 0.197 | 17.9 | 0.8463 | 0.088 | 0.522 | 0.025 | FALSE | negligible |
| Strategy #1 | IBA | Topological | MACCS | 0.74 | 0.736 | 0.004 | 0.12 | 18 | 0.9058 | 0.054 | 0.513 | 0.05 | FALSE | negligible |
| Strategy #2 | IBA | Morgan | MACCS | 0.795 | 0.73 | 0.065 | 2.683 | 16.4 | 0.0161 | 1.2 | 0.8 | 0.008 | FALSE | large |
| Strategy #2 | IBA | Morgan | Atompair | 0.795 | 0.749 | 0.046 | 1.879 | 16.3 | 0.0783 | 0.84 | 0.71 | 0.01 | FALSE | large |
| Strategy #2 | IBA | Topological | MACCS | 0.772 | 0.73 | 0.042 | 1.853 | 14.4 | 0.0845 | 0.828 | 0.707 | 0.0125 | FALSE | large |
| Strategy #2 | IBA | Morgan | Topological | 0.795 | 0.772 | 0.023 | 1.262 | 17.2 | 0.2238 | 0.564 | 0.641 | 0.016 | FALSE | medium |
| Strategy #2 | IBA | Atompair | Topological | 0.749 | 0.772 | -0.023 | -1.002 | 14.3 | 0.3328 | -0.448 | 0.612 | 0.025 | FALSE | small |
| Strategy #2 | IBA | Atompair | MACCS | 0.749 | 0.73 | 0.019 | 0.68 | 18 | 0.5053 | 0.304 | 0.576 | 0.05 | FALSE | small |
| Strategy #3 | IBA | Topological | MACCS | 0.852 | 0.808 | 0.044 | 2.441 | 17 | 0.0259 | 1.092 | 0.773 | 0.008 | FALSE | large |
| Strategy #3 | IBA | Atompair | MACCS | 0.832 | 0.808 | 0.024 | 1.388 | 17.4 | 0.1826 | 0.621 | 0.655 | 0.01 | FALSE | medium |
| Strategy #3 | IBA | Morgan | MACCS | 0.836 | 0.808 | 0.028 | 1.119 | 13.1 | 0.2834 | 0.5 | 0.625 | 0.0125 | FALSE | medium |
| Strategy #3 | IBA | Atompair | Topological | 0.832 | 0.852 | -0.02 | -1.027 | 17.9 | 0.3179 | -0.459 | 0.615 | 0.016 | FALSE | small |
| Strategy #3 | IBA | Morgan | Topological | 0.836 | 0.852 | -0.016 | -0.602 | 15.2 | 0.5561 | -0.269 | 0.567 | 0.025 | FALSE | small |
| Strategy #3 | IBA | Morgan | Atompair | 0.836 | 0.832 | 0.004 | 0.153 | 14.6 | 0.8802 | 0.069 | 0.517 | 0.05 | FALSE | negligible |

**Response Table 8. Holm–Bonferroni step-down correction pairwise comparisons for AUC of trained MLP models under different fingerprint types.**

| Strategy | Metric | FP_A | FP_B | Mean_A | Mean_B | Δ(A-B) | t | df | p_value | Cohen's_d | P(A>B) | HB_threshold | Significant | Effect_size |
| --- | --- | --- | --- | --- | --- | --- | --- | --- | --- | --- | --- | --- | --- | --- |
| Strategy #1 | AUC | Morgan | Topological | 0.913 | 0.891 | 0.022 | 1.562 | 18 | 0.1358 | 0.698 | 0.675 | 0.008 | FALSE | medium |
| Strategy #1 | AUC | Morgan | Atompair | 0.913 | 0.885 | 0.028 | 1.55 | 15.4 | 0.1416 | 0.693 | 0.673 | 0.01 | FALSE | medium |
| Strategy #1 | AUC | Morgan | MACCS | 0.913 | 0.895 | 0.018 | 1.257 | 17.9 | 0.2248 | 0.562 | 0.641 | 0.0125 | FALSE | medium |
| Strategy #1 | AUC | Atompair | MACCS | 0.885 | 0.895 | -0.01 | -0.543 | 16 | 0.5947 | -0.243 | 0.561 | 0.016 | FALSE | small |
| Strategy #1 | AUC | Atompair | Topological | 0.885 | 0.891 | -0.006 | -0.329 | 15.7 | 0.7466 | -0.147 | 0.537 | 0.025 | FALSE | negligible |
| Strategy #1 | AUC | Topological | MACCS | 0.891 | 0.895 | -0.004 | -0.275 | 18 | 0.7863 | -0.123 | 0.531 | 0.05 | FALSE | negligible |
| Strategy #2 | AUC | Topological | MACCS | 0.929 | 0.903 | 0.026 | 2.02 | 13.7 | 0.0635 | 0.903 | 0.726 | 0.008 | FALSE | large |
| Strategy #2 | AUC | Morgan | MACCS | 0.928 | 0.903 | 0.025 | 1.71 | 17.2 | 0.1052 | 0.765 | 0.691 | 0.01 | FALSE | medium |
| Strategy #2 | AUC | Atompair | Topological | 0.914 | 0.929 | -0.015 | -1.473 | 16.5 | 0.1596 | -0.659 | 0.665 | 0.0125 | FALSE | medium |
| Strategy #2 | AUC | Morgan | Atompair | 0.928 | 0.914 | 0.014 | 1.137 | 17.8 | 0.2708 | 0.508 | 0.627 | 0.016 | FALSE | medium |
| Strategy #2 | AUC | Atompair | MACCS | 0.914 | 0.903 | 0.011 | 0.783 | 16.4 | 0.4446 | 0.35 | 0.588 | 0.025 | FALSE | small |
| Strategy #2 | AUC | Morgan | Topological | 0.928 | 0.929 | -0.001 | -0.091 | 15.5 | 0.9285 | -0.041 | 0.51 | 0.05 | FALSE | negligible |
| Strategy #3 | AUC | Atompair | MACCS | 0.96 | 0.944 | 0.016 | 1.653 | 17.1 | 0.1166 | 0.739 | 0.685 | 0.008 | FALSE | medium |
| Strategy #3 | AUC | Topological | MACCS | 0.959 | 0.944 | 0.015 | 1.613 | 16.2 | 0.1261 | 0.721 | 0.68 | 0.01 | FALSE | medium |
| Strategy #3 | AUC | Morgan | MACCS | 0.955 | 0.944 | 0.011 | 1.046 | 18 | 0.3092 | 0.468 | 0.617 | 0.0125 | FALSE | small |
| Strategy #3 | AUC | Morgan | Atompair | 0.955 | 0.96 | -0.005 | -0.53 | 17.4 | 0.6028 | -0.237 | 0.559 | 0.016 | FALSE | small |
| Strategy #3 | AUC | Morgan | Topological | 0.955 | 0.959 | -0.004 | -0.442 | 16.6 | 0.664 | -0.198 | 0.549 | 0.025 | FALSE | negligible |
| Strategy #3 | AUC | Atompair | Topological | 0.96 | 0.959 | 0.001 | 0.124 | 17.8 | 0.9027 | 0.055 | 0.514 | 0.05 | FALSE | negligible |

**Response Table 9. Holm–Bonferroni step-down correction pairwise comparisons for Precision of trained MLP models under different fingerprint types.**

| Strategy | Metric | FP_A | FP_B | Mean_A | Mean_B | Δ(A-B) | t | df | p_value | Cohen's_d | P(A>B) | HB_threshold | Significant | Effect_size |
| --- | --- | --- | --- | --- | --- | --- | --- | --- | --- | --- | --- | --- | --- | --- |
| Strategy #1 | Precision | Atompair | Topological | 0.841 | 0.863 | -0.022 | -1.056 | 17.8 | 0.3049 | -0.472 | 0.618 | 0.008 | FALSE | small |
| Strategy #1 | Precision | Morgan | Atompair | 0.859 | 0.841 | 0.018 | 0.945 | 16.3 | 0.3583 | 0.423 | 0.606 | 0.01 | FALSE | small |
| Strategy #1 | Precision | Atompair | MACCS | 0.841 | 0.86 | -0.019 | -0.832 | 17.9 | 0.4162 | -0.372 | 0.593 | 0.0125 | FALSE | small |
| Strategy #1 | Precision | Morgan | Topological | 0.859 | 0.863 | -0.004 | -0.225 | 17.1 | 0.8247 | -0.101 | 0.525 | 0.016 | FALSE | negligible |
| Strategy #1 | Precision | Topological | MACCS | 0.863 | 0.86 | 0.003 | 0.138 | 17.4 | 0.892 | 0.062 | 0.515 | 0.025 | FALSE | negligible |
| Strategy #1 | Precision | Morgan | MACCS | 0.859 | 0.86 | -0.001 | -0.05 | 15.6 | 0.9609 | -0.022 | 0.506 | 0.05 | FALSE | negligible |
| Strategy #2 | Precision | Topological | MACCS | 0.89 | 0.865 | 0.025 | 2.595 | 12.2 | 0.0232 | 1.161 | 0.79 | 0.008 | FALSE | large |
| Strategy #2 | Precision | Morgan | MACCS | 0.886 | 0.865 | 0.021 | 1.769 | 17.8 | 0.094 | 0.791 | 0.698 | 0.01 | FALSE | medium |
| Strategy #2 | Precision | Atompair | MACCS | 0.881 | 0.865 | 0.016 | 1.396 | 17.3 | 0.1802 | 0.624 | 0.656 | 0.0125 | FALSE | medium |
| Strategy #2 | Precision | Atompair | Topological | 0.881 | 0.89 | -0.009 | -1.097 | 13.6 | 0.2917 | -0.491 | 0.623 | 0.016 | FALSE | small |
| Strategy #2 | Precision | Morgan | Atompair | 0.886 | 0.881 | 0.005 | 0.465 | 17.9 | 0.6472 | 0.208 | 0.552 | 0.025 | FALSE | small |
| Strategy #2 | Precision | Morgan | Topological | 0.886 | 0.89 | -0.004 | -0.456 | 12.9 | 0.6559 | -0.204 | 0.551 | 0.05 | FALSE | small |
| Strategy #3 | Precision | Topological | MACCS | 0.902 | 0.898 | 0.004 | 0.363 | 13.5 | 0.7225 | 0.162 | 0.541 | 0.008 | FALSE | negligible |
| Strategy #3 | Precision | Morgan | Topological | 0.899 | 0.902 | -0.003 | -0.286 | 14 | 0.7787 | -0.128 | 0.532 | 0.01 | FALSE | negligible |
| Strategy #3 | Precision | Atompair | Topological | 0.9 | 0.902 | -0.002 | -0.186 | 13.7 | 0.8552 | -0.083 | 0.521 | 0.0125 | FALSE | negligible |
| Strategy #3 | Precision | Atompair | MACCS | 0.9 | 0.898 | 0.002 | 0.147 | 18 | 0.8851 | 0.066 | 0.516 | 0.016 | FALSE | negligible |
| Strategy #3 | Precision | Morgan | Atompair | 0.899 | 0.9 | -0.001 | -0.076 | 18 | 0.9404 | -0.034 | 0.508 | 0.025 | FALSE | negligible |
| Strategy #3 | Precision | Morgan | MACCS | 0.899 | 0.898 | 0.001 | 0.074 | 17.9 | 0.9414 | 0.033 | 0.508 | 0.05 | FALSE | negligible |

**Response Table 10. Holm–Bonferroni step-down correction pairwise comparisons for Recall of trained MLP models under different fingerprint types.**

| Strategy | Metric | FP_A | FP_B | Mean_A | Mean_B | Δ(A-B) | t | df | p_value | Cohen's_d | P(A>B) | HB_threshold | Significant | Effect_size |
| --- | --- | --- | --- | --- | --- | --- | --- | --- | --- | --- | --- | --- | --- | --- |
| Strategy #1 | Recall | Atompair | MACCS | 0.815 | 0.848 | -0.033 | -1.181 | 18 | 0.2531 | -0.528 | 0.632 | 0.008 | FALSE | medium |
| Strategy #1 | Recall | Atompair | Topological | 0.815 | 0.838 | -0.023 | -0.911 | 17 | 0.3749 | -0.408 | 0.602 | 0.01 | FALSE | small |
| Strategy #1 | Recall | Morgan | Atompair | 0.835 | 0.815 | 0.02 | 0.774 | 17.4 | 0.4492 | 0.346 | 0.587 | 0.0125 | FALSE | small |
| Strategy #1 | Recall | Morgan | MACCS | 0.835 | 0.848 | -0.013 | -0.508 | 17.5 | 0.6178 | -0.227 | 0.557 | 0.016 | FALSE | small |
| Strategy #1 | Recall | Topological | MACCS | 0.838 | 0.848 | -0.01 | -0.4 | 17.1 | 0.694 | -0.179 | 0.545 | 0.025 | FALSE | negligible |
| Strategy #1 | Recall | Morgan | Topological | 0.835 | 0.838 | -0.003 | -0.133 | 17.9 | 0.8958 | -0.059 | 0.515 | 0.05 | FALSE | negligible |
| Strategy #2 | Recall | Morgan | MACCS | 0.866 | 0.839 | 0.027 | 2.012 | 15.9 | 0.0615 | 0.9 | 0.725 | 0.008 | FALSE | large |
| Strategy #2 | Recall | Topological | MACCS | 0.864 | 0.839 | 0.025 | 1.888 | 15.6 | 0.0779 | 0.844 | 0.711 | 0.01 | FALSE | large |
| Strategy #2 | Recall | Morgan | Atompair | 0.866 | 0.853 | 0.013 | 1.049 | 16.9 | 0.3091 | 0.469 | 0.617 | 0.0125 | FALSE | small |
| Strategy #2 | Recall | Atompair | MACCS | 0.853 | 0.839 | 0.014 | 0.947 | 17.7 | 0.3564 | 0.423 | 0.606 | 0.016 | FALSE | small |
| Strategy #2 | Recall | Atompair | Topological | 0.853 | 0.864 | -0.011 | -0.901 | 16.6 | 0.3804 | -0.403 | 0.601 | 0.025 | FALSE | small |
| Strategy #2 | Recall | Morgan | Topological | 0.866 | 0.864 | 0.002 | 0.19 | 18 | 0.8512 | 0.085 | 0.521 | 0.05 | FALSE | negligible |
| Strategy #3 | Recall | Topological | MACCS | 0.9 | 0.895 | 0.005 | 0.436 | 13.7 | 0.6694 | 0.195 | 0.549 | 0.008 | FALSE | negligible |
| Strategy #3 | Recall | Morgan | Topological | 0.897 | 0.9 | -0.003 | -0.29 | 14.8 | 0.7761 | -0.13 | 0.532 | 0.01 | FALSE | negligible |
| Strategy #3 | Recall | Atompair | Topological | 0.897 | 0.9 | -0.003 | -0.262 | 13.7 | 0.7974 | -0.117 | 0.529 | 0.0125 | FALSE | negligible |
| Strategy #3 | Recall | Morgan | MACCS | 0.897 | 0.895 | 0.002 | 0.149 | 17.7 | 0.8834 | 0.067 | 0.517 | 0.016 | FALSE | negligible |
| Strategy #3 | Recall | Atompair | MACCS | 0.897 | 0.895 | 0.002 | 0.14 | 18 | 0.8904 | 0.063 | 0.516 | 0.025 | FALSE | negligible |
| Strategy #3 | Recall | Morgan | Atompair | 0.897 | 0.897 | 0 | 0 | 17.7 | 1 | 0 | 0.5 | 0.05 | FALSE | negligible |

**Response Table 11. Holm–Bonferroni step-down correction pairwise comparisons for Specificity of trained MLP models under different fingerprint types.**

| Strategy | Metric | FP_A | FP_B | Mean_A | Mean_B | Δ(A-B) | t | df | p_value | Cohen's_d | P(A>B) | HB_threshold | Significant | Effect_size |
| --- | --- | --- | --- | --- | --- | --- | --- | --- | --- | --- | --- | --- | --- | --- |
| Strategy #1 | Specificity | Atompair | Topological | 0.829 | 0.865 | -0.036 | -1.319 | 17.9 | 0.2038 | -0.59 | 0.647 | 0.008 | FALSE | medium |
| Strategy #1 | Specificity | Topological | MACCS | 0.865 | 0.839 | 0.026 | 1.019 | 17.9 | 0.3216 | 0.456 | 0.614 | 0.01 | FALSE | small |
| Strategy #1 | Specificity | Morgan | Atompair | 0.848 | 0.829 | 0.019 | 0.891 | 11.6 | 0.391 | 0.399 | 0.6 | 0.0125 | FALSE | small |
| Strategy #1 | Specificity | Morgan | Topological | 0.848 | 0.865 | -0.017 | -0.844 | 11.9 | 0.4153 | -0.377 | 0.594 | 0.016 | FALSE | small |
| Strategy #1 | Specificity | Morgan | MACCS | 0.848 | 0.839 | 0.009 | 0.474 | 12.3 | 0.6436 | 0.212 | 0.553 | 0.025 | FALSE | small |
| Strategy #1 | Specificity | Atompair | MACCS | 0.829 | 0.839 | -0.01 | -0.378 | 17.7 | 0.7098 | -0.169 | 0.542 | 0.05 | FALSE | negligible |
| Strategy #2 | Specificity | Topological | MACCS | 0.87 | 0.823 | 0.047 | 2.293 | 17 | 0.0348 | 1.026 | 0.756 | 0.008 | FALSE | large |
| Strategy #2 | Specificity | Atompair | MACCS | 0.855 | 0.823 | 0.032 | 1.431 | 18 | 0.1696 | 0.64 | 0.66 | 0.01 | FALSE | medium |
| Strategy #2 | Specificity | Morgan | MACCS | 0.851 | 0.823 | 0.028 | 1.022 | 16.5 | 0.3214 | 0.457 | 0.614 | 0.0125 | FALSE | small |
| Strategy #2 | Specificity | Atompair | Topological | 0.855 | 0.87 | -0.015 | -0.75 | 17.3 | 0.4634 | -0.335 | 0.584 | 0.016 | FALSE | small |
| Strategy #2 | Specificity | Morgan | Topological | 0.851 | 0.87 | -0.019 | -0.745 | 14.3 | 0.4682 | -0.333 | 0.583 | 0.025 | FALSE | small |
| Strategy #2 | Specificity | Morgan | Atompair | 0.851 | 0.855 | -0.004 | -0.148 | 16.1 | 0.8842 | -0.066 | 0.517 | 0.05 | FALSE | negligible |
| Strategy #3 | Specificity | Atompair | Topological | 0.889 | 0.895 | -0.006 | -0.51 | 14.6 | 0.6178 | -0.228 | 0.557 | 0.008 | FALSE | small |
| Strategy #3 | Specificity | Topological | MACCS | 0.895 | 0.891 | 0.004 | 0.356 | 15.2 | 0.7266 | 0.159 | 0.54 | 0.01 | FALSE | negligible |
| Strategy #3 | Specificity | Morgan | Topological | 0.891 | 0.895 | -0.004 | -0.332 | 14.4 | 0.7445 | -0.149 | 0.537 | 0.0125 | FALSE | negligible |
| Strategy #3 | Specificity | Atompair | MACCS | 0.889 | 0.891 | -0.002 | -0.144 | 17.9 | 0.887 | -0.064 | 0.516 | 0.016 | FALSE | negligible |
| Strategy #3 | Specificity | Morgan | Atompair | 0.891 | 0.889 | 0.002 | 0.138 | 18 | 0.8921 | 0.062 | 0.515 | 0.025 | FALSE | negligible |
| Strategy #3 | Specificity | Morgan | MACCS | 0.891 | 0.891 | 0 | 0 | 17.8 | 1 | 0 | 0.5 | 0.05 | FALSE | negligible |

**Response Table 12. Holm–Bonferroni step-down correction pairwise comparisons for F1 Score of trained MLP models under different fingerprint types.**

| Strategy | Metric | FP_A | FP_B | Mean_A | Mean_B | Δ(A-B) | t | df | p_value | Cohen's_d | P(A>B) | HB_threshold | Significant | Effect_size |
| --- | --- | --- | --- | --- | --- | --- | --- | --- | --- | --- | --- | --- | --- | --- |
| Strategy #1 | F1 | Atompair | MACCS | 0.819 | 0.85 | -0.031 | -1.146 | 18 | 0.2669 | -0.512 | 0.628 | 0.008 | FALSE | medium |
| Strategy #1 | F1 | Atompair | Topological | 0.819 | 0.842 | -0.023 | -0.944 | 16.9 | 0.3582 | -0.422 | 0.606 | 0.01 | FALSE | small |
| Strategy #1 | F1 | Morgan | Atompair | 0.838 | 0.819 | 0.019 | 0.768 | 17.2 | 0.453 | 0.343 | 0.586 | 0.0125 | FALSE | small |
| Strategy #1 | F1 | Morgan | MACCS | 0.838 | 0.85 | -0.012 | -0.49 | 17.3 | 0.6304 | -0.219 | 0.555 | 0.016 | FALSE | small |
| Strategy #1 | F1 | Topological | MACCS | 0.842 | 0.85 | -0.008 | -0.332 | 17 | 0.744 | -0.148 | 0.537 | 0.025 | FALSE | negligible |
| Strategy #1 | F1 | Morgan | Topological | 0.838 | 0.842 | -0.004 | -0.186 | 18 | 0.8543 | -0.083 | 0.521 | 0.05 | FALSE | negligible |
| Strategy #2 | F1 | Topological | MACCS | 0.87 | 0.846 | 0.024 | 2.011 | 15.1 | 0.0625 | 0.899 | 0.725 | 0.008 | FALSE | large |
| Strategy #2 | F1 | Morgan | MACCS | 0.871 | 0.846 | 0.025 | 1.976 | 16.7 | 0.0649 | 0.884 | 0.721 | 0.01 | FALSE | large |
| Strategy #2 | F1 | Atompair | MACCS | 0.86 | 0.846 | 0.014 | 1.041 | 17.7 | 0.3118 | 0.466 | 0.616 | 0.0125 | FALSE | small |
| Strategy #2 | F1 | Morgan | Atompair | 0.871 | 0.86 | 0.011 | 0.943 | 17.6 | 0.3583 | 0.422 | 0.605 | 0.016 | FALSE | small |
| Strategy #2 | F1 | Atompair | Topological | 0.86 | 0.87 | -0.01 | -0.919 | 16.3 | 0.3715 | -0.411 | 0.603 | 0.025 | FALSE | small |
| Strategy #2 | F1 | Morgan | Topological | 0.871 | 0.87 | 0.001 | 0.101 | 17.4 | 0.9205 | 0.045 | 0.511 | 0.05 | FALSE | negligible |
| Strategy #3 | F1 | Topological | MACCS | 0.9 | 0.895 | 0.005 | 0.436 | 13.7 | 0.6694 | 0.195 | 0.549 | 0.008 | FALSE | negligible |
| Strategy #3 | F1 | Atompair | Topological | 0.896 | 0.9 | -0.004 | -0.349 | 13.7 | 0.7323 | -0.156 | 0.539 | 0.01 | FALSE | negligible |
| Strategy #3 | F1 | Morgan | Topological | 0.897 | 0.9 | -0.003 | -0.29 | 14.8 | 0.7761 | -0.13 | 0.532 | 0.0125 | FALSE | negligible |
| Strategy #3 | F1 | Morgan | MACCS | 0.897 | 0.895 | 0.002 | 0.149 | 17.7 | 0.8834 | 0.067 | 0.517 | 0.016 | FALSE | negligible |
| Strategy #3 | F1 | Morgan | Atompair | 0.897 | 0.896 | 0.001 | 0.074 | 17.7 | 0.9416 | 0.033 | 0.508 | 0.025 | FALSE | negligible |
| Strategy #3 | F1 | Atompair | MACCS | 0.896 | 0.895 | 0.001 | 0.07 | 18 | 0.9451 | 0.031 | 0.508 | 0.05 | FALSE | negligible |

**Response Table 13. Holm–Bonferroni step-down correction pairwise comparisons for Geometric Mean of trained MLP models under different fingerprint types.**

| Strategy | Metric | FP_A | FP_B | Mean_A | Mean_B | Δ(A-B) | t | df | p_value | Cohen's_d | P(A>B) | HB_threshold | Significant | Effect_size |
| --- | --- | --- | --- | --- | --- | --- | --- | --- | --- | --- | --- | --- | --- | --- |
| Strategy #1 | GM | Atompair | Topological | 0.818 | 0.849 | -0.031 | -1.255 | 17.4 | 0.226 | -0.561 | 0.64 | 0.008 | FALSE | medium |
| Strategy #1 | GM | Morgan | Atompair | 0.839 | 0.818 | 0.021 | 0.956 | 14.5 | 0.3547 | 0.428 | 0.607 | 0.01 | FALSE | small |
| Strategy #1 | GM | Atompair | MACCS | 0.818 | 0.842 | -0.024 | -0.917 | 18 | 0.3713 | -0.41 | 0.603 | 0.0125 | FALSE | small |
| Strategy #1 | GM | Morgan | Topological | 0.839 | 0.849 | -0.01 | -0.518 | 16.1 | 0.6114 | -0.232 | 0.558 | 0.016 | FALSE | small |
| Strategy #1 | GM | Topological | MACCS | 0.849 | 0.842 | 0.007 | 0.292 | 17.7 | 0.7737 | 0.131 | 0.533 | 0.025 | FALSE | negligible |
| Strategy #1 | GM | Morgan | MACCS | 0.839 | 0.842 | -0.003 | -0.142 | 14.9 | 0.8891 | -0.063 | 0.516 | 0.05 | FALSE | negligible |
| Strategy #2 | GM | Topological | MACCS | 0.866 | 0.83 | 0.036 | 2.471 | 13.4 | 0.0276 | 1.105 | 0.776 | 0.008 | FALSE | large |
| Strategy #2 | GM | Atompair | MACCS | 0.854 | 0.83 | 0.024 | 1.391 | 17.7 | 0.1815 | 0.622 | 0.656 | 0.01 | FALSE | medium |
| Strategy #2 | GM | Morgan | MACCS | 0.856 | 0.83 | 0.026 | 1.334 | 17.8 | 0.199 | 0.597 | 0.649 | 0.0125 | FALSE | medium |
| Strategy #2 | GM | Atompair | Topological | 0.854 | 0.866 | -0.012 | -0.911 | 14.5 | 0.3775 | -0.407 | 0.602 | 0.016 | FALSE | small |
| Strategy #2 | GM | Morgan | Topological | 0.856 | 0.866 | -0.01 | -0.625 | 12.6 | 0.5429 | -0.28 | 0.57 | 0.025 | FALSE | small |
| Strategy #2 | GM | Morgan | Atompair | 0.856 | 0.854 | 0.002 | 0.108 | 17 | 0.915 | 0.048 | 0.512 | 0.05 | FALSE | negligible |
| Strategy #3 | GM | Atompair | Topological | 0.891 | 0.897 | -0.006 | -0.517 | 14.2 | 0.6133 | -0.231 | 0.558 | 0.008 | FALSE | small |
| Strategy #3 | GM | Topological | MACCS | 0.897 | 0.892 | 0.005 | 0.452 | 14.7 | 0.6579 | 0.202 | 0.551 | 0.01 | FALSE | small |
| Strategy #3 | GM | Morgan | Topological | 0.893 | 0.897 | -0.004 | -0.362 | 14.7 | 0.7228 | -0.162 | 0.54 | 0.0125 | FALSE | negligible |
| Strategy #3 | GM | Morgan | Atompair | 0.893 | 0.891 | 0.002 | 0.144 | 17.9 | 0.887 | 0.064 | 0.516 | 0.016 | FALSE | negligible |
| Strategy #3 | GM | Morgan | MACCS | 0.893 | 0.892 | 0.001 | 0.075 | 18 | 0.9414 | 0.033 | 0.508 | 0.025 | FALSE | negligible |
| Strategy #3 | GM | Atompair | MACCS | 0.891 | 0.892 | -0.001 | -0.072 | 17.9 | 0.9433 | -0.032 | 0.508 | 0.05 | FALSE | negligible |

**Response Table 14. Holm–Bonferroni step-down correction pairwise comparisons for IBA of trained MLP models under different fingerprint types.**

| Strategy | Metric | FP_A | FP_B | Mean_A | Mean_B | Δ(A-B) | t | df | p_value | Cohen's_d | P(A>B) | HB_threshold | Significant | Effect_size |
| --- | --- | --- | --- | --- | --- | --- | --- | --- | --- | --- | --- | --- | --- | --- |
| Strategy #1 | IBA | Atompair | Topological | 0.673 | 0.721 | -0.048 | -1.175 | 17.5 | 0.2558 | -0.525 | 0.631 | 0.008 | FALSE | medium |
| Strategy #1 | IBA | Atompair | MACCS | 0.673 | 0.713 | -0.04 | -0.908 | 18 | 0.3759 | -0.406 | 0.602 | 0.01 | FALSE | small |
| Strategy #1 | IBA | Morgan | Atompair | 0.704 | 0.673 | 0.031 | 0.847 | 14.8 | 0.4106 | 0.379 | 0.595 | 0.0125 | FALSE | small |
| Strategy #1 | IBA | Morgan | Topological | 0.704 | 0.721 | -0.017 | -0.525 | 16.4 | 0.6067 | -0.235 | 0.559 | 0.016 | FALSE | small |
| Strategy #1 | IBA | Morgan | MACCS | 0.704 | 0.713 | -0.009 | -0.248 | 14.9 | 0.8078 | -0.111 | 0.528 | 0.025 | FALSE | negligible |
| Strategy #1 | IBA | Topological | MACCS | 0.721 | 0.713 | 0.008 | 0.197 | 17.5 | 0.8461 | 0.088 | 0.522 | 0.05 | FALSE | negligible |
| Strategy #2 | IBA | Topological | MACCS | 0.75 | 0.692 | 0.058 | 2.412 | 13.2 | 0.031 | 1.079 | 0.77 | 0.008 | FALSE | large |
| Strategy #2 | IBA | Morgan | MACCS | 0.736 | 0.692 | 0.044 | 1.415 | 18 | 0.1741 | 0.633 | 0.658 | 0.01 | FALSE | medium |
| Strategy #2 | IBA | Atompair | MACCS | 0.73 | 0.692 | 0.038 | 1.335 | 17.6 | 0.1989 | 0.597 | 0.649 | 0.0125 | FALSE | medium |
| Strategy #2 | IBA | Atompair | Topological | 0.73 | 0.75 | -0.02 | -0.929 | 14.4 | 0.3683 | -0.415 | 0.604 | 0.016 | FALSE | small |
| Strategy #2 | IBA | Morgan | Topological | 0.736 | 0.75 | -0.014 | -0.562 | 12.9 | 0.5835 | -0.252 | 0.563 | 0.025 | FALSE | small |
| Strategy #2 | IBA | Morgan | Atompair | 0.736 | 0.73 | 0.006 | 0.206 | 17.4 | 0.8395 | 0.092 | 0.523 | 0.05 | FALSE | negligible |
| Strategy #3 | IBA | Atompair | Topological | 0.796 | 0.805 | -0.009 | -0.439 | 13.9 | 0.6677 | -0.196 | 0.549 | 0.008 | FALSE | negligible |
| Strategy #3 | IBA | Topological | MACCS | 0.805 | 0.797 | 0.008 | 0.406 | 14.4 | 0.6905 | 0.182 | 0.545 | 0.01 | FALSE | negligible |
| Strategy #3 | IBA | Morgan | Topological | 0.799 | 0.805 | -0.006 | -0.309 | 14.5 | 0.7617 | -0.138 | 0.535 | 0.0125 | FALSE | negligible |
| Strategy #3 | IBA | Morgan | Atompair | 0.799 | 0.796 | 0.003 | 0.122 | 17.9 | 0.9043 | 0.055 | 0.514 | 0.016 | FALSE | negligible |
| Strategy #3 | IBA | Morgan | MACCS | 0.799 | 0.797 | 0.002 | 0.084 | 18 | 0.9343 | 0.037 | 0.509 | 0.025 | FALSE | negligible |
| Strategy #3 | IBA | Atompair | MACCS | 0.796 | 0.797 | -0.001 | -0.04 | 17.9 | 0.9683 | -0.018 | 0.505 | 0.05 | FALSE | negligible |
