# Supplementary material for: Target-driven machine learning–enabled virtual screening (TAME-VS) enables prioritization of AKR1C3 inhibitors
Source: Commun Chem. 2026 Jun 15;9:311. doi: 10.1038/s42004-026-02092-6 (PMC13631243; doi:10.1038/s42004-026-02092-6)
Supplement: Supplementary file 6 [file 42004_2026_2092_MOESM5_ESM.zip › 20260530_Supplementary_Data_2_Spectroscopic_Characterization_of_Synthesized_Compounds.docx]

**Table of Contents**

1. ^1^H NMR spectra**………………………………………………………………………………**2
2. ^13^C NMR spectra**………………………………………………………………………...….**23
3. HPLC chromatograms**……………………………………………………………………...**44
4. HRMS spectrometry**………………………………………………………………………..**58

**^1^H NMR spectra**

^1^H NMR of compound **S35-1001**

^1^H NMR of compound **S35-1003.**

^1^H NMR of compound **S35-1005.**

^1^H NMR of compound **S35-1006.**

^1^H NMR of compound **S35-1008.**

^1^H NMR of compound **S35-1011.**

^1^H NMR of compound **S35-1012.**

^1^H NMR of compound **S35-1014.**

^1^H NMR of compound **S35-1015.**

^1^H NMR of compound **S35-1016.**

^1^H NMR of compound **S35-1017.**

^1^H NMR of compound **S35-1018.**

^1^H NMR of compound **S35-1019.**

^1^H NMR of compound **S35-1020.**

^1^H NMR of compound **S35-1021.**

^1^H NMR of compound **S35-1023.**

^1^H NMR of compound **S35-1024.**

^1^H NMR of compound **S34-1001.**

^1^H NMR of compound **S34-1002.**

^1^H NMR of compound **S34-1003.**

^1^H NMR of compound **S34-1004.**

^1^H NMR of compound **S34-1005.**

^1^H NMR of compound **S34-1006.**

^1^H NMR of compound **S34-1007.**

^1^H NMR of compound **S34-1010.**

^1^H NMR of compound **S34-1013.**

^1^H NMR of compound **S34-1021.**

^1^H NMR of compound **S34-1022.**

^1^H NMR of compound **S34-1023.**

^1^H NMR of compound **S34-1024.**

^1^H NMR of compound **S34-1025.**

^1^H NMR of compound **S34-1030.**

^1^H NMR of compound **S34-1031.**

^1^H NMR of compound **S34-1032.**

^1^H NMR of compound **S34-1033.**

^1^H NMR of compound **S34-1034.**

^1^H NMR of compound **S34-1035.**

^1^H NMR of compound **S34-1036.**

^1^H NMR of compound **S34-1037.**

^1^H NMR of compound **S34-1038.**

^1^H NMR of compound **S34-1040.**

**^13^C NMR spectra**

^13^C NMR of compound **S35-1001**

^13^C NMR of compound **S35-1003.**

^13^C NMR of compound **S35-1005.**

^13^C NMR of compound **S35-1006.**

^13^C NMR of compound **S35-1008.**

^13^C NMR of compound **S35-1011.**

^13^C NMR of compound **S35-1012.**

^13^C NMR of compound **S35-1014.**

^13^C NMR of compound **S35-1015.**

^13^C NMR of compound **S35-1016.**

^13^C NMR of compound **S35-1017.**

^13^C NMR of compound **S35-1018.**

^13^C NMR of compound **S35-1019.**

^13^C NMR of compound **S35-1020.**

^13^C NMR of compound **S35-1021.**

^13^C NMR of compound **S35-1023.**

^13^C NMR of compound **S35-1024.**

^13^C NMR of compound **S34-1001.**

^13^C NMR of compound **S34-1002.**

^13^C NMR of compound **S34-1003.**

^13^C NMR of compound **S34-1004.**

^13^C NMR of compound **S34-1005.**

^13^C NMR of compound **S34-1006.**

^13^C NMR of compound **S34-1007.**

^13^C NMR of compound **S34-1010.**

^13^C NMR of compound **S34-1013.**

^13^C NMR of compound **S34-1021.**

^13^C NMR of compound **S34-1022.**

^13^C NMR of compound **S34-1023.**

^13^C NMR of compound **S34-1024.**

^13^C NMR of compound **S34-1025.**

^13^C NMR of compound **S34-1030.**

^13^C NMR of compound **S34-1031.**

^13^C NMR of compound **S34-1032.**

^13^C NMR of compound **S34-1033.**

^13^C NMR of compound **S34-1034.**

^13^C NMR of compound **S34-1035.**

^13^C NMR of compound **S34-1036.**

^13^C NMR of compound **S34-1037.**

^13^C NMR of compound **S34-1038.**

^13^C NMR of compound **S34-1040.**

**HPLC chromatograms**

HPLC of compound **S35-1001.** t_R_ = 1.828 min, 100%.

HPLC of compound **S35-1003.** t_R_ = 4.908 min, 97.420%.

HPLC of compound **S35-1005.** t_R_ = 5.797 min, 98.954%.

HPLC of compound **S35-1006.** t_R_ = 6.382 min, 99.359%.

HPLC of compound **S35-1008.** t_R_ = 7.500 min, 100%.

HPLC of compound **S35-1011.** t_R_ = 4.560 min, 95.725%.

HPLC of compound **S35-1012.** t_R_ = 2.720 min, 97.323%.

HPLC of compound **S35-1014.** t_R_ = 4.460 min, 99.788%.

HPLC of compound **S35-1015.** t_R_ = 6.472 min, 99.936%.

HPLC of compound **S35-1016.** t_R_ = 4.118 min, 98.753%.

HPLC of compound **S35-1017.** t_R_ = 4.552 min, 95.822%.

HPLC of compound **S35-1018.** t_R_ = 4.055 min, 100%.

HPLC of compound **S35-1019.** t_R_ = 4.550 min, 99.063%..

HPLC of compound **S35-1020.** t_R_ = 3.983 min, 99.671 %.

HPLC of compound **S35-1021.** t_R_ = 3.920 min, 100%.

HPLC of compound **S35-1023.** t_R_ = 4.562 min, 96.742%.

HPLC of compound **S35-1024.** t_R_ = 5.763 min, 98.145%.

HPLC of compound **S34-1001.** t_R_ = 5.275 min, 99.418%.

HPLC of compound **S34-1002.** t_R_ = 5.576 min, 100.000%.

HPLC of compound **S34-1003.** t_R_ = 5.432 min, 100.000%.

HPLC of compound **S34-1004.** t_R_ = 4.367 min, 100.000%.

HPLC of compound **S34-1005.** t_R_ = 4.462 min, 100.000%.

HPLC of compound **S34-1006.** t_R_ = 5.057 min, 100.000%.

HPLC of compound **S34-1007.** t_R_ = 4.688 min, 100.000%.

HPLC of compound **S34-1010.** t_R_ = 4.004 min, 98.933%.

HPLC of compound **S34-1013.** t_R_ = 9.927 min, 99.901%.

HPLC of compound **S34-1021.** t_R_ = 4.252 min, 100.000%.

HPLC of compound **S34-1022.** t_R_ = 6.383 min, 100.000%.

HPLC of compound **S34-1023.** t_R_ = 6.464 min, 98.565%.

HPLC of compound **S34-1024.** t_R_ = 3.986 min, 100.000%.

HPLC of compound **S34-1025.** t_R_ = 4.016 min, 100.000%.

HPLC of compound **S34-1030.** t_R_ = 5.159 min, 99.515%.

HPLC of compound **S34-1031.** t_R_ = 4.833 min, 100.000%.

HPLC of compound **S34-1032.** t_R_ = 5.833 min, 99.613%.

HPLC of compound **S34-1033.** t_R_ = 5.788 min, 99.101%.

HPLC of compound **S34-1034.** t_R_ = 7.211 min, 97.724%.

HPLC of compound **S34-1035.** t_R_ = 4.986 min, 100.000%.

HPLC of compound **S34-1036.** t_R_ = 6.327 min, 97.518%.

HPLC of compound **S34-1037.** t_R_ = 4.918 min, 100.000%.

HPLC of compound **S34-1038.** t_R_ = 5.364 min, 99.574%.

HPLC of compound **S34-1040.** t_R_ = 5.771 min, 98.326%.

**HRMS spectrometry**

HRMS of compound **S35-1001**. ESI-HRMS (*m*/*z*): [M + H]^+^ calcd for C_20_H_18_N_2_O_5_, 367.1288, found, 367.1289.

HRMS of compound **S35-1003**. ESI-HRMS (*m*/*z*): [M + H]^+^ calcd for C_19_H_18_N_2_O_4_, 339.1399, found, 339.1340.

HRMS of compound **S35-1005**. ESI-HRMS (*m*/*z*): [M + H]^+^ calcd for C_23_H_25_N_3_O_4_, 408.1918, found, 408.1919.

HRMS of compound **S35-1006**. ESI-HRMS (*m*/*z*): [M + H]^+^ calcd for C_23_H_25_N_3_O_4_, 408.1918, found, 408.1919.

HRMS of compound **S35-1008**. ESI-HRMS (*m*/*z*): [M + H]^+^ calcd for C_19_H_18_N_2_O_4_, 421.2235, found, 421.2234.

HRMS of compound **S35-1011**. ESI-HRMS (*m*/*z*): [M + H]^+^ calcd for C_24_H_27_N_3_O_4_, 422.2074, found, 422.2075.

HRMS of compound **S35-1012**. ESI-HRMS (*m*/*z*): [M + H] ^+^ calcd for C_21_H_22_N_2_O_4_, 367.1652, found, 367.1653.

HRMS of compound **S35-1014**. ESI-HRMS (*m/z*): [M + H]^+^ calcd for C_18_H_17_N_3_O_3_, 324.1343, found, 324.1343.

HRMS of compound **S35-1015**. ESI-HRMS (*m/z*): [M + H]^+^ calcd for C_18_H_17_N_3_O_3_, 324.1343, found, 324.1344.

HRMS of compound **S35-1016**. ESI-HRMS (*m/z*): [M + H] ^+^ calcd for C_18_H_17_N_3_O_3_, 324.1343, found, 324.1345.

HRMS of compound **S35-1017**. ESI-HRMS (*m/z*): [M + Na] ^+^ calcd for C_17_H_16_N_4_O_3_, 347.1115, found, 347.1116.

HRMS of compound **S35-1018**. ESI-HRMS (*m/z*): [M + H] ^+^ calcd for C_17_H_16_N_4_O_3_, 325.1295, found, 325.1296.

HRMS of compound **S35-1019**. ESI-HRMS (*m/z*): [M + Na] ^+^ calcd for C_17_H_16_N_4_O_3_, 347.1115, found, 347.1099.

HRMS of compound **S35-1020**. ESI-HRMS (*m/z*): [M + H] ^+^ calcd for C_17_H_16_N_4_O_3_, 325.1295, found, 325.1295.

HRMS of compound **S35-1021**. ESI-HRMS (*m/z*): [M + H] ^+^ calcd for C_17_H_16_N_4_O_3_, 325.1295, found, 325.1296.

HRMS of compound **S35-1023**. ESI-HRMS (*m/z*): [M + H] ^+^ calcd for C_30_H_34_N_4_O_6_, 547.2551, found, 547.2551.

HRMS of compound **S35-1024**. ESI-HRMS (*m/z*): [M + H] ^+^ calcd for C_32_H_38_N_4_O_6_, 575.2864, found, 575.2863.

HRMS of compound **S34-1001**. ESI-HRMS (*m*/*z*): [M + H]^+^ calcd for C_15_H_16_N_2_O_2_, 257.1285, found, 257.1285.

HRMS of compound **S34-1002.** ESI-HRMS (*m*/*z*): [M + H]^+^ calcd for C_15_H_16_N_2_O_2_, 257.1285, found, 257.1286.

HRMS of compound **S34-1003.** ESI-HRMS (*m*/*z*): [M + H]^+^ calcd for C_15_H_16_N_2_O_2_, 257.1285, found, 257.1284.

HRMS of compound **S34-1004.** ESI-HRMS (*m/z*): [M + H]^+^ calcd for C_15_H_16_N_2_O_3_: 273.1234, found 273.1234.

HRMS of compound **S34-1005.** ESI-HRMS (*m/z*): [M + H]^+^ calcd for C_15_H_16_N_2_O_3_: 273.1234, found 273.1231.

HRMS of compound **S34-1006.** ESI-HRMS (*m/z*): [M + H]^+^ calcd for C_15_H_16_N_2_O_3_: 273.1234, found 273.12.

HRMS of compound **S34-1007.** ESI-HRMS (*m/z*): [M + H]^+^ calcd for C_14_H_13_FN_2_O: 261.1034, found 261.1031.

HRMS of compound **S34-1010.** ESI-HRMS (*m/z*): [M + H]^+^ calcd for C_15_H_13_N_3_O_2_: 268.1081, found 268.1079.

HRMS of compound **S34-1013.** ESI-HRMS (*m/z*): [M + H]^+^ calcd for C_18_H_22_N_2_O_2_: 299.1754, found 299.1754.

HRMS of compound **S34-1021.** ESI-HRMS (*m/z*): [M + H]^+^ calcd for C_15_H_14_N_2_O_4_: 287.1026, found 287.1022.

HRMS of compound **S34-1022.** ESI-HRMS (*m/z*): [M + H]^+^ calcd for C_18_H_16_N_2_O_2_: 293.1285, found 293.1286.

HRMS of compound **S34-1023.** ESI-HRMS (*m/z*): [M + H]^+^ calcd for C_18_H_16_N_2_O_2_: 293.1285, found 293.1283.

HRMS of compound **S34-1024.** ESI-HRMS (*m/z*): [M + H]^+^ calcd for C_16_H_15_N_3_O_2:_ 282.1237, found 282.1239.

HRMS of compound **S34-1025.** ESI-HRMS (*m/z*): [M - H]^-^ calcd for C_16_H_15_N_3_O_2_: 280.1091, found 280.1089.

HRMS of compound **S34-1030.** ESI-HRMS (*m/z*): [M + H]^+^ calcd for C_12_H_9_N_3_O_2_: 228.0768, found 228.0770.

HRMS of compound **S34-1031.** ESI-HRMS (*m/z*): [M + H]^+^ calcd for C_16_H_15_N_3_O_2_: 282.1237, found 282.1236.

HRMS of compound **S34-1032.** ESI-HRMS (*m/z*): [M + H]^+^ calcd for C_17_H_17_N_3_O_2_: 296.1394, found 296.1390.

HRMS of compound **S34-1033.** ESI-HRMS (*m/z*): [M + H]^+^ calcd for C_17_H_17_N_3_O_2_: 296.1394, found 296.1389.

HRMS of compound **S34-1034.** ESI-HRMS (*m/z*): [M + H]^+^ calcd for C_20_H_15_N_3_O_2_: 330.1237, found 330.1231.

HRMS of compound **S34-1035.** ESI-HRMS (*m/z*): [M + H]^+^ calcd for C_17_H_15_N_3_O_2_: 294.1237, found 294.1234.

HRMS of compound **S34-1036.** ESI-HRMS (*m/z*): [M + H]^+^ calcd for C_18_H_17_N_3_O_2_: 308.1394, found 308.1391.

HRMS of compound **S34-1037.** ESI-HRMS (*m/z*): [M + H]^+^ calcd for C_16_H_15_N_3_O: 282.1237, found 282.1246.

HRMS of compound **S34-1038.** ESI-HRMS (*m/z*): [M + H]^+^ calcd for C_16_H_15_N_6_O: 308.1394, found 308.1394.

HRMS of compound **S34-1040.** ESI-HRMS (*m/z*): [M + H]^+^ calcd for C_18_H_17_N_3_O_2_: 308.1394, found 308.1395.
